# Organocatalytic Asymmetric [2 + 4] Cycloadditions of 3-Vinylindoles with *ortho*-Quinone Methides

**DOI:** 10.3390/molecules26216751

**Published:** 2021-11-08

**Authors:** Si-Jia Liu, Man-Su Tu, Kai-Yue Liu, Jia-Yi Chen, Shao-Fei Ni, Yu-Chen Zhang, Feng Shi

**Affiliations:** 1 School of Chemistry and Materials Science, Jiangsu Normal University, Xuzhou 221116, China; yoghourt1128@163.com (S.-J.L.); manmandexiaowu1987@163.com (M.-S.T.); L15383434496@163.com (K.-Y.L.); 2 Department of Chemistry, Key Laboratory for Preparation and Application of Ordered Structural Materials of Guangdong Province, Shantou University, Shantou 515063, China; 19jychen4@stu.edu.cn

**Keywords:** vinylindoles, cycloaddition, *ortho*-quinone methide, chiral phosphoric acid, asymmetric organocatalysis

## Abstract

Catalytic asymmetric [2 + 4] cycloadditions of 3-vinylindoles with *ortho*-quinone methides and their precursors were carried out in the presence of chiral phosphoric acid to afford a series of indole-containing chroman derivatives with structural diversity in overall high yields (up to 98%), good diastereoselectivities (up to 93:7 dr) and moderate to excellent enantioselectivities (up to 98% ee). This approach not only enriches the chemistry of catalytic asymmetric cycloadditions involving 3-vinylindoles but is also useful for synthesizing chiral chroman derivatives.

## 1. Introduction

Chiral indole derivatives are ubiquitous in biologically important natural products, pharmaceuticals and materials [1,2,3,4,5]. In recent years, vinylindoles have been recognized as versatile reactants for the synthesis of enantioenriched indole derivatives [6,7]. The 3-vinylindoles belong to a class of vinylindoles with multiple reactive sites and are widely applied in organocatalytic asymmetric cycloadditions and substitutions [8,9,10,11,12,13,14,15,16,17,18,19,20,21,22,23,24,25,26,27,28,29].

**Figure 1:**
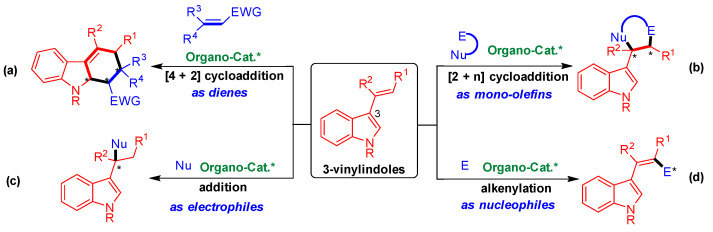
Profile of organocatalytic asymmetric reactions involving 3-vinylindoles. (**a**) [4 + 2] Cycloaddition; (**b**) [2 + n] Cycloaddition; (**c**) Addition reaction; (**d**) Alkenylation reaction. The asterisk * indicates chiral center.

As shown in Figure 1, 3-vinylindoles exhibit versatile reactivities and participate in four main types of organocatalytic asymmetric reactions. Namely, 3-vinylindoles act as dienes in asymmetric [4 + 2] cycloaddition (Figure 1a) [8,9,10,11,12], as mono-olefins in asymmetric [2 + n] cycloaddition (Figure 1b) [13,14,15,16,17,18,19,20,21,22,23], as electrophiles in asymmetric addition reaction (Figure 1c) [24,25,26,27], and as nucleophiles in asymmetric alkenylation (Figure 1d) [28,29]. Among these reactions, organocatalytic asymmetric [2 + n] cycloaddition of 3-vinylindoles as mono-olefins has proven to be an important reaction (Figure 1b) to efficiently synthesize indole-containing heterocycles with optical purity [30,31].

**Figure 2:**
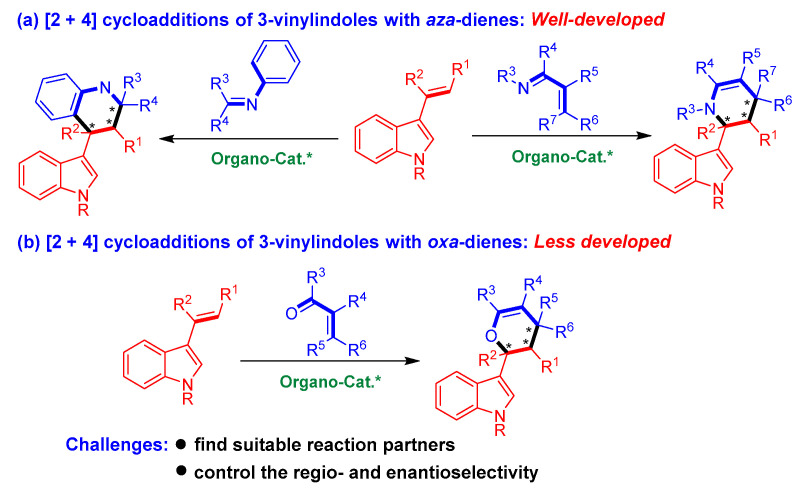
Profile of organocatalytic asymmetric [2 + 4] cycloadditions of 3-vinylindoles. The asterisk * indicates chiral center.

Among the organocatalytic asymmetric [2 + n] cycloadditions of 3-vinylindoles, [2 + 4] cycloadditions using 3-vinylindoles as dienophiles belong to a class of important inverse-electron-demand Diels−Alder reactions (Figure 2). However, most of these reactions involve [2 + 4] cycloadditions of 3-vinylindoles with *aza*-dienes (Figure 2a) [13,14,15]. By sharp contrast, organocatalytic asymmetric [2 + 4] cycloadditions of 3-vinylindoles with *oxa*-dienes have been sporadically reported in the literature (Figure 2b) [16,17,18]. The underdevelopment of this class of reactions could be ascribed to the considerable challenges encountered in conducting these reactions, which mainly include (1) finding suitable reaction partners and (2) controlling the regioselectivity (whether 3-vinylindoles act as mono-olefins or dienes) and enantioselectivity of the cycloaddition reaction.

**Figure 3:**
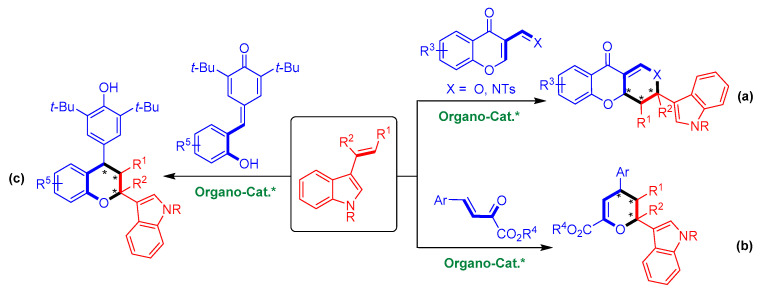
Limited examples of organocatalytic asymmetric [2 + 4] cycloadditions of 3-vinylindoles with *oxa*-dienes. (**a**) With chromone-derived *oxa*-dienes; (**b**) With β,γ-unsaturated α-ketoesters; (**c**) With *ortho*-hydroxyphenyl-substituted *para*-quinone methides. The asterisk * indicates chiral center.

To date, there are only three cases of organocatalytic asymmetric [2 + 4] cycloadditions of 3-vinylindoles with *oxa*-dienes (Figure 3) [16,17,18]. In 2011, Zhu’s group realized the organocatalytic asymmetric [2 + 4] cycloaddition of 3-vinylindoles with chromone-derived *oxa*-dienes, generating enantioenriched indole-containing heterocycles (Figure 3a) [16]. In 2019, Zhang and coworkers performed an organocatalytic asymmetric [2 + 4] cycloaddition of 3-vinylindoles with β,γ-unsaturated α-ketoesters with high enantioselectivities (Figure 3b) [17]. Very recently, our group reported an organocatalytic asymmetric [2 + 4] cycloaddition of 3-vinylindoles with *ortho*-hydroxyphenyl-substituted *para*-quinone methide derivatives that provides a series of chiral chroman derivatives bearing an indole moiety (Figure 3c) [18]. Other than these cases, organocatalytic asymmetric [2 + 4] cycloadditions of 3-vinylindoles with *oxa*-dienes remain rather limited. Therefore, developing this class of reactions and overcoming the associated inherent challenges are urgently required.

**Figure 4:**
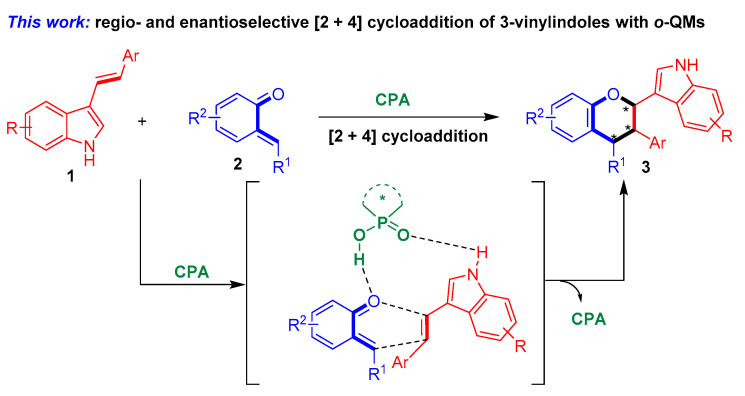
Design of CPA-catalyzed asymmetric [2 + 4] cycloaddition of 3-vinylindoles using *o*-QMs. The asterisk * indicates chiral center.

To fulfill this task and in continuation of our ongoing efforts in the enantioselective synthesis of indole-based chiral heterocycles [32,33,34,35], we designed a chiral phosphoric acid [36,37,38,39,40,41,42,43,44] (CPA)-catalyzed asymmetric [2 + 4] cycloaddition of 3-vinylindoles with *ortho*-quinone methides (*o*-QMs) and their precursors (Figure 4). The *o*-QMs were selected as suitable reaction partners because of their high reactivity as *oxa*-dienes in catalytic asymmetric cycloadditions [45,46,47,48,49,50,51,52,53,54,55,56,57,58,59,60,61,62,63]. Within this design scheme, 3-vinylindoles **1** and *o*-QMs **2** can be simultaneously activated by CPA via hydrogen-bonding interactions. This dual activation mode of CPA facilitates regioselective and enantioselective [2 + 4] cycloaddition between 3-vinylindoles **1** and *o*-QMs **2**, thus affording the chiral indole-containing chroman derivatives **3**.

Herein, we report the CPA-catalyzed asymmetric [2 + 4] cycloaddition of 3-vinylindoles with *o*-QMs and their precursors to afford chiral indole-containing chroman derivatives in overall good yields (up to 98% yield) and moderate to excellent stereoselectivities (up to 93:7 dr, 98% ee).

## 2. Results and Discussion

### 2.1. Organocatalytic Asymmetric [2 + 4] Cycloaddition of 3-Vinylindoles with Sesamol-Derived o-QMs

#### 2.1.1. Optimization of Reaction Conditions

To test the feasibility of the designed catalytic asymmetric [2 + 4] cycloaddition, 3-vinylindole **1a** was reacted with sesamol-derived *o*-QM **2a** in the presence of CPA (***R***)-**4a** at 25 °C in toluene (Table 1, entry 1). Catalytic asymmetric [2 + 4] cycloaddition occurred, as expected, to afford the chiral chroman derivative **3aa** in a moderate yield with excellent diastereoselectivity, albeit with no enantio-control (43% yield, 96:4 dr, 0% ee). To control the enantioselectivity of this [2 + 4] cycloaddition, a series of CPAs **4** were screened (entries 2–7). The CPA **(*R*)-4c** bearing two bulky 3,3′-(1-naphthyl) groups facilitated the [2 + 4] cycloaddition with the highest enantioselectivity of 66% ee (for the major diastereomer) among the investigated catalysts (entry 3 vs. entries 1–2 and 4–7), which could be ascribed to the steric hindrance effect of the 3,3′-disubstituents of CPA in creating a chiral environment for controlling the enantioselectivity [64,65]. The subsequent evaluation of a series of representative solvents (entries 8–12) in the presence of CPA **(*R*)-4c** showed that toluene remained the most suitable solvent in terms of controlling the enantioselectivity (entry 3 vs. entries 8–12).

Next, we investigated the effect of the reaction temperature (Table 2, entries 1–4) and found 0 °C to be the optimal reaction temperature (entry 1 vs. entry 3). Modulating the molar ratio of the reactants (entries 5–8) revealed that increasing the quantity of sesamol-derived *o*-QM **2a** improved the yield but decreased the enantioselectivity (entry 3 vs. entries 5–6), whereas increasing the quantity of 3-vinylindole **1a** was detrimental to the reaction (entry 3 vs. entries 7–8). Therefore, the most suitable molar reagent ratio remained 1:1.2. Finally, some additives were screened (entries 9–13), and the optimal conditions for this [2 + 4] cycloaddition were set as shown in entry 12.

#### 2.1.2. Substrate Scope

After establishing the optimal reaction conditions, we investigated the substrate scope of the 3-vinylindoles **1** for catalytic asymmetric [2 + 4] cycloadditions with sesamol-derived *o*-QM **2a**. As shown in Table 3, a variety of 3-vinylindoles **1** bearing different R/R^1^ groups underwent [2 + 4] cycloadditions to generate chiral indole-containing chroman derivatives **3** in overall good yields (54–98%) and moderate to excellent stereoselectivities (78:22 dr to 93:7 dr, 55–97% ee). In detail, C5-, C6- and C7-substituted 3-vinylindoles **1b**–**1f** participated in the [2 + 4] cycloaddition with high yields and moderate enantioselectivities (entries 2–6). In addition, a series of *ortho*-, *meta*- and *para*-substituted phenyl groups were utilized as R^1^ groups for the 3-vinylindoles **1**, and the corresponding substrates participated in [2 + 4] cycloaddition with moderate to good results (entries 7–13). Among these 3-vinylindoles, **1l**–**1m** bearing *para*-substituted phenyl groups (R^1^) delivered the corresponding products **3la**–**3ma** with the best enantioselectivities (85% ee and 97% ee, entries 12–13). Notably, these *para*-substituted substrates **1l**–**1m** displayed a much higher capability in controlling the enantioselectivity than their *ortho*- and *meta*-substituted counterparts (entries 12–13 vs. entries 7–8 and 10–11), which might be ascribed to the steric effect of the *para*-substituents.

Next, the substrate scope of sesamol-derived *o*-QMs **2** was explored by catalytic asymmetric [2 + 4] cycloaddition with 3-vinylindole **1a** (Table 4). This reaction was clearly amenable to participation by a series of sesamol-derived *o*-QMs **2a**–**2g** bearing either electron-donating or electron-withdrawing groups at different positions of the phenyl ring (entries 1−7), producing chiral indole-containing chroman derivatives **3** in generally high yields (53–98%) and moderate to excellent diastereo- and enantioselectivities (75:25 dr to 89:11 dr, 60–98% ee). Among these *o*-QMs, **2f**–**2g** bearing *para*-halogen-substituted phenyl groups delivered products **3af**–**3ag** in the highest enantioselectivities of 97–98% ee (entries 4–7). Notably, *o*-QM **2h** bearing a heteroaromatic 2-thiophenyl group could also be utilized as a reaction partner to yield the product **3ah** with a high enantioselectivity of 87% ee (entry 8).

**Figure 5:**
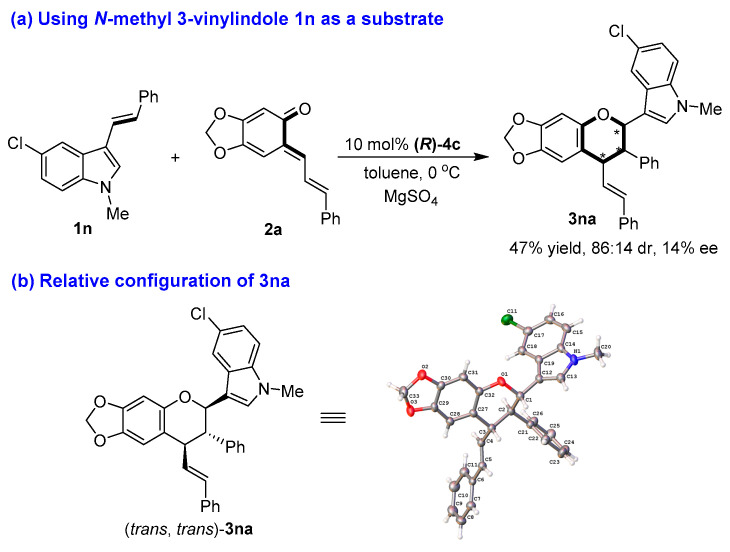
Determination of the relative configuration of **3na**. The asterisk * indicates chiral center.

The structures of all products **3** were identified by their NMR, IR and HR MS data, and the ee value of all products **3** were calculated by their HPLC traces (see the Supplementary Materials). Although we tried to cultivate the single crystal from enantioenriched products **3**, we failed to achieve this goal. So, the absolute configurations of chiral products **3** could not be determined. Nevertheless, when *N*-methyl-protected 3-vinylindole **1n** was employed as a substrate in the reaction with sesamol-derived *o*-QM **2a** under standard conditions (Figure 5a), the [2 + 4] cycloaddition occurred to generated product **3na** in a moderate yield and diastereoselectivity (47% yield, 86:14 dr) albeit with an extremely low enantioselectivity (14% ee). Fortunately, we cultivated the single crystal of product **3na**, whose relative configuration was determined to be (*trans*, *trans*) by X-ray diffraction analysis of the single crystal (CCDC 2100427, see the Supplementary Materials) (Figure 5b).

#### 2.1.3. Theoretical Calculations of the Reaction Pathway and Key Transition States

**Figure 6:**
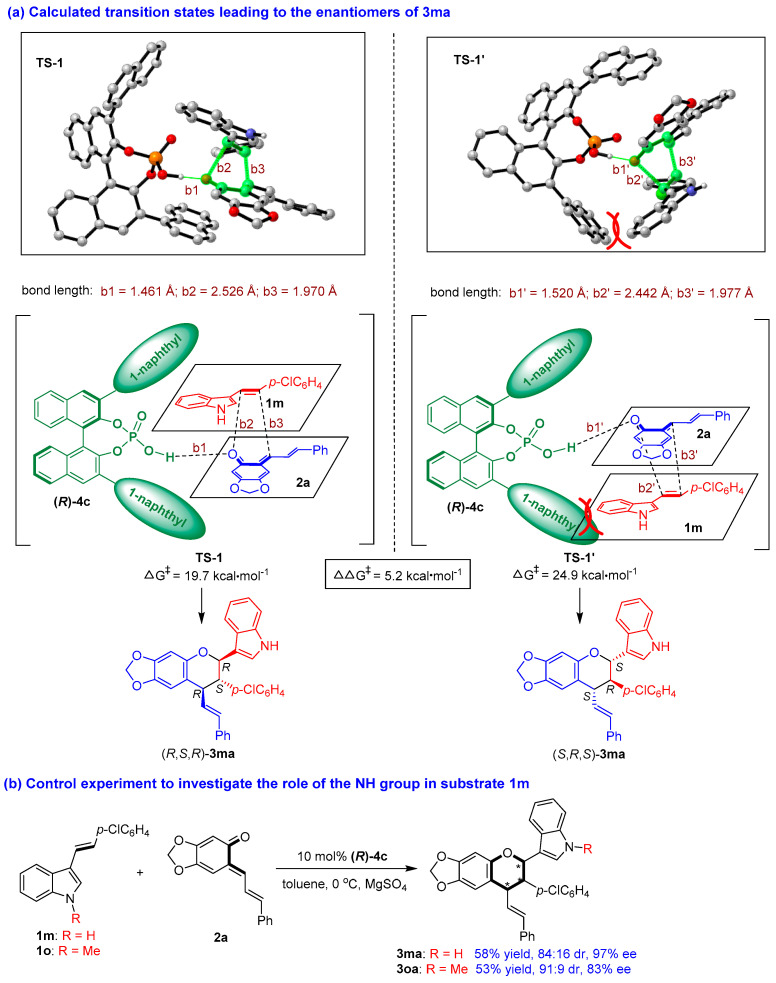
Calculated transition states leading to the enantiomers of **3ma** and control experiment. The asterisk * indicates chiral center.

To elucidate the reaction pathway and the interaction of CPA with the substrates, we carried out theoretical calculations on the reaction pathway of catalytic asymmetric [2 + 4] cycloaddition (see the Supplementary Materials) based on previous mechanistic studies [66,67]. As exemplified by the formation of product **3ma** (Figure 6a), the key transition states (TSs) and the Gibbs free energy leading to the enantiomers of **3ma** were determined, wherein **TS-1** led to the major enantiomer (*R*,*S*,*R*)-**3ma** and **TS-1′** led to the minor enantiomer (*S*,*R*,*S*)-**3ma**. DFT calculations revealed that the two bulky 3,3′-(1-naphthyl) groups and the BINOL scaffold of CPA **(*R*)-4c** formed a pocket-like chiral environment to hold the two substrates of **1m** and **2a** in a confined orientation. Specifically, in **TS-1,** 3-vinylindole **1m** was located above *o*-QM **2a** in the chiral pocket of CPA **(*R*)-4c**, wherein the space above **2a** was enough to make **1m** have little steric effect on other groups. While in **TS-1′**, **1m** was located below *o*-QM **2a**, wherein the space below **2a** was limited, thus making the phenyl group of **1m** have some steric effect on the 1-naphthyl group of **(*R*)-4c**. This steric repulsion made **TS-1′** inferior to **TS-1**, which led to the formation of the major enantiomer (*R*,*S*,*R*)-**3ma**.

In **TS-1**, CPA **(*R*)-4c** utilized its O-H group to form a strong hydrogen bond (b1 = 1.461 Å) with the C=O group of *o*-QM **2a,** but there was no discernible hydrogen-bonding interaction between CPA **(*R*)-4c** and 3-vinylindole **1m**. In addition, the calculations suggested that the [2 + 4] cycloaddition largely occurred via a concerted reaction pathway involving the formation of two new bonds (b2 = 2.526 Å, b3 = 1.970 Å). However, the longer bond length of b2 than b3 indicated that b3 (a C-C bond) formed slightly earlier than b2 (a C-O bond), which is in accordance with the reactivity of 3-vinylindole (based on the nucleophilicity of the vinyl group). In **TS-1′**, there were similar interactions between CPA **(*R*)-4c** and the substrates. However, the hydrogen bond (b1′ = 1.520 Å) between **(*R*)-4c** and **2a** in **TS-1′** was weaker than that in **TS-1** (b1 = 1.461 Å), which resulted in a significantly higher Gibbs free energy barrier for the generation of **TS-1′** (24.9 kcal/mol) compared to that for **TS-1** (19.7 kcal/mol). The calculated difference in the energy barriers for the two transition states of **TS-1′** and **TS-1** of 5.2 kcal/mol explained the excellent experimentally obtained enantioselectivity of **3ma** (97% ee).

Very interestingly, in the calculated transition states, there was no discernible hydrogen-bonding interaction between CPA **(*R*)-4c** and 3-vinylindole **1m**, which was seldom reported in CPA-catalyzed reactions involving 3-vinylindoles. To verify this issue, we performed a control experiment to investigate the role of the NH group in substrate **1m** (Figure 6b). Namely, 3-vinylindole **1o**, as *N*-methyl protected counterpart of **1m**, was employed as a substrate in the [2 + 4] cycloaddition with *o*-QM **2a** under standard conditions, which smoothly generated product **3oa** in a moderate yield of 53% with a good diastereo- and enantioselectivity (91:9 dr, 83% ee). Compared to the results of product **3ma** which was generated from *N*-unprotected 3-vinylindole **1m**, the yield and the stereoselectivity of product **3oa** were on a similar level, thus supporting the calculated activation mode that the NH group of 3-vinylindole **1m** had no discernible hydrogen-bonding interaction with CPA **(*R*)-4c**.

It should be noted that the *E*-configuration of vinylindoles **1** has been retained as *trans*-configuration in products **3** due to a concerted [2 + 4] cycloaddition pathway as illustrated in **TS-1**. So, the diastereomeric ratio of product **3** reflects the stereoselectivity of the two adjacent chiral centers generated by the two individual substrates **1** and **2**.

#### 2.1.4. Large-Scale Synthesis of Product **3aa**

**Figure 7:**
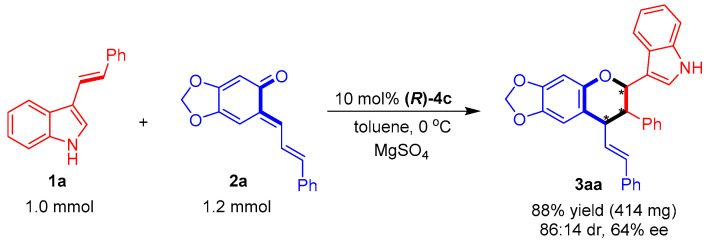
One-mmol-scale synthesis of product **3aa**. The asterisk * indicates chiral center.

Finally, the catalytic asymmetric [2 + 4] cycloaddition of **1a** with **2a** was carried out on a one mmol scale (Figure 7). The yield and stereoselectivity of this one-mmol-scale reaction were at the same level as those of the small-scale reaction (Table 3, entry 1), which implied that the catalytic asymmetric [2 + 4] cycloaddition could be scaled up.

### 2.2. Organocatalytic Asymmetric [2 + 4] Cycloaddition of 3-Vinylindoles with o-Hydroxybenzyl Alcohols

To expand the substrate scope of this organocatalytic asymmetric [2 + 4] cycloaddition, we attempted to react 3-vinylindole **1a** with *o*-hydroxybenzyl alcohol **5a** as a precursor of *o*-QM (Table 5). In the presence of CPA **(*R*)-4a** (entry 1), the desired product **6aa** was afforded in a moderate yield, albeit with a low stereoselectivity (51% yield, 67:33 dr, 42% ee). Then, a series of CPAs **(*R*)-4** were screened. Among these CPAs, **(*R*)-4e**, bearing two 3,3′-(9-anthracenyl) groups, displayed the highest catalytic activity in delivering product **6aa** with a better enantioselectivity than the other catalysts (entry 5 vs. entries 1–4 and 6–7), which could also be ascribed to the steric hindrance effect of the bulky 3,3′-disubstituents of CPA **(*R*)-4e** in controlling the enantioselectivity. Next, different solvents were evaluated in the presence of **(*R*)-4e**, revealing toluene to still be the most suitable solvent (entry 5 vs. entries 8–12). Finally, the reaction temperature was modulated (entries 13–15), and the optimal reaction conditions were set as shown in entry 14.

With the optimal conditions in hand, we investigated the substrate scope of 3-vinylindole **1** in catalytic asymmetric [2 + 4] cycloaddition with the *o*-hydroxybenzyl alcohol **5a**. As shown in Table 6, this [2 + 4] cycloaddition was amenable to participation by a wide range of 3-vinylindoles **1** bearing different R/R^1^ groups. In detail, C5-, C6- and C7-substituted 3-vinylindoles participated in the [2 + 4] cycloaddition with the *o*-hydroxybenzyl alcohol **5a** to generate the chiral indole-containing chroman derivatives **6** in moderate to good diastereo- and enantioselectivities (75:25 dr to 83:17 dr, 74–82% ee, entries 2–7). In addition, *meta*- and *para*-substituted phenyl groups were found to be suitable R^1^ groups for the 3-vinylindoles **1**, and the corresponding substrates participated in [2 + 4] cycloaddition with good results (entries 8–9).

Then, the substrate scope of *o*-hydroxybenzyl alcohols **5** was investigated for [2 + 4] cycloaddition with 3-vinylindole **1a** under standard reaction conditions. As shown in Table 7, the *o*-hydroxybenzyl alcohols **5b**–**5c** bearing a methyl group or a halogen group at the C5 position successfully participated in [2 + 4] cycloaddition with 3-vinylindole **1a**, providing products **6ab**–**6ac** in moderate to good diastereo- and enantioselectivities (68:32 dr to 81:19 dr, 73–76% ee, entries 2–3). In addition, aromatic R^1^ groups with *ortho, meta* and *para*-substituents were successfully employed in the reaction, affording products **6ad**–**6af** in overall good enantioselectivities (76–81% ee, entries 4–6).

**Figure 8:**
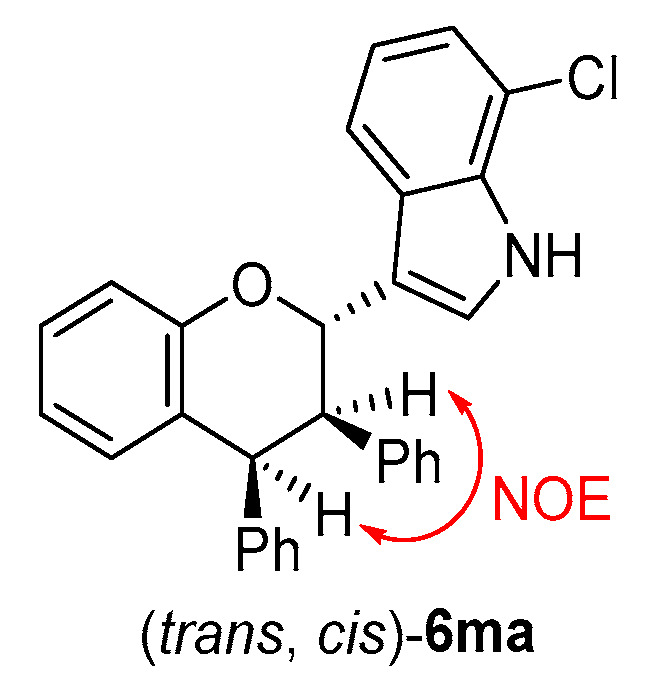
Relative configuration of **6ma**.

The structures of all products **6** were identified by their NMR, IR and HR MS data, and the ee value of all products **6** were calculated by their HPLC traces (see the Supplementary Materials). The relative configuration of product **6ma** was determined to be (*trans*, *cis*) by a NOE experiment (see the Supplementary Materials) (Figure 8) and comparing the ^1^H NMR spectra with that of a similar compound [18].

## 3. Materials and Methods

The detailed procedures for the synthesis and characterization of the products are given in Appendix A section.

## 4. Conclusions

In summary, we performed catalytic asymmetric [2 + 4] cycloaddition of 3-vinylindoles with *ortho*-quinone methides and their precursors in the presence of chiral phosphoric acid. This approach was used to synthesize a series of indole-containing chroman derivatives with structural diversity in overall high yields (up to 98%), good diastereoselectivities (up to 93:7 dr) and moderate to excellent enantioselectivities (up to 98% ee). This approach not only enriches the chemistry of 3-vinylindole-inolved catalytic asymmetric cycloadditions but is also useful for the enantioselective synthesis of chiral chroman derivatives.

## Figures and Tables

**Table 1:** Screening of catalysts and solvents *^a^*.

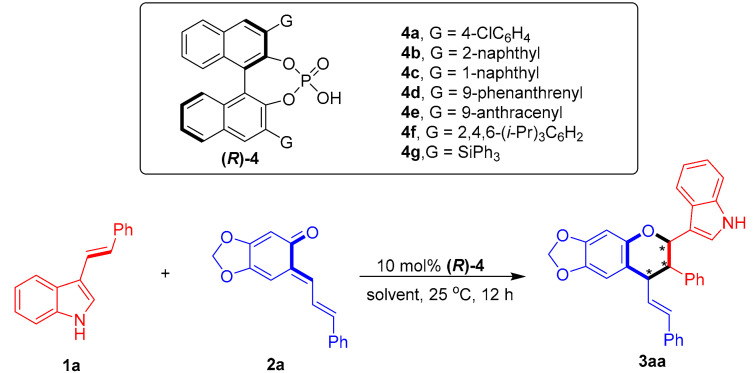					
Entry	4	Solvent	Yield (%) *^b^*	Dr *^c^*	Ee (%) *^d^*
1	**4a**	toluene	43	96:4	0
2	**4b**	toluene	44	97:3	5
3	**4c**	toluene	67	82:18	66
4	**4d**	toluene	55	90:10	0
5	**4e**	toluene	61	84:16	51
6	**4f**	toluene	32	88:12	12
7	**4g**	toluene	44	96:4	4
8	**4c**	ClCH_2_CH_2_Cl	89	86:14	62
9	**4c**	1,4-dioxane	90	78:22	42
10	**4c**	CH_3_CN	86	78:22	6
11	**4c**	EtOAc	93	80:20	40
12	**4c**	acetone	86	76:24	22

*^a^* Unless otherwise indicated, the reaction was carried out at a 0.05 mmol scale in a solvent (0.5 mL) at 25 °C for 12 h using a **1a**:**2a** molar ratio of 1:1.2. *^b^* Isolated total yield of the diastereomeric mixtures. *^c^* The diastereomeric ratio (dr) was determined by ^1^H NMR and HPLC. *^d^* The ee value refers to that of the major diastereomer and was determined by HPLC. The asterisk * indicates chiral center.

**Table 2:** Further optimization of reaction conditions *^a^*.

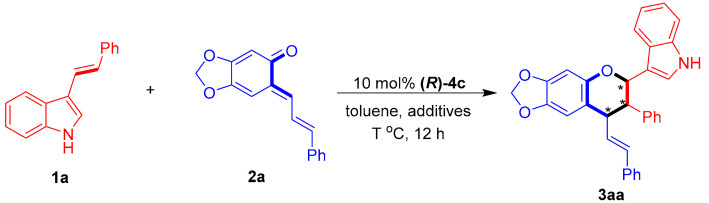						
Entry	T (°C)	1a:2a	Additives	Yield (%) *^b^*	Dr *^c^*	Ee (%) *^d^*
1	25	1:1.2	-	67	82:18	66
2	50	1:1.2	-	68	85:15	23
3	0	1:1.2		86	87:13	70
4	−10	1:1.2		79	87:13	62
5	0	1:2	-	95	87:13	59
6	0	1:3	-	97	87:13	57
7	0	2:1	-	85	88:12	63
8	0	3:1	-	81	88:12	64
9	0	1:1.2	3Å MS	88	87:13	61
10	0	1:1.2	4Å MS	89	88:12	63
11	0	1:1.2	5Å MS	86	86:14	62
12	0	1:1.2	MgSO_4_	81	89:11	72
13	0	1:1.2	Na_2_SO_4_	90	87:13	61

*^a^* Unless otherwise indicated, the reaction was carried out at a 0.05 mmol scale in toluene (0.5 mL) with additives (25 mg) for 12 h. *^b^* Isolated total yield of the diastereomeric mixtures. *^c^* The diastereomeric ratio (dr) was determined by ^1^H NMR and HPLC. *^d^* The ee value refers to that of the major diastereomer and was determined by HPLC. The asterisk * indicates chiral center.

**Table 3:** Substrate scope of 3-vinylindoles **1** *^a^*.

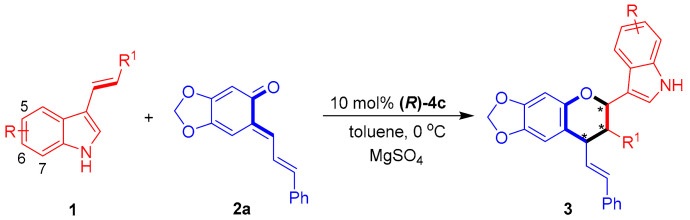					
Entry	R/R^1^ (1)	3	Yield (%) *^b^*	Dr *^c^*	Ee (%) *^d^*
1	H/Ph (**1a**)	**3aa**	81	89:11	72
2	5-Me/Ph (**1b**)	**3ba**	80	83:17	55
3	6-F/Ph (**1c**)	**3ca**	98	86:14	69
4	6-Br/Ph (**1d**)	**3da**	98	91:9	65
5	7-Me/Ph (**1e**)	**3ea**	91	85:15	59
6	7-F/Ph (**1f**)	**3fa**	98	88:12	60
7	H/*o*-MeC_6_H_4_ (**1g**)	**3ga**	60	93:7	65
8	H/*o*-ClC_6_H_4_ (**1h**)	**3ha**	62	91:9	65
9	H/*o*-BrC_6_H_4_ (**1i**)	**3ia**	96	92:8	66
10	H/*m*-MeC_6_H_4_ (**1j**)	**3ja**	84	84:16	59
11	H/*m*-ClC_6_H_4_ (**1k**)	**3ka**	98	87:13	58
12	H/*p*-MeC_6_H_4_ (**1l**)	**3la**	54	78:22	85
13	H/*p*-ClC_6_H_4_ (**1m**)	**3ma**	58	84:16	97

*^a^* Unless otherwise indicated, the reaction was carried out at a 0.1 mmol scale in toluene (1.0 mL) with MgSO_4_ (50 mg) for 12 h using a **1**:**2a** molar ratio of 1:1.2. *^b^* Isolated total yield of the diastereomeric mixtures. *^c^* The diastereomeric ratio (dr) was determined by ^1^H NMR. *^d^* The ee value refers to that of the major diastereomer and was determined by HPLC. The asterisk * indicates chiral center.

**Table 4:** Substrate scope of *o*-QMs **2** *^a^*.

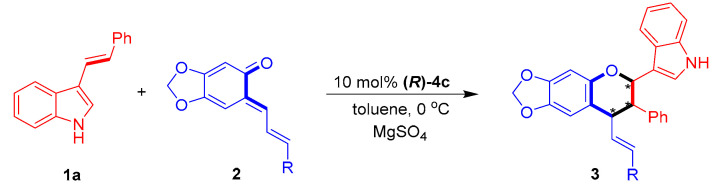					
Entry	R (2)	3	Yield (%) *^b^*	Dr *^c^*	Ee (%) *^d^*
1	Ph (**2a**)	**3aa**	81	89:11	72
2	2-MeC_6_H_4_ (**2b**)	**3ab**	98	88:12	62
3	3-MeOC_6_H_4_ (**2c**)	**3ac**	98	86:14	63
4	3-FC_6_H_4_ (**2d**)	**3ad**	98	85:15	60
5	4-MeOC_6_H_4_ (**2e**)	**3ae**	73	89:11	77
6	4-FC_6_H_4_ (**2f**)	**3af**	53	75:25	97
7	4-ClC_6_H_4_ (**2g**)	**3ag**	57	84:16	98
8	2-thiophenyl (**2h**)	**3ah**	70	85:15	87

*^a^* Unless otherwise indicated, the reaction was carried out at a 0.1 mmol scale in toluene (1.0 mL) with MgSO_4_ (50 mg) for 12 h using a **1a**:**2** molar ratio of 1:1.2. *^b^* Isolated total yield of the diastereomeric mixtures. *^c^* The diastereomeric ratio (dr) was determined by ^1^H NMR. *^d^* The ee value refers to that of the major diastereomer and was determined by HPLC. The asterisk * indicates chiral center.

**Table 5:** Optimization of reaction conditions for [2 + 4] cycloaddition of **1a** with **5a** *^a^*.

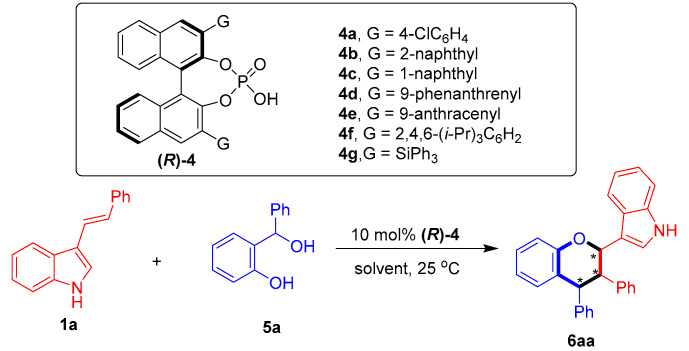					
Entry	4	Solvent	Yield (%) *^b^*	Dr *^c^*	Ee (%) *^d^*
1	**4a**	toluene	51	67:33	42
2	**4b**	toluene	65	60:40	45
3	**4c**	toluene	trace	-	-
4	**4d**	toluene	74	68:32	66
5	**4e**	toluene	59	77:23	77
6	**4f**	toluene	65	86:14	35
7	**4g**	toluene	48	76:24	44
8	**4e**	DCE	67	81:19	73
9	**4e**	THF	trace	-	-
10	**4e**	CH_3_CN	53	69:31	42
11	**4e**	EtOAc	trace	-	-
12	**4e**	acetone	trace	-	-
13 *^e^*	**4e**	toluene	63	74:26	72
14 *^f^*	**4e**	toluene	61	75:25	79
15 *^g^*	**4e**	toluene	60	72:28	79

*^a^* Unless otherwise indicated, the reaction was carried out at a 0.1 mmol scale in a solvent (0.1 mL) at 25 °C for 6 h using a **1a**:**5a** molar ratio of 1:1.2. *^b^* Isolated total yield of the diastereomeric mixtures. *^c^* The diastereomeric ratio (dr) was determined by ^1^H NMR and HPLC. *^d^* The ee value refers to that of the major diastereomer and was determined by HPLC. *^e^* At 50 °C. *^f^* At 0 °C. *^g^* At −10 °C. The asterisk * indicates chiral center.

**Table 6:** Substrate scope of 3-vinylindoles **1** for [2 + 4] cycloaddition with *o*-hydroxybenzyl alcohol **5a** *^a^*.

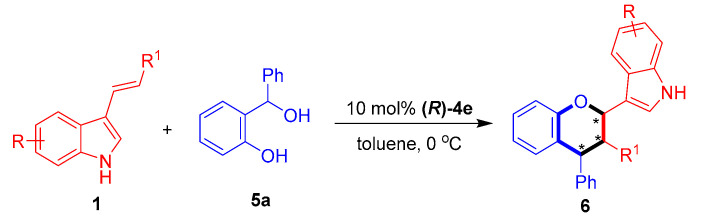					
Entry	R/R^1^ (1)	6	Yield (%) *^b^*	Dr *^c^*	Ee (%) *^d^*
1	H/Ph (**1a**)	**6aa**	61	77:23	79
2	5-Me/Ph (**1b**)	**6ba**	50	75:25	76
3	6-Cl/Ph (**1p**)	**6pa**	72	75:25	74
4	6-Br/Ph (**1d**)	**6da**	77	78:22	74
5	7-Me/Ph (**1e**)	**6ea**	53	75:25	74
6	7-Cl/Ph (**1q**)	**6qa**	90	82:18	75
7	7-Br/Ph (**1r**)	**6ra**	89	83:17	82
8	H/*m*-ClC_6_H_4_ (**1k**)	**6ka**	72	76:24	80
9	H/*p*-ClC_6_H_4_ (**1m**)	**6ma**	70	78:22	83

*^a^* Unless otherwise indicated, the reaction was carried out at a 0.1 mmol scale in toluene (1.0 mL) for 6 h using a **1**:**5a** molar ratio of 1:1.2. *^b^* Isolated total yield of the diastereomeric mixtures. *^c^* The diastereomeric ratio (dr) was determined by ^1^H NMR. *^d^* The ee value refers to that of the major diastereomer and was determined by HPLC. The asterisk * indicates chiral center.

**Table 7:** Substrate scope of *o*-hydroxybenzyl alcohols **5** for [2 + 4] cycloaddition *^a^*.

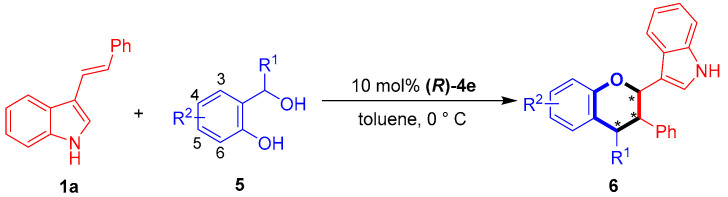					
Entry	R^1^/R^2^ (5)	6	Yield (%) *^b^*	Dr *^c^*	Ee (%) *^d^*
1	Ph/H (**5a**)	**6aa**	61	77:23	79
2	Ph/5-Me (**5b**)	**6ab**	73	81:19	76
3 *^e^*	Ph/5-Br (**5c**)	**6ac**	57	68:32	73(60) *^f^*
4	*p*-FC_6_H_4_/H (**5d**)	**6ad**	59	75:25	76
5	*m*-MeOC_6_H_4_/H (**5e**)	**6ae**	80	75:25	79
6 *^g^*	*o*-MeOC_6_H_4_/4-OMe (**5f**)	**6af**	71	90:10	81

*^a^* Unless otherwise indicated, the reaction was carried out at a 0.1 mmol scale in toluene (1.0 mL) for 6 h using a **1a**:**5** molar ratio of 1:1.2. *^b^* Isolated total yield of the diastereomeric mixtures. *^c^* The diastereomeric ratio (dr) was determined by ^1^H NMR. *^d^* The ee value refers to that of the major diastereomer and was determined by HPLC. *^e^* At 0 °C for 6 h and then 25 °C for 2 h. *^f^* The ee value of the minor diastereoisomer. *^g^* At 25 °C for 2 h. The asterisk * indicates chiral center.

## Data Availability

The data presented in this study are available in this article.

## Supplementary Materials

The following are available online. NMR and HPLC spectra of products **3** and **6**, NOE spectrum of product **6ma**, X-ray single-crystal data for product **3na**, and theoretical calculations of the reaction pathway.

## Sample Availability

Samples of the compounds are not available from the authors.

## Appendix A. Experimental Section

^1^H and ^13^C NMR spectra were measured at 400 and 100 MHz, respectively. The solvents used for NMR spectroscopy were acetone-*d*_6_ and CDCl_3_, using tetramethylsilane as the internal reference. HR MS (ESI) was determined by an HR MS/MS instrument. The X-ray source used for the single crystal X-ray diffraction analysis of compound **3na** was MoKα (λ = 0.71073), and the thermal ellipsoid was drawn at the 30% probability level. Analytical grade solvents for the column chromatography were used after distillation, and commercially available reagents were used as received. Substrates **1** were synthesized according to the literature method [17]. Substrates **2** and **5** were synthesized according to the literature method [52,68].

**General procedure for the synthesis of products** **3**

To the mixture of 3-vinylindoles **1** (0.1 mmol), *ortho*-quinone methides **2** (0.12 mmol), catalyst **(*R*)-4c** (0.01 mmol), and MgSO_4_ (50 mg) was added toluene (1 mL). Then, the reaction mixture was stirred at 0 °C for 12 h. After completion of the reaction, which was indicated by TLC, the reaction mixture was directly purified through flash column chromatography to afford products **3**.

**(*E*)-2-(7-phenyl-8-styryl-7,8-dihydro-6*H*-[1,3]dioxolo[4,5-*g*]chromen-6-yl)-1*H*-indole (3aa):**

Yield: 81% (38.0 mg); 89:11 dr; white solid; m.p. 97.6–99.0 °C; [α]_D_^20^ = −19.0 (c 0.76, acetone); ^1^H NMR (400 MHz, CDCl_3_) δ 7.88–7.80 (m, 2H), 7.26–7.22 (m, 5H), 7.20–7.18 (m, 1H), 7.17–7.12 (m, 2H), 7.07 (m, 2H), 7.03–7.00 (m, 1H), 7.00–6.96 (m, 2H), 6.79 (d, *J* = 2.4 Hz, 1H), 6.77 (s, 1H), 6.56 (s, 1H), 6.16 (d, *J* = 15.7 Hz, 1H), 6.06–5.96 (m, 1H), 5.92–5.89 (m, 2H), 5.48 (d, *J* = 10.5 Hz, 1H), 4.01–3.95 (m, 1H), 3.57–3.50 (m, 1H). ^13^C NMR (100 MHz, CDCl_3_) δ 149.8, 147.0, 141.7, 140.9, 137.1, 136.2, 132.8, 131.3, 128.4, 128.3, 128.1, 127.2, 126.4, 126.2, 125.9, 123.3, 122.2, 120.1, 119.8, 116.4, 114.4, 111.2, 108.2, 101.0, 98.8, 76.9, 50.5, 48.2; IR (KBr): 3419, 3057, 3026, 2894, 1499, 1477, 1264, 1153, 1072, 864 cm^−1^; (C_32_H_25_NO_3_-H)^−^ requires *m*/*z* 470.1761, found *m*/*z* 470.1749; ee: 72%, AD-H, hexane/isopropanol = 70/30, t_R_ = 11.147 (minor), t_R_ = 30.360 (major).

**(*E*)-5-methyl-2-(7-phenyl-8-styryl-7,8-dihydro-6*H*-[1,3]dioxolo[4,5-*g*]chromen-6-yl)-1*H*-indole (3ba):**

Yield: 80% (38.9 mg); 83:17 dr; white solid; m.p. 123.2–125.3 °C; [α]_D_^20^ = −26.1 (c 0.78, acetone); ^1^H NMR (400 MHz, CDCl_3_) δ 7.68 (s, 1H), 7.61 (s, 1H), 7.30–7.27 (m, 1H), 7.26–7.17 (m, 4H), 7.13–7.06 (m, 3H), 7.05–6.98 (m, 4H), 6.80 (s, 1H), 6.72 (d, *J* = 2.5 Hz, 1H), 6.60 (s, 1H), 6.20–6.16 (m, 1H), 6.09–6.00 (m, 1H), 5.92 (s, 2H), 5.45 (d, *J* = 10.5 Hz, 1H), 4.02–3.96 (m, 1H), 3.59–3.50 (m, 1H), 2.49 (s, 3H); ^13^C NMR (100 MHz, CDCl_3_) δ 149.8, 147.0, 141.7, 141.1, 137.2, 134.6, 132.8, 131.4, 129.2, 129.0, 128.5, 128.4, 128.3, 128.1, 128.0, 127.2, 126.5, 126.4, 126.3, 126.2, 123.8, 123.5, 119.6, 116.4, 113.7, 110.9, 108.2, 101.0, 98.9, 77.0, 50.3, 48.3, 21.6; IR (KBr): 3416, 3025, 2895, 1499, 1478, 1425, 1238, 1152, 1037, 748 cm^−1^; (C_33_H_27_NO_3_-H)^−^ requires *m*/*z* 484.1918, found *m*/*z* 484.1890; ee: 55%, IA, hexane/isopropanol = 70/30, t_R_ = 10.293 (minor), t_R_ = 30.297 (major).

(***E*)-6-fluoro-2-(7-phenyl-8-styryl-7,8-dihydro-6*H*-[1,3]dioxolo[4,5-*g*]chromen-6-yl)-1*H*-indole (3ca):**

Yield: 98% (47.8 mg); 86:14 dr; brown solid; m.p. 108.7–110.0 °C; [α]_D_^20^ = −27.8 (c 0.96, acetone); ^1^H NMR (400 MHz, CDCl_3_) δ 7.77–7.70 (m, 2H), 7.29–7.26 (m, 1H), 7.25–7.22 (m, 3H), 7.20–7.16 (m, 1H), 7.12–7.06 (m, 2H), 7.06–7.01 (m, 1H), 7.00–6.95 (m, 2H), 6.91–6.84 (m, 2H), 6.78 (s, 1H), 6.73 (d, *J* = 2.1 Hz, 1H), 6.57 (s, 1H), 6.18 (d, *J* = 15.5 Hz, 1H), 6.05–5.97 (m, 1H), 5.92–5.89 (m, 2H), 5.43 (d, *J* = 10.5 Hz, 1H), 4.03–3.95 (m, 1H), 3.53–3.43 (m, 1H); ^13^C NMR (100 MHz, CDCl_3_) δ 159.9 (d, *J* = 236.0 Hz), 149.6, 147.0, 141.8, 140.8, 137.1, 136.1 (*J* = 12.0 Hz), 132.9, 131.1, 128.4, 128.3, 128.2, 127.3, 126.5, 126.2, 123.7 (d, *J* = 2.0 Hz), 122.4, 120.9 (d, *J* = 10.0 Hz), 116.4, 114.5, 108.6 (d, *J* = 24.0 Hz), 108.2, 101.0, 98.7, 97.5 (d, *J* = 26.0 Hz), 76.9, 50.7, 48.1; IR (KBr): 3891, 3725, 3421, 1844, 1699, 1239, 1153, 862, 747 cm^−1^; (C_32_H_24_FNO_3_-H)^−^ requires *m*/*z* 488.1667, found *m*/*z* 488.1676; ee: 69%, IA, hexane/isopropanol = 70/30, t_R_ = 9.773 (major), t_R_ = 25.597 (minor).

**(*E*)-6-bromo-2-(7-phenyl-8-styryl-7,8-dihydro-6*H*-[1,3]** **dioxolo[4,5-*g*]chromen-6-yl)-1*H*-indole (3da):**

Yield: 98% (53.8 mg); 91:9 dr; brown solid; m.p. 104.5–105.1 °C; [α]_D_^20^ = −18.2 (c 1.08, acetone); ^1^H NMR (400 MHz, CDCl_3_) δ 7.73 (s, 1H), 7.67 (d, *J* = 8.5 Hz, 1H), 7.32–7.26 (m, 2H), 7.25–7.23 (m, 3H), 7.22–7.21 (m, 1H), 7.21–7.16 (m, 1H), 7.12–7.06 (m, 2H), 7.05–7.00 (m, 1H), 6.99–6.93 (m, 2H), 6.78 (s, 1H), 6.71–6.67 (m, 1H), 6.55 (s, 1H), 6.18 (d, *J* = 15.7 Hz, 1H), 6.05–5.96 (m, 1H), 5.92–5.88 (m, 2H), 5.42 (d, *J* = 10.5 Hz, 1H), 4.01–3.94 (m, 1H), 3.50–3.41 (m, 1H); ^13^C NMR (100 MHz, CDCl_3_) δ 149.5, 147.0, 141.9, 140.7, 137.0, 136.9, 133.0, 131.0, 128.4, 128.3, 127.3, 126.6, 126.2, 124.7, 123.9, 123.1, 121.3, 116.4, 115.8, 114.6, 114.2, 108.2, 101.1, 98.7, 76.8, 50.7, 48.1; IR (KBr): 3801, 3735, 3670, 3421, 1844, 1576, 1670, 964, 861, 747 cm^−1^; (C_32_H_24_BrNO_3_-H)^−^ requires *m*/*z* 548.0867, found *m*/*z* 548.0844; ee: 65%, IA, hexane/isopropanol = 70/30, t_R_ =13.480 (minor), t_R_ = 28.633 (major).

**(*E*)-7-methyl-2-(7-phenyl-8-styryl-7,8-dihydro-6*H*-[1,3]** **dioxolo[4,5-*g*]chromen-6-yl)-1*H*-indole (3ea):**

Yield: 91% (44.1 mg); 85:15 dr; brown solid; m.p. 106.3–107.5 °C; [α]_D_^20^ = −20.0 (c 0.88, acetone); ^1^H NMR (400 MHz, CDCl_3_) δ 7.71–7.62 (m, 2H), 7.25–7.16 (m, 5H), 7.13–7.04 (m, 3H), 7.04–7.00 (m, 3H), 6.96 (d, *J* = 7.0 Hz, 1H), 6.82–6.75 (m, 2H), 6.57–6.54 (m, 1H), 6.16 (d, *J* = 16.6 Hz, 1H), 6.06–5.98 (m, 1H), 5.91 (s, 2H), 5.49 (d, *J* = 10.5 Hz, 1H), 4.00–3.94 (m, 1H), 3.60–3.50 (m, 1H), 2.35–2.29 (m, 3H); ^13^C NMR (100 MHz, CDCl_3_) δ 149.8, 146.9, 141.7, 141.1, 137.1, 135.8, 132.8, 131.3, 128.4, 128.3, 128.2, 127.2, 126.4, 126.2, 125.4, 123.1, 122.7, 120.3, 120.0, 117.8, 116.4, 114.8, 108.2, 101.0, 98.8, 76.8, 50.4, 48.4, 16.5; IR (KBr): 3853, 3751, 3735, 3711, 1734, 1684, 1476, 1152, 1038, 748 cm^−1^; (C_33_H_27_NO_3_-H)^−^ requires *m*/*z* 484.1918, found *m*/*z* 484.1893; ee: 59%, IA, hexane/isopropanol = 90/10, t_R_ =55.313 (minor), t_R_ = 117.610 (major).

**(*E*)-7-fluoro-2-(7-phenyl-8-styryl-7,8-dihydro-6*H*-[1,3]** **dioxolo[4,5-*g*]chromen-6-yl)-1*H*-indole (3fa):**

Yield: 98% (47.9 mg); 88:12 dr; brown solid; m.p. 78.2–79.0 °C; [α]_D_^20^ = −30.3 (c 0.96, acetone); ^1^H NMR (400 MHz, CDCl_3_) δ 8.03 (s, 1H), 7.58 (d, *J* = 8.0 Hz, 1H), 7.25–7.14 (m, 5H), 7.12–7.07 (m, 2H), 7.05–7.00 (m, 2H), 7.00–6.96 (m, 2H), 6.91–6.86 (m, 1H), 6.82 (d, *J* = 2.4 Hz, 1H), 6.78 (s, 1H), 6.57 (s, 1H), 6.20–6.16 (m, 1H), 6.06–5.97 (m, 1H), 5.92–5.89 (m, 2H), 5.45 (d, *J* = 10.5 Hz, 1H), 4.03–3.96 (m, 1H), 3.55–3.45 (m, 1H); ^13^C NMR (100 MHz, CDCl_3_) δ 148.4 (d, *J* = 261.0 Hz), 141.9, 140.7, 137.1, 133.0, 131.1, 128.4 (d, *J* = 18.0 Hz), 128.3, 127.3, 126.6, 126.2, 124.0, 120.2 (d, *J* = 6.0 Hz), 116.4, 116.0 (d, *J* = 3.0 Hz), 115.4, 108.3, 107.1 (d, *J* = 16.0 Hz), 101.1, 98.8, 76.8, 50.7, 48.2; IR (KBr): 3779, 3702, 3689, 3675, 3567, 1869, 1700, 1559, 1039, 748 cm^−1^; (C_32_H_24_FNO_3_-H)^−^ requires *m*/*z* 488.1667, found *m*/*z* 488.1672; ee: 60%, IA, hexane/isopropanol = 70/30, t_R_ = 11.473 (major), t_R_ = 14.520 (minor).

**(*E*)-2-(8-styryl-7-(o-tolyl)-7,8-dihydro-6*H*-[1,3]** **dioxolo[4,5-*g*]chromen-6-yl)-1*H*-indole (3ga):**

Yield: 60% (29.2 mg); 93:7 dr; brown solid; m.p. 181.2–182.9 °C; [α]_D_^20^ = −63.1 (c 0.58, acetone); ^1^H NMR (400 MHz, CDCl_3_) δ 7.85 (d, *J* = 7.6 Hz, 1H), 7.73 (s, 1H), 7.28 (s, 1H), 7.25–7.19 (m, 5H), 7.19–7.09 (m, 4H), 6.96–6.90 (m, 1H), 6.82–6.76 (m, 2H), 6.74–6.70 (m, 1H), 6.60 (s, 1H), 6.13–6.05 (m, 2H), 5.93–5.89 (m, 2H), 5.53 (d, *J* = 10.1 Hz, 1H), 4.00–3.91 (m, 1H), 3.90–3.83 (m, 1H), 1.91 (s, 3H); ^13^C NMR (100 MHz, CDCl_3_) δ 149.8, 147.0, 141.7, 139.9, 137.2, 136.2, 132.4, 130.7, 130.0, 128.5, 127.2, 126.2, 126.1, 125.9, 122.9, 122.1, 120.2, 119.7, 116.7, 114.2, 111.2, 108.2, 101.0, 98.8, 77.3, 49.5, 44.9, 20.0; IR (KBr): 3702, 3690, 3676, 1751, 1522, 1240, 1153, 743 cm^−1^; (C_33_H_27_NO_3_-H)^−^ requires *m*/*z* 484.1918, found *m*/*z* 484.1896; ee: 65%, IB, hexane/isopropanol = 90/10, t_R_ = 21.567 (minor), t_R_ = 23.540 (major).

**(*E*)-2-(7-(2-chlorophenyl)-8-styryl-7,8-dihydro-6*H*-[1,3]** **dioxolo[4,5-*g*]chromen-6-yl)-1*H*-indole (3ha):**

Yield: 62% (31.7 mg); 91:9 dr; brown solid; m.p. 195.1–195.6 °C; [α]_D_^20^ = −57.9 (c 0.63, acetone); ^1^H NMR (400 MHz, CDCl_3_) δ 7.92 (s, 1H), 7.79 (d, *J* = 7.0 Hz, 1H), 7.26–7.20 (m, 6H), 7.20–7.17 (m, 1H), 7.16–7.10 (m, 3H), 7.07 (m, 2H), 6.94–6.90 (m, 1H), 6.74 (s, 1H), 6.55 (s, 1H), 6.12–6.07 (m, 2H), 5.90 (s, 2H), 5.62 (d, *J* = 9.6 Hz, 1H), 4.38–4.27 (m, 1H), 3.91–3.80 (m, 1H); ^13^C NMR (100 MHz, CDCl_3_) δ 149.7, 147.1, 141.8, 139.4, 137.2, 136.1, 134.8, 132.6, 130.6, 129.3, 128.5, 127.8, 127.4, 127.3, 127.1, 126.3, 123.1, 122.3, 119.9, 119.6, 116.2, 114.2, 111.1, 108.1, 101.1, 98.8, 75.3, 50.3, 44.7; IR (KBr): 3853, 3676, 3650, 3629, 1685, 1507, 1477, 1037, 745 cm^−1^; (C_32_H_24_ClNO_3_-H)^−^ requires *m*/*z* 504.1372, found *m*/*z* 504.1377; ee: 65%, IA, hexane/isopropanol = 70/30, t_R_ = 10.020 (minor), t_R_ = 27.087 (major).

**(*E*)-2-(7-(2-bromophenyl)-8-styryl-7,8-dihydro-6*H*-[1,3]** **dioxolo[4,5-*g*]chromen-6-yl)-1*H*-indole (3ia):**

Yield: 96% (52.8 mg); 92:8 dr; brown solid; m.p. 185.0–185.4 °C; [α]_D_^20^ = −59.3 (c 1.06, acetone); ^1^H NMR (400 MHz, CDCl_3_) δ 7.90 (s, 1H), 7.81 (d, *J* = 7.3 Hz, 1H), 7.30–7.27 (m, 4H), 7.25–7.19 (m, 4H), 7.18–7.10 (m, 3H), 7.08 (d, *J* = 2.4 Hz, 1H), 6.88–6.82 (m, 1H), 6.76 (s, 1H), 6.56 (s, 1H), 6.18–6.13 (m, 1H), 6.12–6.05 (m, 1H), 5.90 (s, 2H), 5.63 (d, *J* = 10.3 Hz, 1H), 4.37–4.27 (m, 1H), 3.91–3.82 (m, 1H); ^13^C NMR (100 MHz, CDCl_3_) δ 149.7, 147.1, 141.9, 141.1, 137.2, 136.0, 132.7, 132.6, 130.5, 128.5, 128.1, 127.8, 127.3, 126.4, 126.1, 123.3, 122.3, 119.9, 119.7, 116.2, 114.0, 111.2, 108.1, 101.1, 98.8, 75.5, 50.7, 47.6; IR (KBr): 3418, 3055, 2885, 1499, 1477, 1240, 1152, 1037, 965, 744 cm^−1^; (C_32_H_24_BrNO_3_-H)^−^ requires *m*/*z* 548.0867 found *m*/*z* 548.0849; ee: 66%, IA, hexane/isopropanol = 70/30, t_R_ = 10.603 (minor), t_R_ = 29.107 (major).

**(*E*)-2-(8-styryl-7-(m-tolyl)-7,8-dihydro-6*H*-[1,3]** **dioxolo[4,5-*g*]chromen-6-yl)-1*H*-indole (3ja):**

Yield: 84% (40.8 mg); 84:16 dr; brown solid; m.p. 148.3–148.6 °C; [α]_D_^20^ = −29.2 (c 0.82, acetone); ^1^H NMR (400 MHz, CDCl_3_) δ 7.82 (d, *J* = 7.4 Hz, 1H), 7.78 (s, 1H), 7.26–7.18 (m, 6H), 7.17–7.11 (m, 2H), 6.98–6.94 (m, 1H), 6.85–6.77 (m, 5H), 6.57 (s, 1H), 6.20–6.16 (m, 1H), 6.06–5.98 (m, 1H), 5.91 (s, 2H), 5.48 (d, *J* = 10.5 Hz, 1H), 4.00–3.93 (m, 1H), 3.53–3.44 (m, 1H), 2.15 (s, 3H); ^13^C NMR (100 MHz, CDCl_3_) δ 149.8, 146.9, 141.7, 140.9, 137.5, 137.2, 136.2, 132.7, 131.5, 129.0, 128.4, 128.0, 127.2, 126.2, 126.0, 125.4, 123.3, 122.1, 120.0, 119.7, 116.5, 114.4, 111.2, 108.2, 101.0, 98.8, 76.8, 50.4, 48.3, 21.4; IR (KBr): 3801, 3734, 3648, 3587, 1749, 1576, 1521, 1038, 746 cm^−1^; (C_33_H_27_NO_3_-H)^−^ requires *m*/*z* 484.1918, found *m*/*z* 484.1905; ee: 59%, IA, hexane/isopropanol = 70/30, t_R_ = 10.097 (minor), t_R_ = 19.040 (major).

**(*E*)-2-(7-(3-chlorophenyl)-8-styryl-7,8-dihydro-6*H***-**[1,3]****dioxolo[4,5-*g*]chromen-6-yl)-1*H*-indole (3ka):**

Yield: 98% (49.5 mg); 87:13 dr; brown solid; m.p. 160.3–160.9 °C; [α]_D_^20^ = −30.5 (c 0.99, acetone); ^1^H NMR (400 MHz, CDCl_3_) δ 7.87 (s, 1H), 7.81 (d, *J* = 7.6 Hz, 1H), 7.30–7.27 (m, 2H), 7.26–7.13 (m, 6H), 7.04 (s, 1H), 7.00–6.93 (m, 2H), 6.84–6.80 (m, 2H), 6.77 (s, 1H), 6.56 (s, 1H), 6.22 (d, *J* = 15.8 Hz, 1H), 6.00 (m, 1H), 5.91 (s, 2H), 5.42 (d, *J* = 10.5 Hz, 1H), 3.98–3.92 (m, 1H), 3.57–3.49 (m, 1H); ^13^C NMR (100 MHz, CDCl_3_) δ 149.7, 147.1, 143.1, 141.9, 136.9, 136.3, 133.9, 133.2, 130.8, 129.4, 128.5, 128.0, 127.4, 126.9, 126.7, 126.3, 125.7, 123.4, 122.3, 119.9, 116.0, 114.0, 111.3, 108.1, 101.1, 98.8, 76.7, 50.4, 48.2; IR (KBr): 3749, 3647, 3617, 3419, 1698, 1683, 1418, 745, 668 cm^−1^; (C_32_H_24_ClNO_3_-H)^−^ requires *m*/*z* 504.1372, found *m*/*z* 504.1389; ee: 58%, IB, hexane/isopropanol = 90/10, t_R_ = 24.790 (minor), t_R_ = 26.910 (major).

**(*E*)-2-(8-styryl-7-(p-tolyl)-7,8-dihydro-6*H*-[1,3]** **dioxolo[4,5-*g*]chromen-6-yl)-1*H*-indole (3la):**

Yield: 54% (26.0 mg); 78:22 dr; brown solid; m.p. 220.1–221.9°C; [α]_D_^20^ = −46.2 (c 0.52, acetone); ^1^H NMR (400 MHz, CDCl_3_) δ 7.86–7.79 (m, 2H), 7.28 (s, 1H), 7.25–7.22 (m, 4H), 7.17–7.12 (m, 2H), 7.05–6.97 (m, 1H), 6.87 (s, 4H), 6.79 (d, *J* = 2.4 Hz, 1H), 6.76 (s, 1H), 6.56 (s, 1H), 6.20–6.16 (m, 1H), 6.05–5.97 (m, 1H), 5.90 (s, 2H), 5.45 (d, *J* = 10.5 Hz, 1H), 3.97–3.92 (m, 1H), 3.54–3.47 (m, 1H), 2.16 (s, 3H); ^13^C NMR (100 MHz, CDCl_3_) δ 149.8, 147.0, 141.7, 137.9, 137.2, 136.3, 135.8, 132.7, 131.5, 128.9, 128.5, 128.1, 127.2, 126.3, 126.0, 123.4, 122.2, 120.2, 119.8, 116.5, 114.5, 111.2, 108.3, 101.0, 98.8, 77.0, 50.0, 48.3, 21.0; IR (KBr): 3726, 3675, 3649, 3587, 1869, 1670, 1395, 1152, 744 cm^−1^; (C_33_H_27_NO_3_-H)^−^ requires *m*/*z* 484.1918, found *m*/*z* 484.1902; ee: 85%, IA, hexane/isopropanol = 70/30, t_R_ = 10.557 (minor), t_R_ = 18.093 (major).

**(*E*)-2-(7-(4-chlorophenyl)-8-styryl-7,8-dihydro-6*H*-[1,3]** **dioxolo[4,5-*g*]chromen-6-yl)-1*H*-indole (3ma):**

Yield: 58% (29.5 mg); 84:16 dr; brown solid; m.p. 85.2–86.0 °C; [α]_D_^20^ = −16.7 (c 0.59, acetone); ^1^H NMR (400 MHz, CDCl_3_) δ 7.88 (s, 1H), 7.81 (d, *J* = 7.8 Hz, 1H), 7.30–7.26 (m, 2H), 7.26–7.25 (m, 2H), 7.23–7.11 (m, 4H), 7.06–7.00 (m, 2H), 6.89 (d, *J* = 8.4 Hz, 2H), 6.79 (d, *J* = 2.5 Hz, 1H), 6.76 (s, 1H), 6.55 (s, 1H), 6.22–6.17 (m, 1H), 6.04–5.95 (m, 1H), 5.91 (s, 2H), 5.40 (d, *J* = 10.5 Hz, 1H), 3.96–3.90 (m, 1H), 3.58–3.49 (m, 1H); ^13^C NMR (100 MHz, CDCl_3_) δ 149.7, 147.1, 141.9, 139.5, 136.9, 136.4, 133.1, 132.1, 130.9, 129.6, 128.5, 128.4, 127.4, 126.3, 125.7, 123.4, 122.4, 120.1, 120.0, 116.1, 114.1, 111.4, 108.2, 101.1, 98.8, 76.9, 50.0, 48.2; IR (KBr): 3870, 3711, 3690, 3629, 1844, 1684, 1576, 1395, 745 cm^−1^; (C_32_H_24_ClNO_3_-H)^−^ requires *m*/*z* 504.1372, found *m*/*z* 504.1374; *ee*: 97%, IA, hexane/isopropanol = 70/30, t_R_ = 11.823 (minor), t_R_ = 20.797 (major).

**(*E*)-5-chloro-1-methyl-2-(7-phenyl-8-styryl-7,8-dihydro-6*H*-[1,3]** **dioxolo[4,5-*g*]chromen-6-yl)-1*H*-indole (3na):**

Yield:47% (24.2 mg); 86:14 dr; brown solid; m.p. 205.1–205.7 °C; [α]_D_^20^ = −11.4 (c 0.48, acetone); ^1^H NMR (400 MHz, acetone-*d_6_*) δ 7.77 (m, 1H), 7.30–7.23 (m, 6H), 7.20–7.15 (m, 3H), 7.11–7.05 (m, 4H), 6.99–6.94 (m, 1H), 6.71 (s, 1H), 6.43 (s, 1H), 6.24–6.20 (m, 1H), 6.17–6.11 (m, 1H), 5.92 (d, *J* = 1.8 Hz, 2H), 5.57 (d, *J* = 10.6 Hz, 1H), 3.65 (s, 3H), 3.62–3.56 (m, 1H); ^13^C NMR (100 MHz, acetone-*d_6_*) δ 149.9, 147.0, 141.7, 141.4, 137.3, 135.5, 132.6, 131.6, 130.3, 129.3, 128.6, 128.4, 128.0, 127.1, 126.2, 126.1, 124.4, 121.3, 119.1, 116.6, 113.1, 110.9, 107.9, 101.0, 98.2, 76.0, 50.4, 48.7, 32.1; IR (KBr): 3868 3852, 3688, 3627, 1791, 1733, 1698, 1521, 1507, 746 cm^−1^; (C_33_H_26_ClNO_3_-H)^−^ requires *m*/*z* 518.1528, found *m*/*z* 518.1526; ee: 14%, IB, hexane/isopropanol = 80/20, t_R_ = 9.320 (major), t_R_ = 9.850 (minor).

**(*E*)-2-(8-(2-methylstyryl)-7-phenyl-7,8-dihydro-6*H*-[1,3]** **dioxolo[4,5-*g*]chromen-6-yl)-1*H*-indole (3ab):**

Yield: 98% (47.5 mg); 88:12 dr; brown solid; m.p. 203.3–205.0 °C; [α]_D_^20^ = −6.0 (c 0.95, acetone); ^1^H NMR (400 MHz, CDCl_3_) δ 7.84 (d, *J* = 7.4 Hz, 1H), 7.77 (s, 1H), 7.34–7.30 (m, 1H), 7.21–7.15 (m, 3H), 7.14–7.09 (m, 4H), 7.09–7.04 (m, 2H), 7.03–7.00 (m, 2H), 6.82 (s, 1H), 6.78 (m, 1H), 6.59 (s, 1H), 6.33 (d, *J* = 15.5 Hz, 1H), 5.94–5.92 (m, 2H), 5.88–5.80 (m, 1H), 5.52 (d, *J* = 10.5 Hz, 1H), 4.05–3.98 (m, 1H), 3.60–3.49 (m, 1H), 2.01 (s, 3H); ^13^C NMR (100 MHz, CDCl_3_) δ 149.7, 147.0, 141.7, 141.0, 136.6, 136.2, 135.2, 132.7, 131.5, 130.0, 128.4, 128.2, 127.2, 126.4, 126.1, 126.0, 123.4, 122.2, 120.1, 119.8, 116.5, 114.4, 111.2, 108.1, 101.0, 98.8, 76.8, 50.4, 48.7, 19.5; IR (KBr): 3763, 3668, 3627, 2899, 1732, 1682, 1568, 1479, 1153, 744 cm^−1^; (C_33_H_27_NO_3_-H)^−^ requires *m*/*z* 484.1918, found *m*/*z* 484.1894; ee: 62%, AD-H, hexane/isopropanol = 70/30, t_R_ = 8.893 (minor), t_R_ = 13.527 (major).

**(*E*)-2-(8-(3-methoxystyryl)-7-phenyl-7,8-dihydro-6*H*-[1,3]** **dioxolo[4,5-*g*]chromen-6-yl)-1*H*-indole (3ac):**

Yield: 98% (49.1 mg); 86:14 dr; brown solid; m.p. 120.9–121.2 °C; [α]_D_^20^ = −39.9 (c 0.98, acetone); ^1^H NMR (400 MHz, CDCl_3_) δ 7.84 (d, *J* = 7.3 Hz, 1H), 7.78 (s, 1H), 7.21–7.16 (m, 3H), 7.16–7.15 (m, 1H), 7.11–7.05 (m, 2H), 7.05–6.97 (m, 3H), 6.84 (d, *J* = 7.8 Hz, 1H), 6.78 (s, 2H), 6.77–6.74 (m, 2H), 6.58 (s, 1H), 6.17–6.12 (m, 1H), 6.07–5.99 (m, 1H), 5.92–5.90 (m, 2H), 5.48 (d, *J* = 10.5 Hz, 1H), 4.01–3.95 (m, 1H), 3.78 (s, 3H), 3.59–3.49 (m, 1H); ^13^C NMR (100 MHz, CDCl_3_) δ 159.7, 149.8, 147.0, 141.8, 140.9, 138.6, 136.2, 132.7, 131.6, 129.4, 128.3, 128.2, 126.4, 125.9, 123.4, 122.2, 120.0, 119.8, 118.9, 116.3, 114.3, 112.9, 111.5, 111.2, 108.2, 101.0, 98.8, 76.9, 55.2, 50.5, 48.2; IR (KBr): 3904, 3690, 3675, 3421, 1734, 1670, 1522, 1153, 746 cm^−1^; (C_33_H_27_NO_4_-H)^−^ requires *m*/*z* 500.1867, found *m*/*z* 500.1868; ee: 63%, IA, hexane/isopropanol = 70/30, t_R_ = 11.337 (minor), t_R_ = 26.440 (major).

**(*E*)-2-(8-(3-fluorostyryl)-7-phenyl-7,8-dihydro-6*H*-[1,3]** **dioxolo[4,5-*g*]chromen-6-yl)-1*H*-indole (3ad):**

Yield: 98% (47.9 mg); 85:15 dr; brown solid m.p. 163.6–164.0 °C; [α]_D_^20^ = −39.6 (c 0.96, acetone); ^1^H NMR (400 MHz, CDCl_3_) δ 7.83 (d, *J* = 7.3 Hz, 1H), 7.78 (s, 1H), 7.23–7.13 (m, 5H), 7.08 (d, *J* = 7.5 Hz, 1H), 7.06–7.01 (m, 1H), 7.01–6.96 (m, 3H), 6.94–6.85 (m, 2H), 6.77 (m, 1H), 6.74 (s, 1H), 6.58 (s, 1H), 6.16–6.10 (m, 1H), 6.08–5.99 (m, 1H), 5.92 (s, 2H), 5.48 (d, *J* = 10.5 Hz, 1H), 4.01–3.95 (m, 1H), 3.58–3.49 (m, 1H); ^13^C NMR (100 MHz, CDCl_3_) δ 163.0 (d, *J* = 244.0 Hz), 149.8, 147.1, 141.8, 140.8, 139.4 (d, *J* = 7.0 Hz), 136.2, 132.7, 131.8, 131.7, 129.9 (d, *J* = 9.0 Hz), 128.3, 128.2, 126.5, 125.9, 123.4, 122.2, 122.1, 119.9 (*J* = 21.0 Hz), 116.0, 114.2, 114.0 (d, *J* = 21.0 Hz), 112.6 (d, *J* = 22.0 Hz), 111.2, 108.1, 101.0, 98.9, 76.8, 50.4, 48.2; IR (KBr): 3869, 3750, 3734, 3587, 1868, 1683, 1521, 1038, 748, 668 cm^−1^; (C_32_H_24_FNO_3_-H)^−^ requires *m*/*z* 488.1667, found *m*/*z* 488.1675; ee: 60%, AD-H, hexane/isopropanol = 70/30, t_R_ = 11.737 (minor), t_R_ = 49.947 (major).

**(*E*)-2-(8-(4-methoxystyryl)-7-phenyl-7,8-dihydro-6*H*-[1,3]** **dioxolo[4,5-*g*]chromen-6-yl)-1*H*-indole (3ae):**

Yield: 73% (36.5 mg); 89:11 dr; brown solid m.p. 207.6–207.7 °C; [α]_D_^20^ = −47.8 (c 0.73, acetone); ^1^H NMR (400 MHz, acetone-*d*_6_) δ 9.98 (s, 1H), 7.81 (d, *J* = 7.7 Hz, 1H), 7.29 (d, *J* = 8.0 Hz, 1H), 7.23–7.20 (m, 2H), 7.17–7.13 (m, 2H), 7.10 (m, 1H), 7.06–7.00 (m, 4H), 6.96–6.91 (m, 1H), 6.82–6.78 (m, 2H), 6.72 (s, 1H), 6.43 (s, 1H), 6.18–6.12 (m, 1H), 6.01–5.94 (m, 1H), 5.91 (m, 2H), 5.59 (d, *J* = 10.6 Hz, 1H), 4.01–3.94 (m, 1H), 3.74 (s, 3H), 3.67–3.60 (m, 1H); ^13^C NMR (100 MHz, acetone-*d_6_*) δ 159.2, 150.1, 147.0, 141.8, 141.5, 136.7, 132.0, 130.0, 129.4, 128.6, 127.8, 127.3, 126.5, 126.0, 124.4, 121.3, 119.7, 118.9, 116.9, 114.1, 113.8, 111.4, 108.0, 101.0, 98.2, 76.6, 54.6, 50.4, 48.7; IR (KBr): 3742, 3720, 3708, 3306, 1574, 1434, 1247, 745, 668 cm^−1^; (C_33_H_27_NO_4_-H)^−^ requires *m*/*z* 500.1867, found *m*/*z* 500.1871; ee: 77%, AD-H, hexane/isopropanol = 80/20, t_R_ = 22.810 (minor), t_R_ = 55.720 (major).

**(*E*)-2-(8-(4-fluorostyryl)-7-phenyl-7,8-dihydro-6*H*-[1,3]** **dioxolo[4,5-*g*]chromen-6-yl)-1*H*-indole (3af):**

Yield: 53% (25.9 mg); 75:25 dr; brown solid; m.p. 81.4–82.6 °C; [α]_D_^20^ = −53.3 (c 0.52, acetone); ^1^H NMR (400 MHz, CDCl_3_) δ 7.85–7.79 (m, 2H), 7.23 (d, *J* = 7.7 Hz, 1H), 7.19–7.12 (m, 6H), 7.07 (d, *J* = 7.5 Hz, 1H), 7.04–7.01 (m, 1H), 6.99–6.96 (m, 2H), 6.95–6.91 (m, 2H), 6.78 (m, 1H), 6.75 (s, 1H), 6.56 (s, 1H), 6.13–6.09 (m, 1H), 5.91 (s, 2H), 5.47 (d, *J* = 10.5 Hz, 1H), 3.98–3.93 (m, 1H), 3.56–3.49 (m, 1H); ^13^C NMR (100 MHz, CDCl_3_) δ 162.1 (d, *J* = 245.0 Hz), 149.8, 147.0, 141.7, 140.9, 136.2, 133.3, 133.2, 131.6, 131.1 (d, *J* = 2.0 Hz) 129.1, 128.2 (d, *J* = 13.0 Hz), 127.7 (d, *J* = 8.0 Hz), 126.4, 125.9, 123.3, 122.2, 120.0, 119.8, 116.3, 115.3 (d, *J* = 21.0 Hz), 114.3, 111.2, 108.1, 101.0, 98.8, 76.9, 50.5, 48.2; IR (KBr): 3891, 3884, 3734, 3669, 3446, 2970, 1669, 1066, 749, 668 cm^−1^; (C_32_H_24_FNO_3_-H)^−^ requires *m*/*z* 488.1667, found *m*/*z* 488.1668; ee: 97%, AD-H, hexane/isopropanol = 70/30, t_R_ = 15.207 (minor), t_R_ = 22.800 (major).

**(*E*)-2-(8-(4-chlorostyryl)-7-phenyl-7,8-dihydro-6*H*-[1,3]** **dioxolo[4,5-*g*]chromen-6-yl)-1*H*-indole (3ag):**

Yield: 57% (28.7 mg); 84:16 dr; brown solid; m.p. 175.7–176.2 °C; [α]_D_^20^ = −89.5 (c 0.51, acetone); ^1^H NMR (400 MHz, CDCl_3_) δ 7.85–7.78 (m, 2H), 7.25–7.21 (m, 2H), 7.19 (s, 1H), 7.17–7.15 (m, 1H), 7.14–7.13 (m, 2H), 7.12–7.11 (m, 1H), 7.10–7.05 (m, 2H), 7.04–7.00 (m, 1H), 6.99–6.95 (m, 2H), 6.78 (d, *J* = 2.5 Hz, 1H), 6.73 (s, 1H), 6.57 (s, 1H), 6.12–6.06 (m, 1H), 6.02–5.94 (m, 1H), 5.92–5.89 (m, 2H), 5.47 (d, *J* = 10.5 Hz, 1H), 3.99–3.93 (m, 1H), 3.57–3.47 (m, 1H); ^13^C NMR (100 MHz, CDCl_3_) 149.8, 147.0, 141.8, 140.9, 136.2, 135.5, 132.8, 132.0, 131.6, 128.5, 128.3, 128.2, 127.4, 126.5, 125.9, 123.3, 122.2, 120.0, 119.8, 116.1, 114.3, 111.2, 108.1, 101.0, 98.8, 76.8, 50.5, 48.2; IR (KBr): 3869, 3688, 3627, 1791, 1683, 1521, 1076, 749, 668 cm^−1^; (C_32_H_24_ClNO_3_-H)^−^ requires *m*/*z* 504.1372, found *m*/*z* 504.1379; ee: 98%, IB, hexane/isopropanol = 70/30, t_R_ = 7.900 (major), t_R_ = 8.993 (minor).

**(*E*)-2-(7-phenyl-8-(2-(thiophen-2-yl)vinyl)-7,8-dihydro-6*H*-[1,3]** **dioxolo[4,5-*g*]chromen-6-yl)-1*H*-indole (3ah):**

Yield: 70% (33.2 mg); 85:15 dr; brown solid; m.p. 216.2–218.0 °C; [α]_D_^20^ = −53.9 (c 0.66, acetone); ^1^H NMR (400 MHz, CDCl_3_) δ 7.84–7.79 (m, 2H), 7.25–7.21 (m, 1H), 7.15–7.11 (m, 2H), 7.10–7.06 (m, 3H), 7.04–7.01 (m, 1H), 7.00–6.97 (m, 2H), 6.91–6.87 (m, 1H), 6.79–6.75 (m, 3H), 6.55 (s, 1H), 6.28 (d, *J* = 15.6 Hz, 1H), 5.92–5.90 (m, 2H), 5.89–5.81 (m, 1H), 5.46 (d, *J* = 10.5 Hz, 1H), 3.95–3.89 (m, 1H), 3.55–3.47 (m, 1H); ^13^C NMR (100 MHz, CDCl_3_) δ 149.8, 147.1, 142.2, 141.8, 141.0, 136.3, 131.1, 128.3, 128.2, 127.2, 126.5, 126.0, 125.9, 125.1, 123.7, 123.4, 122.2, 120.1, 119.8, 116.2, 114.3, 111.2, 108.2, 101.1, 98.9, 76.9, 50.5, 48.2; IR (KBr): 3868, 3851, 3742, 3674, 3565, 1715, 1506, 1265, 1153, 743 cm^−1^; (C_30_H_23_NO_3_S-H)^−^ requires *m*/*z* 476.1326, found *m*/*z* 476.1315; ee: 87%, IB, hexane/isopropanol = 90/10, t_R_ = 22.563 (minor), t_R_ = 24.167 (major).

**(*E*)-3-(7-(4-chlorophenyl)-8-styryl-7,8-dihydro-6*H*-[1,3]** **dioxolo[4,5-*g*]chromen-6-yl)-1-methyl-1*H*-indole:(3oa)**

Yield: 53% (27.7 mg); 91:9 dr; white solid; m.p. 183.0–183.7 °C; [α]_D_^20^ = −46.6 (c 0.15, acetone); ^1^H NMR (400 MHz, CDCl_3_) δ 7.75 (d, *J* = 7.9 Hz, 1H), 7.25–7.17 (m, 7H), 7.13–7.08 (m, 1H), 7.03 (d, *J* = 8.2 Hz, 2H), 6.91 (d, *J* = 8.3 Hz, 2H), 6.73 (d, *J* = 4.3 Hz, 2H), 6.52 (s, 1H), 6.15 (d, *J* = 15.7 Hz, 1H), 6.01–5.94 (m, 1H), 5.90 (s, 2H), 5.40 (d, *J* = 10.5 Hz, 1H), 3.92–3.86 (m, 1H), 3.62 (s, 3H), 3.56–3.49 (m, 1H); ^13^C NMR (100 MHz, CDCl_3_) δ 149.7, 147.0, 141.8, 139.6, 137.2, 136.9, 133.0, 132.0, 130.9, 129.5, 128.5, 128.3, 128.0, 127.4, 126.3, 126.2, 121.8, 119.9, 119.4, 116.0, 112.4, 109.4, 108.1, 101.0, 98.8, 76.5, 50.0, 48.5, 32.7; IR (KBr): 3852, 3749, 3648, 2920, 1477, 1238, 1152, 1037, 1013, 742 cm^−1^; (C_33_H_26_ClNO_3_+H)^+^ requires *m*/*z* 520.1674, found *m*/*z* 520.1668; ee: 83%, AD-H, hexane/isopropanol = 70/30, t_R_ = 9.900 (minor), t_R_ = 10.893 (major).

**Procedure for one-mmol-scale synthesis of product 3aa**

To the mixture of 3-vinylindole **1a** (1.0 mmol), *ortho*-quinone methide **2a** (1.2 mmol), catalyst **(*R*)-4c** (0.1 mmol), and MgSO_4_ (500 mg) was added toluene (10 mL). Then, the reaction mixture was stirred at 0 °C for 12 h. After completion of the reaction, which was indicated by TLC, the reaction mixture was directly purified through flash column chromatography to afford product **3aa** in 88% yield (414 mg) with 86:14 dr and 64% ee.

**General procedure for the synthesis of products 6**

To the mixture of 3-vinylindoles **1** (0.1 mmol), *ortho*-hydroxybenzyl alcohols **5** (0.12 mmol), catalyst **4e** (0.01 mmol) were added toluene (1 mL). Then, the reaction mixture was stirred at 0 °C for 6 h. After completion of the reaction, which was indicated by TLC, the reaction mixture was directly purified through flash column chromatography to afford products **6**.

**(*E*)-3-(3,4-diphenylchroman-2-yl)-1*H*-indole (6aa):**

Yield: 61% (24.5 mg); 77:23 dr; white solid; m.p. 95.0–97.0 °C; [α]_D_^20^ = −169.4 (c 0.26, acetone); ^1^H NMR (400 MHz, CDCl_3_) δ 7.84 (s, 1H), 7.79 (d, *J* = 7.7 Hz, 1H), 7.25–7.20 (m, 2H), 7.17–7.08 (m, 5H), 7.07–7.01 (m, 2H), 7.00–6.97 (m, 1H), 6.97–6.92 (m, 2H), 6.91–6.86 (m, 2H), 6.72 (d, *J* = 7.5 Hz, 2H), 6.59 (d, *J* = 7.5 Hz, 2H), 5.93 (d, *J* = 10.7 Hz, 1H), 4.40 (d, *J* = 5.2 Hz, 1H), 4.20–4.11 (m, 1H); ^13^C NMR (100 MHz, CDCl_3_) δ 154.9, 141.5, 139.6, 136.2, 130.6, 130.2, 129.1, 128.1, 127.5, 126.4, 126.2, 126.1, 124.8, 123.7, 122.2, 120.4, 119.9, 119.8, 116.8, 114.7, 111.2, 71.2, 48.7, 48.1; IR (KBr): 3648, 3566, 1733, 1456, 1418, 1242, 1111, 990, 748, 700 cm^−1^; (C_29_H_23_NO+H)^+^ requires *m*/*z* 402.1853, found *m*/*z* 402.1853; ee: 79%, OD-H, hexane/isopropanol = 95/5, t_R_ = 20.910 (major), t_R_ = 23.560 (minor).

**(*E*)-3-(3,4-diphenylchroman-2-yl)-5-methyl-1*H*-indole (6ba**):

Yield: 50% (20.7 mg); 75:25 dr; white solid; m.p. 84.0–86.0 °C; [α]_D_^20^ = −145.2 (c 0.17, acetone); ^1^H NMR (400 MHz, CDCl_3_) δ 7.76 (s, 1H), 7.56 (s, 1H), 7.25–7.20 (m, 1H), 7.18–7.09 (m, 4H), 7.08–7.04 (m, 1H), 7.04–7.01 (m, 1H), 7.00–6.91 (m, 4H), 6.91–6.86 (m, 1H), 6.84 (d, *J* = 2.2 Hz, 1H), 6.72 (d, *J* = 7.3 Hz, 2H), 6.60 (d, *J* = 7.4 Hz, 2H), 5.90 (d, *J* = 10.6 Hz, 1H), 4.40 (d, *J* = 5.3 Hz, 1H), 4.20–4.11 (m, 1H), 2.44 (s, 3H); ^13^C NMR (100 MHz, CDCl_3_) δ 154.9, 141.6, 139.7, 134.6, 130.6, 130.3, 129.1, 129.0, 128.1, 127.5, 127.4, 126.4, 126.2, 124.8, 123.8, 120.3, 119.4, 116.9, 114.1, 110.8, 71.2, 48.7, 47.9, 21.6; IR (KBr): 3648, 3566, 2922, 1748, 1558, 1507, 1456, 1242, 750, 668 cm^−1^; (C_30_H_25_NO+H)^+^ requires *m*/*z* 416.2009, found *m*/*z* 416.1992; ee: 76%, AD-H, hexane/isopropanol = 70/30, t_R_ = 5.200 (minor), t_R_ = 7.937 (major).

**(*E*)-6-chloro-3-(3,4-diphenylchroman-2-yl)-1*H*-indole (6pa):**

Yield: 72% (31.3 mg); 75:25 dr; white solid; m.p. 112.0–114.0 °C; [α]_D_^20^ = −253.5 (c 0.20, acetone); ^1^H NMR (400 MHz, CDCl_3_) δ 7.77 (s, 1H), 7.69 (d, *J* = 8.5 Hz, 1H), 7.22 (d, *J* = 7.7 Hz, 1H), 7.20–7.10 (m, 4H), 7.09–7.01 (m, 3H), 7.01–6.97 (m, 1H), 6.97–6.87 (m, 3H), 6.86–6.83 (m, 1H), 6.72 (d, *J* = 7.4 Hz, 2H), 6.55 (d, *J* = 7.4 Hz, 2H), 5.87 (d, *J* = 10.9 Hz, 1H), 4.39 (d, *J* = 5.2 Hz, 1H), 4.14–4.06 (m, 1H); ^13^C NMR (100 MHz, CDCl_3_) δ 154.7, 141.4, 139.3, 136.5, 130.6, 130.3, 129.1, 128.2, 128.1, 127.5, 126.5, 126.4, 124.8, 124.6, 124.3, 120.7, 120.6, 116.8, 114.9, 111.1, 71.0, 48.7, 48.3; IR (KBr): 3750, 3724, 3648, 3026, 1488, 1456, 1239, 1216, 752, 705 cm^−1^; (C_29_H_22_ClNO+H)^+^ requires *m*/*z* 436.1463, found *m*/*z* 436.1460; ee: 74%, AD-H, hexane/isopropanol = 70/30, t_R_ = 5.843 (minor), t_R_ = 7.477 (major).

**(*E*)-6-bromo-3-(3,4-diphenylchroman-2-yl)-1*H*-indole (6da):**

Yield: 77% (36.8 mg); 78:22 dr; white solid; m.p. 95.0–97.0 °C; [α]_D_^20^ = −203.3 (c 0.28, acetone); ^1^H NMR (400 MHz, CDCl_3_) δ 7.77 (s, 1H), 7.64 (d, *J* = 8.5 Hz, 1H), 7.33 (s, 1H), 7.24–7.15 (m, 3H), 7.15–7.10 (m, 2H), 7.05–7.01 (m, 2H), 6.99 (d, *J* = 7.3 Hz, 1H), 6.97–6.92 (m, 2H), 6.92–6.87 (m, 1H), 6.82 (d, *J* = 2.1 Hz, 1H), 6.72 (d, *J* = 7.4 Hz, 2H), 6.55 (d, *J* = 7.4 Hz, 2H), 5.87 (d, *J* = 10.9 Hz, 1H), 4.39 (d, *J* = 5.2 Hz, 1H), 4.13–4.06 (m, 1H); ^13^C NMR (100 MHz, CDCl_3_) δ 154.7, 141.4, 139.3, 137.0, 130.6, 130.3, 129.1, 128.2, 127.5, 126.5, 126.4, 124.9, 124.8, 124.3, 123.1, 121.1, 120.6, 116.7, 115.7, 114.9, 114.1, 71.0, 48.7, 48.3; IR (KBr): 3648, 3566, 1716, 1540, 1507, 1456, 1226, 801, 750, 668 cm^−1^; (C_29_H_22_BrNO+H)^+^ requires *m*/*z* 480.0958, found *m*/*z* 480.0947; ee: 74%, AD-H, hexane/isopropanol = 70/30, t_R_ = 6.190 (minor), t_R_ = 8.233 (major).

**(*E*)-3-(3,4-diphenylchroman-2-yl)-7-methyl-1*H*-indole (6ea):**

Yield: 53% (21.9 mg); 75:25 dr; white solid; m.p. 92.0–94.0 °C; [α]_D_^20^ = −158.6 (c 0.19, acetone); ^1^H NMR (400 MHz, CDCl_3_) δ 7.72 (s, 1H), 7.63 (d, *J* = 7.9 Hz, 1H), 7.25–7.19 (m, 1H), 7.18–7.09 (m, 3H), 7.06–6.98 (m, 4H), 6.97–6.92 (m, 3H), 6.91–6.85 (m, 2H), 6.72 (d, *J* = 7.4 Hz, 2H), 6.61 (d, *J* = 7.4 Hz, 2H), 5.92 (d, *J* = 10.6 Hz, 1H), 4.39 (d, *J* = 5.2 Hz, 1H), 4.21–4.11 (m, 1H), 2.33 (s, 3H); ^13^C NMR (100 MHz, CDCl_3_) δ 154.9, 141.5, 139.7, 135.8, 130.6, 130.2, 129.1, 128.1, 127.5, 127.4, 126.4, 126.2, 125.6, 124.8, 123.4, 122.7, 120.3, 120.0, 117.5, 116.8, 115.2, 71.2, 48.7, 48.0, 16.5; IR (KBr): 3628, 3587, 3566, 3030, 1868, 1716, 1569, 1558, 749, 668 cm^−1^; (C_30_H_25_NO+H)^+^ requires *m*/*z* 416.2009, found *m*/*z* 416.2004; ee: 74%, AD-H, hexane/isopropanol = 70/30, t_R_ = 5.437 (major), t_R_ = 6.597 (minor).

**(*E*)-7-chloro-3-(3,4-diphenylchroman-2-yl)-1*H*-indole (6qa):**

Yield: 90% (39.2 mg); 82:18 dr; white solid; m.p. 55.0–57.0 °C; [α]_D_^20^ = −191.1 (c 0.54, acetone); ^1^H NMR (400 MHz, CDCl_3_) δ 8.07 (s, 1H), 7.70 (d, *J* = 7.9 Hz, 1H), 7.25–7.21 (m, 1H), 7.20–7.11 (m, 4H), 7.08–6.99 (m, 4H), 6.99 –6.88 (m, 4H), 6.73 (d, *J* = 7.2 Hz, 2H), 6.58 (d, *J* = 7.3 Hz, 2H), 5.91 (d, *J* = 10.9 Hz, 1H), 4.40 (d, *J* = 5.1 Hz, 1H), 4.20–4.07 (m, 1H); ^13^C NMR (100 MHz, CDCl_3_) δ 154.7, 141.4, 139.3, 133.5, 130.6, 130.3, 129.1, 128.2, 127.6, 127.5, 126.5, 126.4, 124.8, 124.3, 121.6, 120.7, 120.6, 118.6, 116.8, 116.7, 115.9, 71.1, 48.8, 48.2; IR (KBr): 3648, 3628, 3566, 3420, 1507, 1472, 1339, 1241, 752, 700 cm^−1^; (C_29_H_22_ClNO+H)^+^ requires *m*/*z* 436.1463, found *m*/*z* 436.1459; ee: 75%, AD-H, hexane/isopropanol = 70/30, t_R_ = 5.490 (major), t_R_ = 6.753 (minor).

**(*E*)-7-bromo-3-(3,4-diphenylchroman-2-yl)-1*H*-indole (6ra):**

Yield: 89% (42.7 mg); 83:17 dr; white solid; m.p. 87.0–89.0 °C; [α]_D_^20^ = −194.8 (c 0.56, acetone); ^1^H NMR (400 MHz, CDCl_3_) δ 8.04 (s, 1H), 7.75 (d, *J* = 8.0 Hz, 1H), 7.30 (d, *J* = 7.6 Hz, 1H), 7.23 (d, *J* = 7.5 Hz, 1H), 7.20–7.12 (m, 3H), 7.07–6.94 (m, 7H), 6.93–6.88 (m, 1H), 6.73 (d, *J* = 7.3 Hz, 2H), 6.58 (d, *J* = 7.4 Hz, 2H), 5.90 (d, *J* = 10.9 Hz, 1H), 4.40 (d, *J* = 5.2 Hz, 1H), 4.20–4.08 (m, 1H); ^13^C NMR (100 MHz, CDCl_3_) δ 154.7, 141.4, 139.3, 134.9, 130.6, 130.3, 129.1, 128.2, 127.6, 127.5, 127.3, 126.5, 126.4, 124.8, 124.6, 124.3, 121.0, 120.6, 119.2, 116.8, 116.0, 104.8, 71.1, 48.8, 48.2; IR (KBr): 3648, 3628, 3566, 1868, 1716, 1507, 1456, 1339, 751, 668 cm^−1^; (C_29_H_22_BrNO+H)^+^ requires *m*/*z* 480.0958, found *m*/*z* 480.0945; ee: 82%, AD-H, hexane/isopropanol = 70/30, t_R_ = 5.820 (major), t_R_ = 7.357 (minor).

**(*E*)-3-(3-(3-chlorophenyl)-4-phenylchroman-2-yl)-1*H*-indole (6ka):**

Yield: 72% (31.3 mg); 76:24 dr; white solid; m.p. 93.0–95.0 °C; [α]_D_^20^ = −174.8 (c 0.29, acetone); ^1^H NMR (400 MHz, CDCl_3_) δ 7.89 (s, 1H), 7.77 (d, *J* = 7.8 Hz, 1H), 7.25–7.19 (m, 2H), 7.19–7.16 (m, 3H), 7.15–7.08 (m, 2H), 7.07–7.01 (m, 2H), 6.99–6.95 (m, 1H), 6.92–6.82 (m, 3H), 6.78–6.70 (m, 2H), 6.57 (s, 1H), 6.44 (d, *J* = 7.6 Hz, 1H), 5.86 (d, *J* = 10.7 Hz, 1H), 4.38 (d, *J* = 5.2 Hz, 1H), 4.17–4.08 (m, 1H); ^13^C NMR (100 MHz, CDCl_3_) δ 154.8, 141.7, 141.1, 136.3, 133.2, 130.6, 130.3, 129.2, 128.7, 128.3, 127.6, 127.3, 126.7, 126.5, 125.9, 124.3, 123.7, 122.3, 120.6, 119.9, 119.8, 116.9, 114.3, 111.3, 71.0, 48.5, 48.1; IR (KBr): 3648, 3628, 3566, 3030, 1868, 1748, 1507, 1456, 749, 701 cm^−1^; (C_29_H_22_ClNO+H)^+^ requires *m*/*z* 436.1463, found *m*/*z* 436.1462; ee: 80%, AD-H, hexane/isopropanol = 70/30, t_R_ = 5.337 (minor), t_R_ = 6.787 (major).

**(*E*)-3-(3-(4-chlorophenyl)-4-phenylchroman-2-yl)-1*H*-indole (6ma):**

Yield: 70% (30.5 mg); 78:22 dr; white solid; m.p. 97.0–99.0 °C; [α]_D_^20^ = −106.1 (c 0.41, acetone); ^1^H NMR (400 MHz, CDCl_3_) δ 7.89 (s, 1H), 7.76 (d, *J* = 7.8 Hz, 1H), 7.26–7.19 (m, 2H), 7.18–7.15 (m, 3H), 7.15–7.07 (m, 2H), 7.07–7.00 (m, 2H), 6.93–6.86 (m, 4H), 6.74 (d, *J* = 6.9 Hz, 2H), 6.49 (d, *J* = 8.1 Hz, 2H), 5.86 (d, *J* = 10.7 Hz, 1H), 4.37 (d, *J* = 5.2 Hz, 1H), 4.18–4.11 (m, 1H); ^13^C NMR (100 MHz, CDCl_3_) δ 154.8, 141.2, 138.1, 136.3, 132.0, 130.6, 130.4, 130.3, 128.3, 127.6, 126.7, 125.9, 124.5, 123.7, 122.3, 120.5, 119.9, 119.7, 116.9, 114.4, 111.3, 71.1, 48.5, 47.7; IR (KBr): 3648, 3628, 3618, 1868, 1716, 1698, 1507, 1456, 748, 702 cm^−1^; (C_29_H_22_ClNO+H)^+^ requires *m*/*z* 436.1463, found *m*/*z* 436.1449; ee: 83%, IA, hexane/isopropanol = 70/30, t_R_ = 5.587 (minor), t_R_ = 6.463 (major).

**(*E*)-3-(7-methyl-3,4-diphenylchroman-2-yl)-1*H*-indole (6ab):**

Yield: 73% (30.5 mg); 81:19 dr; white solid; m.p. 107.0–109.0 °C; [α]_D_^20^ = −167.9 (c 0.37, acetone); ^1^H NMR (400 MHz, CDCl_3_) δ 7.80 (s, 1H), 7.78 (s, 1H), 7.23–7.19 (m, 1H), 7.18–7.08 (m, 5H), 7.06–7.02 (m, 1H), 7.00–6.92 (m, 4H), 6.86–6.82 (m, 2H), 6.74 (d, *J* = 7.5 Hz, 2H), 6.58 (d, *J* = 7.4 Hz, 2H), 5.91 (d, *J* = 10.7 Hz, 1H), 4.35 (d, *J* = 5.2 Hz, 1H), 4.18–4.10 (m, 1H), 2.24 (s, 3H); ^13^C NMR (100 MHz, CDCl_3_) δ 152.7, 141.6, 139.7, 136.2, 130.6, 130.4, 129.5, 129.1, 128.9, 127.4, 126.4, 126.2, 126.1, 124.4, 123.7, 122.1, 119.9, 119.7, 116.5, 114.8, 111.2, 71.1, 48.8, 48.3, 20.5; IR (KBr): 3648, 3586, 3566, 1868, 1716, 1496, 1456, 1218, 740, 699 cm^−1^; (C_30_H_25_NO+H)^+^ requires *m*/*z* 416.2009, found *m*/*z* 416.1999; ee: 76%, IA, hexane/isopropanol = 70/30, t_R_ = 6.360 (minor), t_R_ = 9.837 (major).

**(*E*)-3-(7-bromo-3,4-diphenylchroman-2-yl)-1*H*-indole (6ac):**

Yield: 57% (27.1 mg); 68:32 dr;

**Major diastereoisomer****:** white solid; m.p. 81.7–82.0 °C; [α]_D_^20^ = −62.5 (c 0.20, acetone); ^1^H NMR (400 MHz, CDCl_3_) δ 8.04 (s, 1H), 7.75 (d, *J* = 7.9 Hz, 1H), 7.30 (d, *J* = 7.6 Hz, 1H), 7.23 (d, *J* = 7.7 Hz, 1H), 7.20–7.11 (m, 3H), 7.07–6.93 (m, 7H), 6.93–6.87 (m, 1H), 6.73 (d, *J* = 7.3 Hz, 2H), 6.58 (d, *J* = 7.3 Hz, 2H), 5.90 (d, *J* = 10.9 Hz, 1H), 4.40 (d, *J* = 5.1 Hz, 1H), 4.18–4.09 (m, 1H); ^13^C NMR (100 MHz, CDCl_3_) δ 155.6, 140.8, 139.2, 136.2, 131.4, 130.5, 129.0, 127.6, 126.6, 126.4, 126.0, 123.9, 123.7, 123.5, 122.3, 121.0, 119.9, 119.8, 119.7, 114.3, 111.3, 71.7, 48.2, 47.8; IR (KBr): 3647, 2922, 2852, 1732, 1716, 1479, 1456, 1261, 741, 700 cm^−1^; (C_29_H_22_BrNO+H)^+^ requires *m*/*z* 480.0958, found *m*/*z* 480.0935; ee: 73%, AD-H, hexane/isopropanol = 70/30, t_R_ = 5.520 (major), t_R_ = 6.530 (minor).

**Minor diastereoisomer****:** white solid; m.p. 85.5–87.0 °C; [α]_D_^20^ = −3.0 (c 0.17, acetone); ^1^H NMR (400 MHz, CDCl_3_) δ 7.84 (s, 1H), 7.68 (d, *J* = 8.5 Hz, 1H), 7.40 (s, 1H), 7.23–7.09 (m, 5H), 7.04–6.93 (m, 6H), 6.89–6.75 (m, 5H), 5.60 (d, *J* = 10.6 Hz, 1H), 4.50 (d, *J* = 11.1 Hz, 1H), 3.71–3.64 (m, 1H); ^13^C NMR (100 MHz, CDCl_3_) δ 155.3, 143.2, 140.4, 136.9, 130.3, 129.1, 128.3, 128.2, 128.1, 127.7, 126.6, 126.5, 126.4, 124.8, 123.8, 123.2, 121.3, 120.8, 116.9, 115.7, 114.8, 114.1, 76.8, 53.1, 50.9; IR (KBr): 3647, 2922, 1575, 1456, 1261, 1214, 1012, 798, 742, 700 cm^−1^; (C_29_H_22_BrNO+H)^+^ requires *m*/*z* 480.0958, found *m*/*z* 480.0931; ee: 60%, OD-H, hexane/isopropanol = 70/30, t_R_ = 6.127 (minor), t_R_ = 7.847 (major).

**(*E*)-3-(4-(4-fluorophenyl)-3-phenylchroman-2-yl)-1*H*-indole (6ad):**

Yield: 59% (24.6 mg); 75:25 dr; white solid; m.p. 105.0–107.0 °C; [α]_D_^20^ = −135.0 (c 0.24, acetone); ^1^H NMR (400 MHz, CDCl_3_) δ 7.87 (s, 1H), 7.78 (d, *J* = 7.7 Hz, 1H), 7.26–7.21 (m, 2H), 7.18–7.08 (m, 2H), 7.05 (d, *J* = 8.2 Hz, 1H), 7.03–6.93 (m, 4H), 6.92–6.86 (m, 2H), 6.85–6.77 (m, 2H), 6.69–6.63 (m, 2H), 6.61 (d, *J* = 7.2 Hz, 2H), 5.87 (d, *J* = 10.6 Hz, 1H), 4.38 (d, *J* = 5.2 Hz, 1H), 4.18–4.10 (m, 1H); ^13^C NMR (100 MHz, CDCl_3_) δ 161.6 (*J* = 243.6 Hz), 154.8, 139.5, 137.3 (*J* = 3.1 Hz), 136.2, 131.9 (*J* = 7.9 Hz), 130.1, 129.1, 128.3, 127.6, 126.4, 126.1, 124.6, 123.7, 123.1, 122.2, 120.5, 119.8 (*J* = 7.7 Hz), 116.9, 114.6, 114.3 (*J* = 21.0 Hz), 111.2, 71.1, 48.0, 47.9; ^19^F NMR (376 MHz, CDCl_3_) δ -116.50; IR (KBr): 3648, 3628, 3618, 1771, 1716, 1653, 1488, 1456, 748, 699 cm^−1^; (C_29_H_22_FNO+H)^+^ requires *m*/*z* 420.1758, found *m*/*z* 420.1760; ee: 76%, AD-H, hexane/isopropanol = 70/30, t_R_ = 5.360 (minor), t_R_ = 6.863 (major).

**(*E*)-3-(4-(3-methoxyphenyl)-3-phenylchroman-2-yl)-1*H*-indole (6ae):**

Yield: 80% (34.6 mg); 75:25 dr; white solid; m.p. 92.0–94.0 °C; [α]_D_^20^ = −170.3 (c 0.27, acetone); ^1^H NMR (400 MHz, CDCl_3_) δ 7.85 (s, 1H), 7.79 (d, *J* = 7.7 Hz, 1H), 7.25–7.20 (m, 2H), 7.16–7.08 (m, 2H), 7.07–7.05 (m, 1H), 7.05–7.01 (m, 2H), 7.01–6.94 (m, 3H), 6.92–6.86 (m, 2H), 6.74–6.68 (m, 1H), 6.64 (d, *J* = 7.3 Hz, 2H), 6.38 (d, *J* = 7.6 Hz, 1H), 6.15 (s, 1H), 5.94 (d, *J* = 10.6 Hz, 1H), 4.37 (d, *J* = 5.2 Hz, 1H), 4.19–4.11 (m, 1H), 3.54 (s, 3H); ^13^C NMR (100 MHz, CDCl_3_) δ 158.7, 154.8, 143.1, 139.7, 136.2, 130.2, 129.1, 128.4, 128.2, 127.5, 126.2, 126.1, 124.6, 123.7, 123.1, 122.2, 120.4, 119.9, 119.8, 116.8, 116.1, 114.7, 112.4, 111.2, 71.2, 55.0, 48.7, 48.0; IR (KBr): 3750, 3648, 3618, 3566, 1583, 1486, 1456, 1241, 744, 702 cm^−1^; (C_30_H_25_NO_2_+H)^+^ requires *m*/*z* 432.1958, found *m*/*z* 432.1949; ee: 79%, IA, hexane/isopropanol = 70/30, t_R_ = 5.743 (minor). t_R_ = 6.993 (major).

**(*E*)-3-(6-methoxy-4-(2-methoxyphenyl)-3-phenylchroman-2-yl)-1*H*-indole (6af):**

Yield: 71% (32.7 mg); 90:10 dr; white solid; m.p. 110.0–112.0 °C; [α]_D_^20^ = −133.8 (c 0.24, acetone); ^1^H NMR (400 MHz, CDCl_3_) δ 7.81 (d, *J* = 7.6 Hz, 1H), 7.78 (s, 1H), 7.21–7.13 (m, 2H), 7.14–7.02 (m, 3H), 6.97 (d, *J* = 8.9 Hz, 1H), 6.93–6.85 (m, 4H), 6.84 (d, *J* = 2.1 Hz, 1H), 6.82–6.77 (m, 1H), 6.63–6.55 (m, 3H), 6.53 (d, *J* = 2.9 Hz, 1H), 5.86 (d, *J* = 10.5 Hz, 1H), 5.05 (d, *J* = 5.4 Hz, 1H), 4.15–4.06 (m, 1H), 3.68 (s, 3H), 3.09 (s, 3H); ^13^C NMR (100 MHz, CDCl_3_) δ 157.5, 153.4, 149.5, 140.3, 136.2, 131.3, 130.6, 128.8, 127.6, 127.0, 126.2, 125.8, 125.5, 123.6, 122.0, 120.0, 119.9, 119.6, 117.3, 115.0, 114.7, 114.2, 111.1, 109.7, 71.2, 55.7, 54.6, 47.8, 40.0; IR (KBr): 3648, 3628, 3618, 1868, 1456, 1435, 1238, 1031, 749, 698 cm^−1^; (C_31_H_27_NO_3_+H)^+^ requires *m*/*z* 462.2064, found *m*/*z* 462.2069; ee: 81%, IA, hexane/isopropanol = 70/30, t_R_ = 5.823 (major), t_R_ = 7.830 (minor).
