# Supplementary material for: Organocatalytic Asymmetric [2 + 4] Cycloadditions of 3-Vinylindoles with ortho-Quinone Methides
Source: Molecules. 2021 Nov 8;26(21):6751. doi: 10.3390/molecules26216751 (PMC8587149; doi:10.3390/molecules26216751)
Supplement: Supplementary file 1 [file molecules-26-06751-s001.zip › molecules-1347509-supplementary.pdf]

Supporting Information for

Organocatalytic Asymmetric [2 + 4] Cycloadditions of 3-Vinylindoles with *ortho*-Quinone Methides

Si-Jia Liu[a], Man-Su Tu[a], Kai-Yue Liu[a], Jia-Yi Chen[b], Shao-Fei Ni*[b], Yu-Chen Zhang*[a] and Feng Shi*[a]

[a]*School of Chemistry and Materials Science, Jiangsu Normal University, Xuzhou, 221116, China*

[b]*Department of Chemistry, Key Laboratory for Preparation and Application of Ordered Structural Materials of Guangdong Province, Shantou University, Shantou, 515063, China*

E-mail: *fshi@jsnu.edu.cn; zhangyc@jsnu.edu.cn; sfni@stu.edu.cn*

Contents:

1. NMR spectra of products 3 (S2-S23)

2. NMR spectra of products 6 (S24-S39)

3. HPLC spectra of products 3 (S40-S61)

4. HPLC spectra of products 6 (S62-S76)

5. NOE spectrum of product 6ma (S77)

6. X-ray single-crystal data for product 3na (S78-S79)

7. Theoretical calculations of the reaction pathway (S80-S98)

1. NMR spectra of substrates 3

^{1}H NMR (400 MHz, $CDCl_3$) of compound **3aa**:(inseparable diastereomers, 89:11 dr)

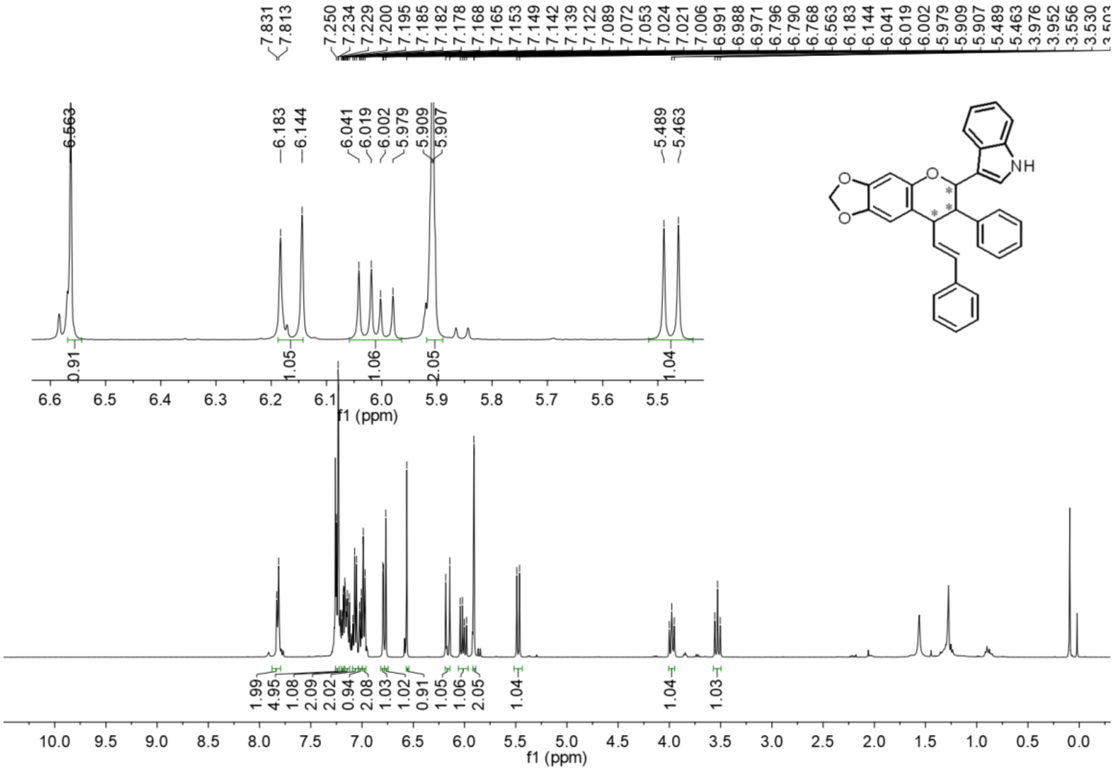

^{13}C NMR (100 MHz, $CDCl_3$) of compound **3aa**: (inseparable diastereomers, 89:11 dr)

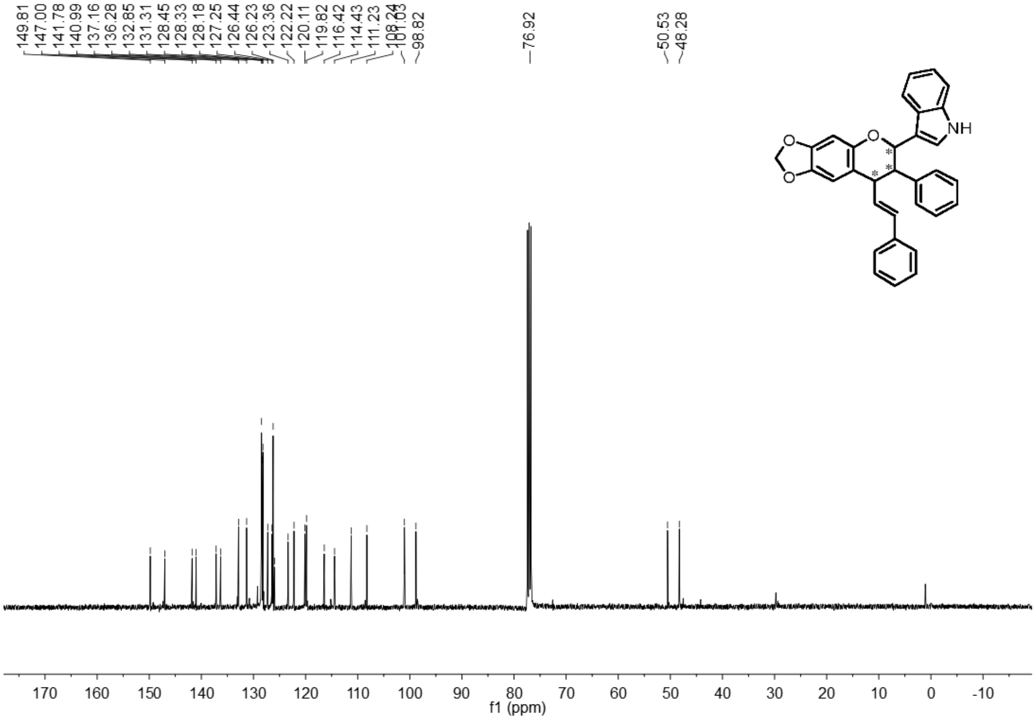

^{1}H NMR (400 MHz, $CDCl_3$) of compound **3ba**: (inseparable diastereomers, 83:17 dr)

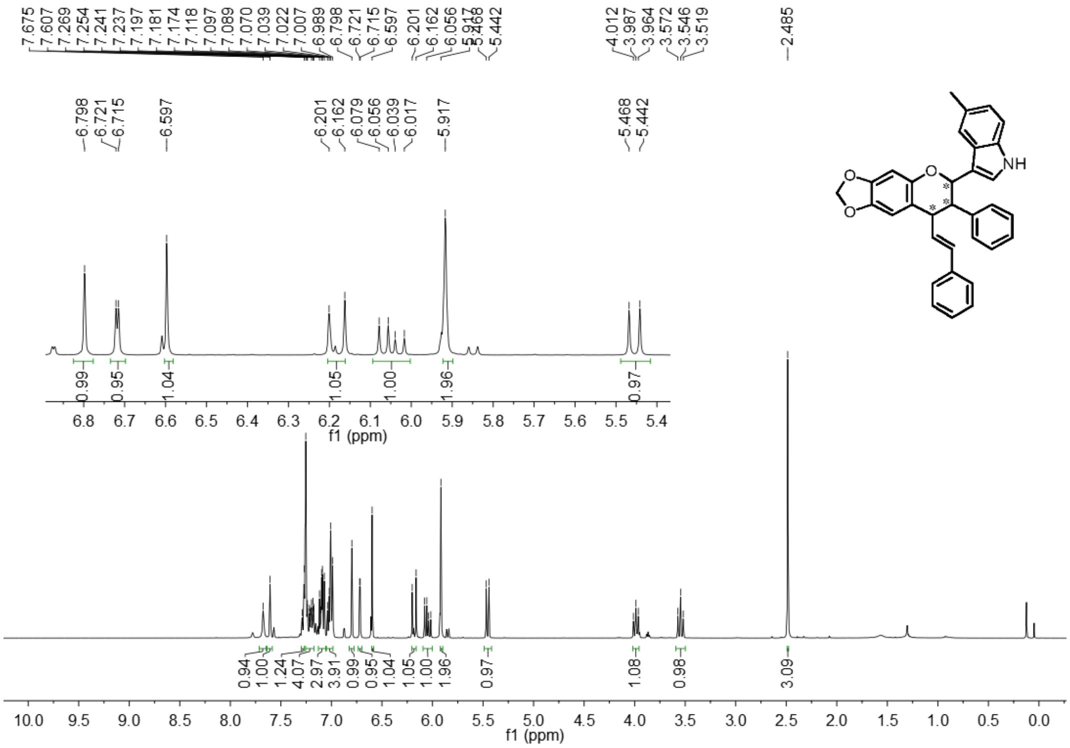

^{13}C NMR (100 MHz, $CDCl_3$) of compound **3ba**: (inseparable diastereomers, 83:17 dr)

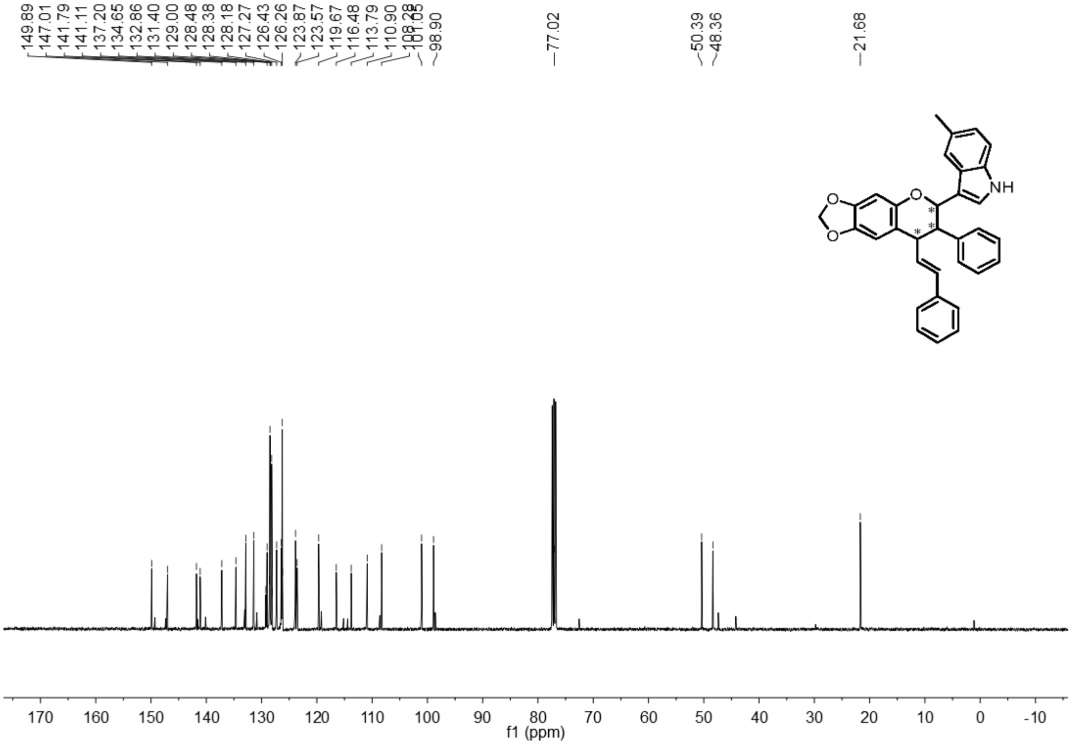

^{1}H NMR (400 MHz, $CDCl_3$) of compound **3ca**: (inseparable diastereomers, 86:14 dr)

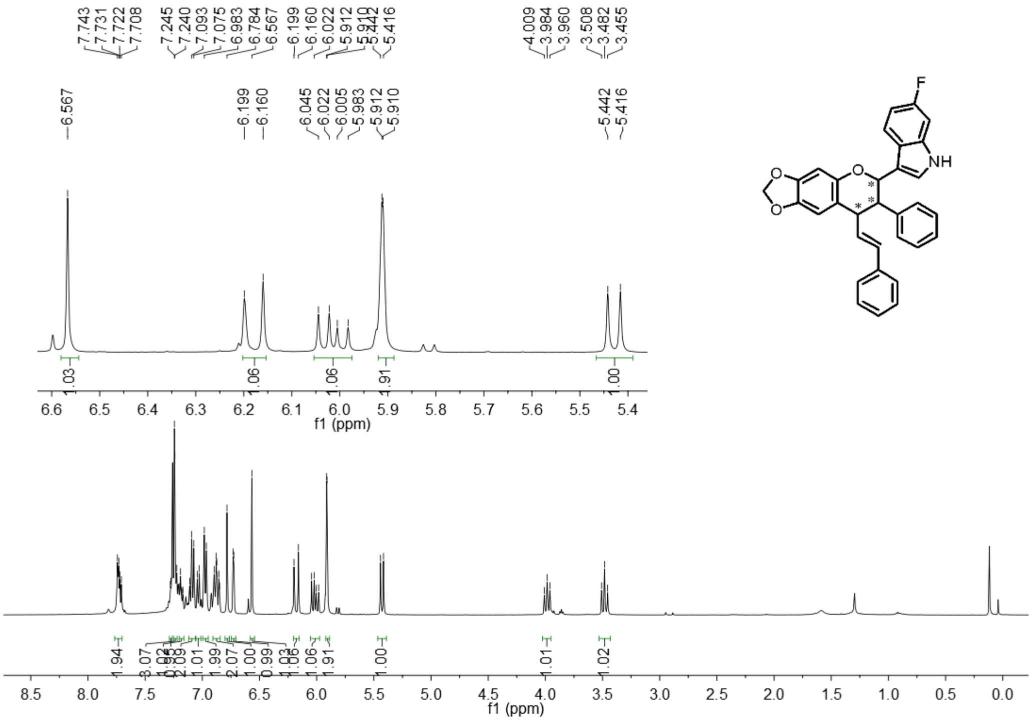

^{13}C NMR (100 MHz, $CDCl_3$) of compound **3ca**: (inseparable diastereomers, 86:14 dr)

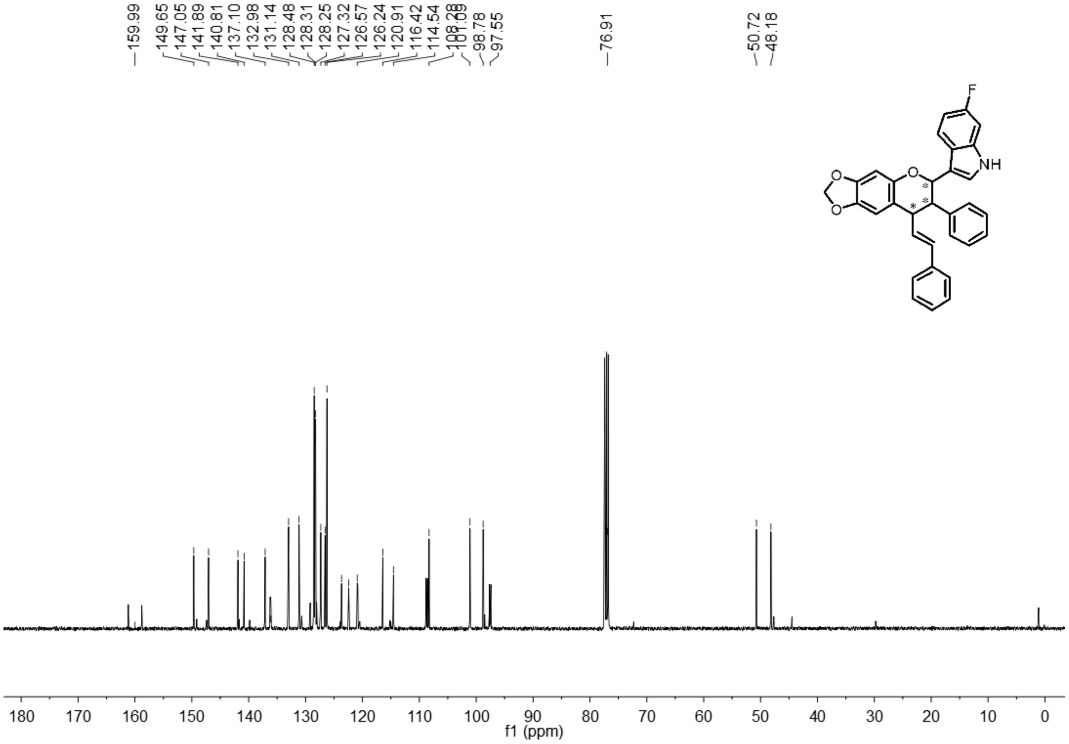

^{1}H NMR (400 MHz, $CDCl_3$) of compound **3da**: (inseparable diastereomers, 91:9 dr)

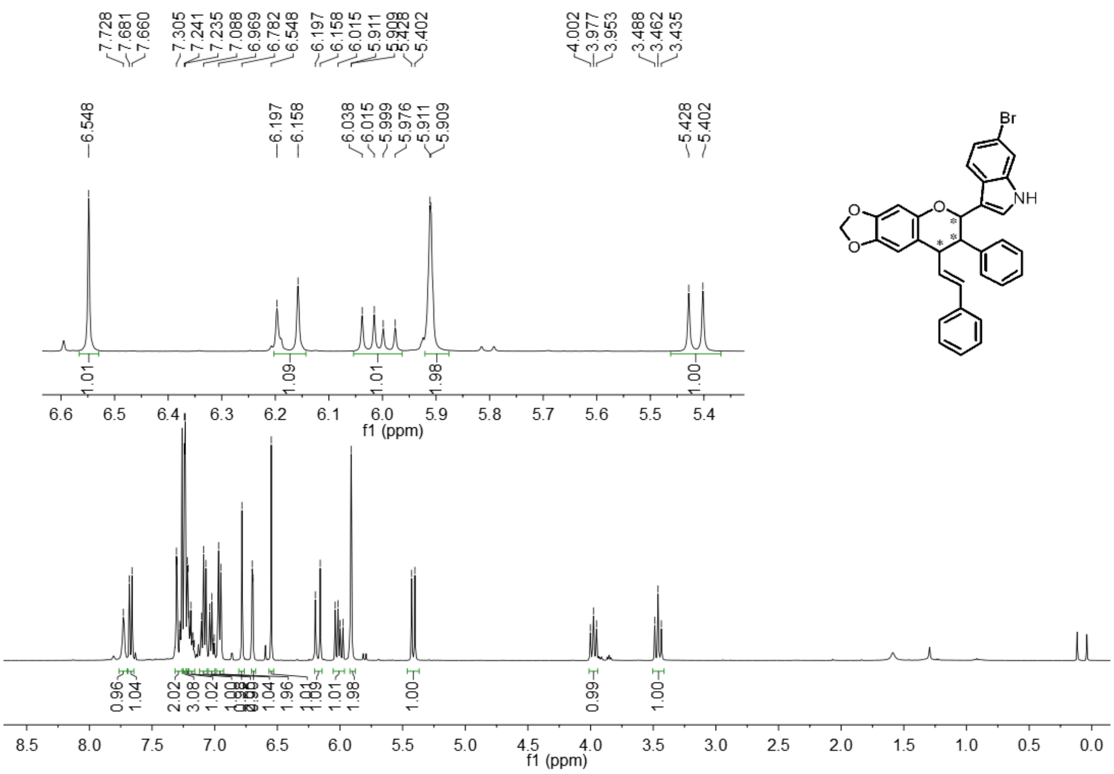

^{13}C NMR (100 MHz, $CDCl_3$) of compound **3da**: (inseparable diastereomers, 91:9 dr)

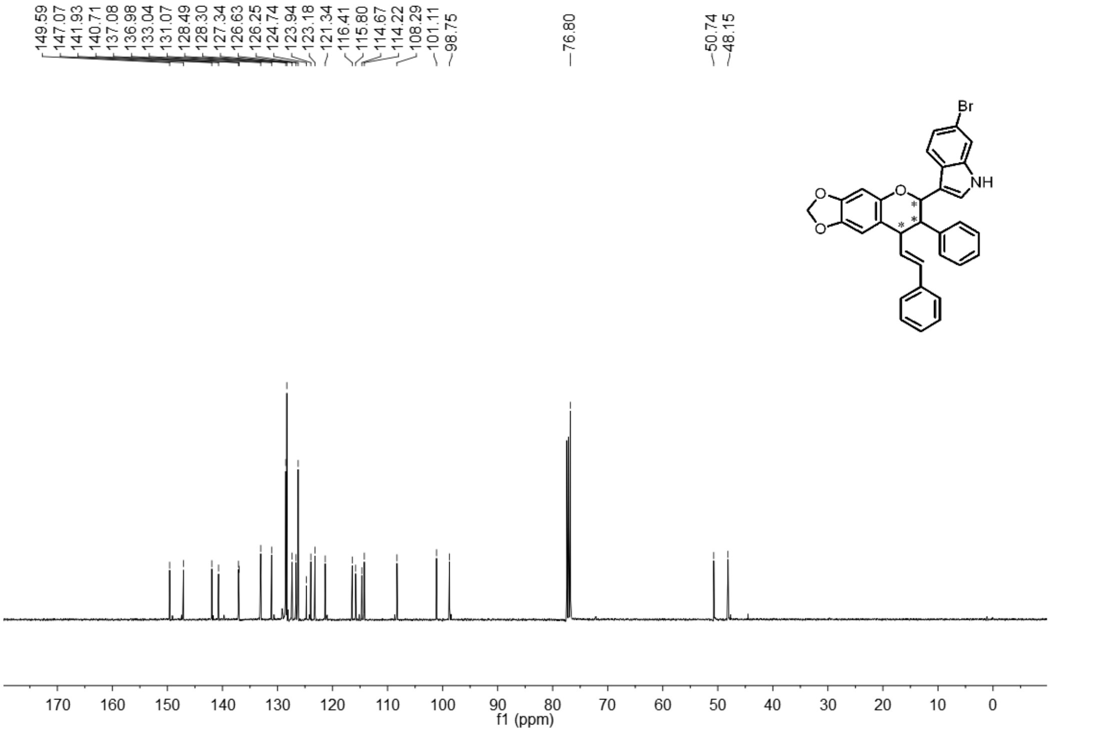

^{1}H NMR (400 MHz, $CDCl_3$) of compound **3ea**: (inseparable diastereomers, 85:15 dr)

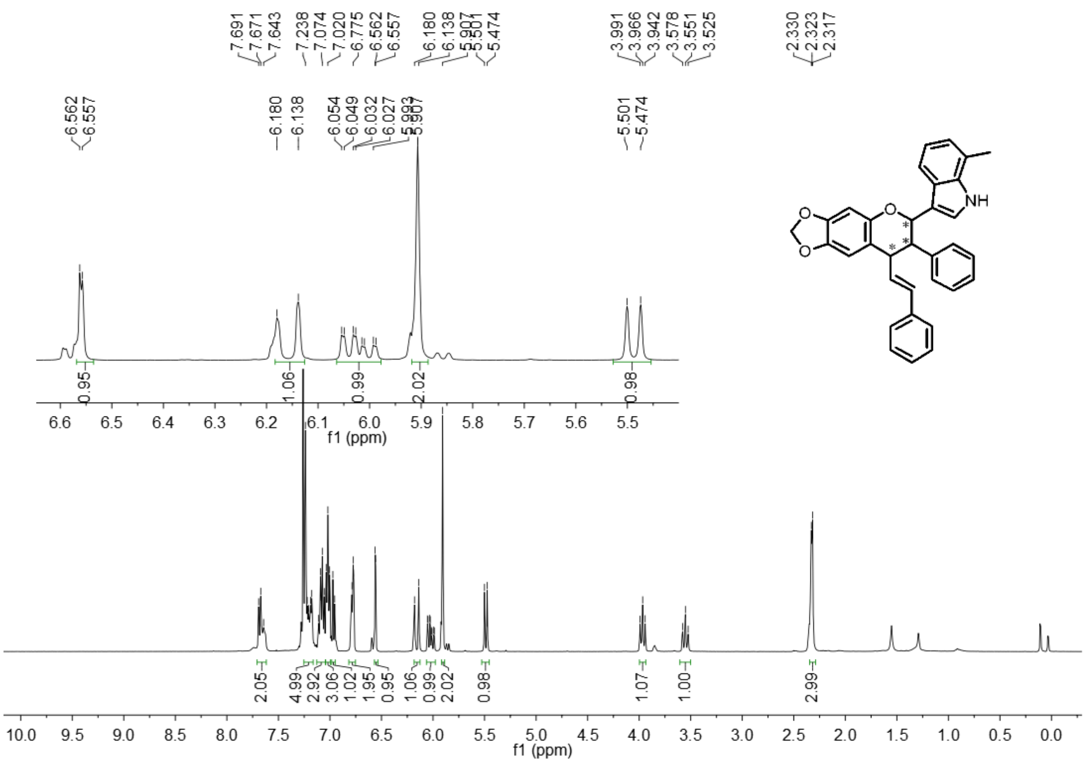

^{13}C NMR (100 MHz, $CDCl_3$) of compound **3ea**: (inseparable diastereomers, 85:15 dr)

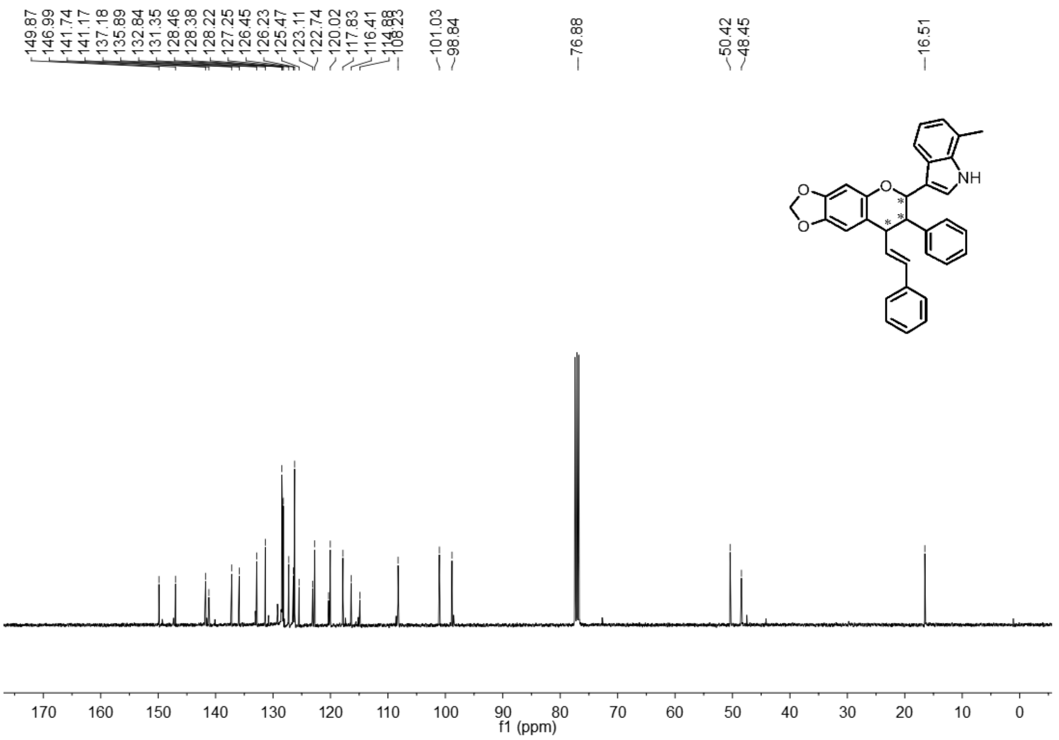

^{1}H NMR (400 MHz, $CDCl_3$) of compound **3fa**: (inseparable diastereomers, 88:12 dr)

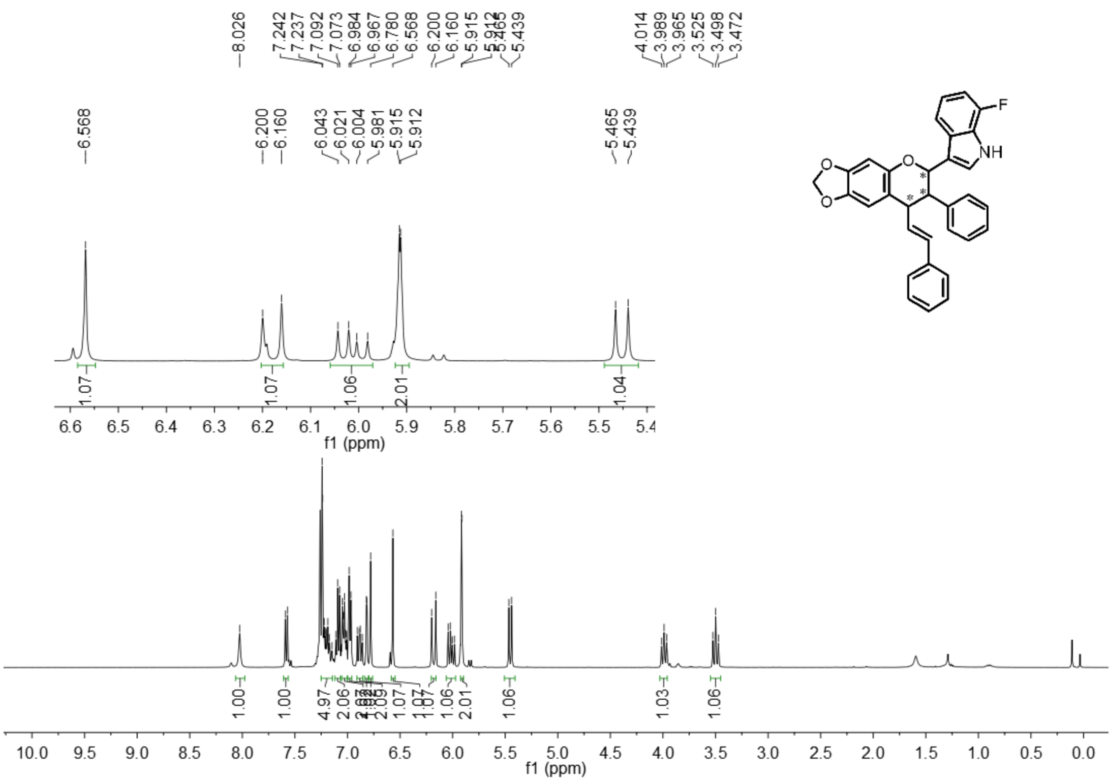

^{13}C NMR (100 MHz, $CDCl_3$) of compound **3fa**: (inseparable diastereomers, 88:12 dr)

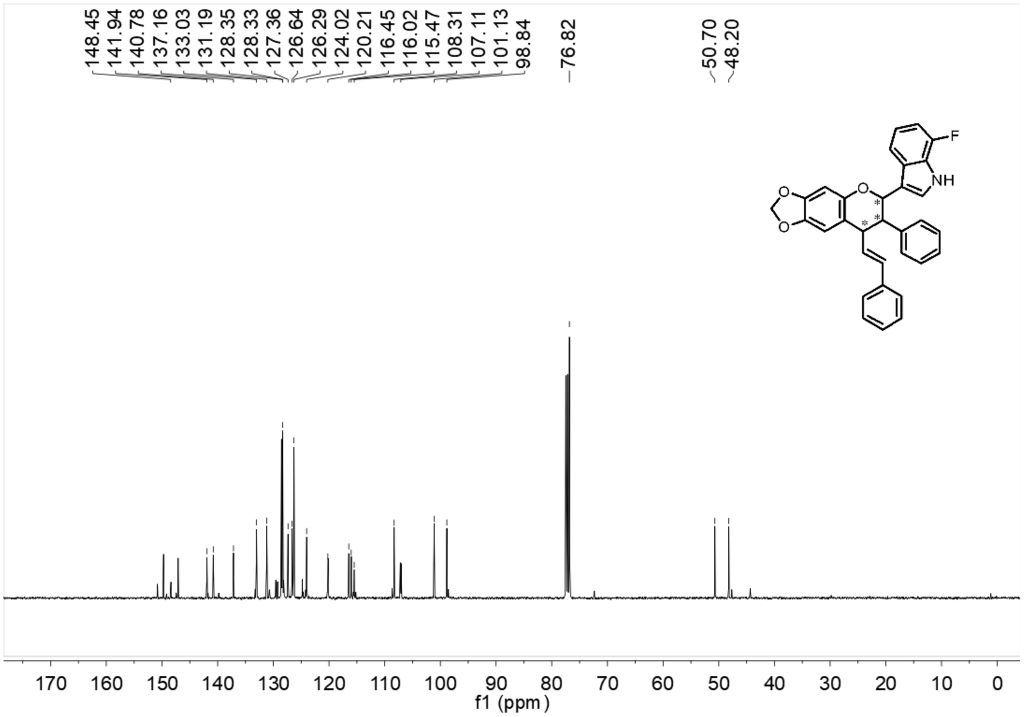

^{1}H NMR (400 MHz, $CDCl_3$) of compound **3ga**: (inseparable diastereomers, 93:7 dr)

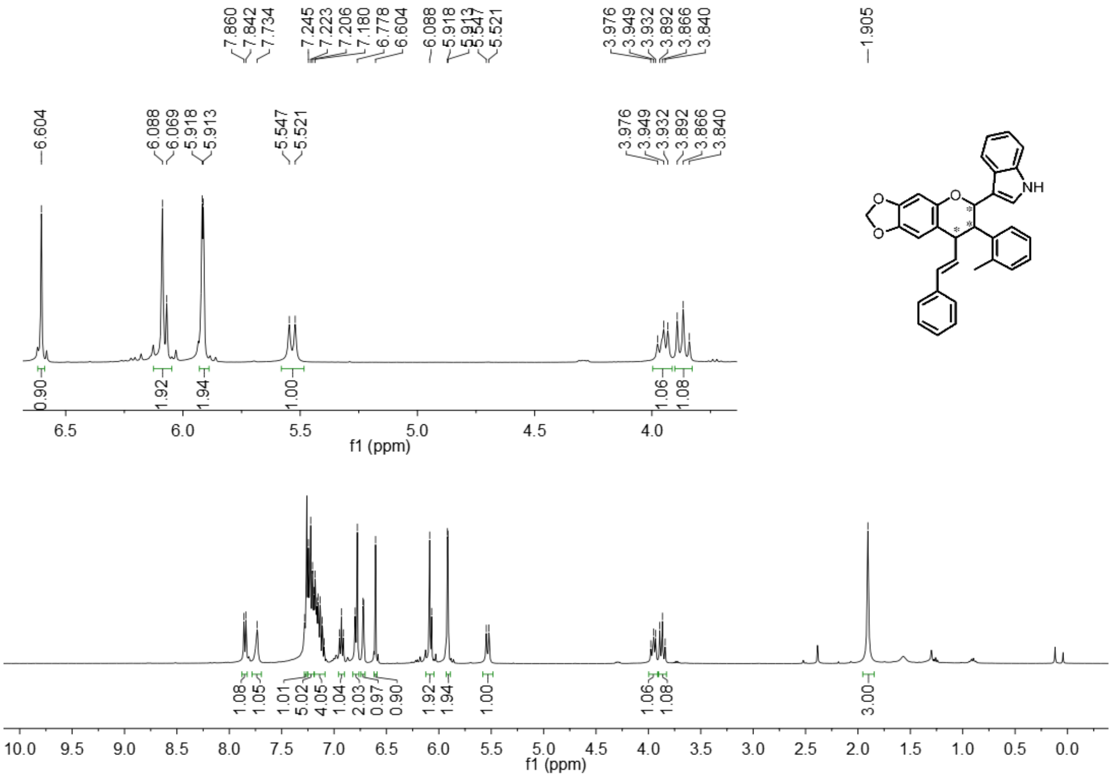

^{13}C NMR (100 MHz, $CDCl_3$) of compound **3ga**: (inseparable diastereomers, 93:7 dr)

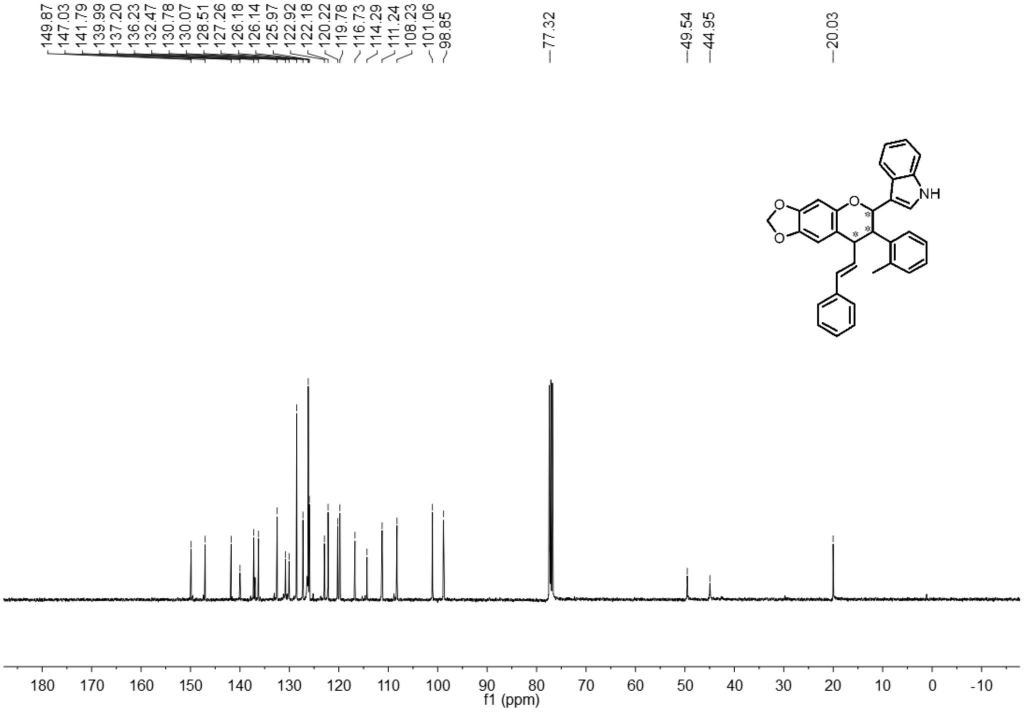

^{1}H NMR (400 MHz, $CDCl_3$) of compound **3ha**: (inseparable diastereomers, 91:9 dr)

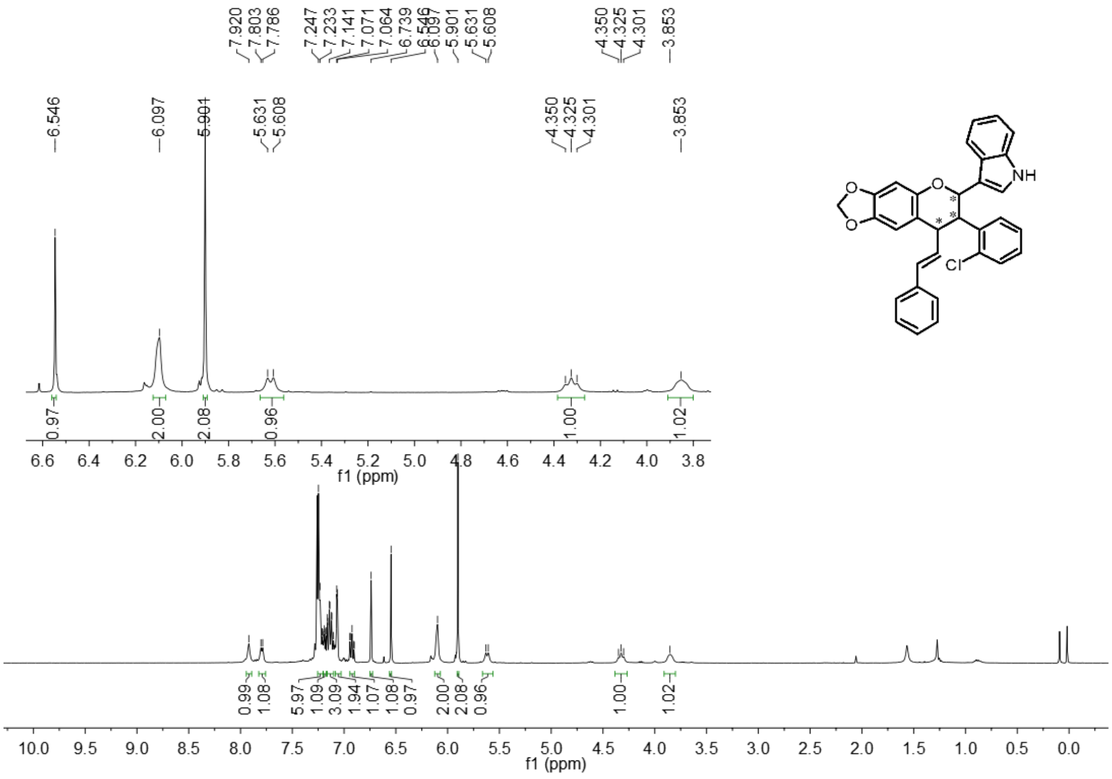

^{13}C NMR (100 MHz, $CDCl_3$) of compound **3ha**: (inseparable diastereomers, 91:9 dr)

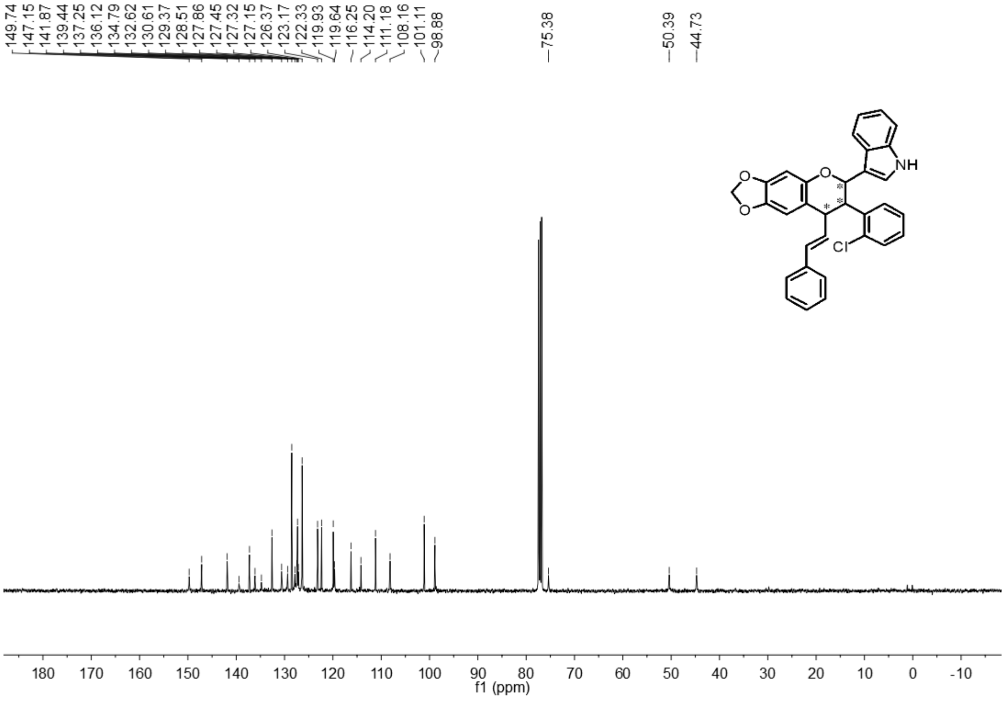

^{1}H NMR (400 MHz, $CDCl_3$) of compound **3ia**: (inseparable diastereomers, 92:8 dr)

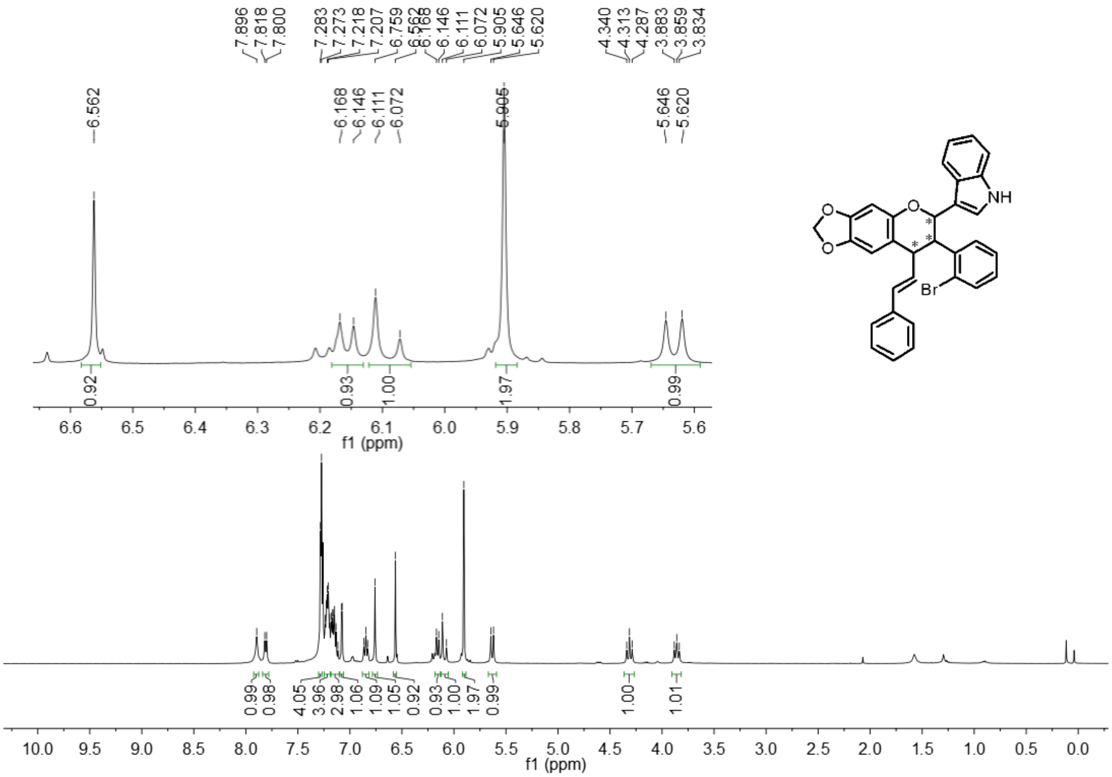

^{13}C NMR (100 MHz, $CDCl_3$) of compound **3ia**: (inseparable diastereomers, 92:8 dr)

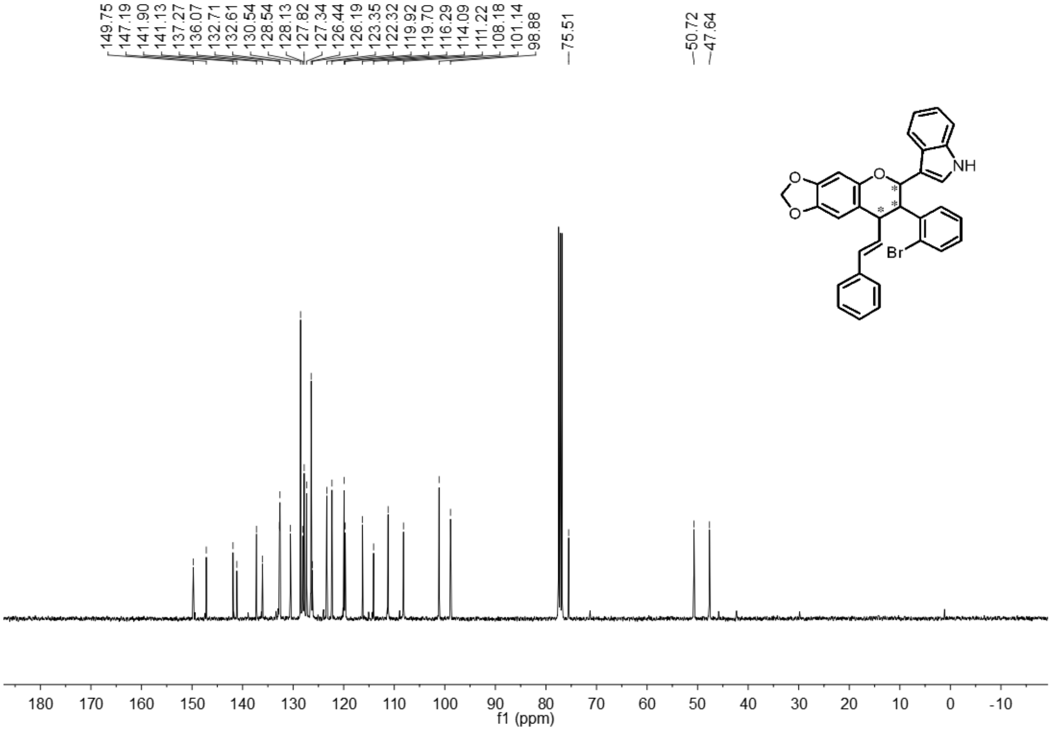

^{1}H NMR (400 MHz, $CDCl_3$) of compound **3ja**: (inseparable diastereomers, 84:16 dr)

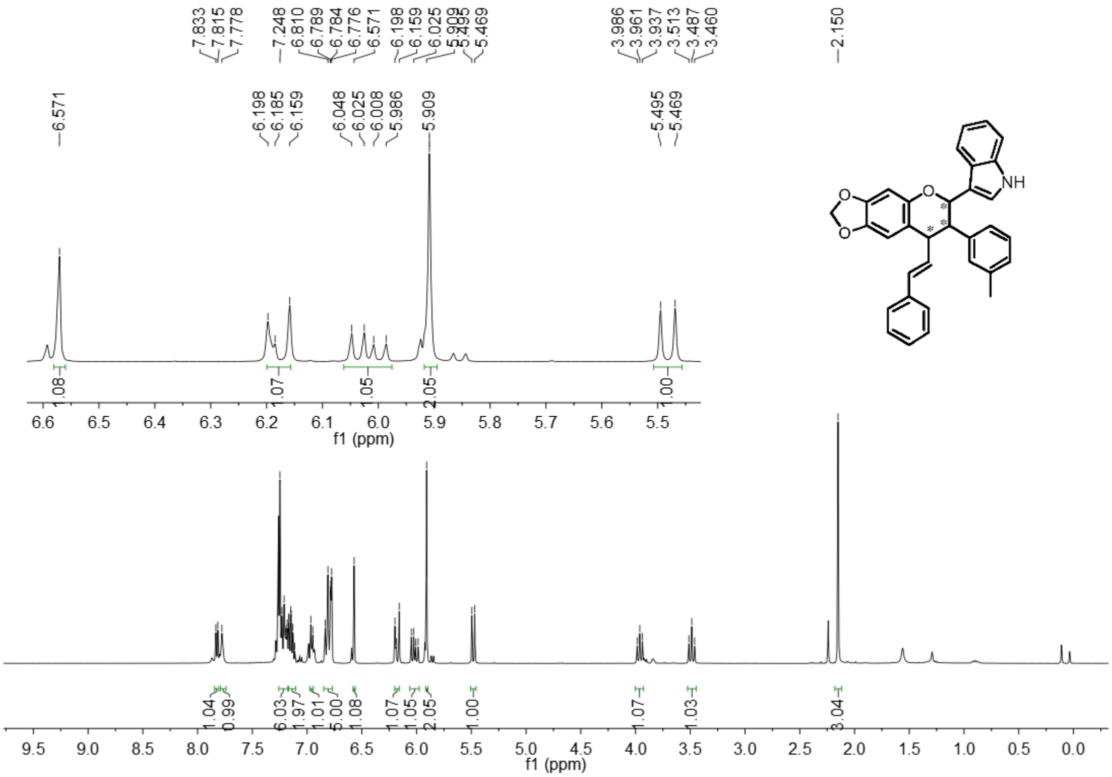

^{13}C NMR (100 MHz, $CDCl_3$) of compound **3ja**: (inseparable diastereomers, 84:16 dr)

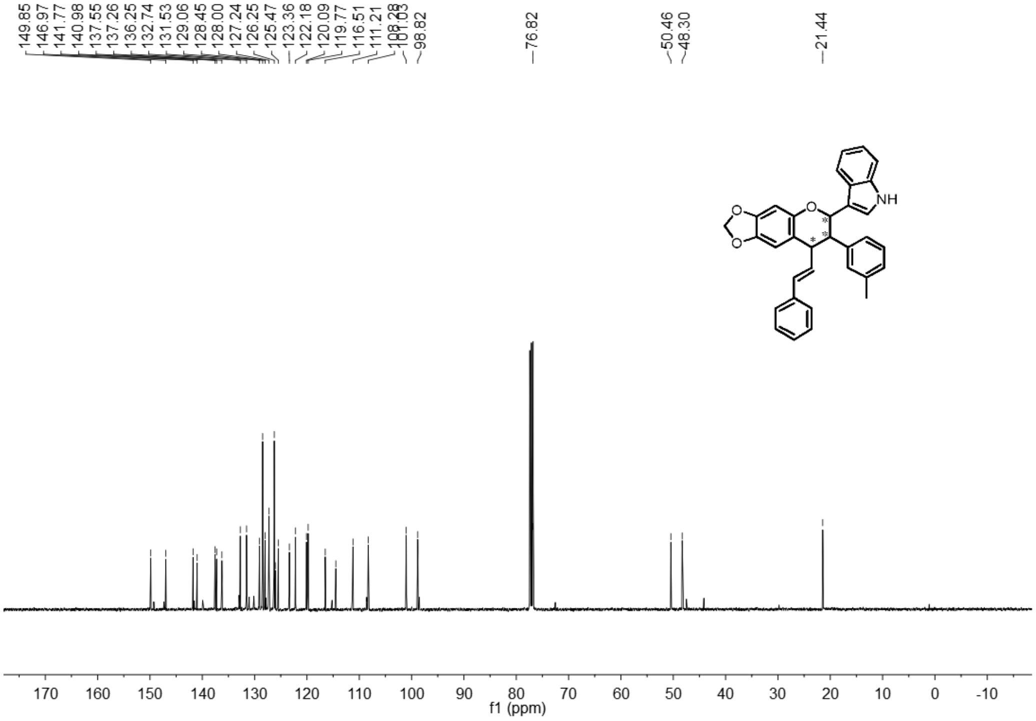

^{1}H NMR (400 MHz, $CDCl_3$) of compound **3ka**: (inseparable diastereomers, 87:13 dr)

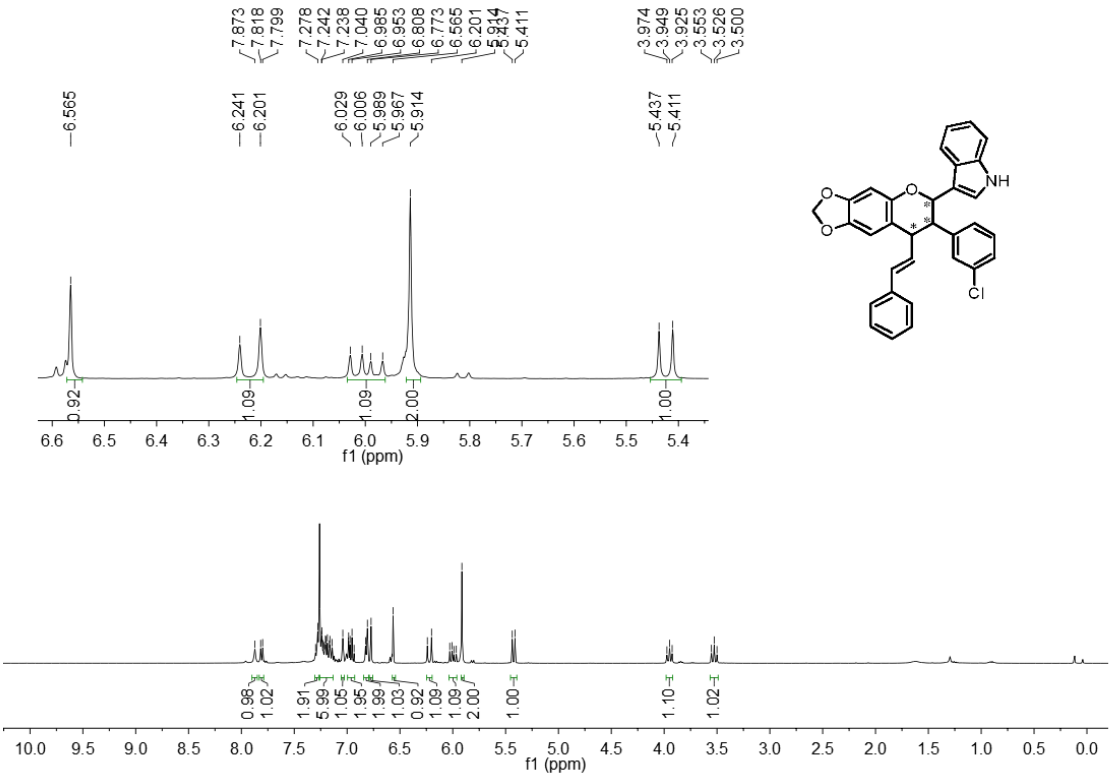

^{13}C NMR (100 MHz, $CDCl_3$) of compound **3ka:** (inseparable diastereomers, 87:13 dr)

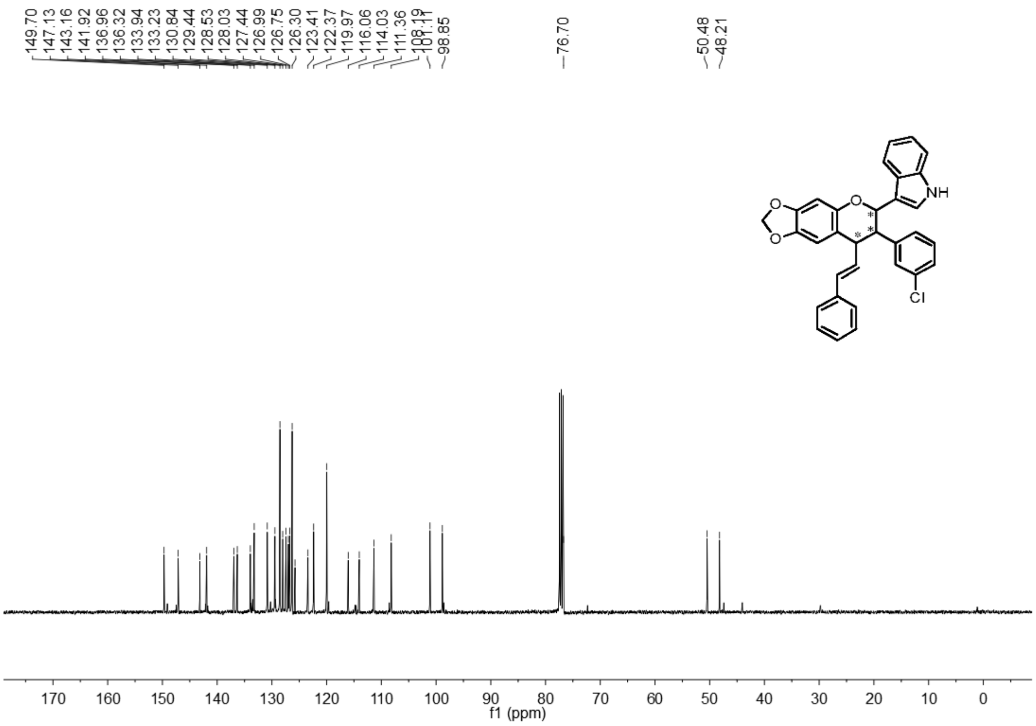

^{1}H NMR (400 MHz, $CDCl_3$) of compound **3la**: (inseparable diastereomers, 78:22 dr)

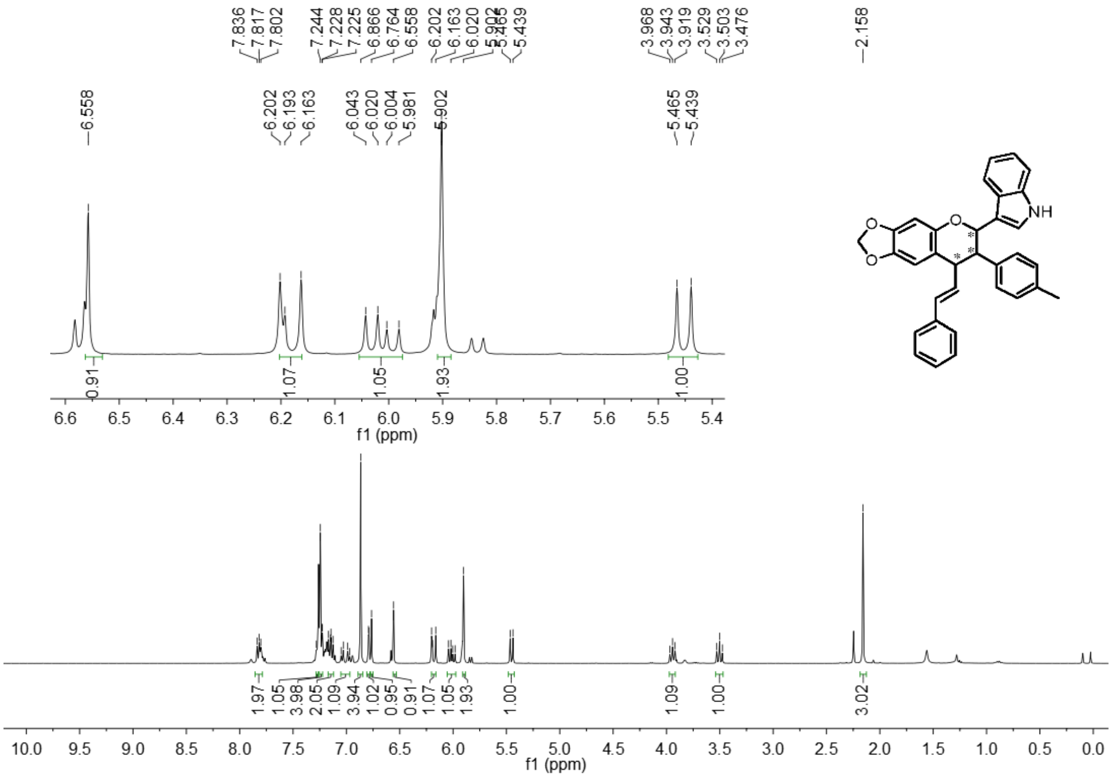

^{13}C NMR (100 MHz, $CDCl_3$) of compound **3la**: (inseparable diastereomers, 78:22 dr)

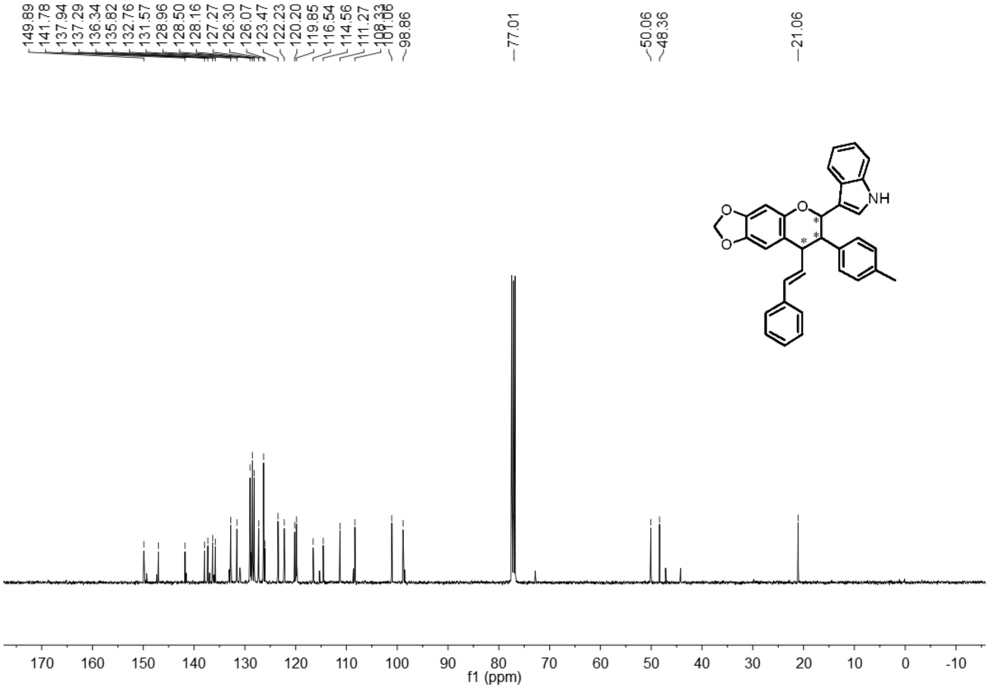

^{1}H NMR (400 MHz, $CDCl_3$) of compound **3ma**: (inseparable diastereomers, 84:16 dr)

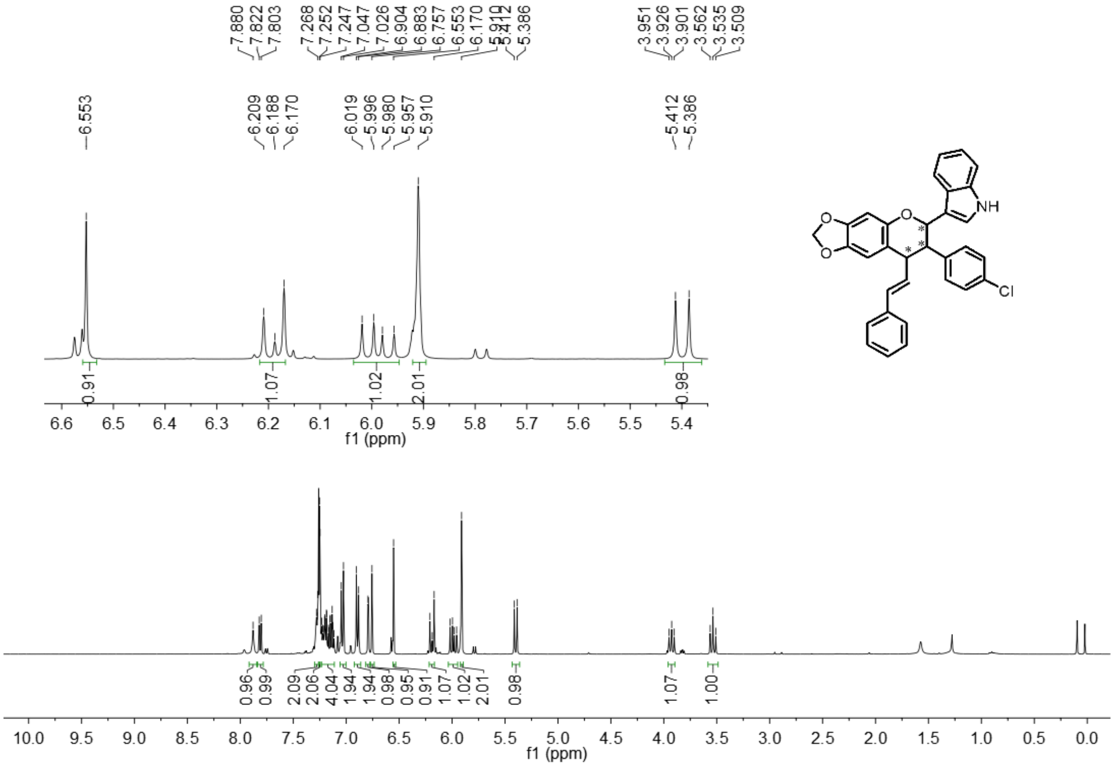

^{13}C NMR (100 MHz, $CDCl_3$) of compound **3ma**: (inseparable diastereomers, 84:16 dr)

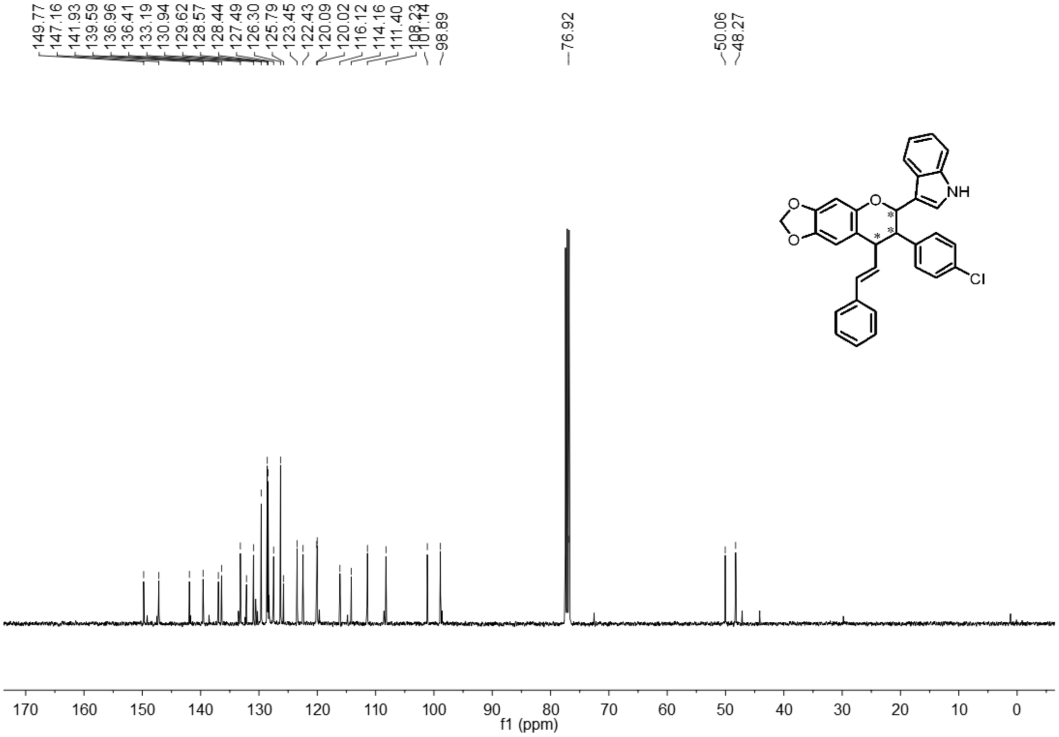

^{1}H NMR (400 MHz, acetone-d_6) of **3na:** (inseparable diastereomers, 86:14 dr)

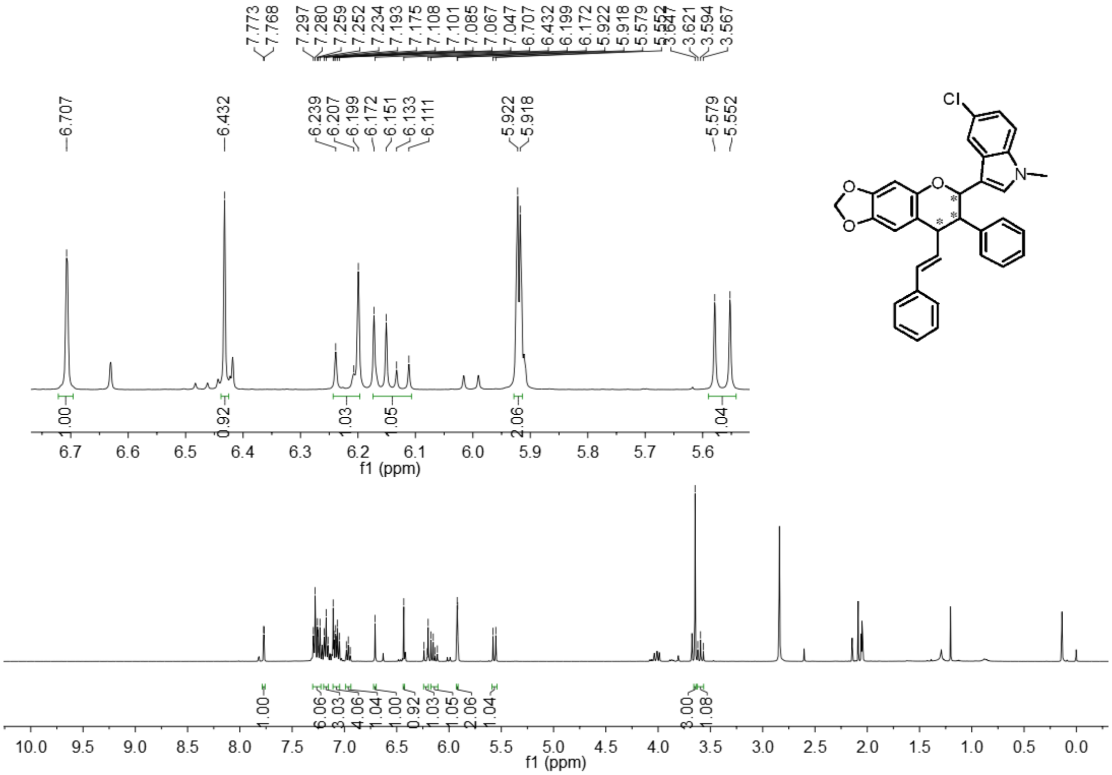

^{13}C NMR (100 MHz, acetone-d_6) of **3na**: (inseparable diastereomers, 86:14 dr)

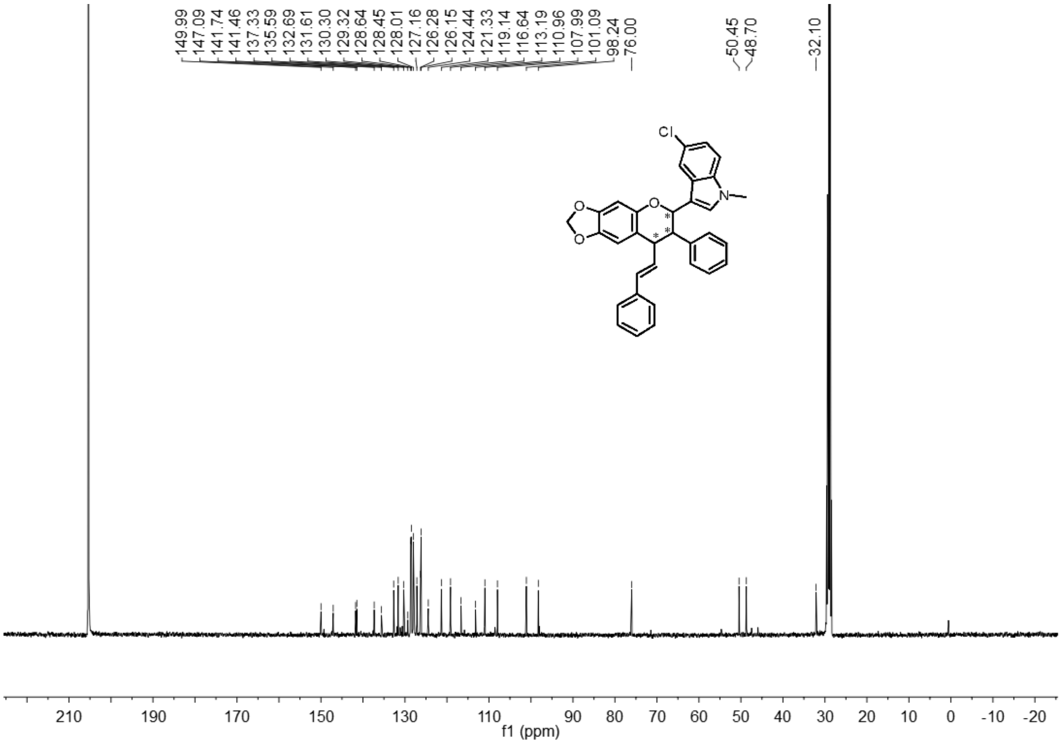

^{1}H NMR (400 MHz, $CDCl_3$) of compound **3oa**: (inseparable diastereomers, 91:9 dr)

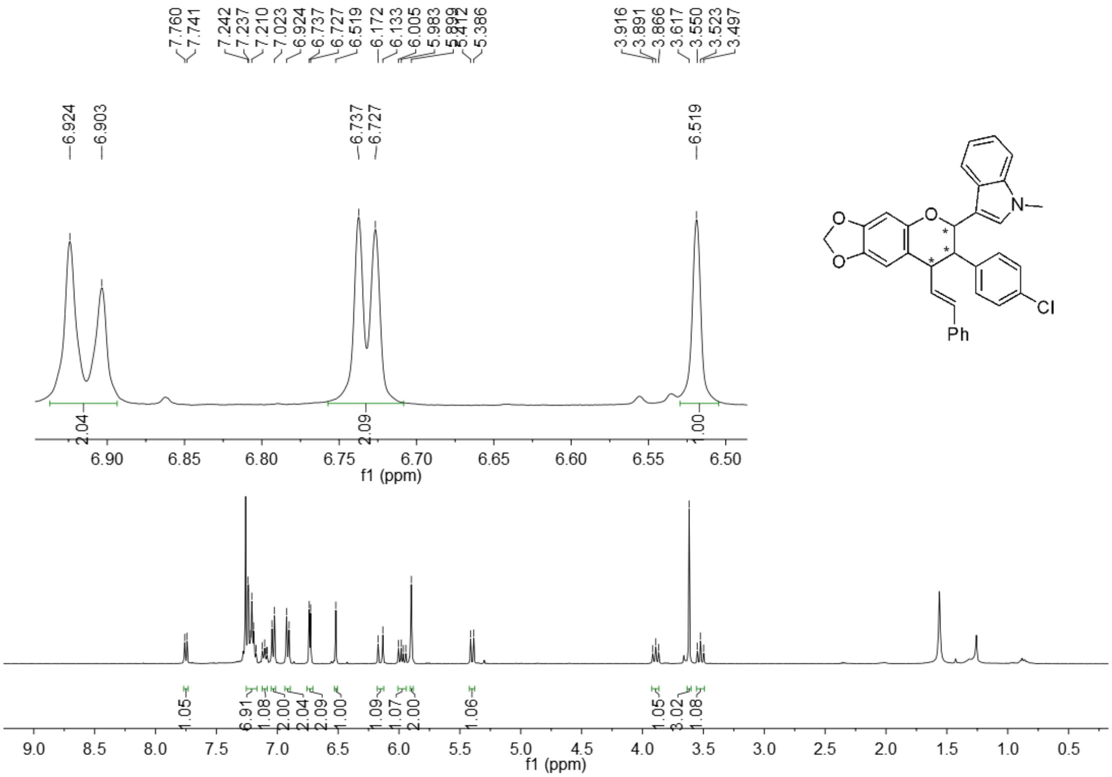

^{13}C NMR (100 MHz, $CDCl_3$) of compound **3oa**: (inseparable diastereomers, 91:9 dr)

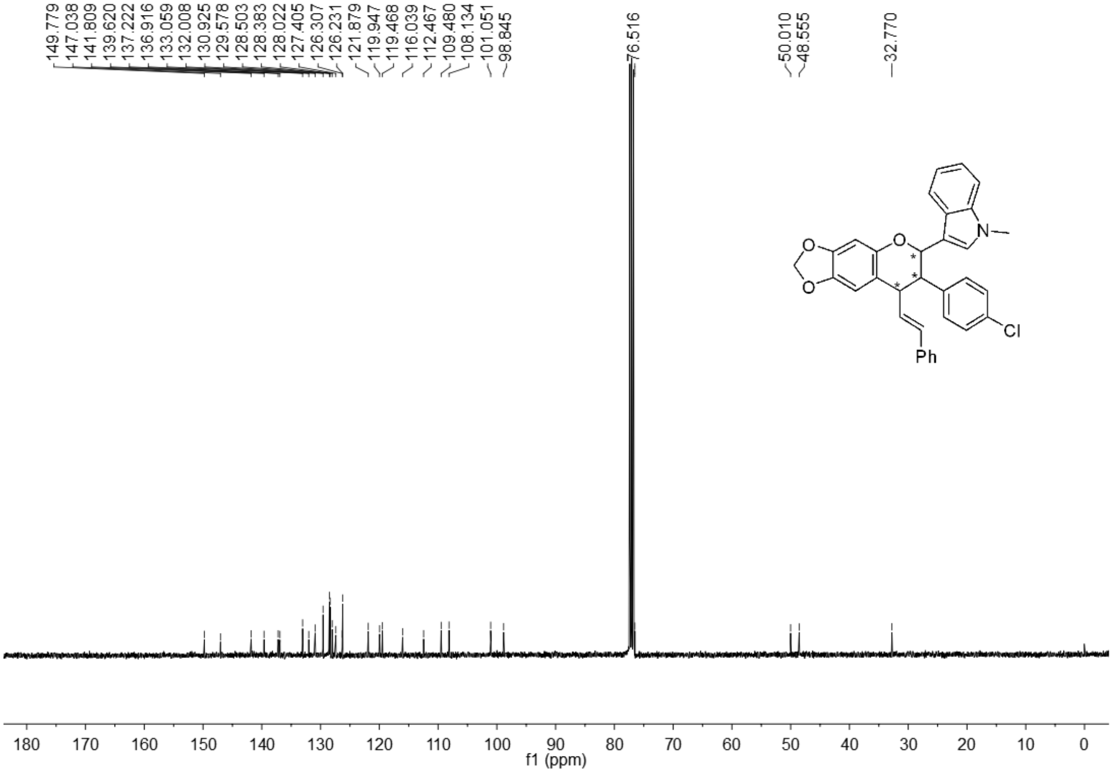

^{1}H NMR (400 MHz, $CDCl_3$) of compound **3ab:** (inseparable diastereomers, 88:12 dr)

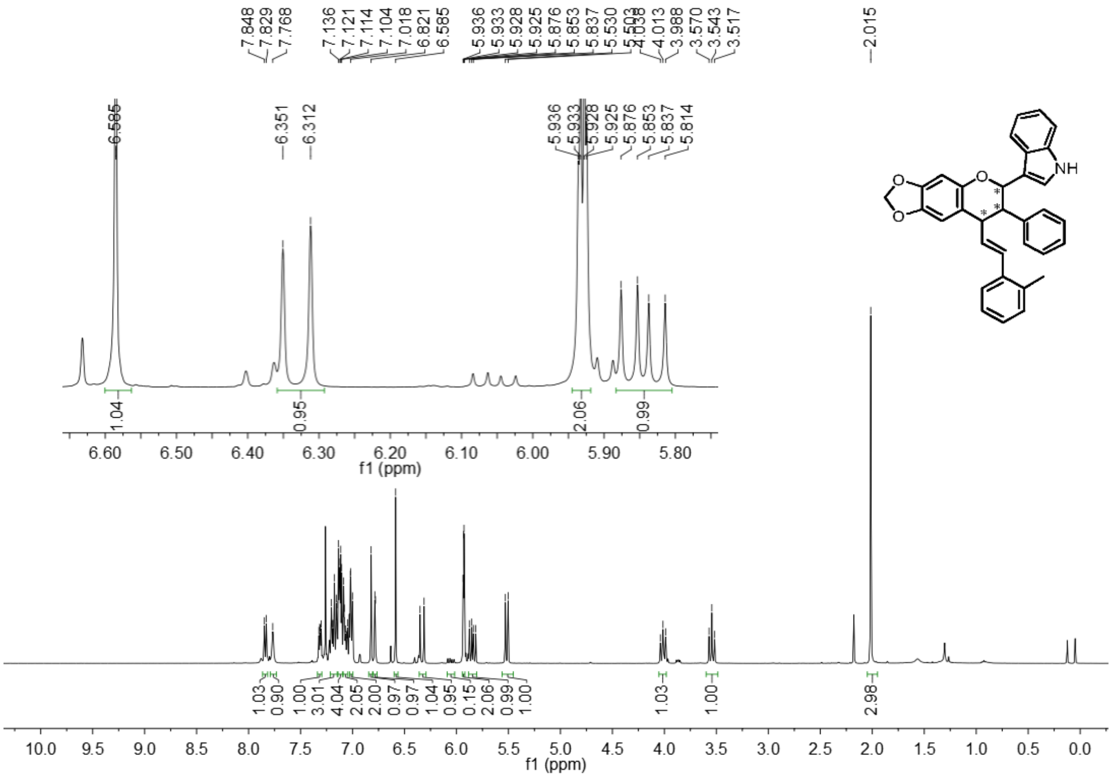

^{13}C NMR (100 MHz, $CDCl_3$) of compound **3ab**: (inseparable diastereomers, 88:12 dr)

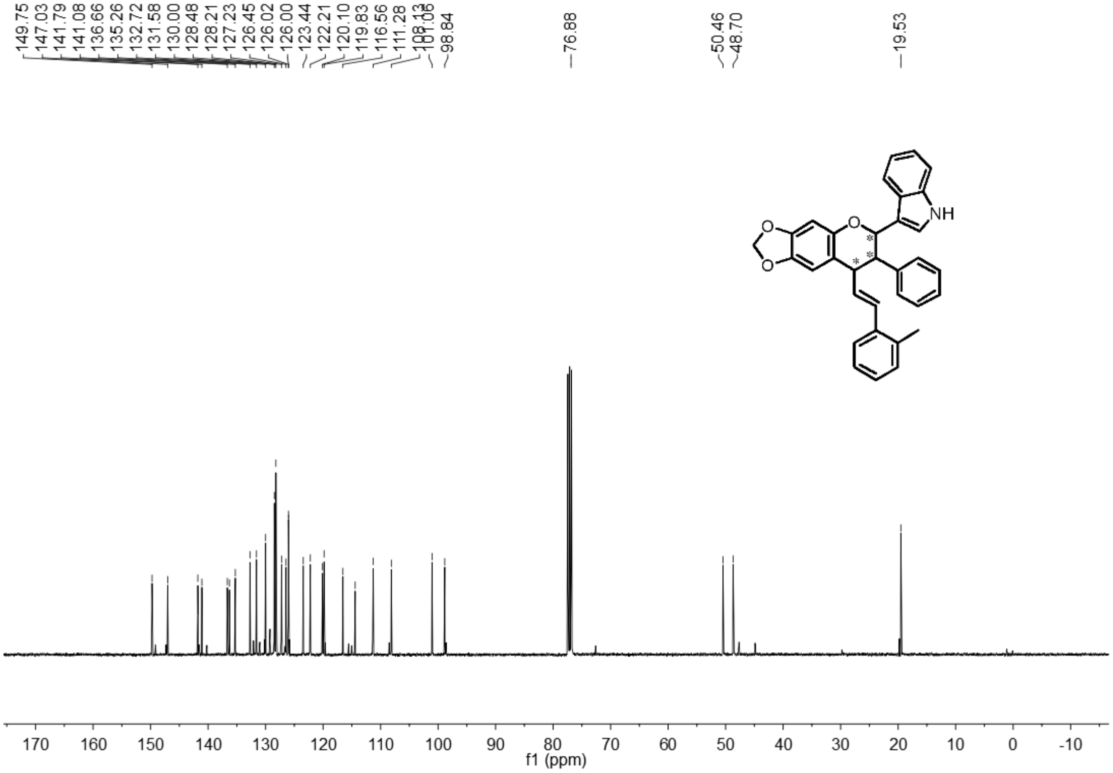

^{1}H NMR (400 MHz, $CDCl_3$) of compound **3ac:** (inseparable diastereomers, 86:14 dr)

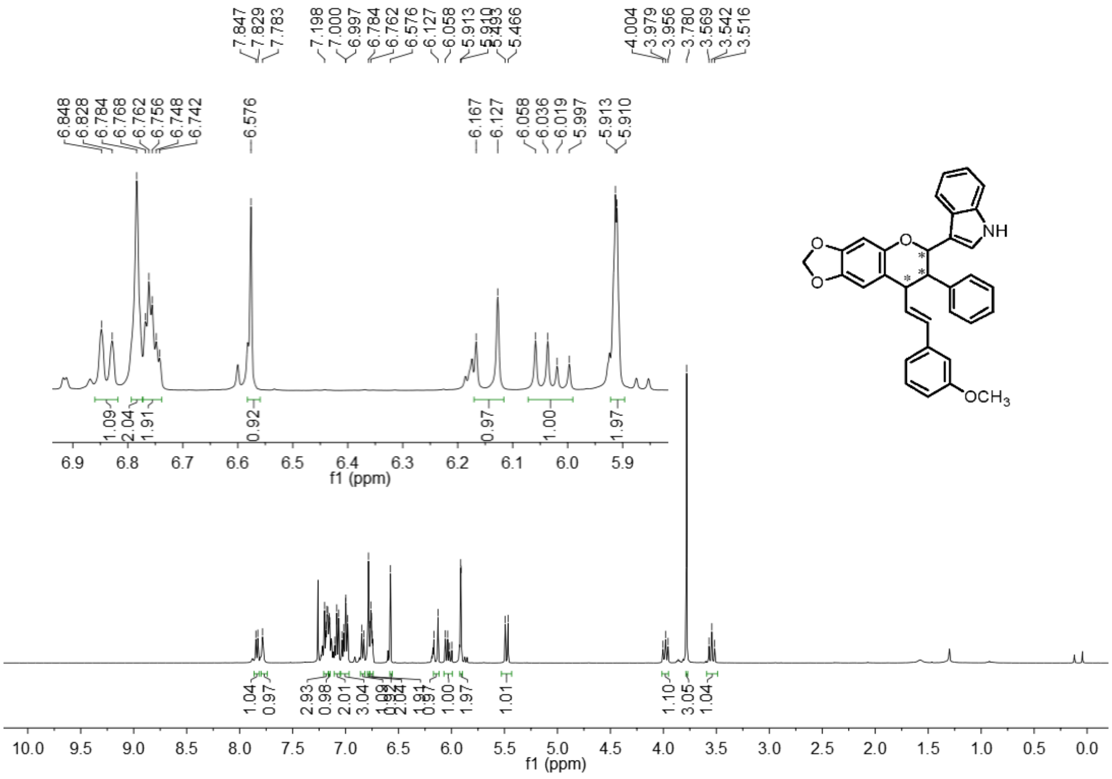

^{13}C NMR (100 MHz, $CDCl_3$) of compound **3ac**: (inseparable diastereomers, 86:14 dr)

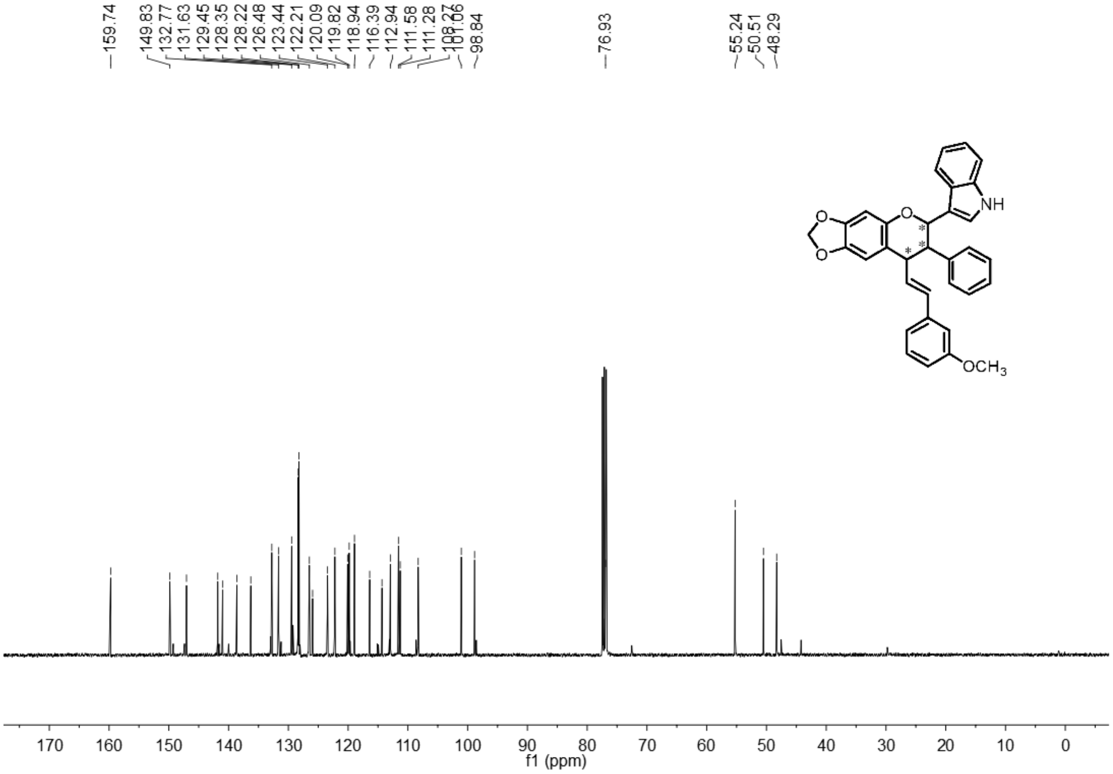

^{1}H NMR (400 MHz, $CDCl_3$) of compound **3ad**: (inseparable diastereomers, 85:15 dr)

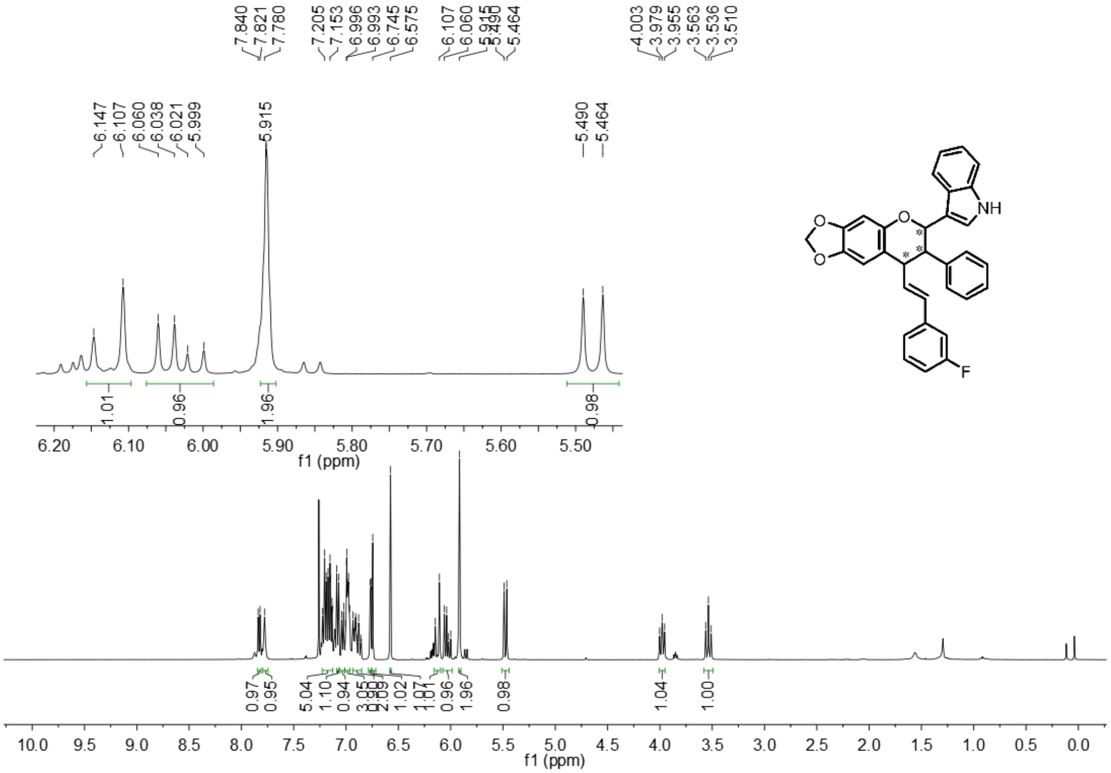

^{13}C NMR (100 MHz, $CDCl_3$) of compound **3ad**: (inseparable diastereomers, 85:15 dr)

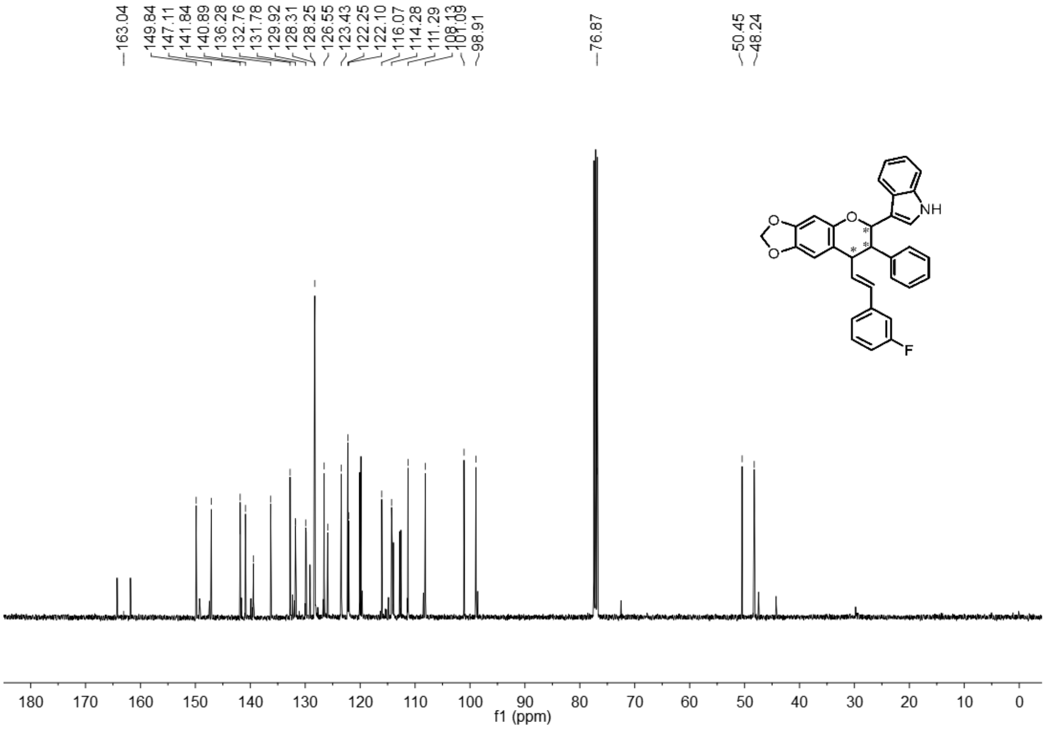

^{1}H NMR (400 MHz, acetone-d_6) of **3ae:** (inseparable diastereomers, 89:11 dr)

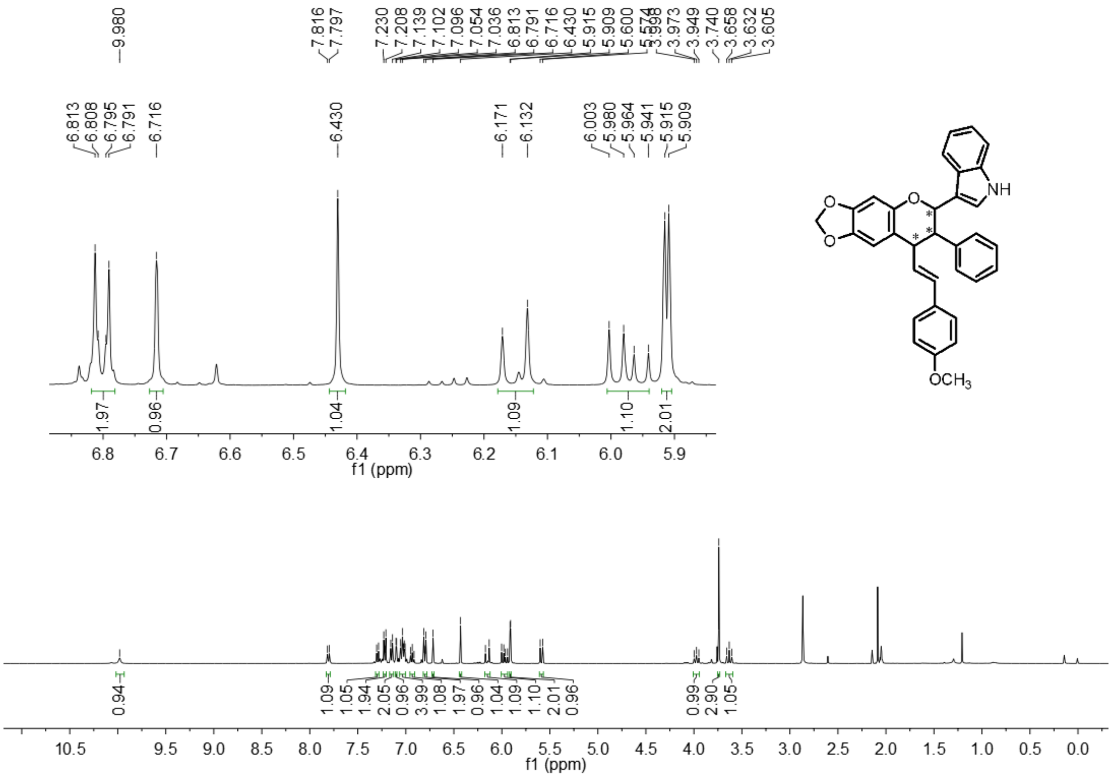

^{13}C NMR (100 MHz, acetone-d_6) of **3ae**: (inseparable diastereomers, 89:11 dr)

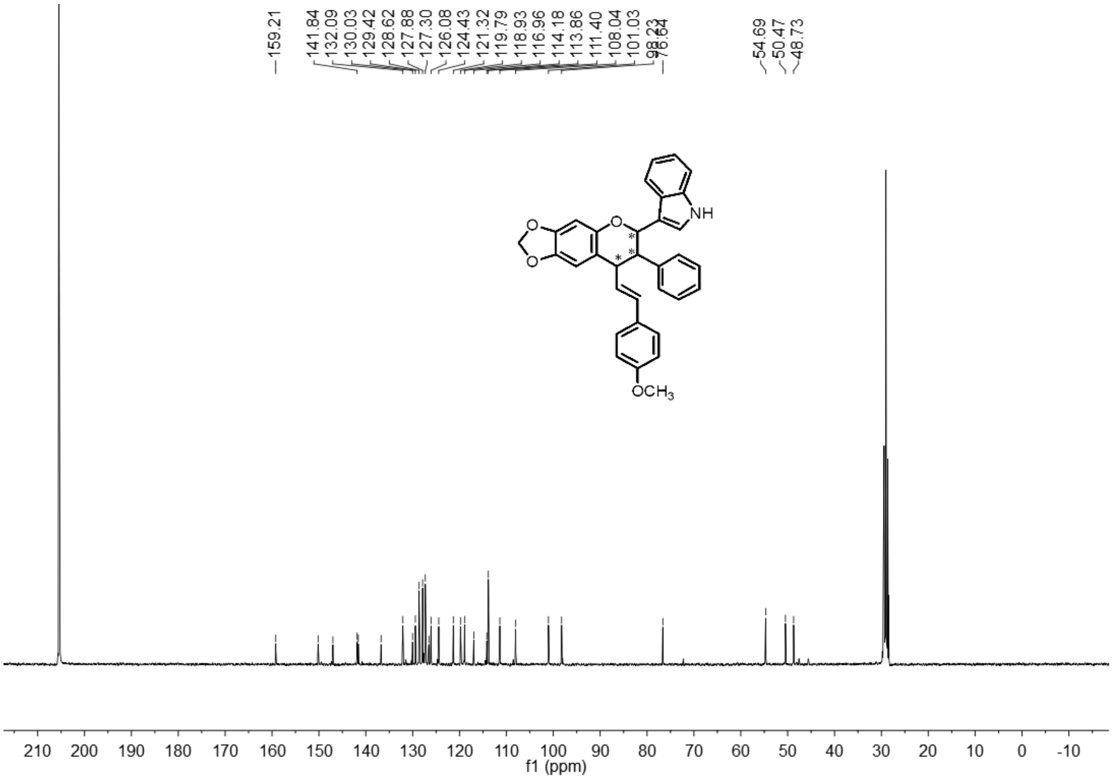

^{1}H NMR (400 MHz, $CDCl_3$) of compound **3af**: (inseparable diastereomers, 75:25 dr)

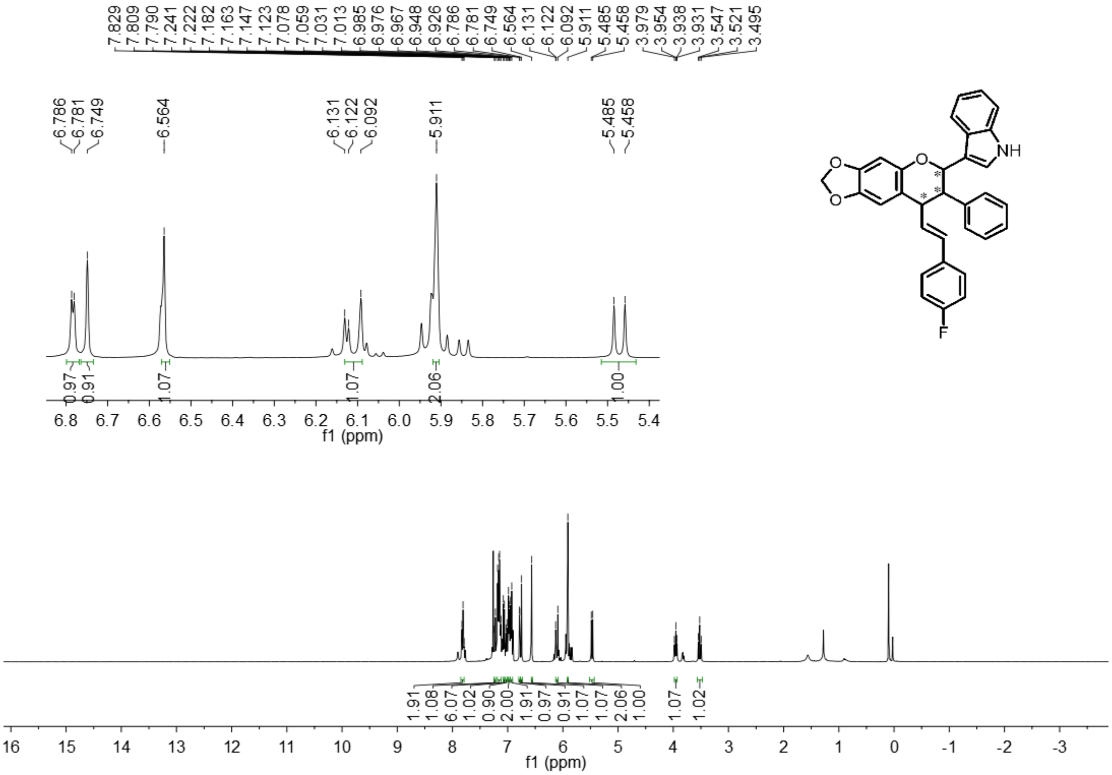

^{13}C NMR (100 MHz, $CDCl_3$) of compound **3af**: (inseparable diastereomers, 75:25 dr)

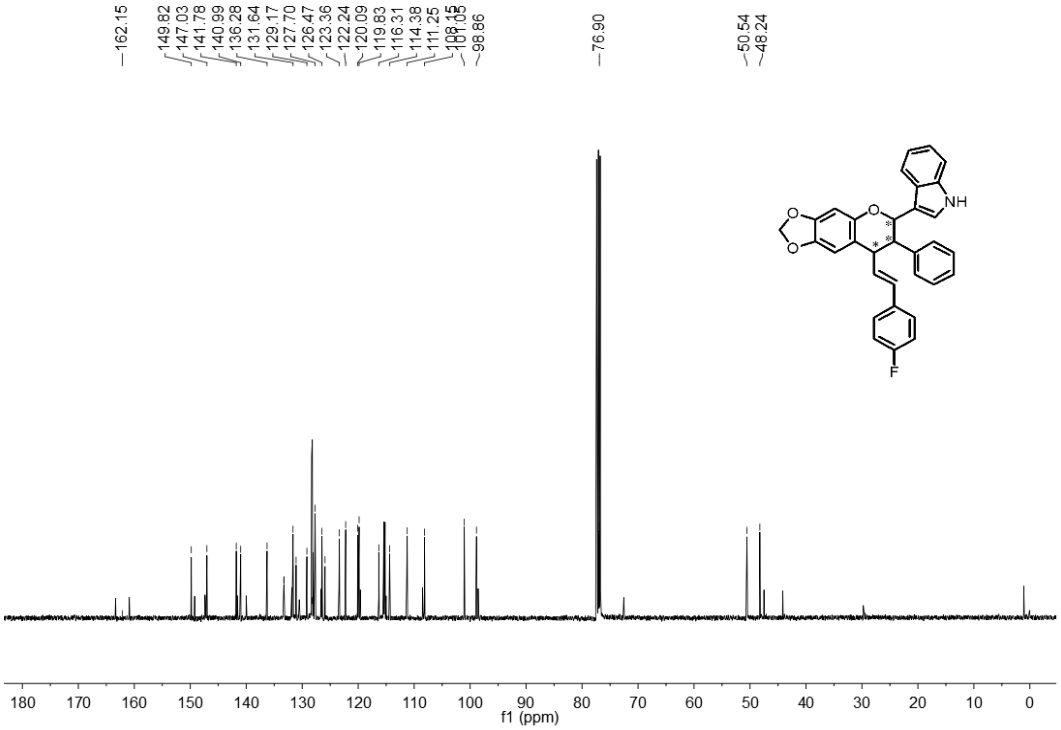

^{1}H NMR (400 MHz, $CDCl_3$) of compound **3ag**: (inseparable diastereomers, 84:16 dr)

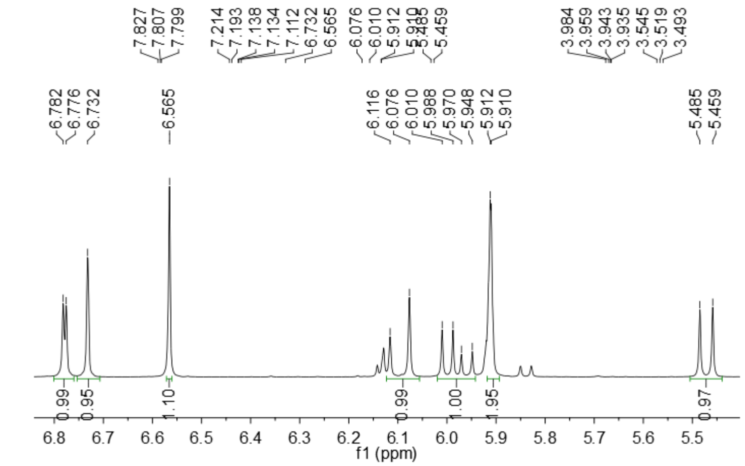

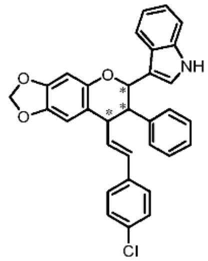

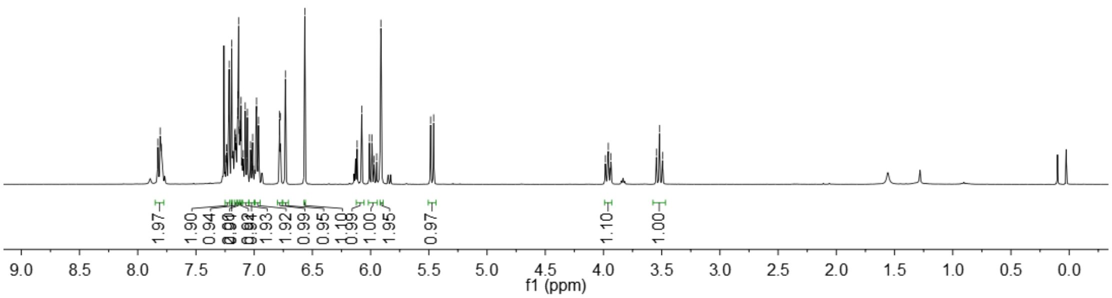

^{13}C NMR (100 MHz, $CDCl_3$) of compound **3ag**: (inseparable diastereomers, 84:16 dr)

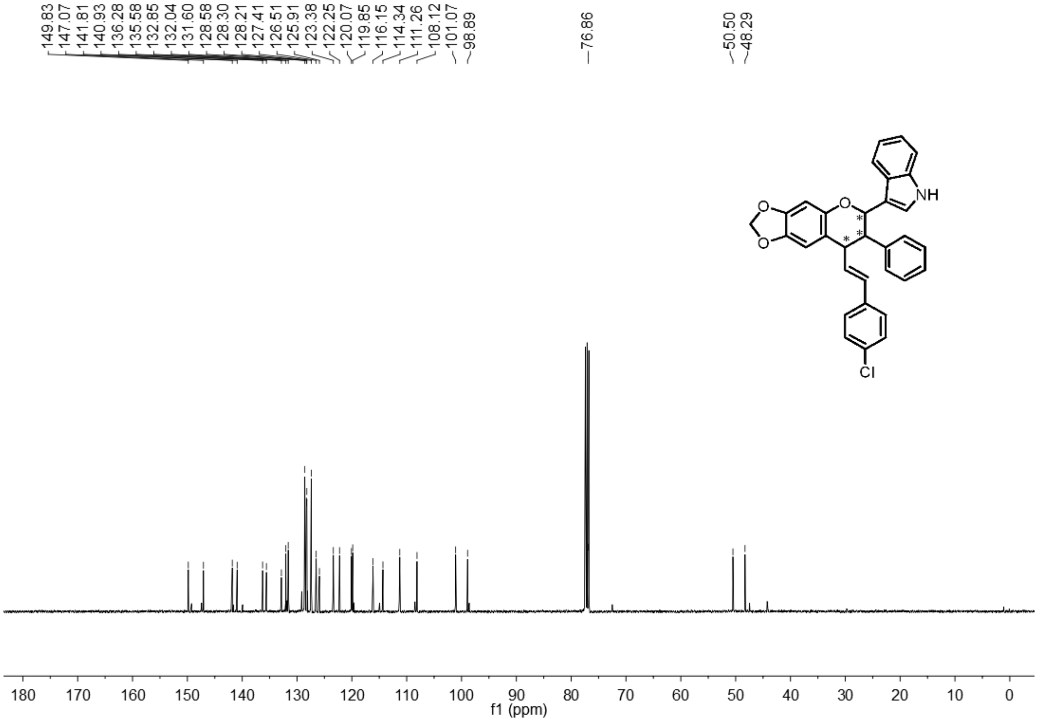

^{1}H NMR (400 MHz, $CDCl_3$) of compound **3ah**: (inseparable diastereomers, 85:15 dr)

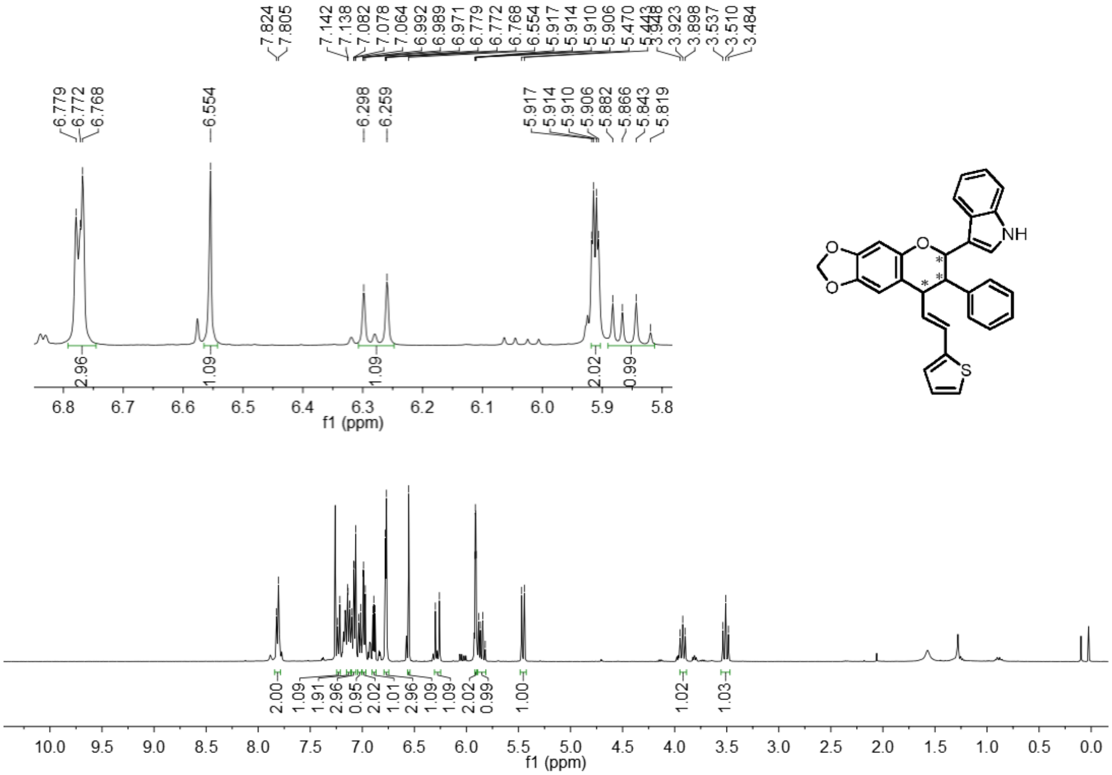

^{13}C NMR (100 MHz, $CDCl_3$) of compound **3ah**: (inseparable diastereomers, 85:15 dr)

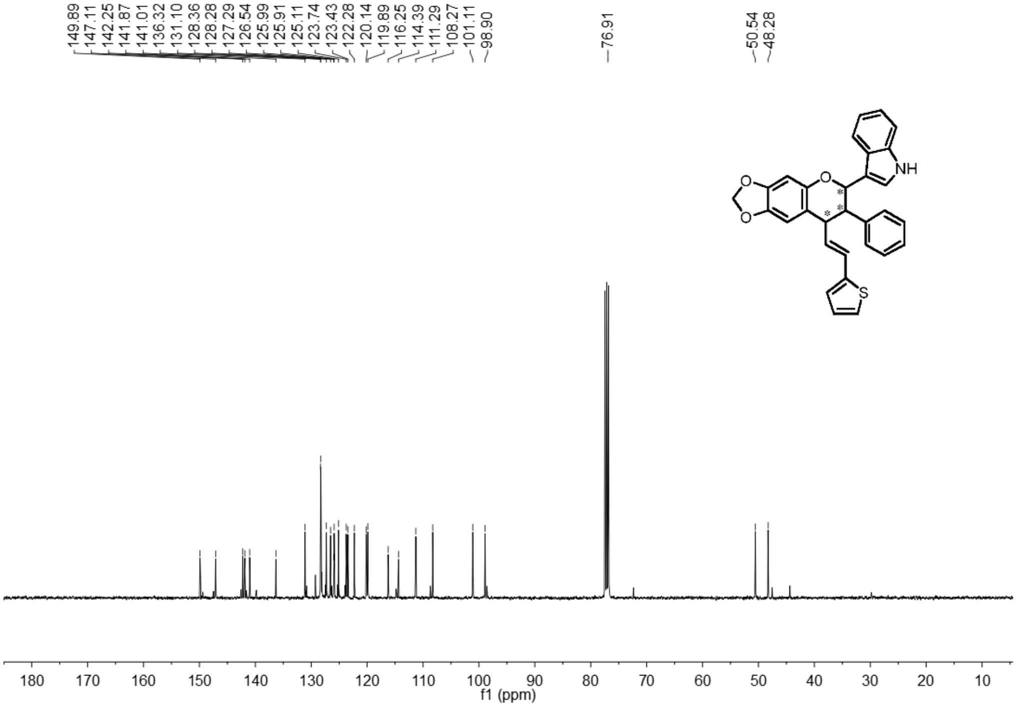

2. NMR spectra of substrates 6

^{1}H NMR (400 MHz, $CDCl_3$) of compound **6aa**:

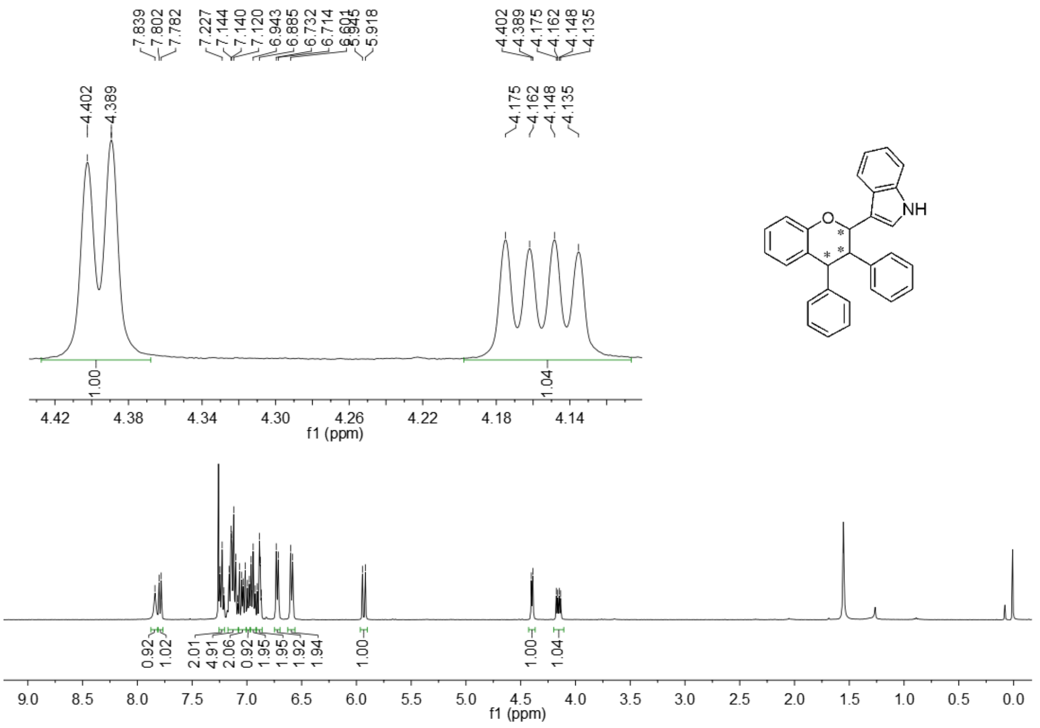

^{13}C NMR (100 MHz, $CDCl_3$) of compound **6aa**:

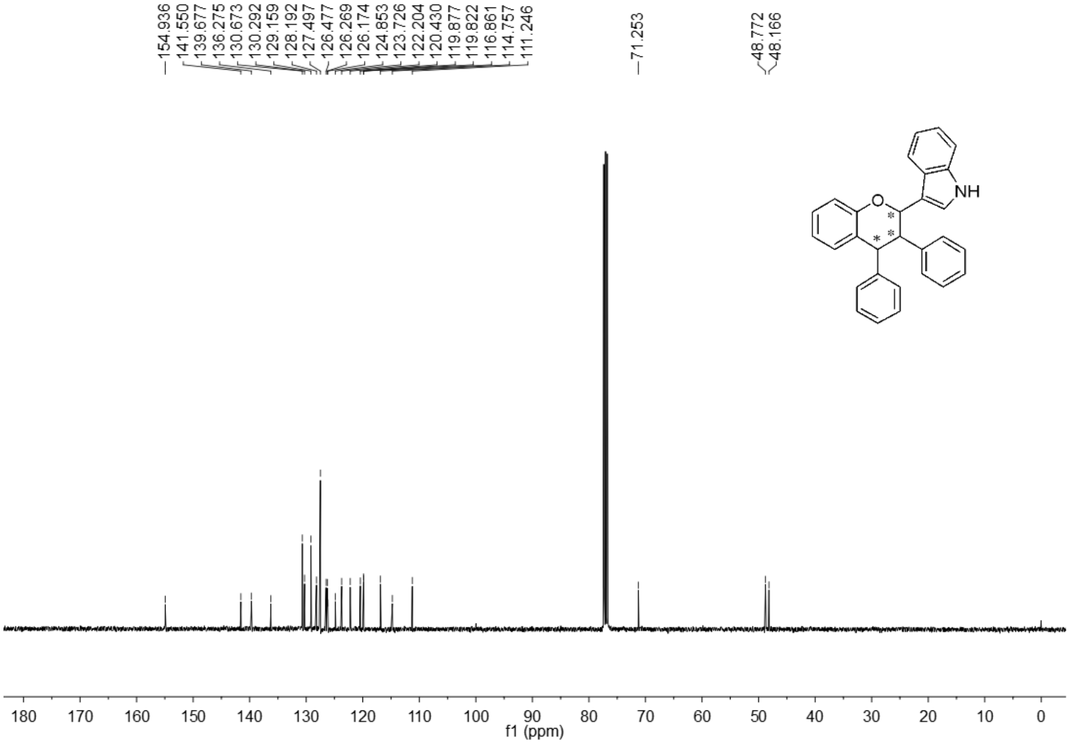

^1H NMR (400 MHz, $CDCl_3$) of compound **6ba**:

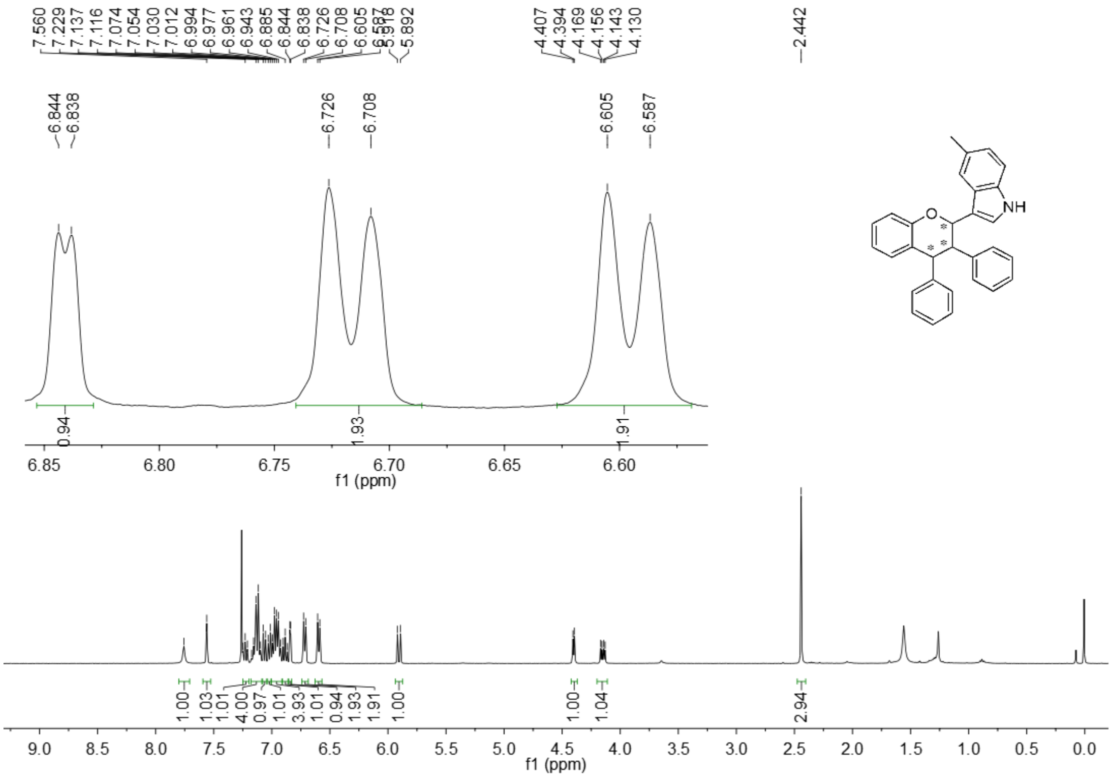

^{13}C NMR (100 MHz, $CDCl_3$) of compound **6ba**:

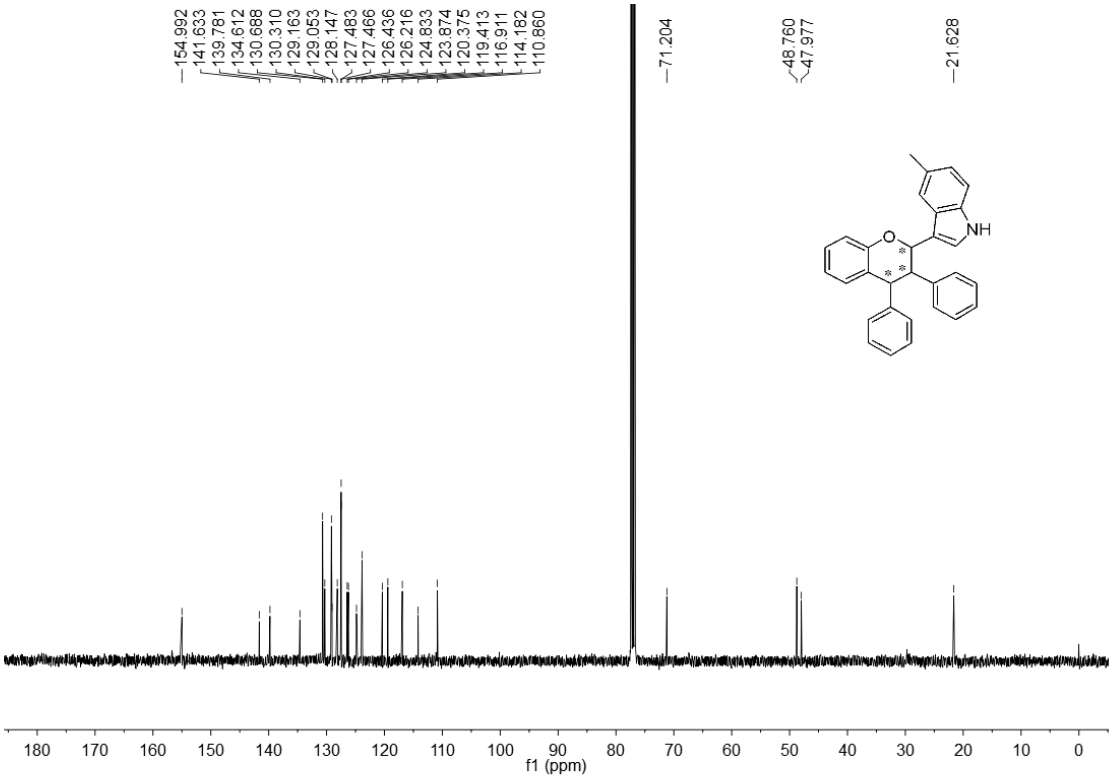

^{1}H NMR (400 MHz, $CDCl_3$) of compound **6pa**:

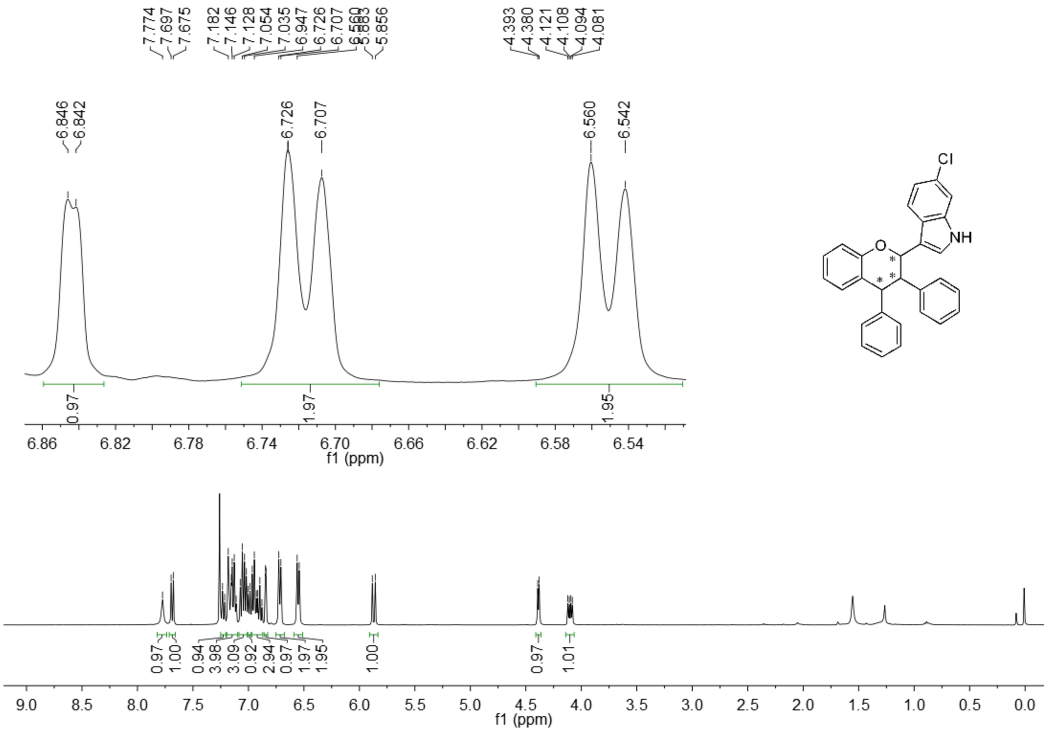

^{13}C NMR (100 MHz, $CDCl_3$) of compound **6pa**:

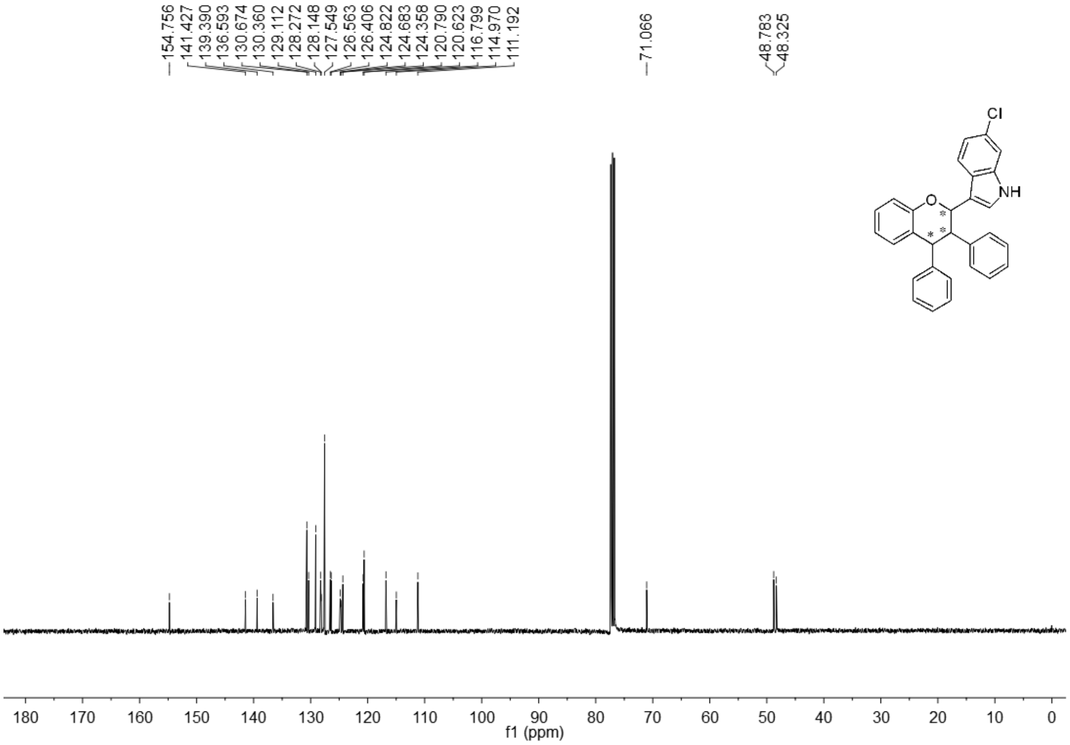

^{1}H NMR (400 MHz, $CDCl_3$) of compound **6da**:

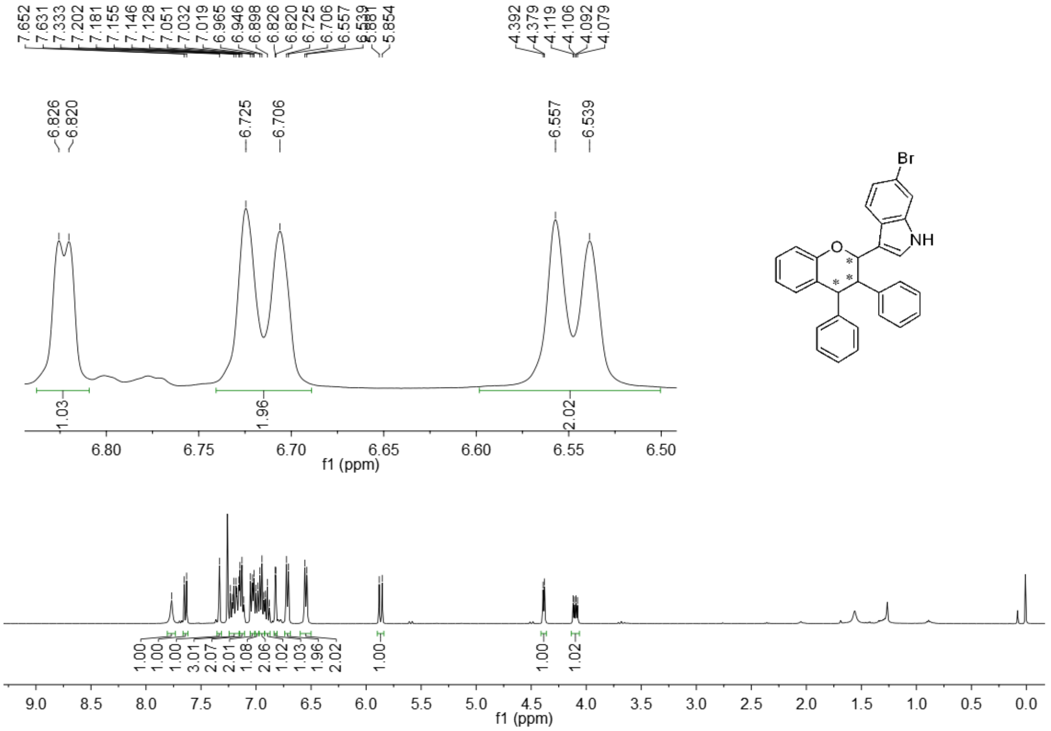

^{13}C NMR (100 MHz, $CDCl_3$) of compound **6da**:

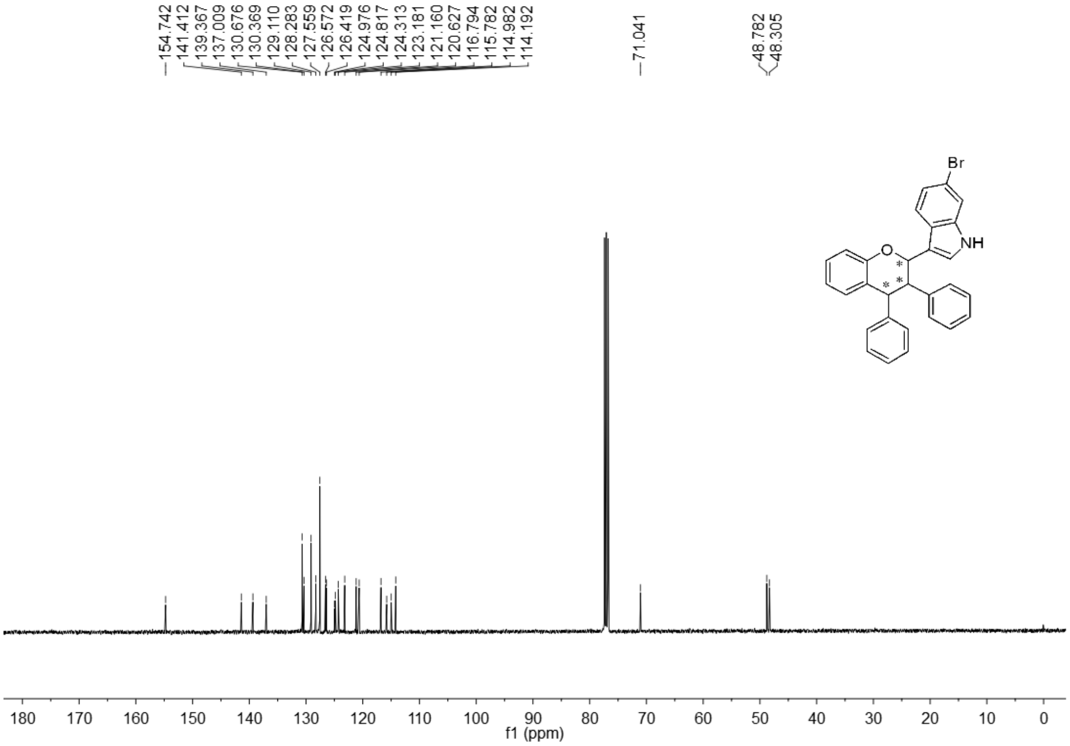

^{1}H NMR (400 MHz, $CDCl_3$) of compound **6ea**:

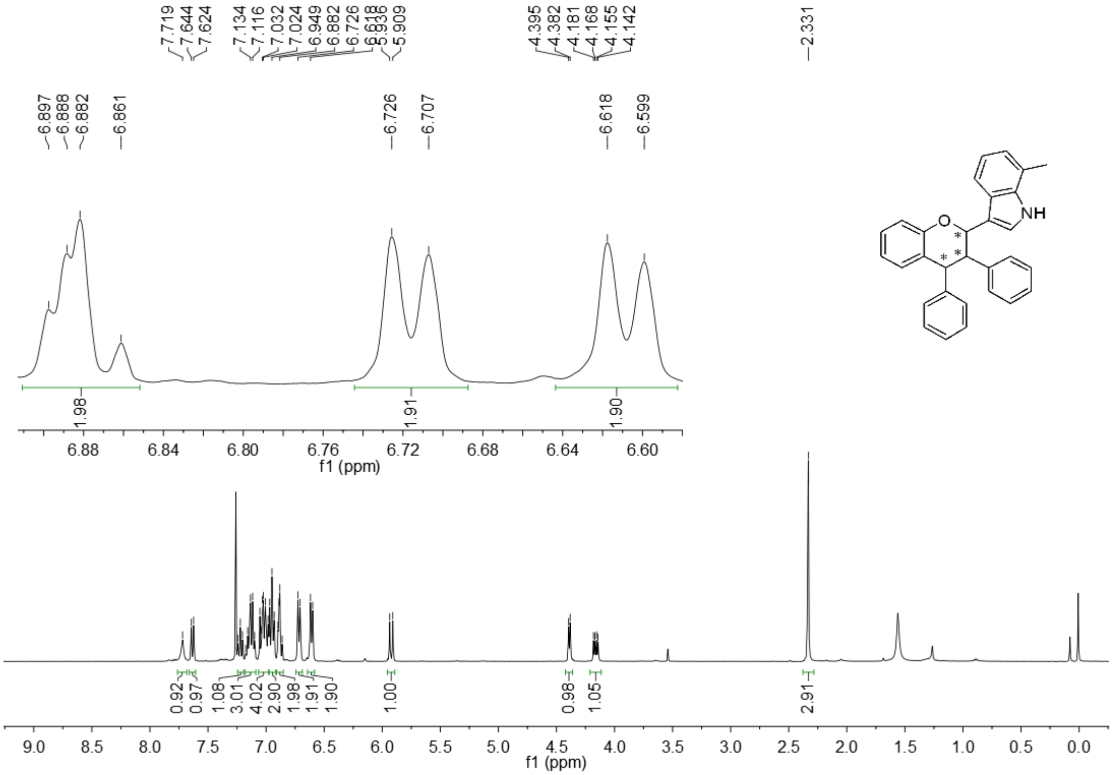

^{13}C NMR (100 MHz, $CDCl_3$) of compound **6ea**:

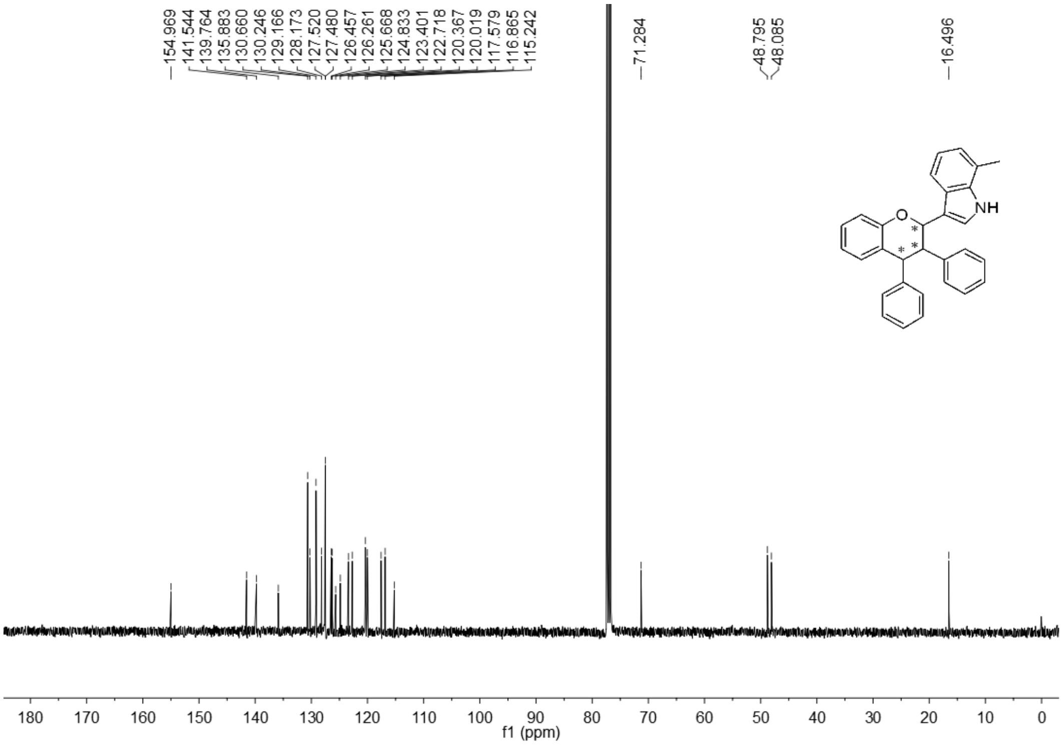

^{1}H NMR (400 MHz, $CDCl_3$) of compound **6qa**:

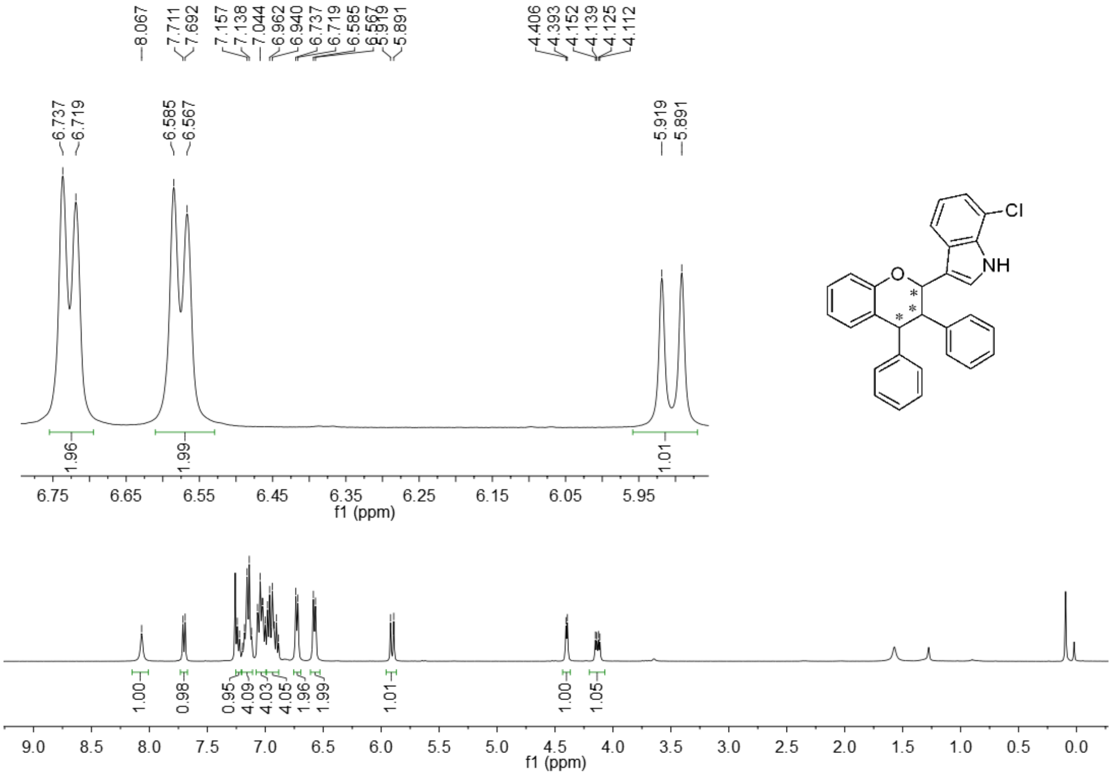

^{13}C NMR (100 MHz, $CDCl_3$) of compound **6qa**:

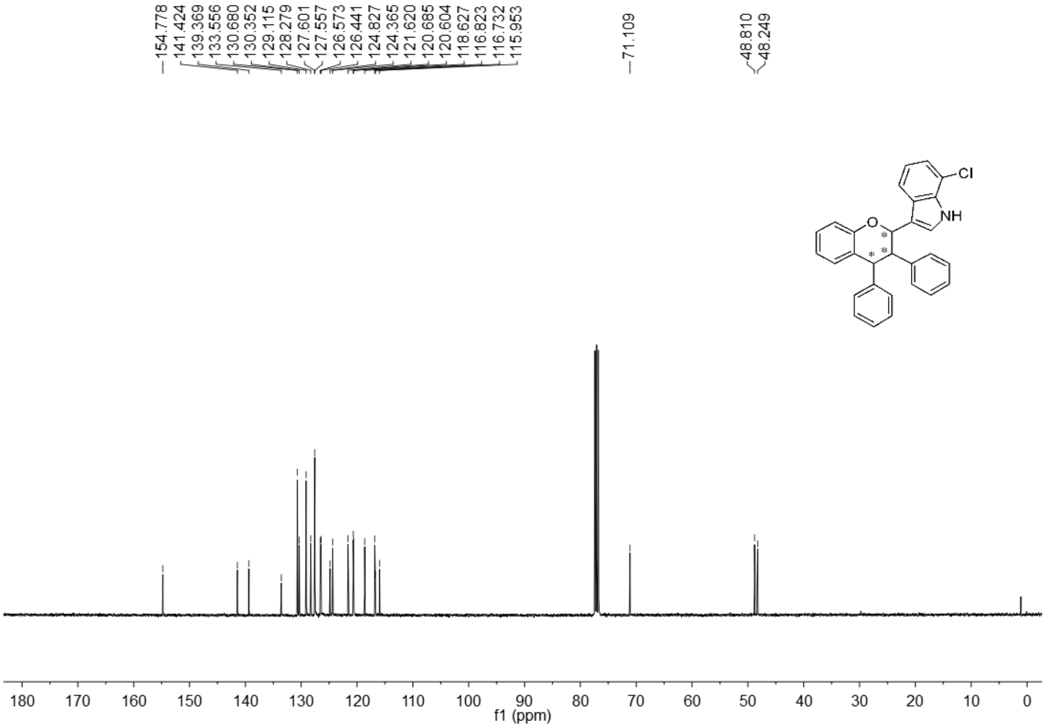

^{1}H NMR (400 MHz, $CDCl_3$) of compound **6ra**:

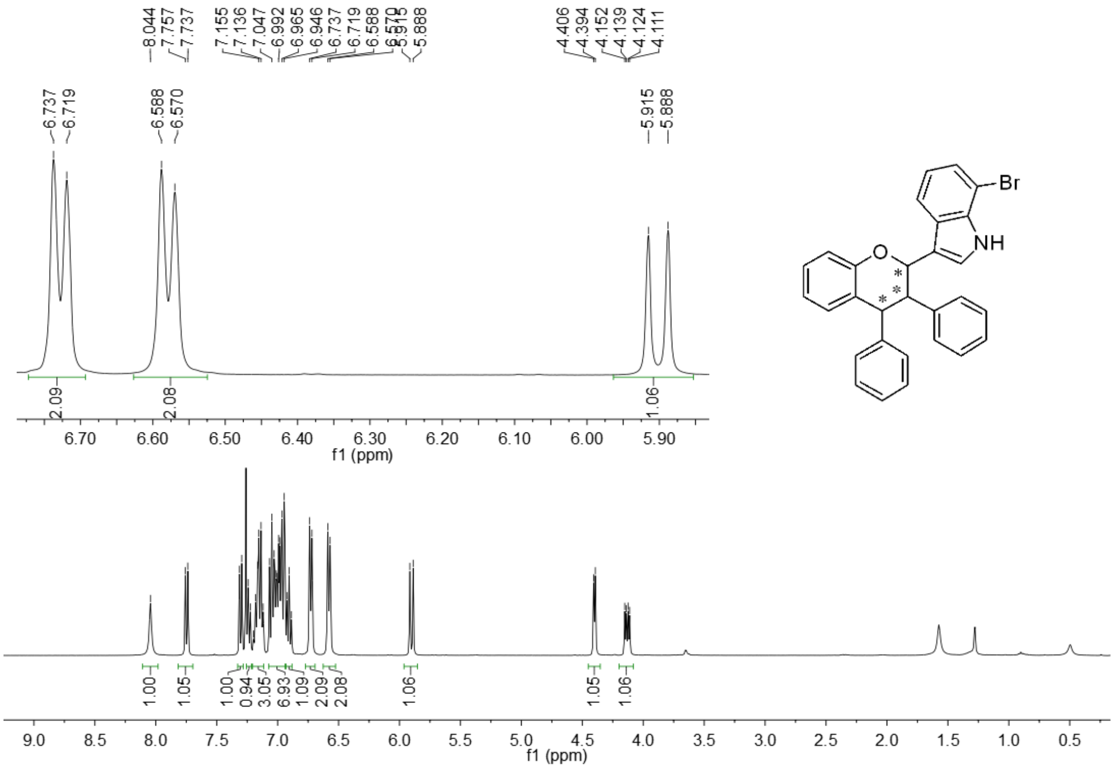

^{13}C NMR (100 MHz, $CDCl_3$) of compound **6ra**:

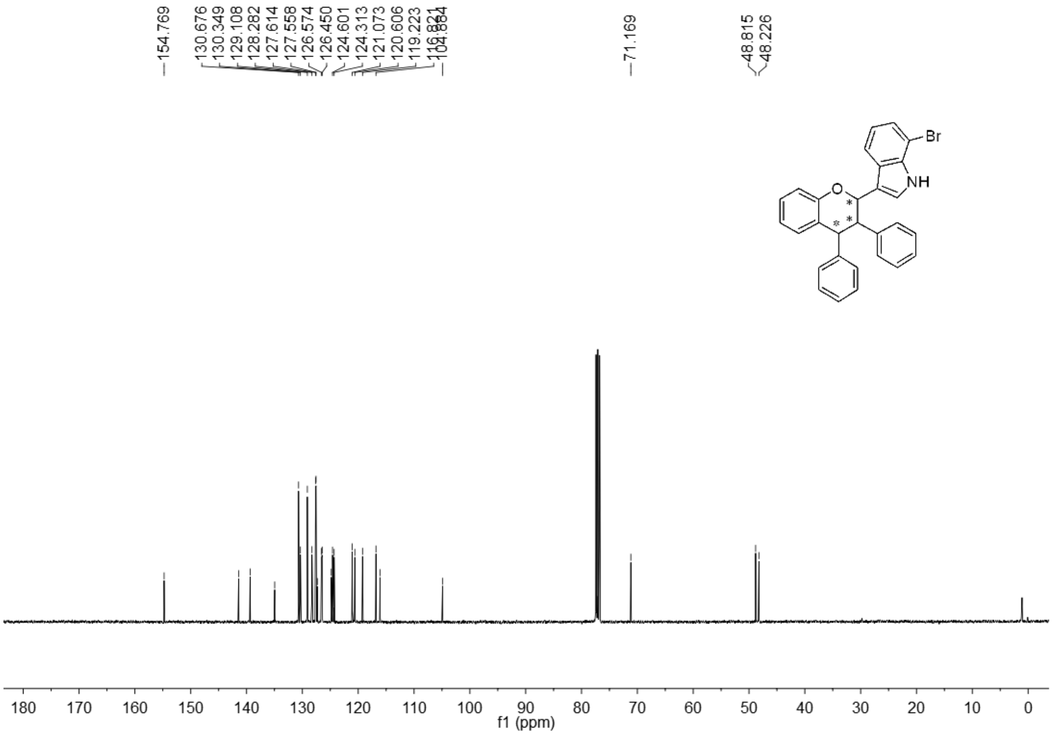

^{1}H NMR (400 MHz, $CDCl_3$) of compound **6ka**:

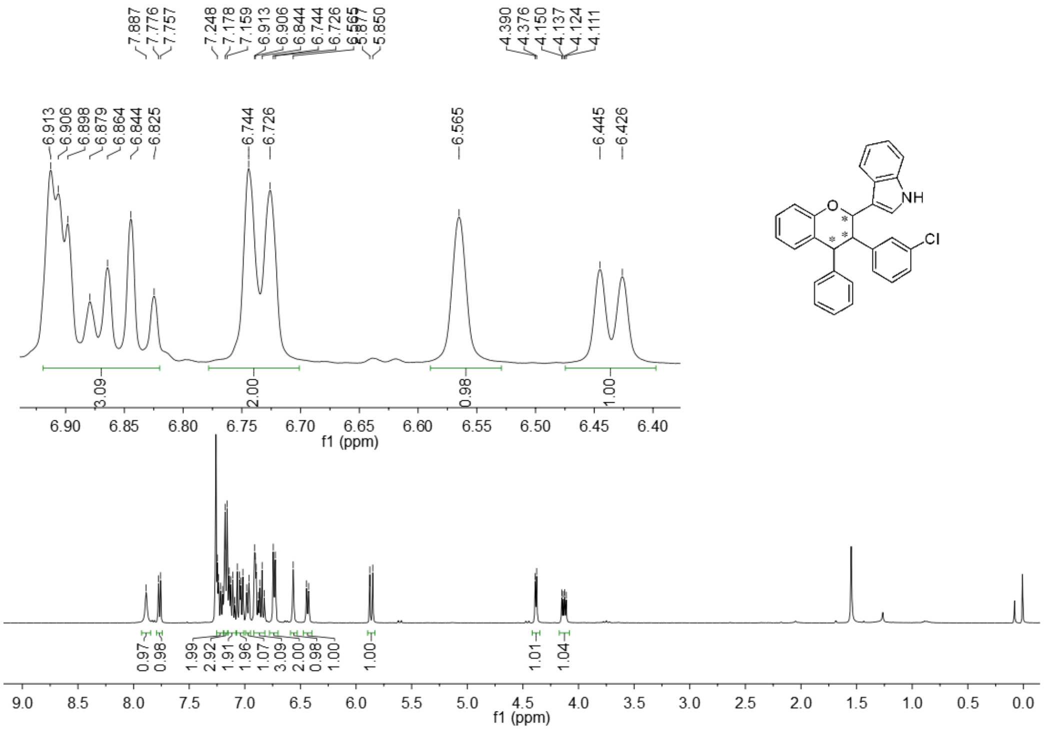

^{13}C NMR (100 MHz, $CDCl_3$) of compound **6ka**:

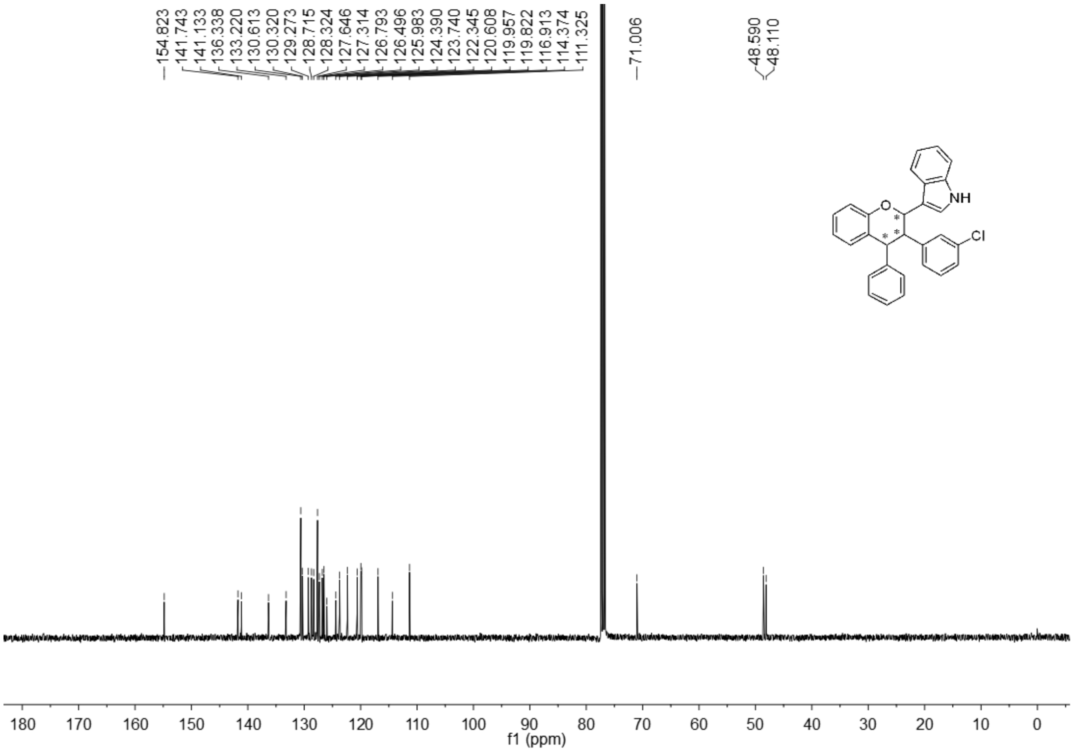

^{1}H NMR (400 MHz, $CDCl_3$) of compound **6ma**:

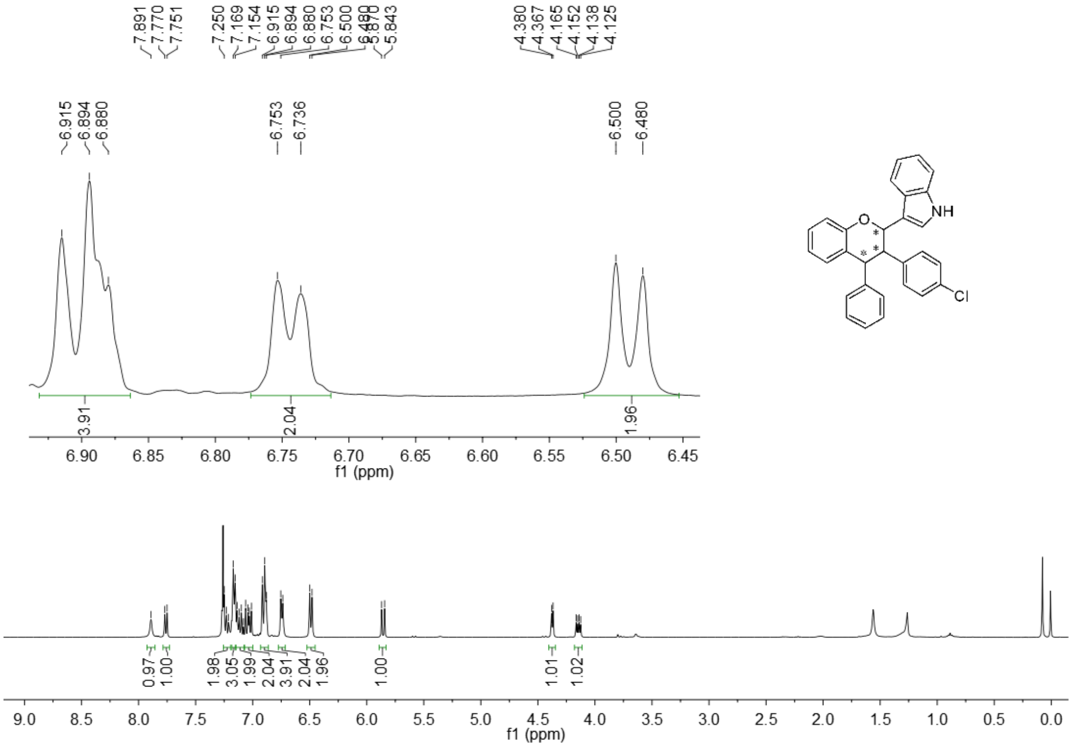

^{13}C NMR (100 MHz, $CDCl_3$) of compound **6ma**:

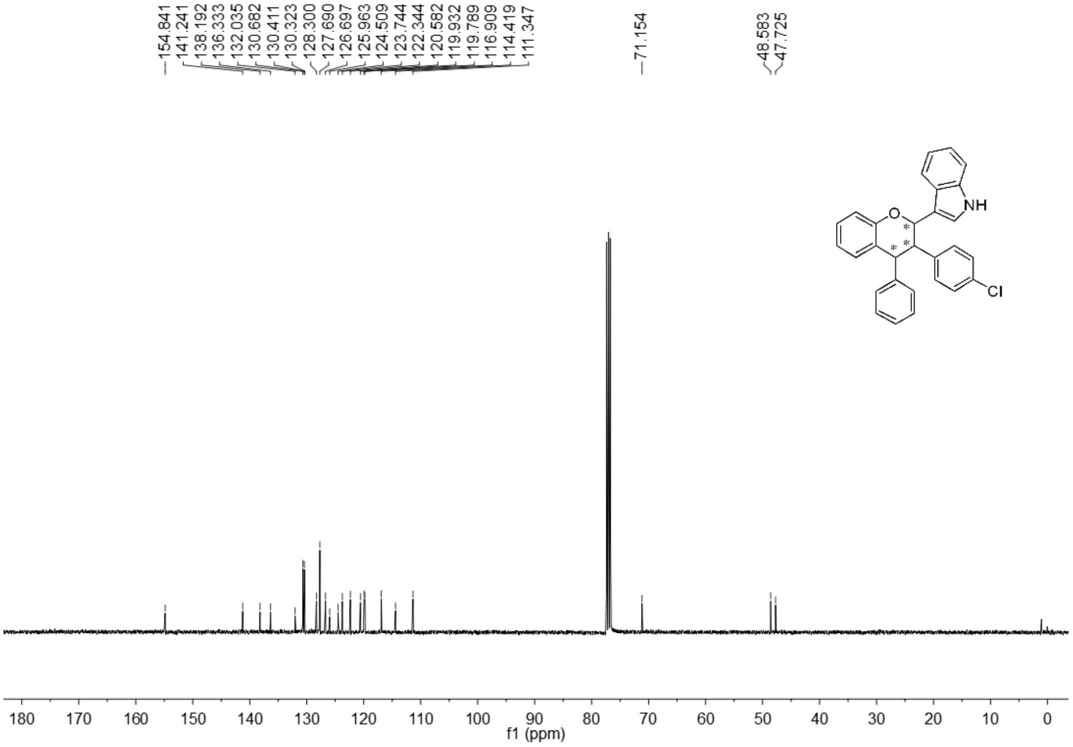

^{1}H NMR (400 MHz, $CDCl_3$) of compound **6ab**:

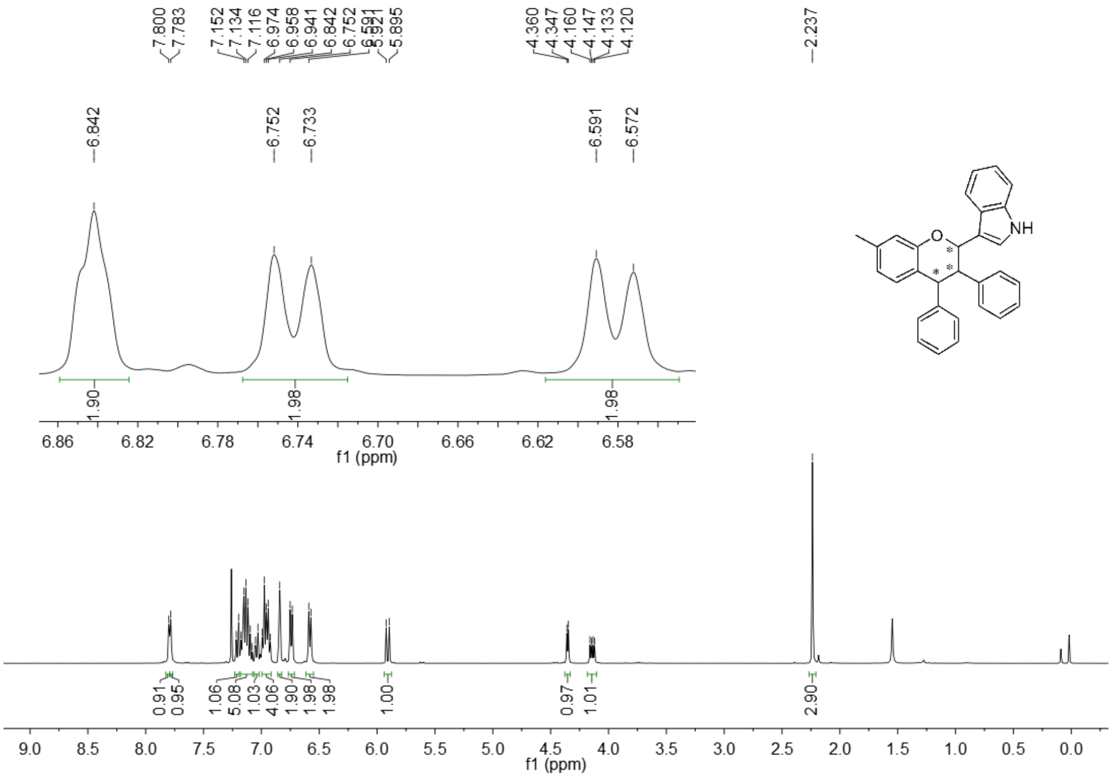

^{13}C NMR (100 MHz, $CDCl_3$) of compound **6ab**:

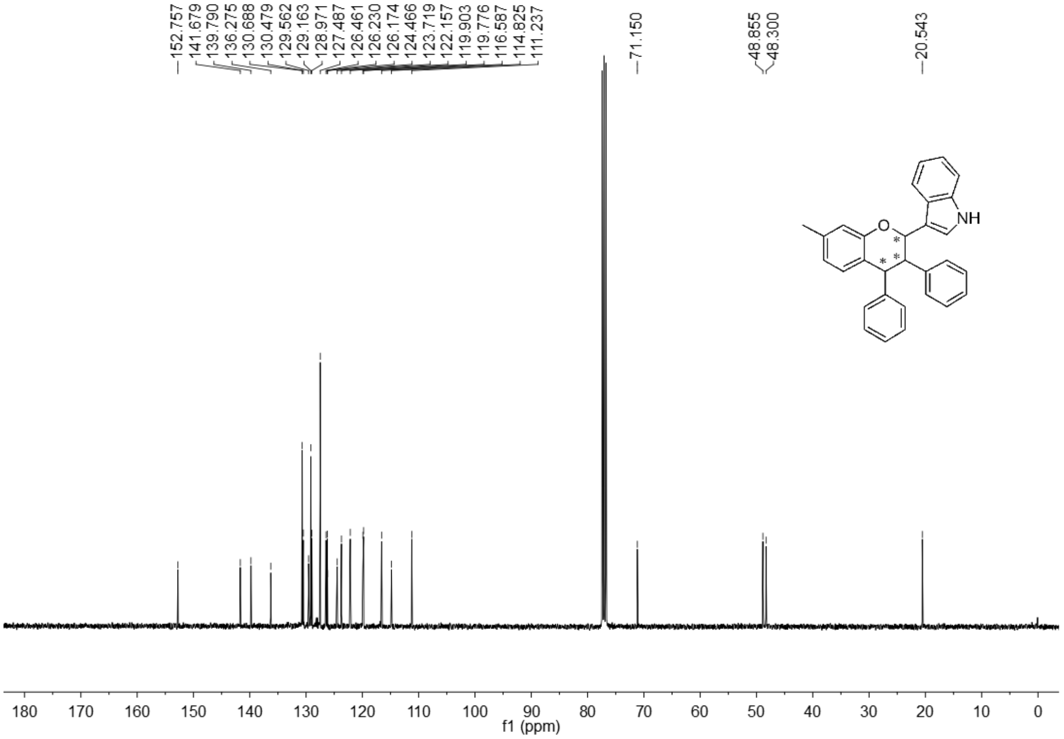

^{1}H NMR (400 MHz, $CDCl_3$) of compound **6ac** (major diastereoisomer):

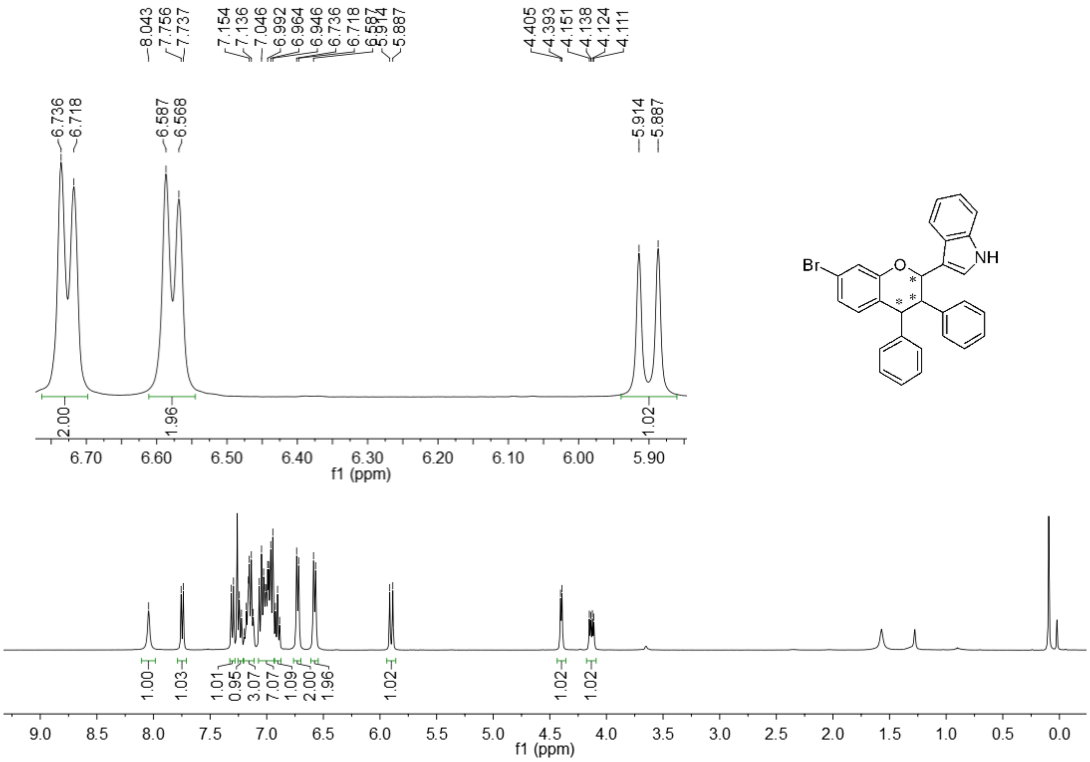

^{13}C NMR (100 MHz, $CDCl_3$) of compound **6ac** (major diastereoisomer):

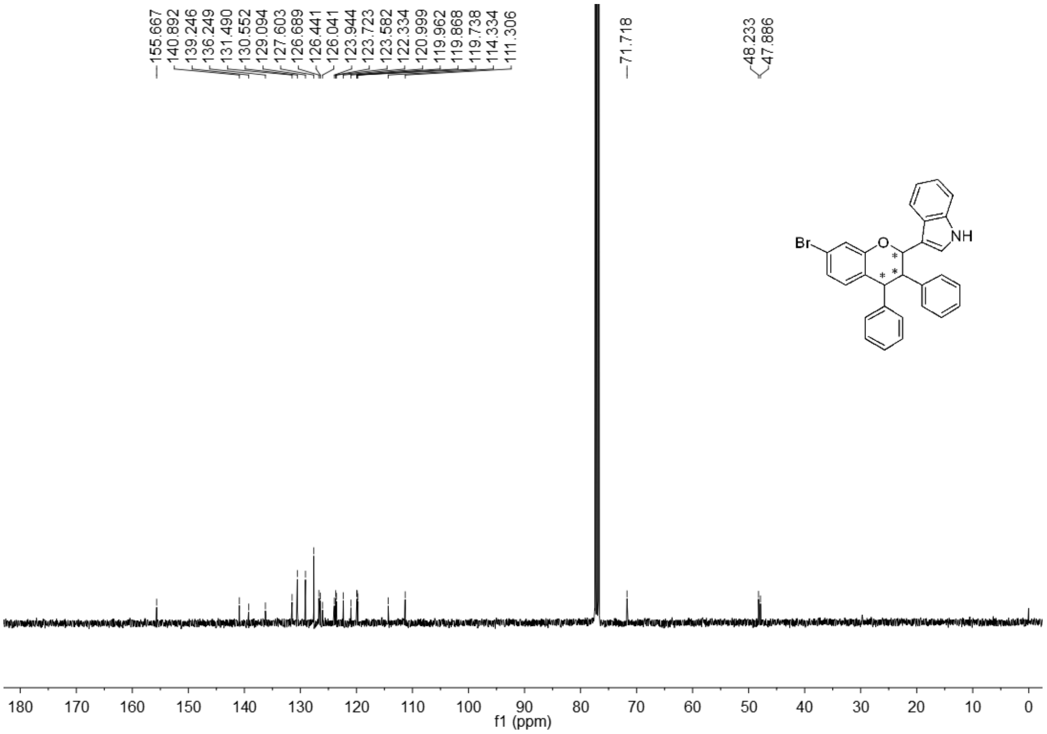

^{1}H NMR (400 MHz, $CDCl_3$) of compound **6ac** (minor diastereoisomer):

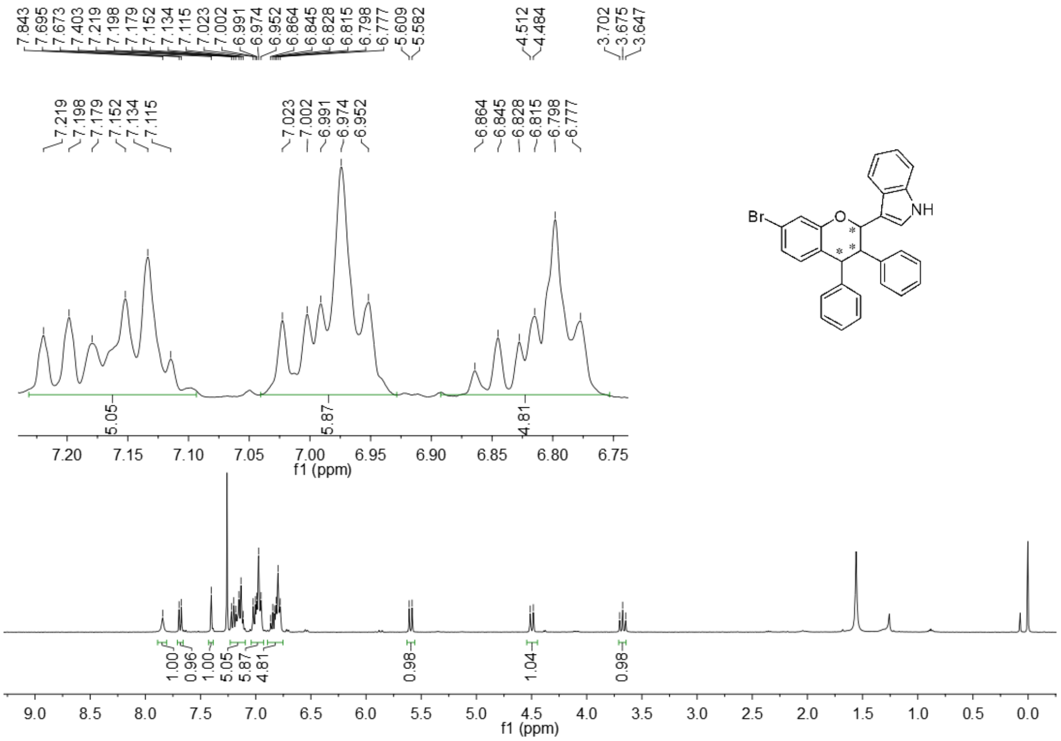

^{13}C NMR (100 MHz, $CDCl_3$) of compound **6ac** (minor diastereoisomer):

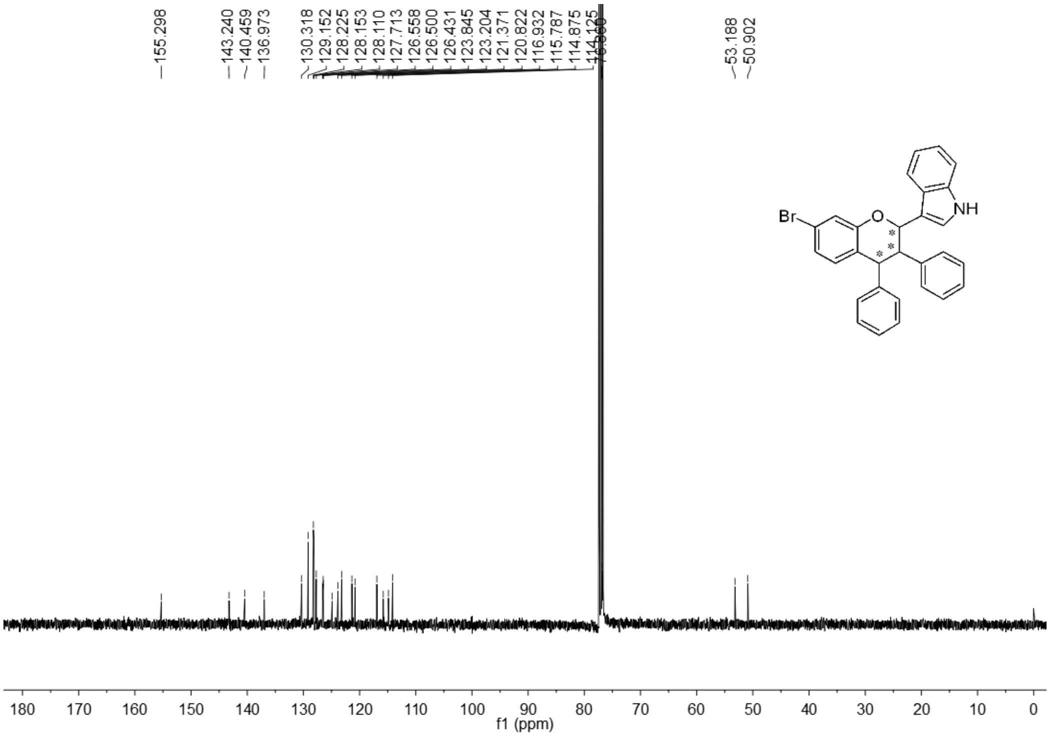

^{1}H NMR (400 MHz, $CDCl_3$) of compound **6ad**:

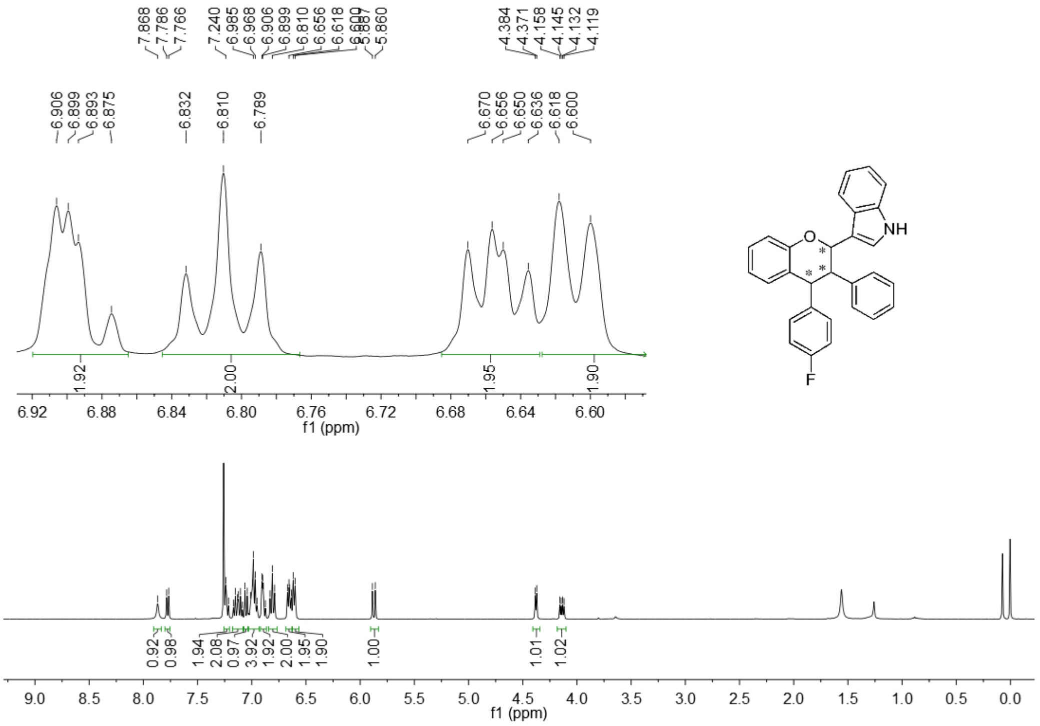

^{13}C NMR (100 MHz, $CDCl_3$) of compound **6ad**:

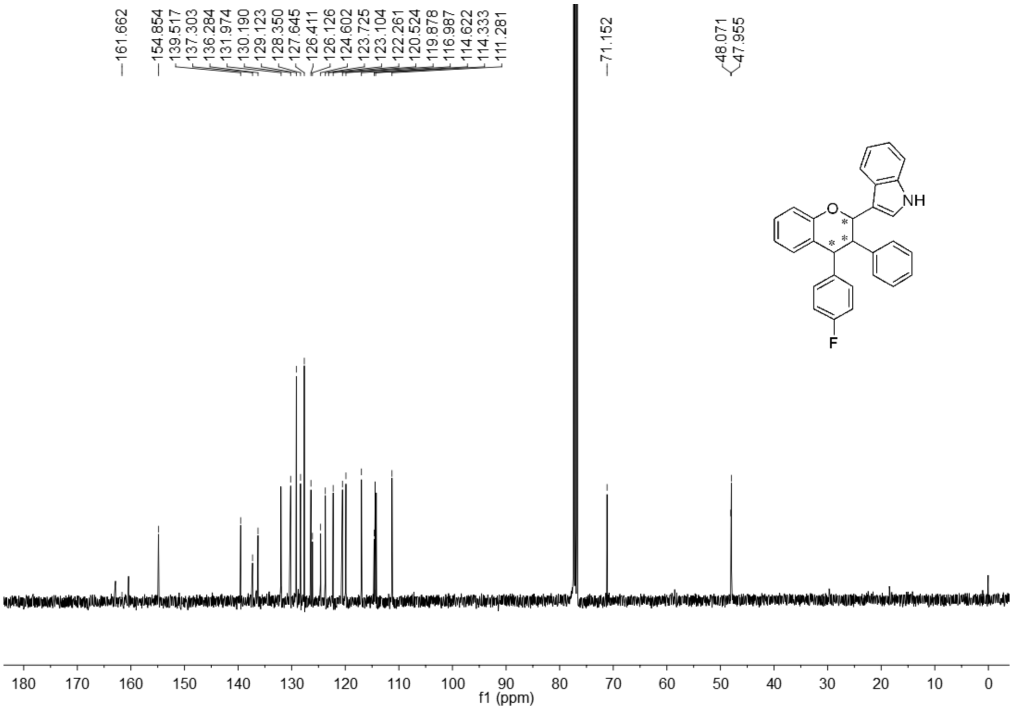

^{19}F NMR (376 MHz, $CDCl_3$) of compound **6ad**:

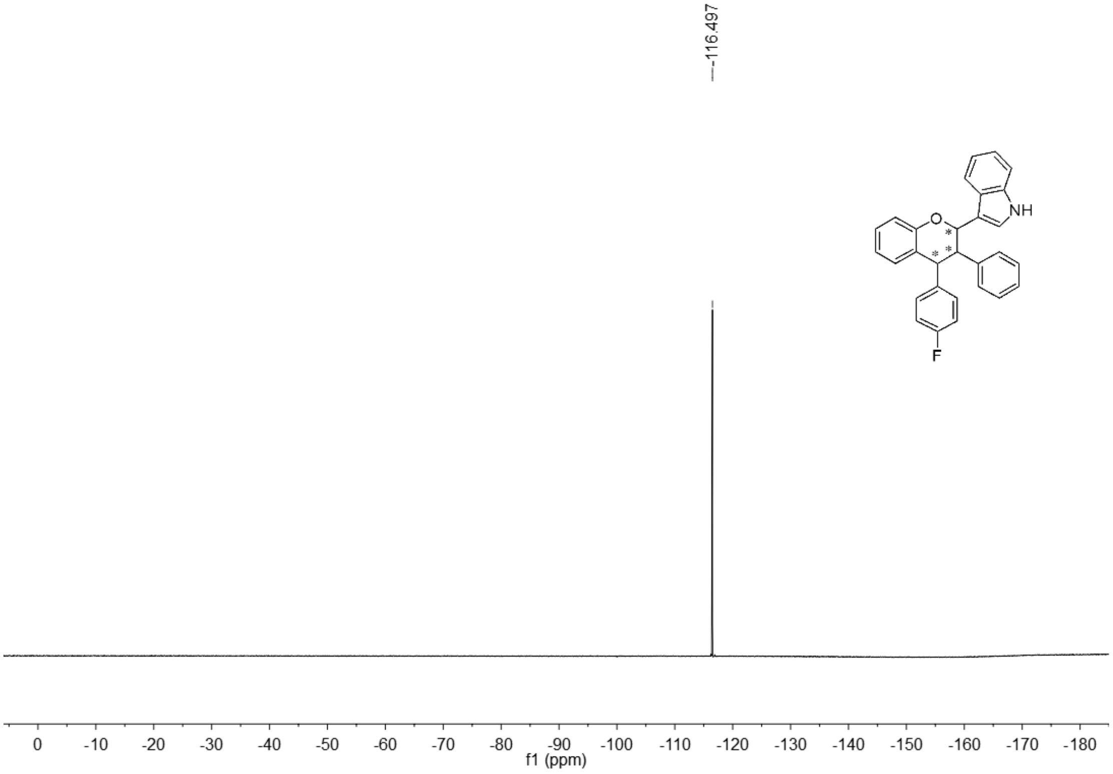

^{1}H NMR (400 MHz, $CDCl_3$) of compound **6ae**:

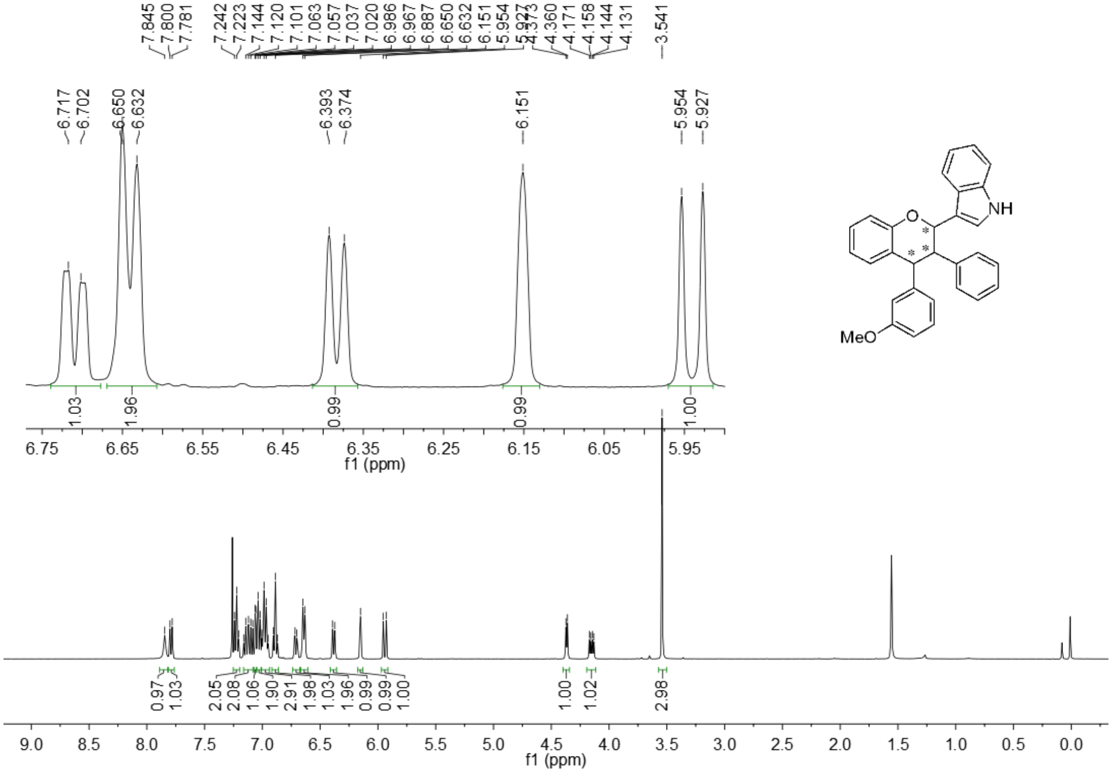

^{13}C NMR (100 MHz, $CDCl_3$) of compound **6ae**:

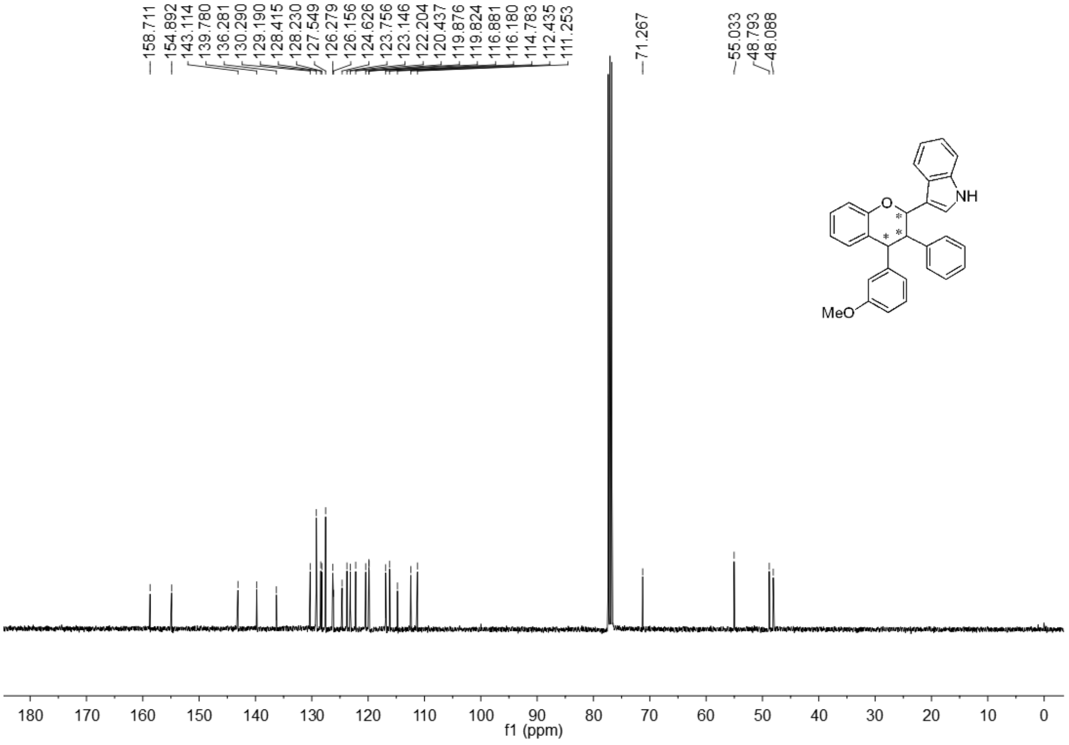

^{1}H NMR (400 MHz, $CDCl_3$) of compound **6af**:

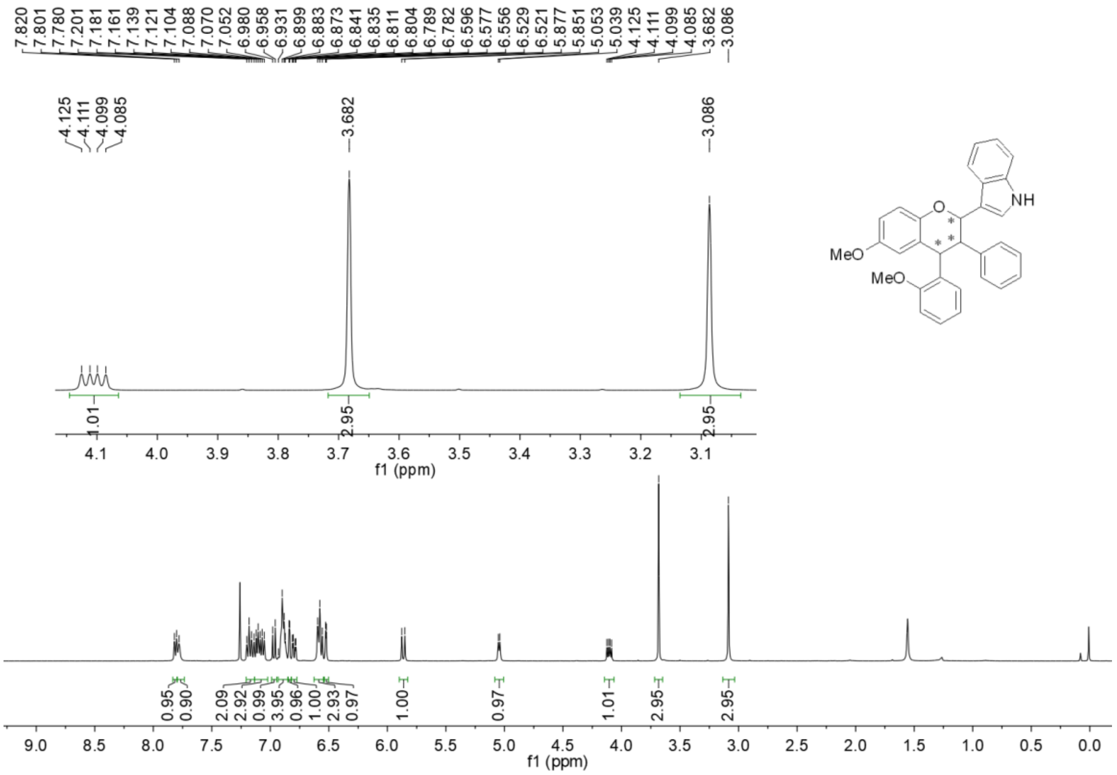

^{13}C NMR (100 MHz, $CDCl_3$) of compound **6af**:

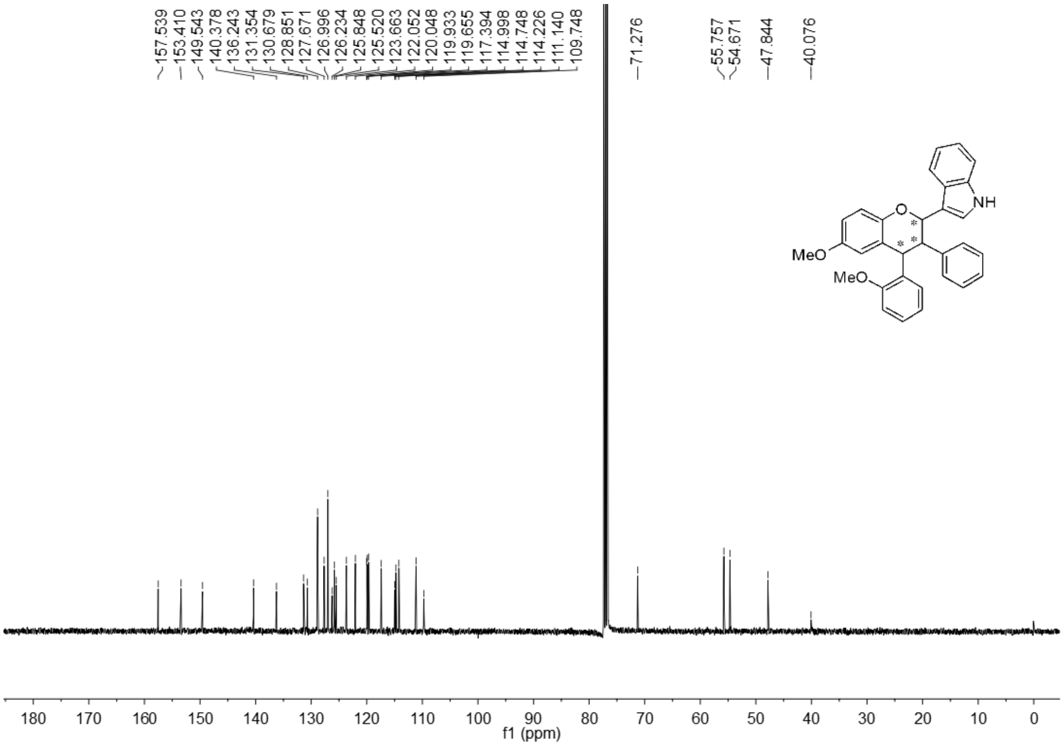

3. HPLC spectra of products 3

3aa: (inseparable diastereomers, 89:11 dr):

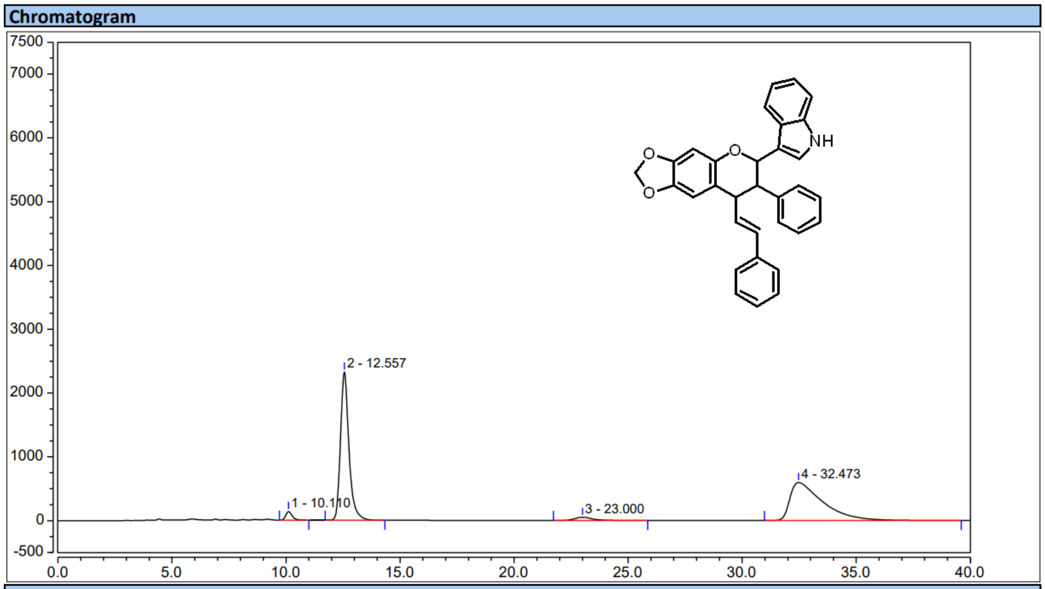

Integration Results

No.	Peak Name	Retention Time min	Area mAU*min	Height mAU	Relative Area %	Relative Height %	Amount n.a.
1		10.110	46.305	138.093	2.22	4.44	n.a.
2		12.557	1002.112	2326.762	48.04	74.79	n.a.
3		23.000	45.881	50.188	2.20	1.61	n.a.
4		32.473	991.908	595.879	47.55	19.15	n.a.
Total:			2086.205	3110.921	100.00	100.00	

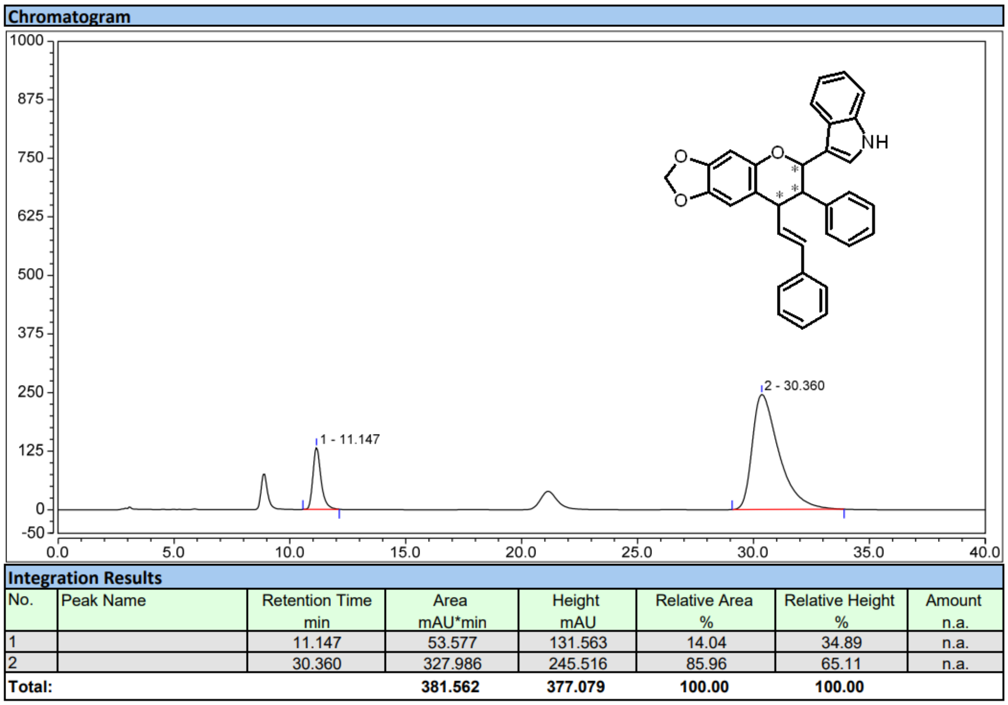

Integration Results

No.	Peak Name	Retention Time min	Area mAU*min	Height mAU	Relative Area %	Relative Height %	Amount n.a.
1		11.147	53.577	131.563	14.04	34.89	n.a.
2		30.360	327.986	245.516	85.96	65.11	n.a.
Total:			381.562	377.079	100.00	100.00	

3ba: (inseparable diastereomers, 83:17 dr):

Chromatogram

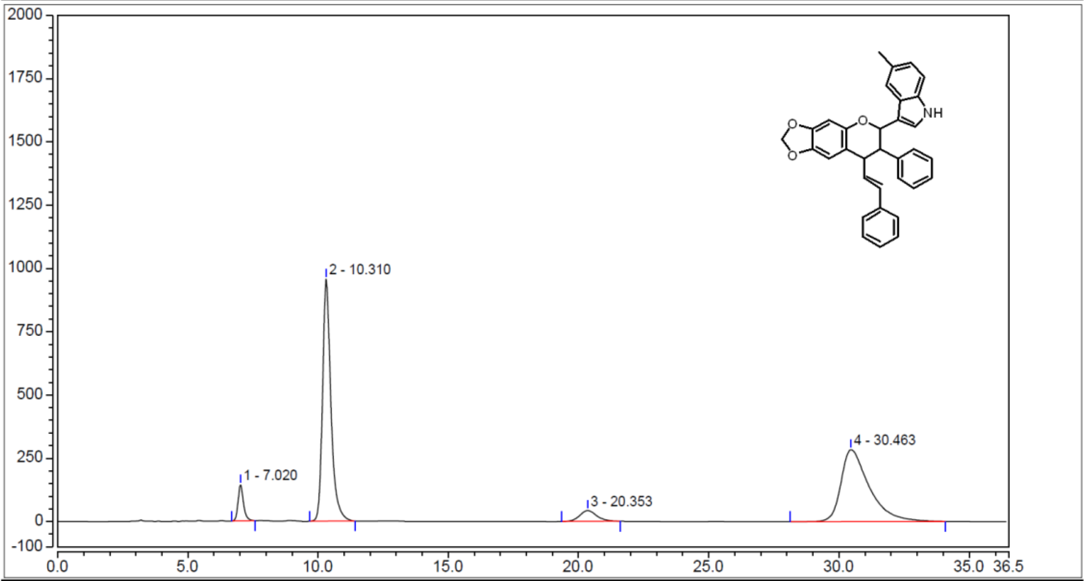

Integration Results

No.	Peak Name	Retention Time min	Area mAU*min	Height mAU	Relative Area %	Relative Height %	Amount n.a.
1		7.020	34.175	142.608	4.39	10.00	n.a.
2		10.310	355.870	956.604	45.71	67.10	n.a.
3		20.353	32.584	42.807	4.19	3.00	n.a.
4		30.463	355.941	283.635	45.72	19.90	n.a.
**Total:**			**778.569**	**1425.654**	**100.00**	**100.00**	

Chromatogram

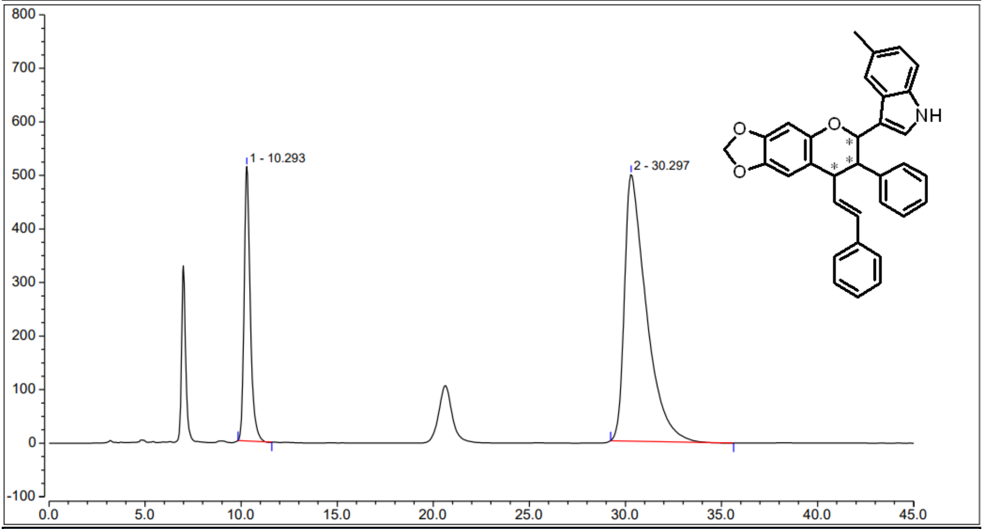

Integration Results

No.	Peak Name	Retention Time min	Area mAU*min	Height mAU	Relative Area %	Relative Height %	Amount n.a.
1		10.293	188.620	512.307	22.40	50.71	n.a.
2		30.297	653.299	497.947	77.60	49.29	n.a.
**Total:**			**841.918**	**1010.254**	**100.00**	**100.00**	

3ca: (inseparable diastereomers, 86:14 dr):

Chromatogram

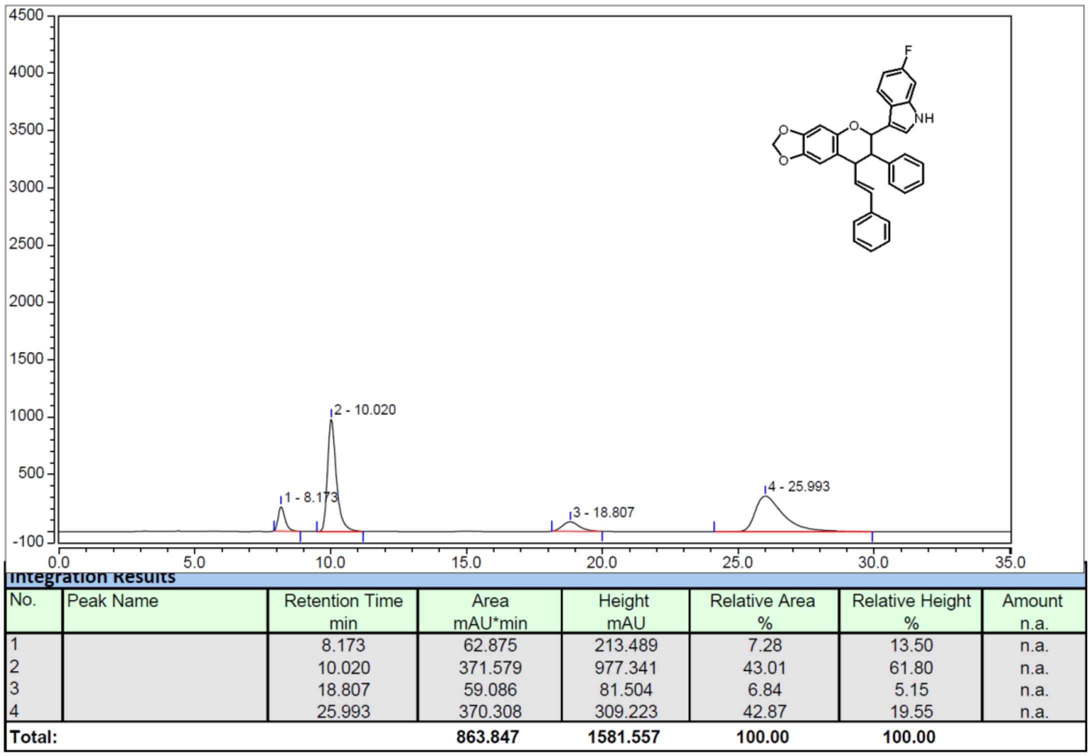

Integration Results

No.	Peak Name	Retention Time min	Area mAU*min	Height mAU	Relative Area %	Relative Height %	Amount n.a.
1		8.173	62.875	213.489	7.28	13.50	n.a.
2		10.020	371.579	977.341	43.01	61.80	n.a.
3		18.807	59.086	81.504	6.84	5.15	n.a.
4		25.993	370.308	309.223	42.87	19.55	n.a.
**Total:**			**863.847**	**1581.557**	**100.00**	**100.00**	

Chromatogram

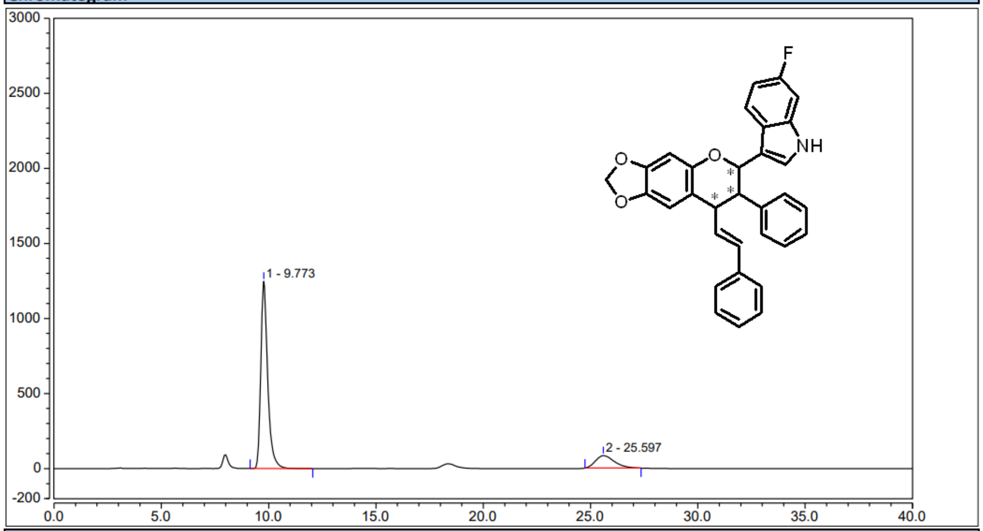

Integration Results

No.	Peak Name	Retention Time min	Area mAU*min	Height mAU	Relative Area %	Relative Height %	Amount n.a.
1		9.773	465.084	1247.252	84.58	93.77	n.a.
2		25.597	84.799	82.924	15.42	6.23	n.a.
**Total:**			**549.882**	**1330.177**	**100.00**	**100.00**	

3da: (inseparable diastereomers, 91:9 dr):

Chromatogram

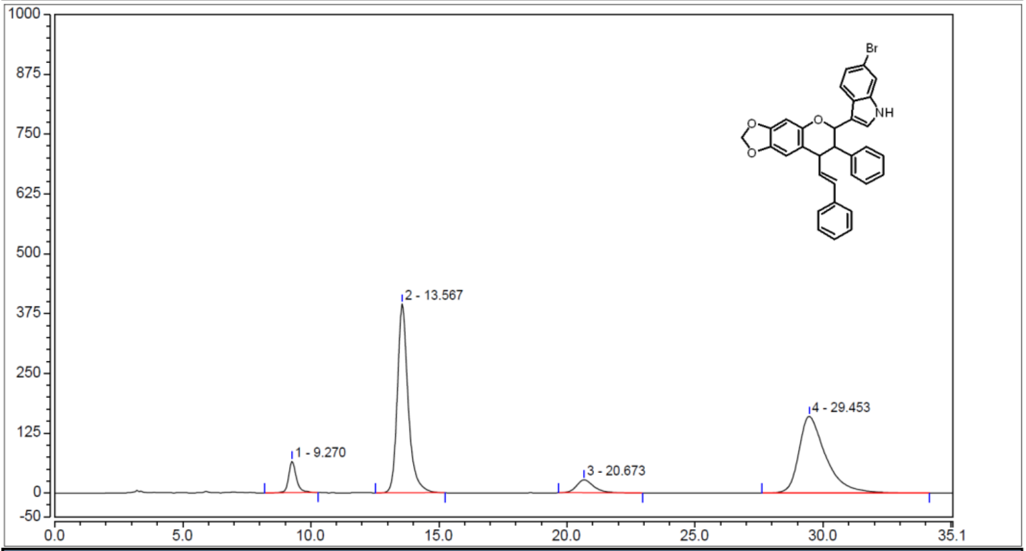

Integration Results

No.	Peak Name	Retention Time min	Area mAU*min	Height mAU	Relative Area %	Relative Height %	Amount n.a.
1		9.270	22.845	65.645	5.27	10.13	n.a.
2		13.567	193.963	394.663	44.77	60.93	n.a.
3		20.673	21.666	27.127	5.00	4.19	n.a.
4		29.453	194.815	160.318	44.96	24.75	n.a.
**Total:**			**433.288**	**647.753**	**100.00**	**100.00**	

Chromatogram

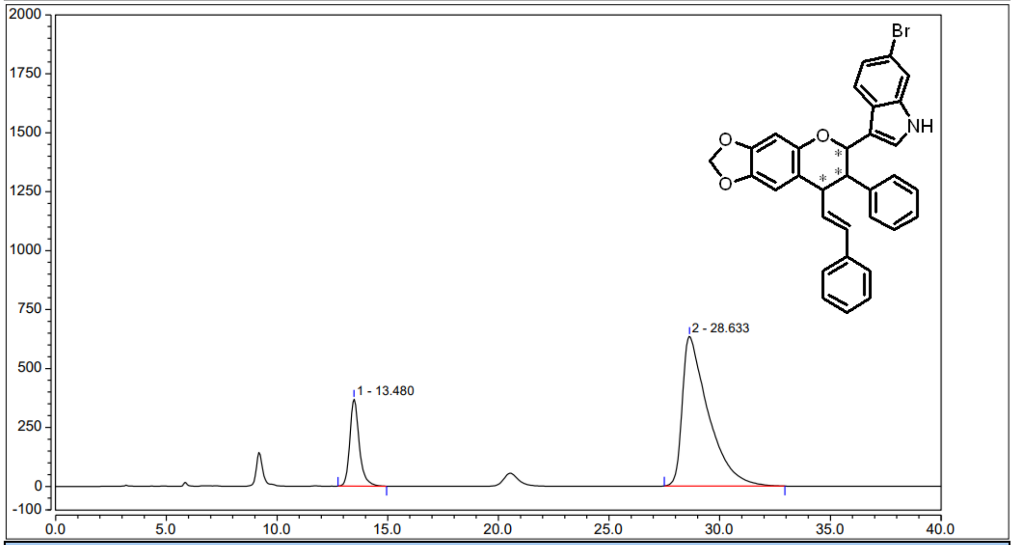

Integration Results

No.	Peak Name	Retention Time min	Area mAU*min	Height mAU	Relative Area %	Relative Height %	Amount n.a.
1		13.480	182.928	369.296	17.47	36.79	n.a.
2		28.633	864.015	634.416	82.53	63.21	n.a.
**Total:**			**1046.943**	**1003.713**	**100.00**	**100.00**	

3ea: (inseparable diastereomers, 85:15 dr):

Chromatogram

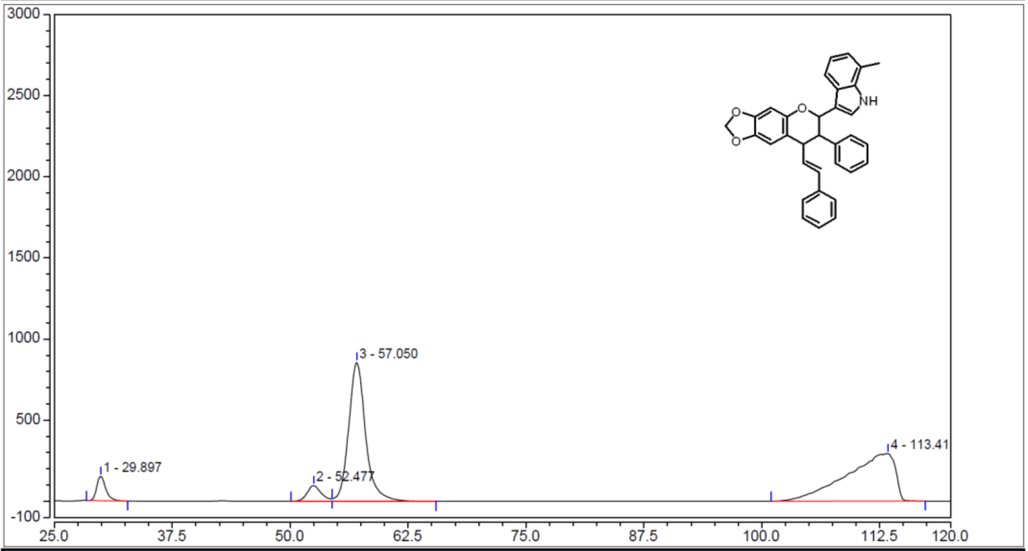

Integration Results

No.	Peak Name	Retention Time min	Area mAU*min	Height mAU	Relative Area %	Relative Height %	Amount n.a.
1		29.897	166.947	150.398	4.30	10.77	n.a.
2		52.477	167.824	96.223	4.32	6.89	n.a.
3		57.050	1783.407	855.641	45.93	61.27	n.a.
4		113.413	1764.518	294.286	45.45	21.07	n.a.
**Total:**			**3882.695**	**1396.548**	**100.00**	**100.00**	

Chromatogram

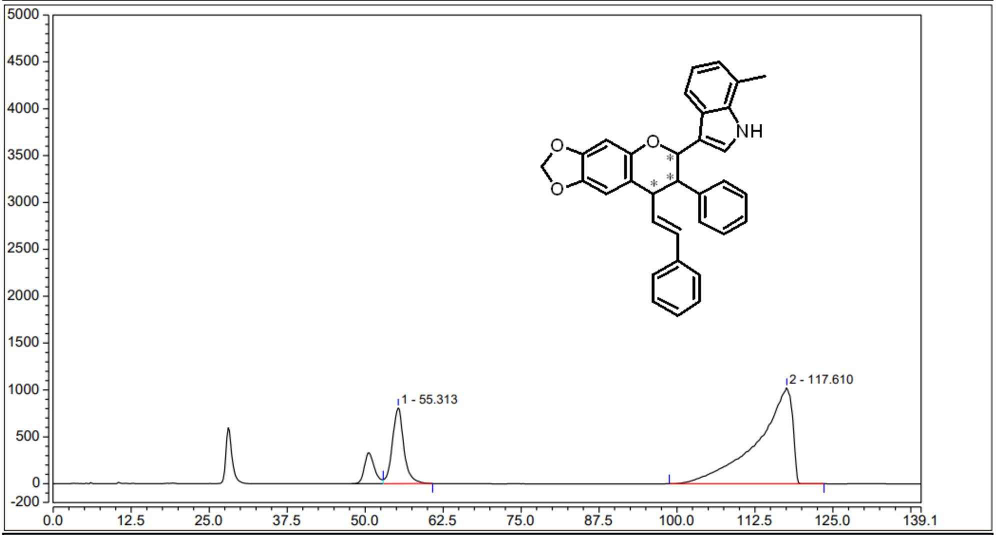

Integration Results

No.	Peak Name	Retention Time min	Area mAU*min	Height mAU	Relative Area %	Relative Height %	Amount n.a.
1		55.313	1732.350	808.428	20.75	44.04	n.a.
2		117.610	6616.316	1027.063	79.25	55.96	n.a.
**Total:**			**8348.666**	**1835.491**	**100.00**	**100.00**	

3fa: (inseparable diastereomers, 88:12 dr):

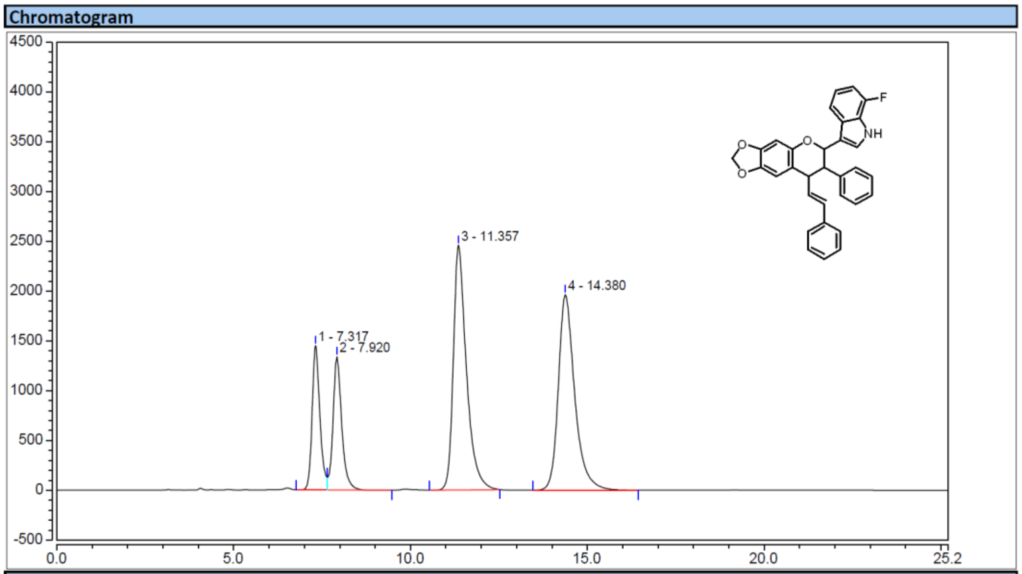

Integration Results

No.	Peak Name	Retention Time min	Area mAU*min	Height mAU	Relative Area %	Relative Height %	Amount n.a.
1		7.317	369.141	1449.795	12.91	20.10	n.a.
2		7.920	383.610	1339.719	13.42	18.58	n.a.
3		11.357	1053.430	2458.223	36.85	34.08	n.a.
4		14.380	1052.689	1964.548	36.82	27.24	n.a.
**Total:**			**2858.871**	**7212.285**	**100.00**	**100.00**	

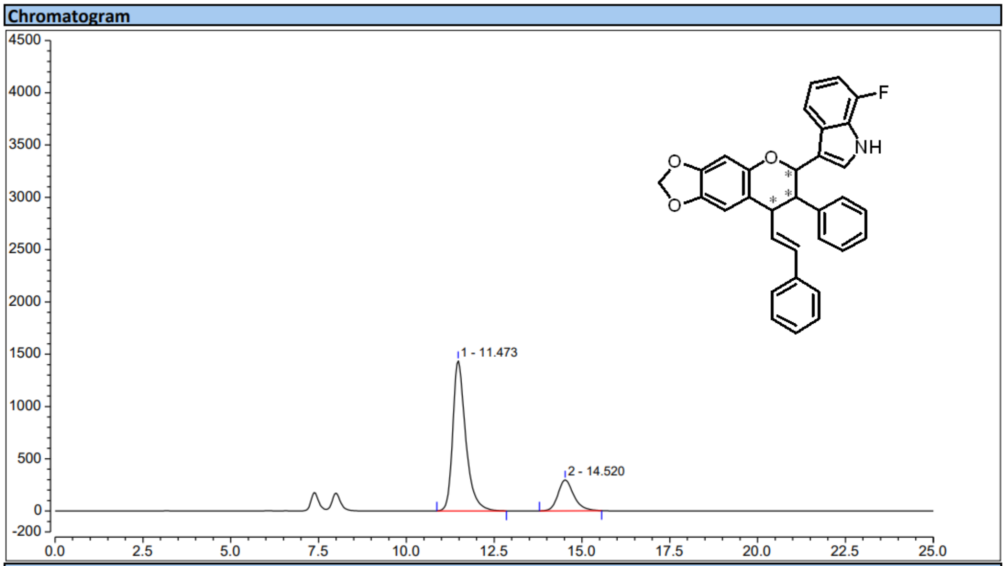

Integration Results

No.	Peak Name	Retention Time min	Area mAU*min	Height mAU	Relative Area %	Relative Height %	Amount n.a.
1		11.473	615.337	1436.521	79.95	82.95	n.a.
2		14.520	154.350	295.335	20.05	17.05	n.a.
**Total:**			**769.687**	**1731.856**	**100.00**	**100.00**	

3ga: (inseparable diastereomers, 93:7 dr):

Chromatogram

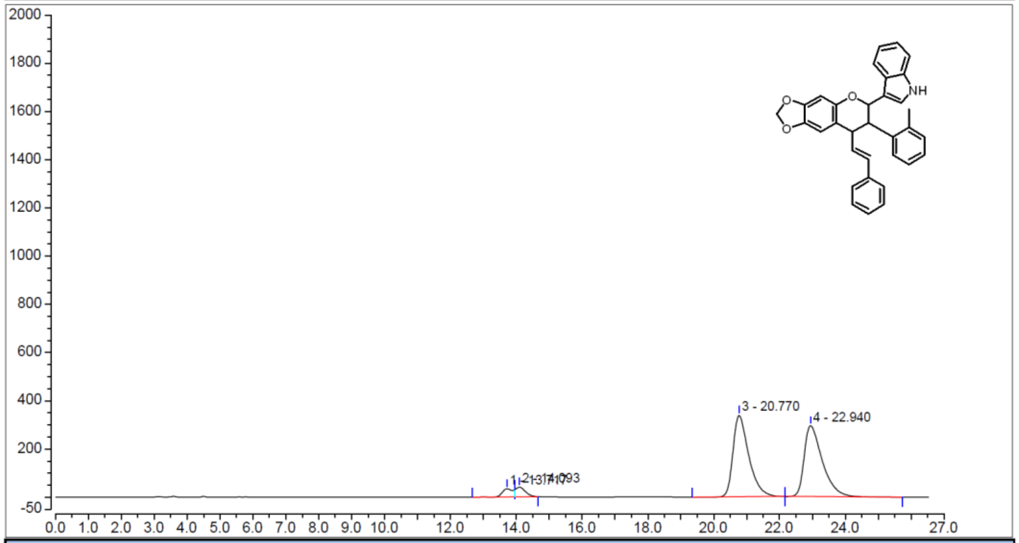

Integration Results

No.	Peak Name	Retention Time min	Area mAU*min	Height mAU	Relative Area %	Relative Height %	Amount n.a.
1		13.717	12.771	33.673	3.19	4.78	n.a.
2		14.093	13.468	40.040	3.37	5.68	n.a.
3		20.770	186.667	337.530	46.64	47.88	n.a.
4		22.940	187.310	293.733	46.80	41.67	n.a.
**Total:**			**400.215**	**704.976**	**100.00**	**100.00**	

Chromatogram

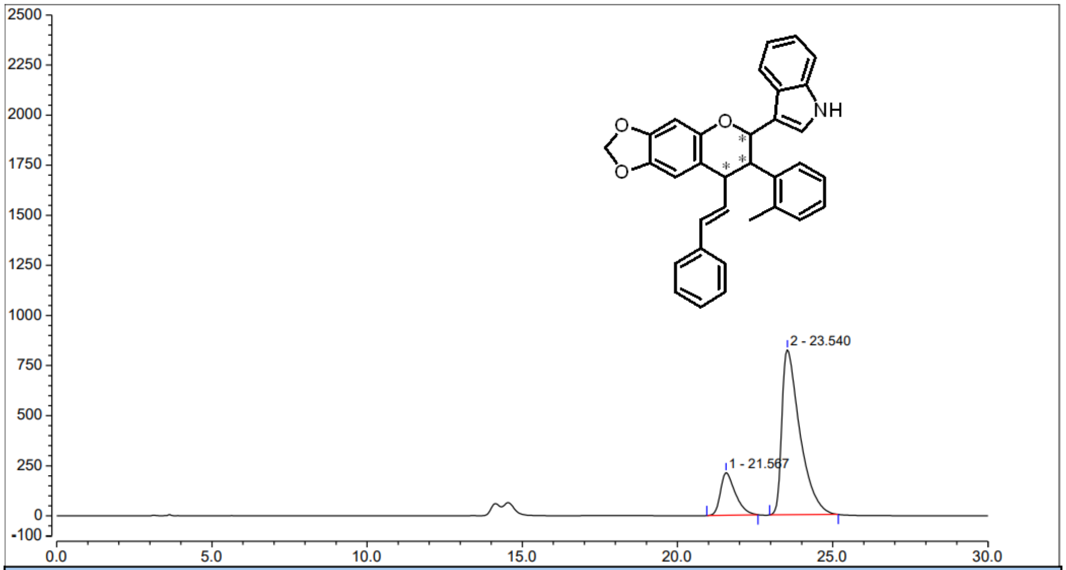

Integration Results

No.	Peak Name	Retention Time min	Area mAU*min	Height mAU	Relative Area %	Relative Height %	Amount n.a.
1		21.567	118.785	212.878	17.59	20.54	n.a.
2		23.540	556.426	823.696	82.41	79.46	n.a.
**Total:**			**675.210**	**1036.574**	**100.00**	**100.00**	

3ha: (inseparable diastereomers, 91:9 dr):

Chromatogram

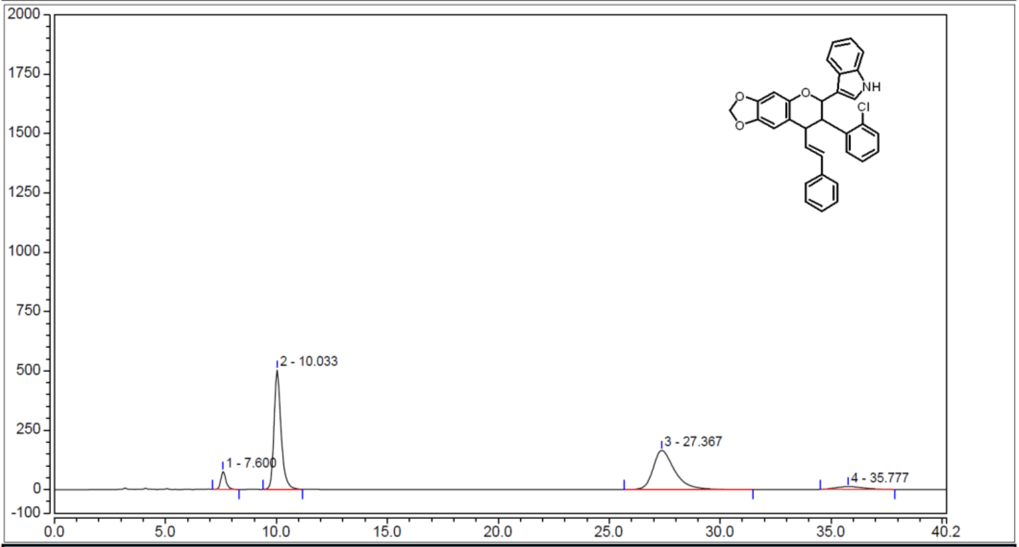

Integration Results

No.	Peak Name	Retention Time min	Area mAU*min	Height mAU	Relative Area %	Relative Height %	Amount n.a.
1		7.600	20.107	74.687	4.93	9.92	n.a.
2		10.033	185.047	501.894	45.36	66.63	n.a.
3		27.367	186.090	164.939	45.62	21.90	n.a.
4		35.777	16.682	11.703	4.09	1.55	n.a.
**Total:**			**407.925**	**753.222**	**100.00**	**100.00**	

Chromatogram

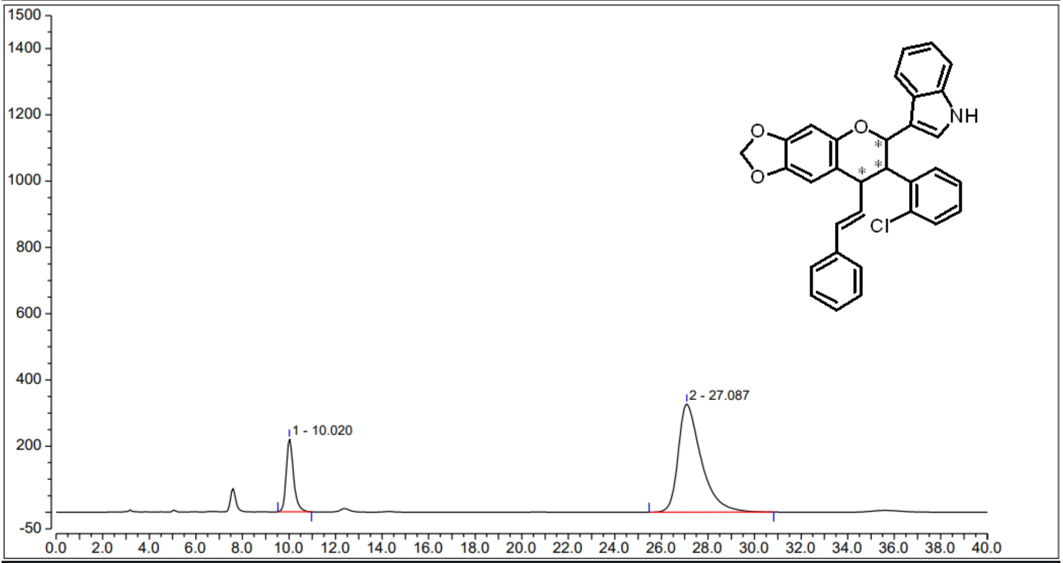

Integration Results

No.	Peak Name	Retention Time min	Area mAU*min	Height mAU	Relative Area %	Relative Height %	Amount n.a.
1		10.020	80.230	219.746	17.62	40.22	n.a.
2		27.087	375.176	326.666	82.38	59.78	n.a.
**Total:**			**455.407**	**546.412**	**100.00**	**100.00**	

3ia: (inseparable diastereomers, 92:8 dr):

Chromatogram

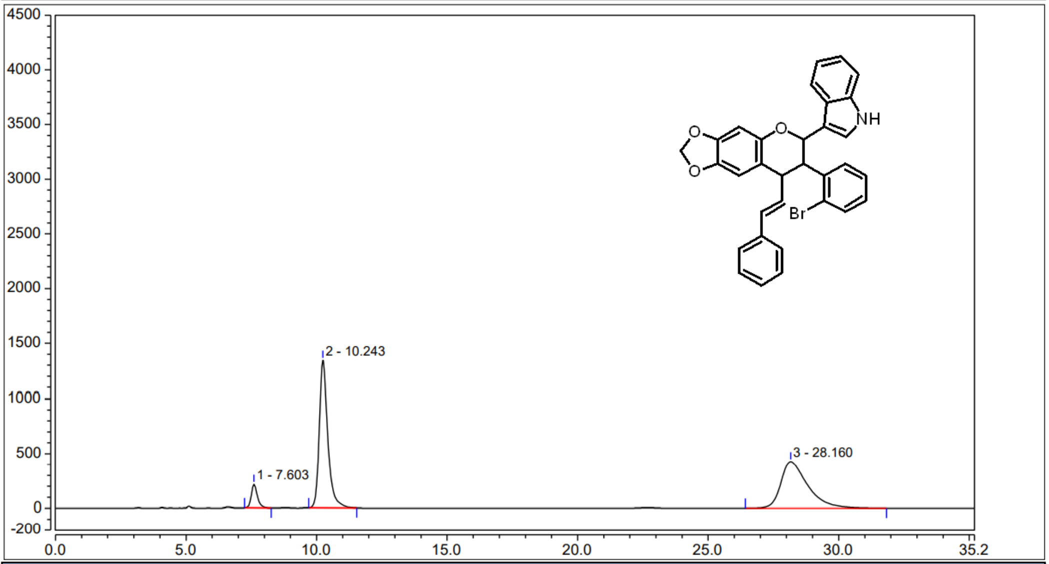

Integration Results

No.	Peak Name	Retention Time<br>min	Area<br>mAU*min	Height<br>mAU	Relative Area<br>%	Relative Height<br>%	Amount<br>n.a.
1		7.603	58.045	215.212	5.41	10.86	n.a.
2		10.243	507.912	1340.266	47.30	67.60	n.a.
3		28.160	507.784	427.031	47.29	21.54	n.a.
**Total:**			**1073.741**	**1982.508**	**100.00**	**100.00**	

Chromatogram

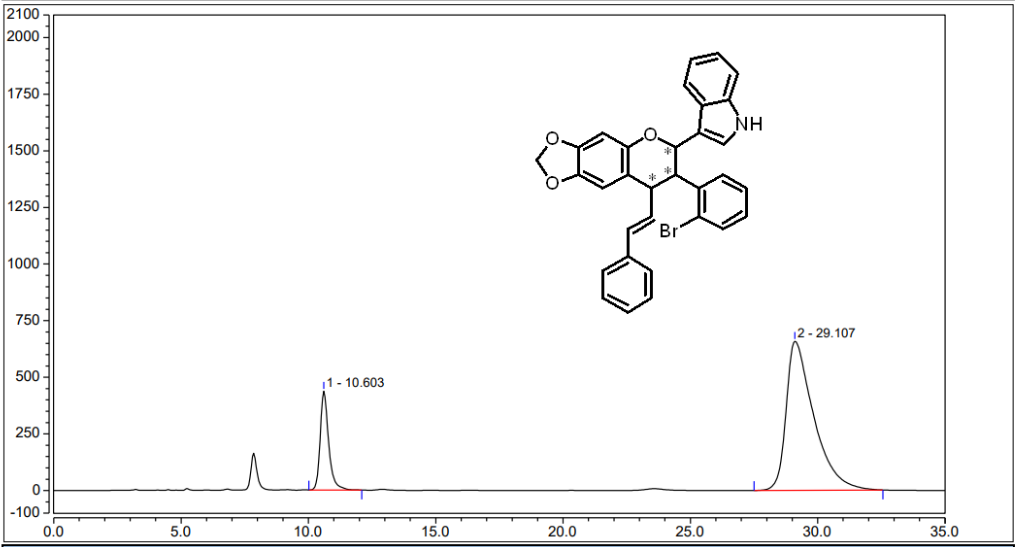

Integration Results

No.	Peak Name	Retention Time<br>min	Area<br>mAU*min	Height<br>mAU	Relative Area<br>%	Relative Height<br>%	Amount<br>n.a.
1		10.603	169.480	436.506	16.97	39.88	n.a.
2		29.107	829.471	658.166	83.03	60.12	n.a.
**Total:**			**998.951**	**1094.672**	**100.00**	**100.00**	

3ja: (inseparable diastereomers, 84:16 dr):

Chromatogram

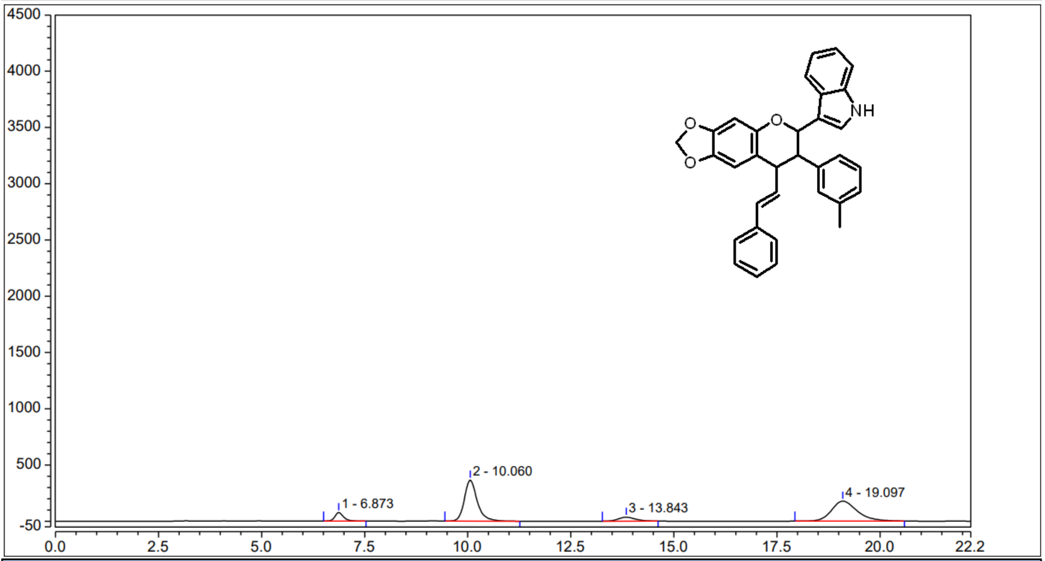

Integration Results

No.	Peak Name	Retention Time min	Area mAU*min	Height mAU	Relative Area %	Relative Height %	Amount n.a.
1		6.873	18.674	76.054	6.27	11.63	n.a.
2		10.060	132.853	365.232	44.57	55.84	n.a.
3		13.843	17.157	34.781	5.76	5.32	n.a.
4		19.097	129.364	177.973	43.40	27.21	n.a.
**Total:**			**298.049**	**654.040**	**100.00**	**100.00**	

Chromatogram

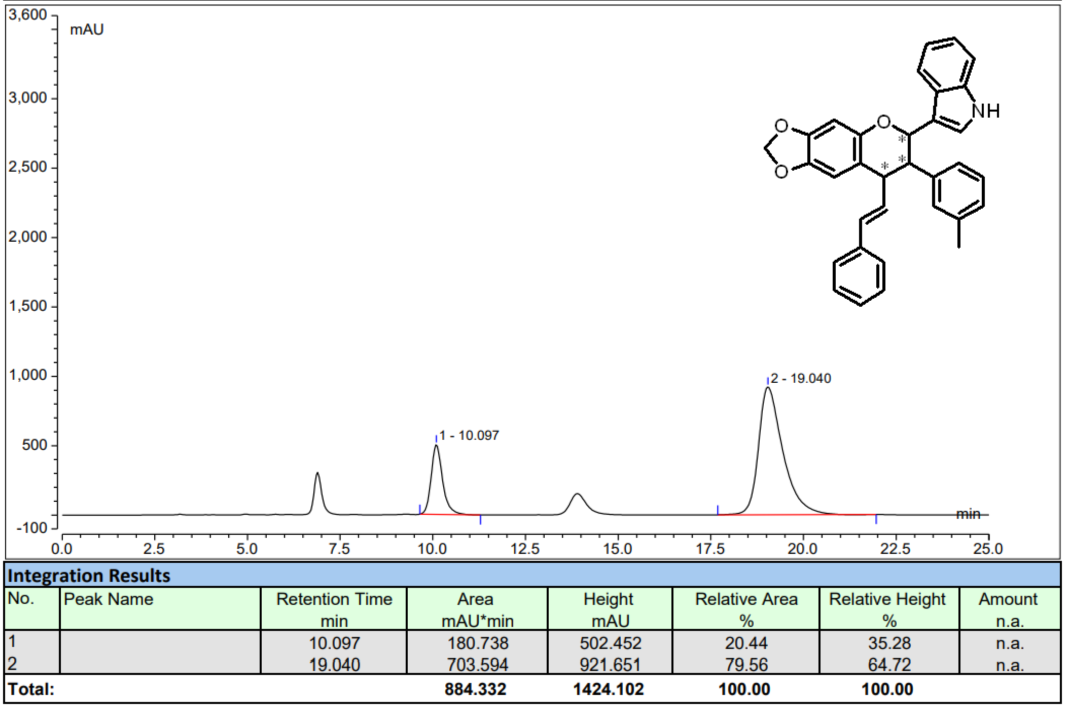

Integration Results

No.	Peak Name	Retention Time min	Area mAU*min	Height mAU	Relative Area %	Relative Height %	Amount n.a.
1		10.097	180.738	502.452	20.44	35.28	n.a.
2		19.040	703.594	921.651	79.56	64.72	n.a.
**Total:**			**884.332**	**1424.102**	**100.00**	**100.00**	

3ka: (inseparable diastereomers, 87:13 dr):

Chromatogram

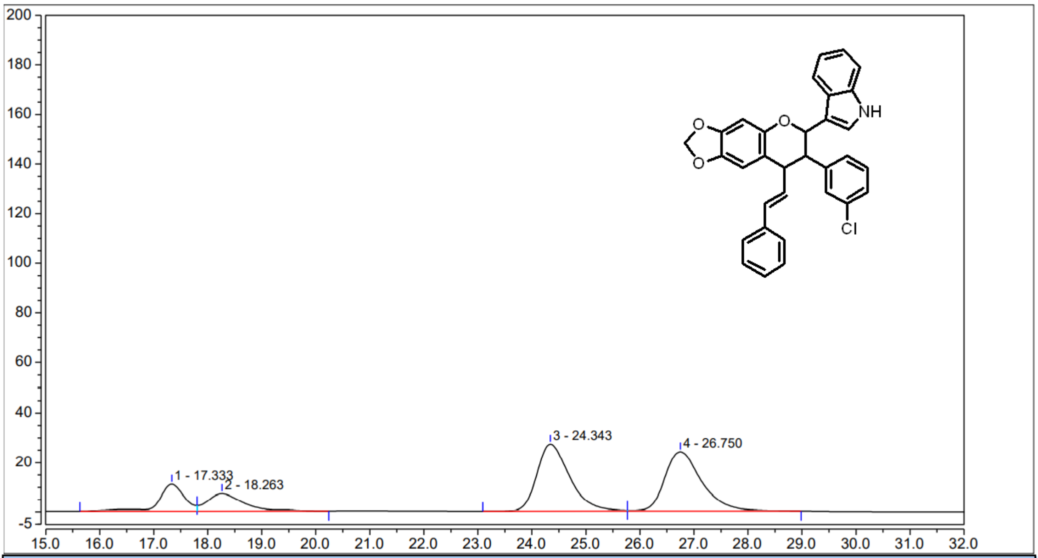

Integration Results

No.	Peak Name	Retention Time min	Area mAU*min	Height mAU	Relative Area %	Relative Height %	Amount n.a.
1		17.333	6.280	11.060	12.34	15.89	n.a.
2		18.263	5.833	7.224	11.46	10.38	n.a.
3		24.343	19.372	27.247	38.06	39.16	n.a.
4		26.750	19.417	24.051	38.14	34.56	n.a.
**Total:**			**50.903**	**69.581**	**100.00**	**100.00**	

Chromatogram

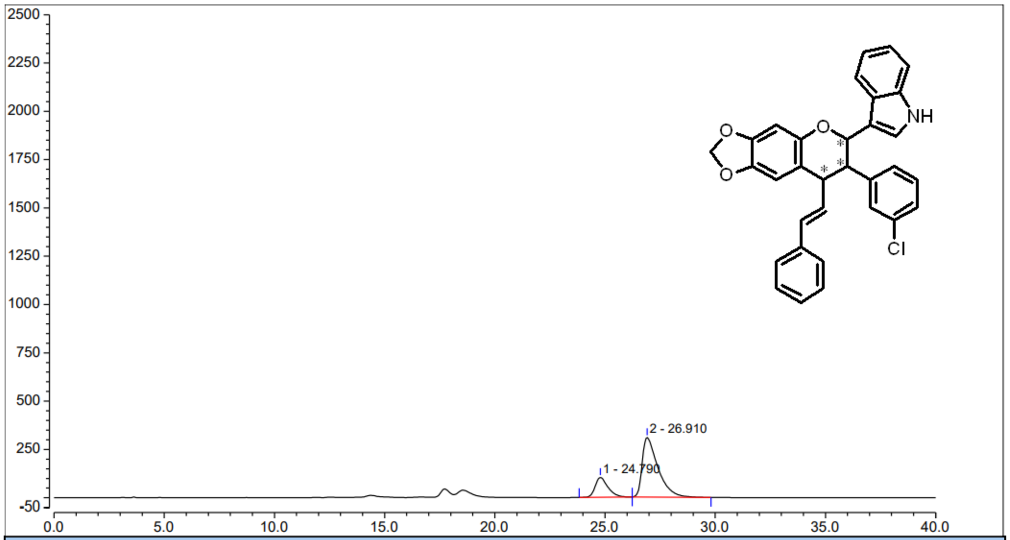

Integration Results

No.	Peak Name	Retention Time min	Area mAU*min	Height mAU	Relative Area %	Relative Height %	Amount n.a.
1		24.790	68.158	102.397	21.08	24.92	n.a.
2		26.910	255.248	308.475	78.92	75.08	n.a.
**Total:**			**323.406**	**410.872**	**100.00**	**100.00**	

3la: (inseparable diastereomers, 78:22 dr):

Chromatogram

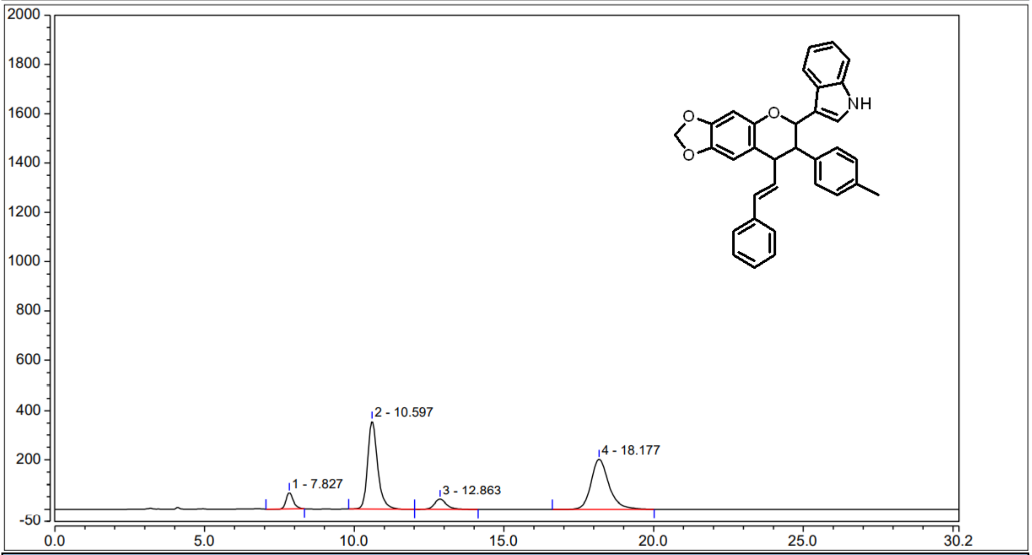

Integration Results

No.	Peak Name	Retention Time min	Area mAU*min	Height mAU	Relative Area %	Relative Height %	Amount n.a.
1		7.827	17.723	65.250	5.67	9.84	n.a.
2		10.597	137.192	357.070	43.88	53.82	n.a.
3		12.863	18.770	39.846	6.00	6.01	n.a.
4		18.177	138.947	201.253	44.44	30.34	n.a.
Total:			312.632	663.419	100.00	100.00	

Chromatogram

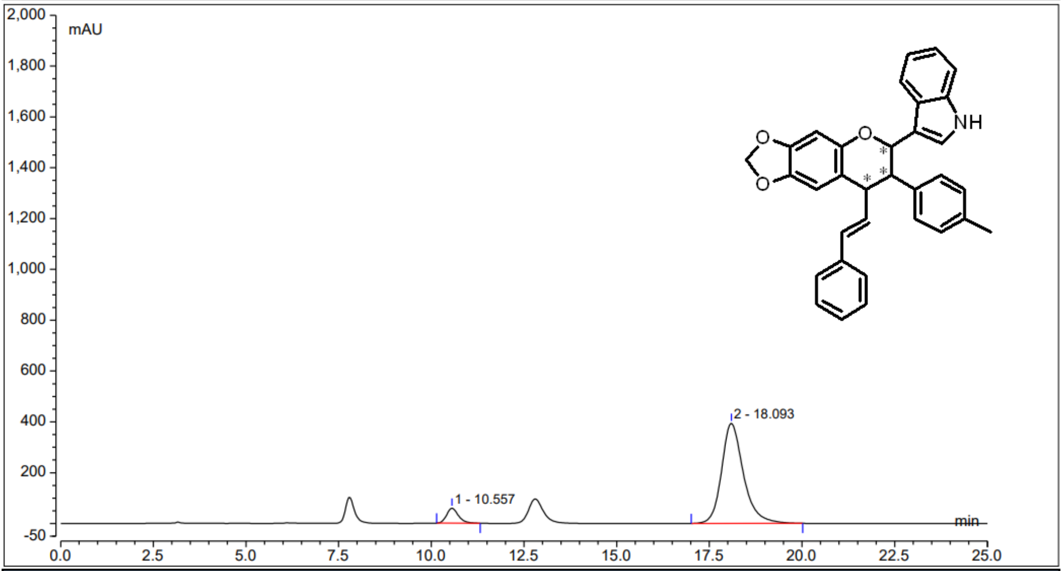

Integration Results

No.	Peak Name	Retention Time min	Area mAU*min	Height mAU	Relative Area %	Relative Height %	Amount n.a.
1		10.557	21.844	59.039	7.49	13.04	n.a.
2		18.093	269.977	393.780	92.51	86.96	n.a.
Total:			291.821	452.819	100.00	100.00	

3ma: (inseparable diastereomers, 84:16 dr):

Chromatogram

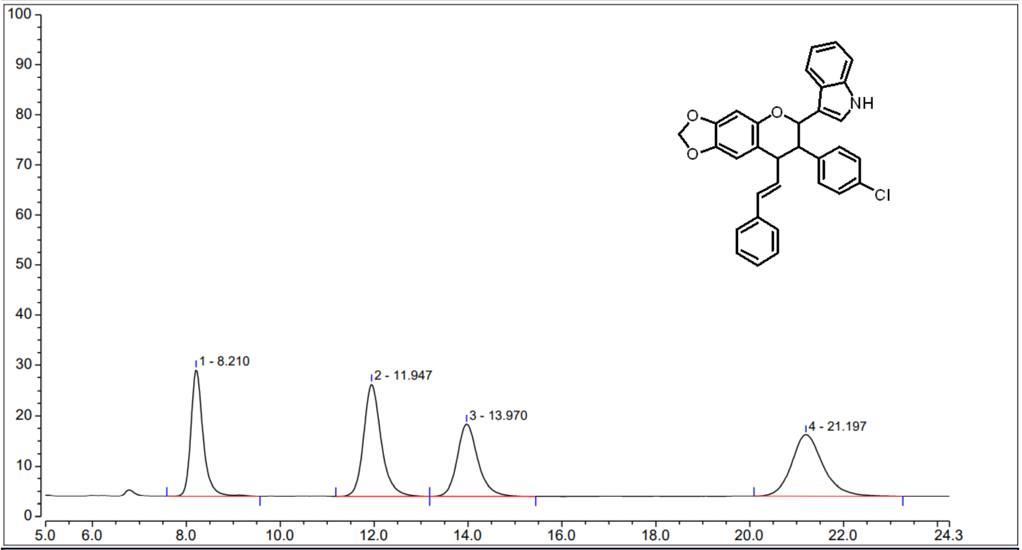

Integration Results

No.	Peak Name	Retention Time min	Area mAU*min	Height mAU	Relative Area %	Relative Height %	Amount n.a.
1		8.210	7.607	25.049	22.01	33.84	n.a.
2		11.947	9.813	22.312	28.40	30.14	n.a.
3		13.970	7.493	14.426	21.68	19.49	n.a.
4		21.197	9.642	12.244	27.90	16.54	n.a.
**Total:**			**34.554**	**74.030**	**100.00**	**100.00**	

Chromatogram

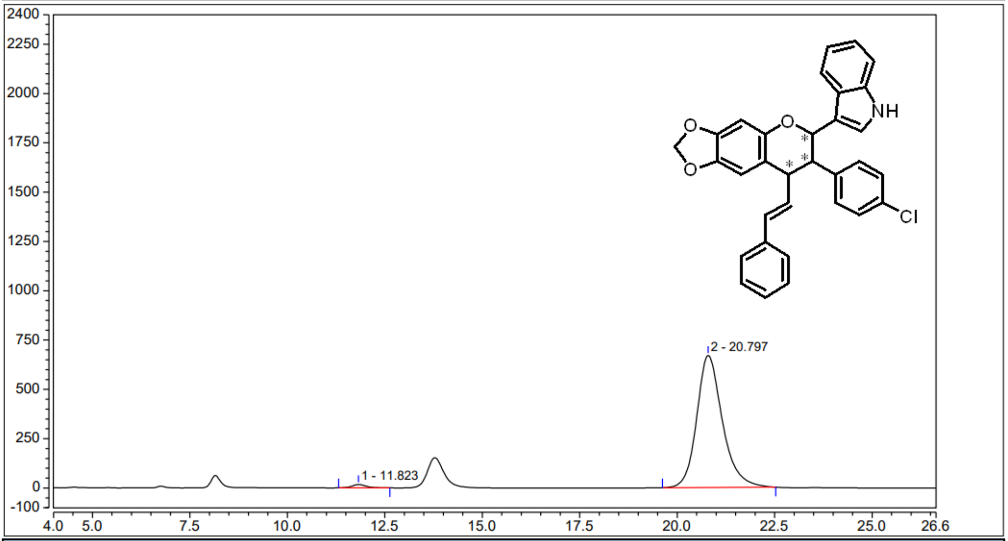

Integration Results

No.	Peak Name	Retention Time min	Area mAU*min	Height mAU	Relative Area %	Relative Height %	Amount n.a.
1		11.823	7.325	17.186	1.41	2.50	n.a.
2		20.797	512.380	670.080	98.59	97.50	n.a.
**Total:**			**519.705**	**687.266**	**100.00**	**100.00**	

3na: (inseparable diastereomers, 86:14 dr):

Chromatogram

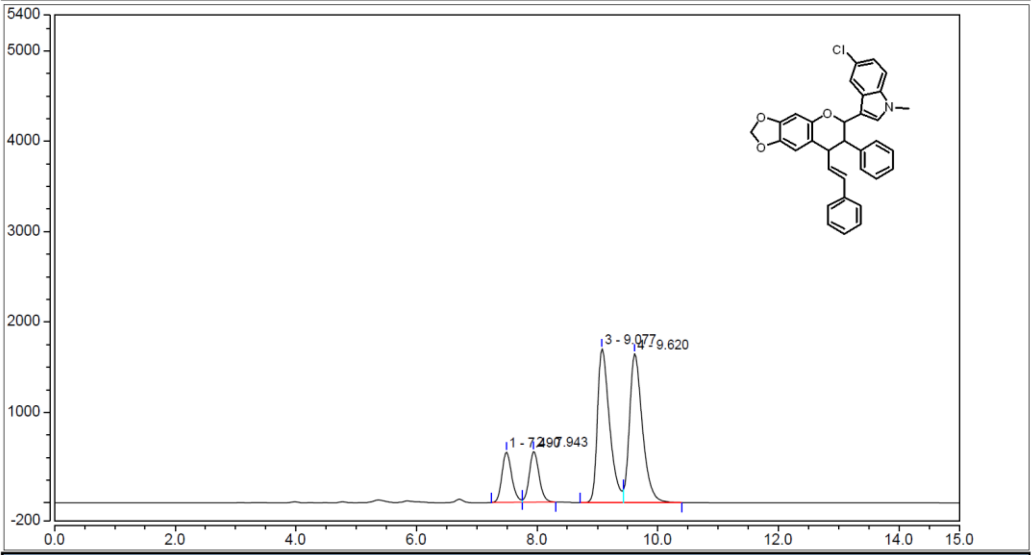

Integration Results

No.	Peak Name	Retention Time min	Area mAU*min	Height mAU	Relative Area %	Relative Height %	Amount n.a.
1		7.490	105.501	556.359	10.38	12.45	n.a.
2		7.943	106.039	562.218	10.43	12.58	n.a.
3		9.077	399.136	1704.093	39.26	38.13	n.a.
4		9.620	405.909	1647.071	39.93	36.85	n.a.
**Total:**			**1016.585**	**4469.741**	**100.00**	**100.00**	

Chromatogram

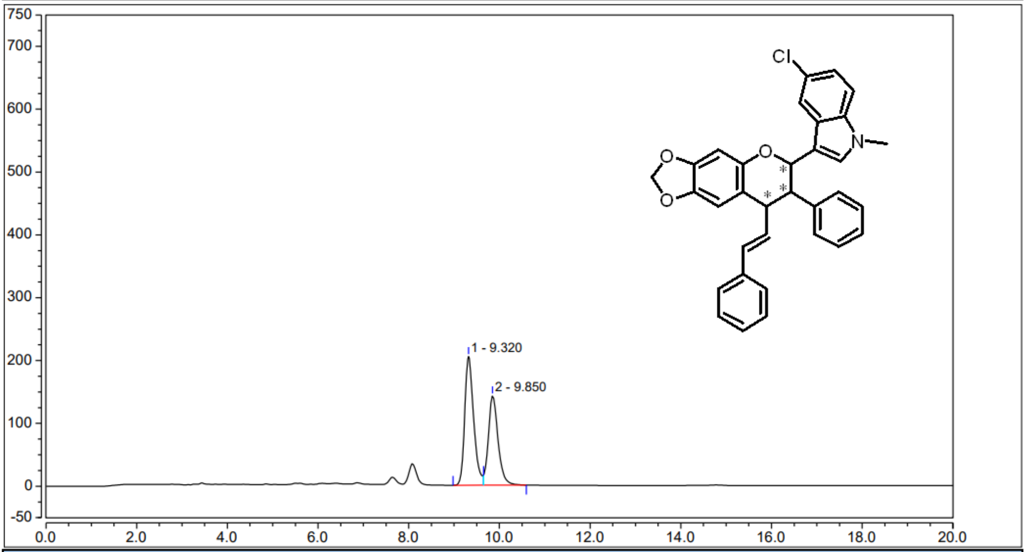

Integration Results

No.	Peak Name	Retention Time min	Area mAU*min	Height mAU	Relative Area %	Relative Height %	Amount n.a.
1		9.320	47.104	204.909	57.18	59.01	n.a.
2		9.850	35.272	142.324	42.82	40.99	n.a.
**Total:**			**82.375**	**347.233**	**100.00**	**100.00**	

3oa: (inseparable diastereomers, 91:9 dr)

Chromatogram

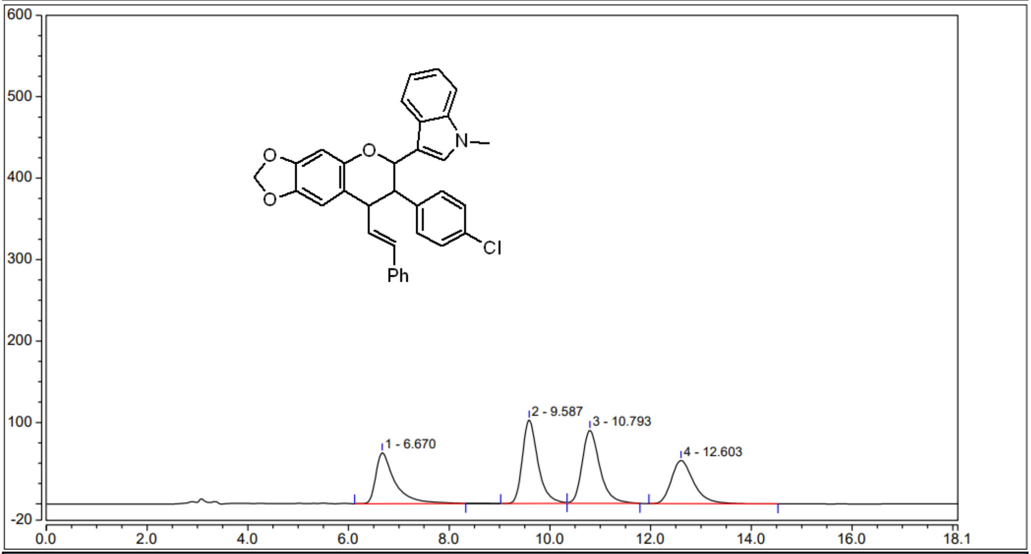

Integration Results

No.	Peak Name	Retention Time min	Area mAU*min	Height mAU	Relative Area %	Relative Height %	Amount n.a.
1		6.670	27.649	62.527	21.48	20.31	n.a.
2		9.587	36.958	102.554	28.72	33.31	n.a.
3		10.793	36.685	89.690	28.51	29.13	n.a.
4		12.603	27.397	53.133	21.29	17.26	n.a.
**Total:**			**128.689**	**307.904**	**100.00**	**100.00**	

Chromatogram

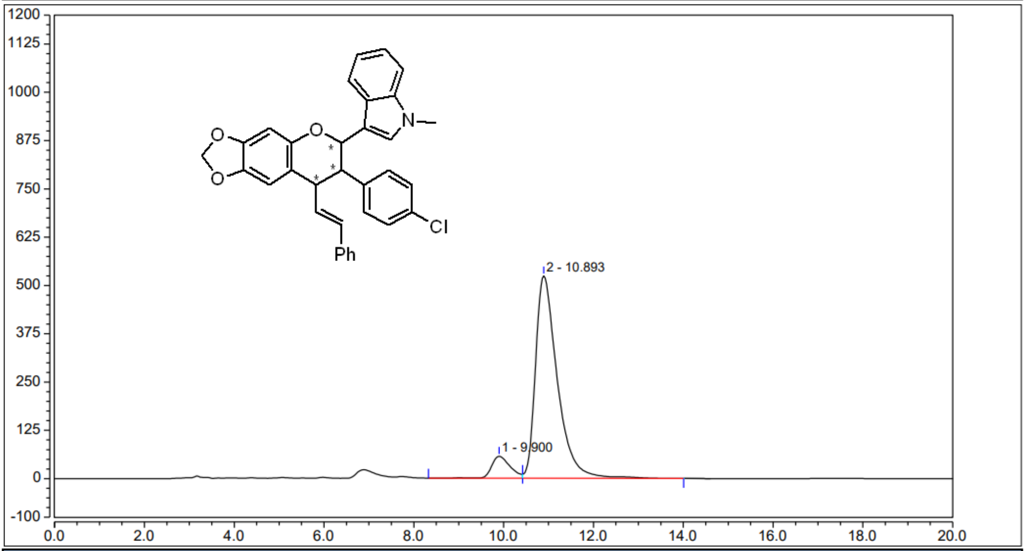

Integration Results

No.	Peak Name	Retention Time min	Area mAU*min	Height mAU	Relative Area %	Relative Height %	Amount n.a.
1		9.900	26.984	56.958	8.38	9.81	n.a.
2		10.893	295.137	523.863	91.62	90.19	n.a.
**Total:**			**322.121**	**580.820**	**100.00**	**100.00**	

3ab: (inseparable diastereomers, 88:12 dr):

Chromatogram

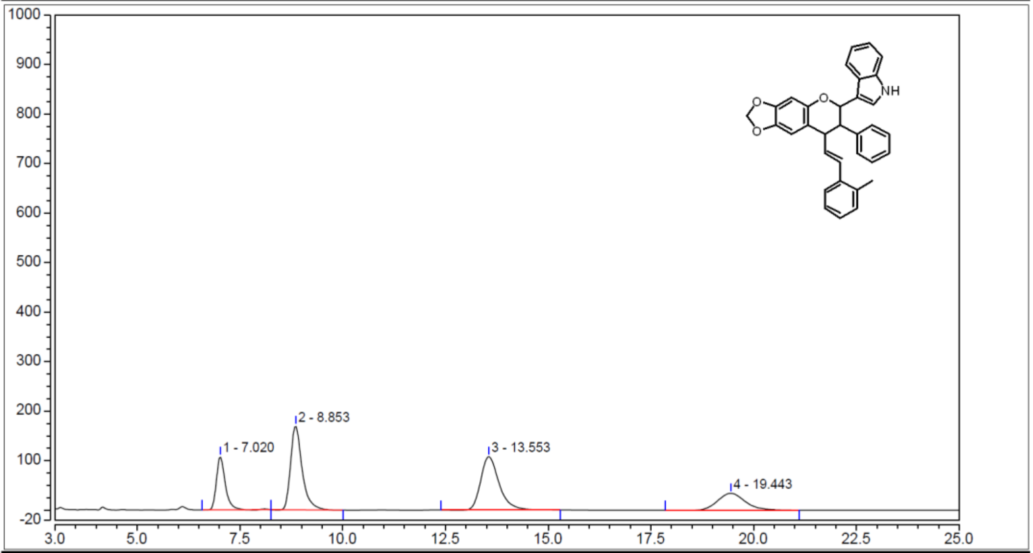

Integration Results

No.	Peak Name	Retention Time<br>min	Area<br>mAU*min	Height<br>mAU	Relative Area<br>%	Relative Height<br>%	Amount<br>n.a.
1		7.020	26.709	107.264	16.21	25.61	n.a.
2		8.853	55.378	169.360	33.60	40.43	n.a.
3		13.553	55.525	107.713	33.69	25.72	n.a.
4		19.443	27.203	34.535	16.51	8.24	n.a.
**Total:**			**164.815**	**418.873**	**100.00**	**100.00**	

Chromatogram

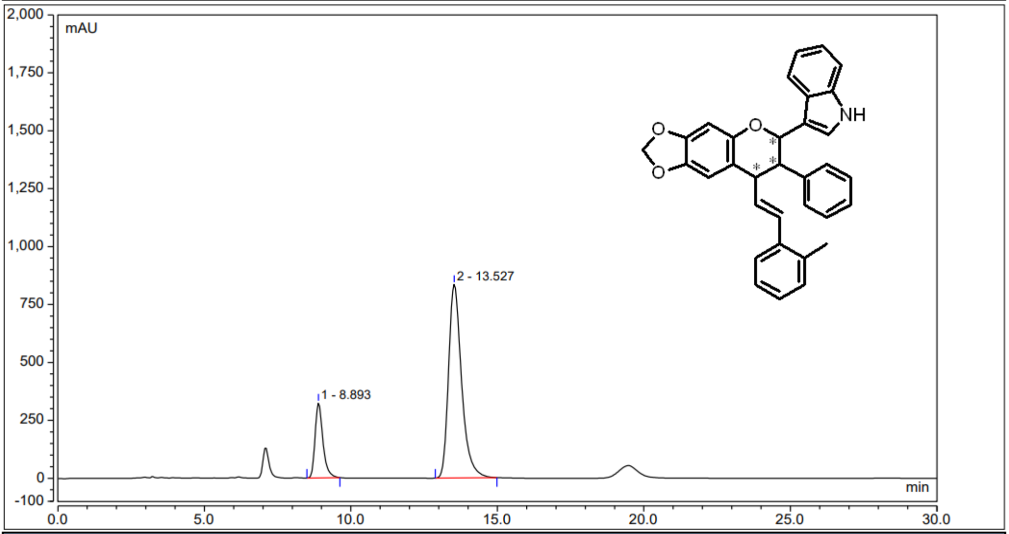

Integration Results

No.	Peak Name	Retention Time<br>min	Area<br>mAU*min	Height<br>mAU	Relative Area<br>%	Relative Height<br>%	Amount<br>n.a.
1		8.893	100.691	323.497	18.94	27.90	n.a.
2		13.527	430.971	835.944	81.06	72.10	n.a.
**Total:**			**531.662**	**1159.442**	**100.00**	**100.00**	

3ac: (inseparable diastereomers, 86:14 dr):

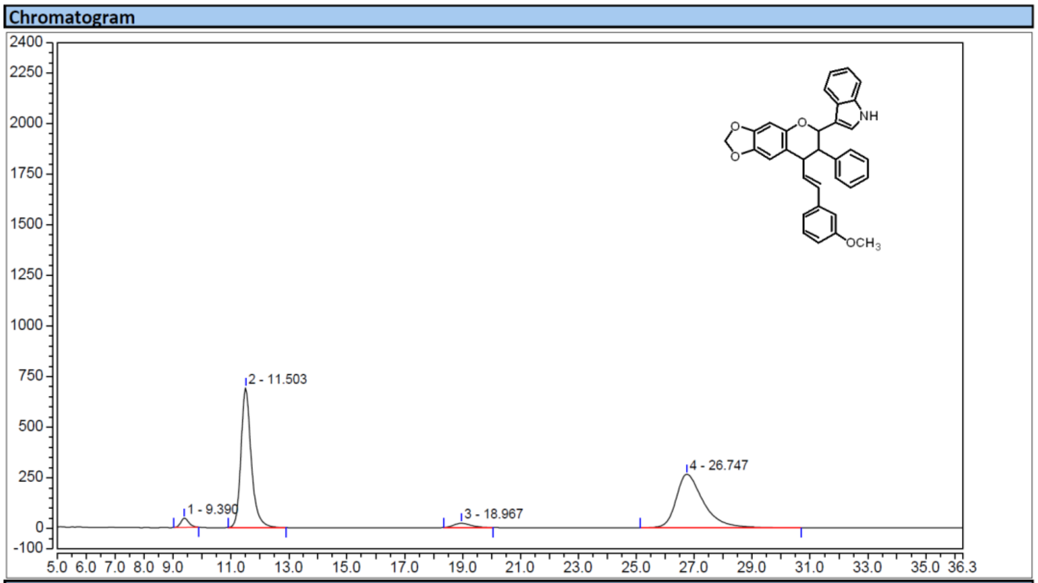

Integration Results

No.	Peak Name	Retention Time min	Area mAU*min	Height mAU	Relative Area %	Relative Height %	Amount n.a.
1		9.390	14.677	45.437	2.44	4.45	n.a.
2		11.503	285.848	689.857	47.49	67.55	n.a.
3		18.967	14.161	21.130	2.35	2.07	n.a.
4		26.747	287.290	264.764	47.72	25.93	n.a.
**Total:**			**601.976**	**1021.187**	**100.00**	**100.00**	

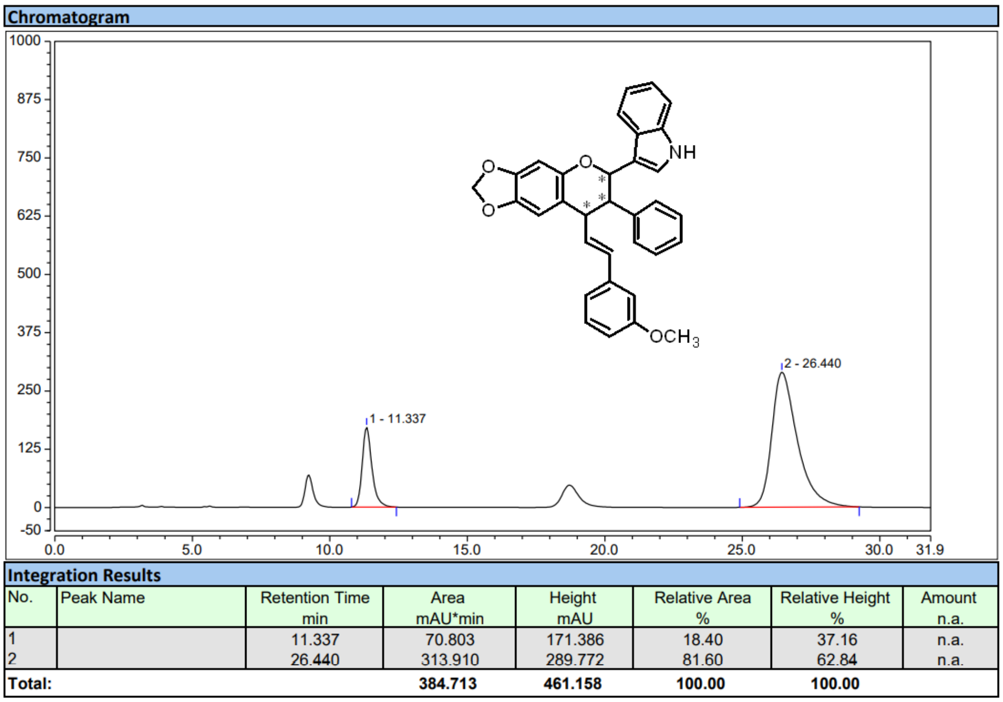

Integration Results

No.	Peak Name	Retention Time min	Area mAU*min	Height mAU	Relative Area %	Relative Height %	Amount n.a.
1		11.337	70.803	171.386	18.40	37.16	n.a.
2		26.440	313.910	289.772	81.60	62.84	n.a.
**Total:**			**384.713**	**461.158**	**100.00**	**100.00**	

3ad: (inseparable diastereomers, 85:15 dr):

Chromatogram

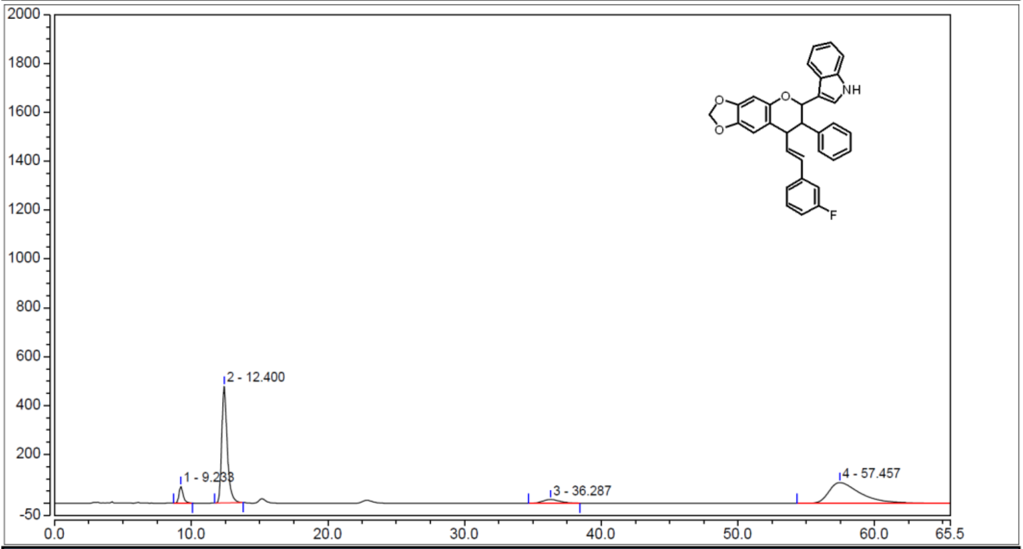

Integration Results

No.	Peak Name	Retention Time min	Area mAU*min	Height mAU	Relative Area %	Relative Height %	Amount n.a.
1		9.233	23.292	68.174	4.69	10.57	n.a.
2		12.400	224.749	477.260	45.23	73.99	n.a.
3		36.287	21.845	14.939	4.40	2.32	n.a.
4		57.457	226.986	84.655	45.68	13.12	n.a.
**Total:**			**496.872**	**645.029**	**100.00**	**100.00**	

Chromatogram

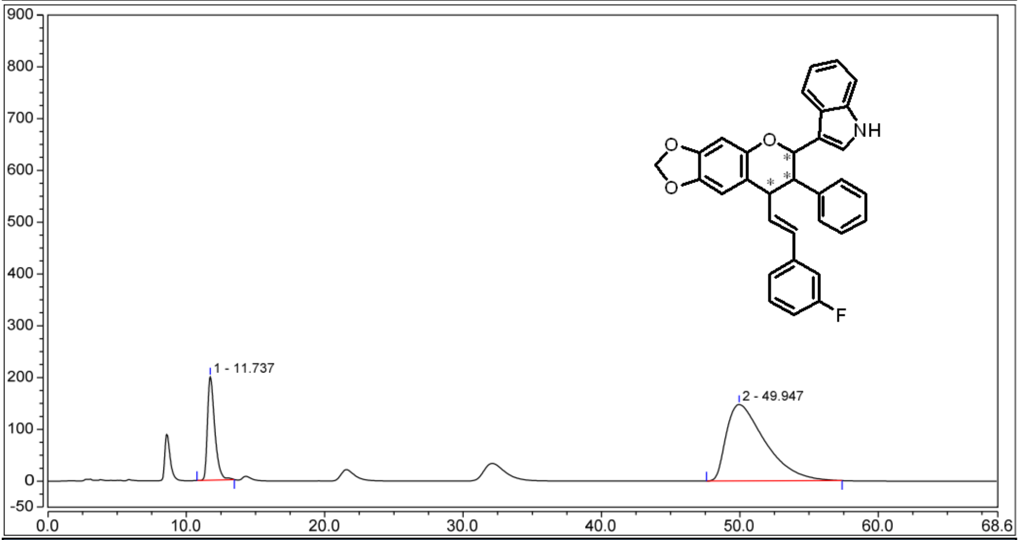

Integration Results

No.	Peak Name	Retention Time min	Area mAU*min	Height mAU	Relative Area %	Relative Height %	Amount n.a.
1		11.737	122.169	199.380	20.24	57.47	n.a.
2		49.947	481.368	147.523	79.76	42.53	n.a.
**Total:**			**603.537**	**346.904**	**100.00**	**100.00**	

3ae: (inseparable diastereomers, 89:11 dr):

Chromatogram

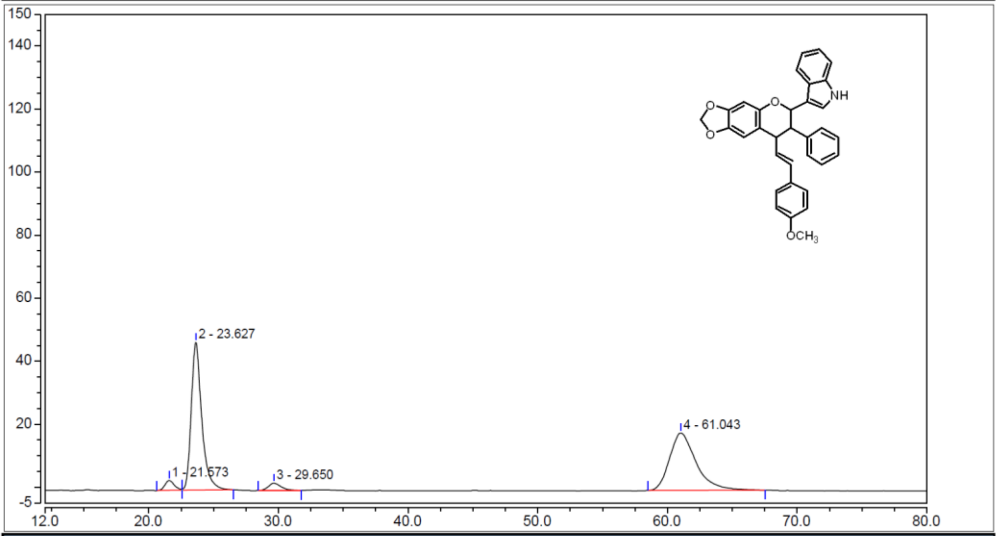

Integration Results

No.	Peak Name	Retention Time min	Area mAU*min	Height mAU	Relative Area %	Relative Height %	Amount n.a.
1		21.573	2.656	3.165	2.86	4.48	n.a.
2		23.627	44.207	46.906	47.64	66.43	n.a.
3		29.650	2.610	2.307	2.81	3.27	n.a.
4		61.043	43.319	18.234	46.68	25.82	n.a.
**Total:**			**92.791**	**70.612**	**100.00**	**100.00**	

Chromatogram

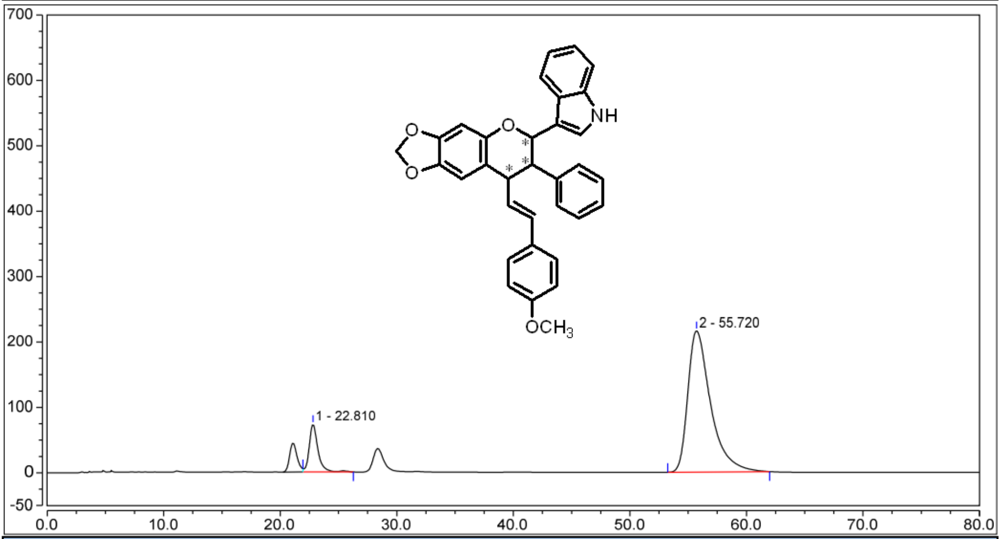

Integration Results

No.	Peak Name	Retention Time min	Area mAU*min	Height mAU	Relative Area %	Relative Height %	Amount n.a.
1		22.810	64.341	72.669	11.61	25.17	n.a.
2		55.720	489.657	216.027	88.39	74.83	n.a.
**Total:**			**553.999**	**288.697**	**100.00**	**100.00**	

3af: (inseparable diastereomers, 75:25 dr):

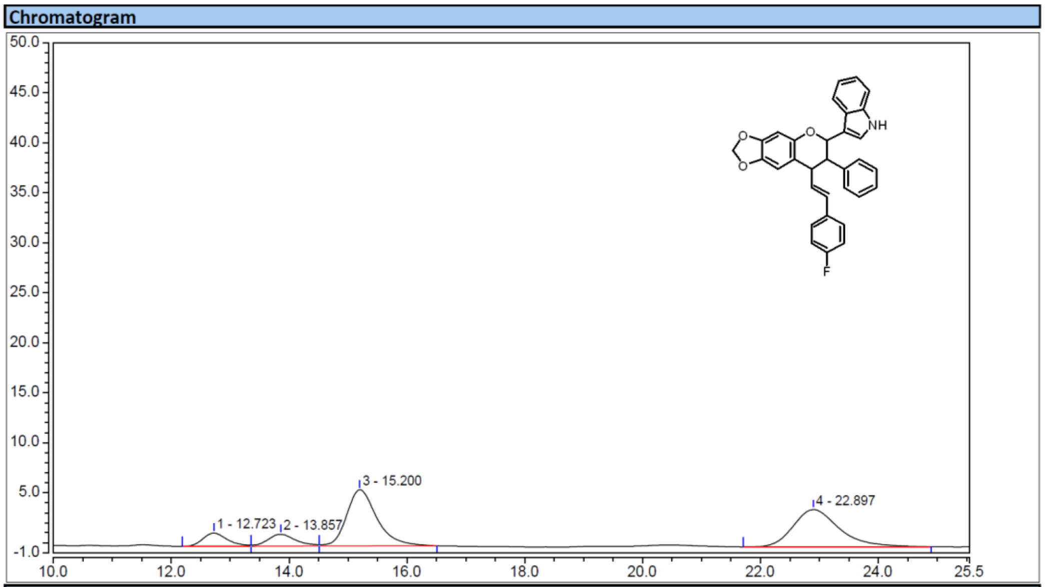

Integration Results

No.	Peak Name	Retention Time min	Area mAU*min	Height mAU	Relative Area %	Relative Height %	Amount n.a.
1		12.723	0.634	1.311	7.91	11.08	n.a.
2		13.857	0.649	1.181	8.10	9.98	n.a.
3		15.200	3.360	5.623	41.92	47.53	n.a.
4		22.897	3.373	3.717	42.07	31.41	n.a.
**Total:**			**8.016**	**11.832**	**100.00**	**100.00**	

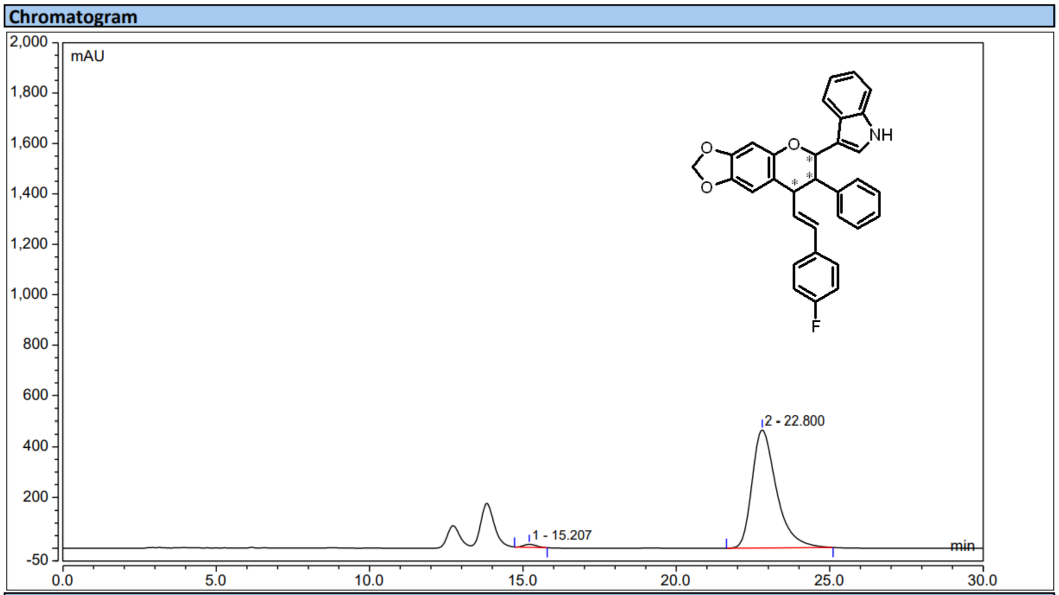

Integration Results

No.	Peak Name	Retention Time min	Area mAU*min	Height mAU	Relative Area %	Relative Height %	Amount n.a.
1		15.207	5.663	12.000	1.30	2.51	n.a.
2		22.800	430.768	466.309	98.70	97.49	n.a.
**Total:**			**436.431**	**478.309**	**100.00**	**100.00**	

3ag: (inseparable diastereomers, 84:16 dr):

Chromatogram

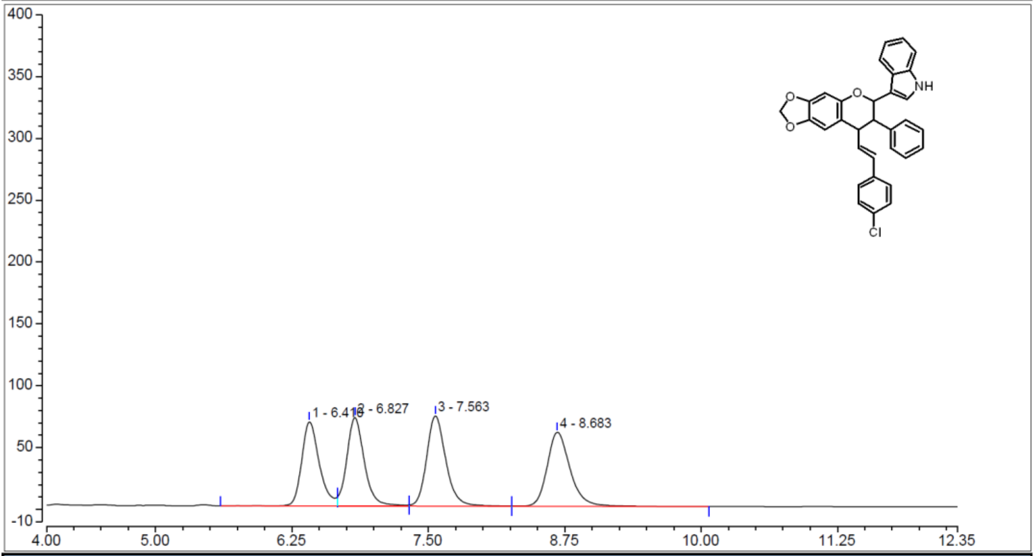

Integration Results

No.	Peak Name	Retention Time min	Area mAU*min	Height mAU	Relative Area %	Relative Height %	Amount n.a.
1		6.410	12.767	68.080	22.87	24.96	n.a.
2		6.827	13.385	71.674	23.98	26.27	n.a.
3		7.563	14.976	73.044	26.83	26.78	n.a.
4		8.683	14.692	59.991	26.32	21.99	n.a.
**Total:**			**55.819**	**272.790**	**100.00**	**100.00**	

Chromatogram

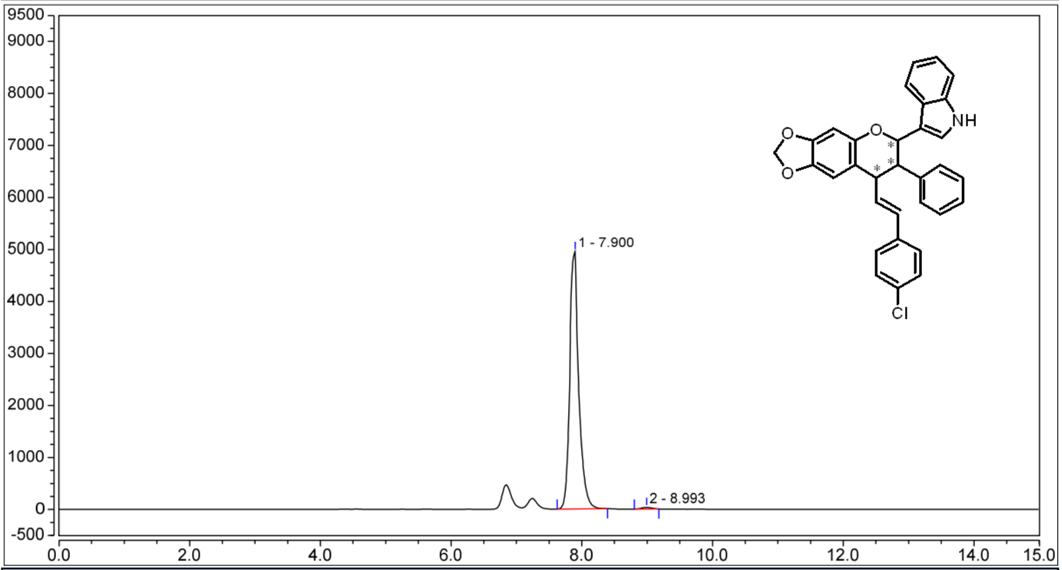

Integration Results

No.	Peak Name	Retention Time min	Area mAU*min	Height mAU	Relative Area %	Relative Height %	Amount n.a.
1		7.900	790.894	4950.800	99.24	99.35	n.a.
2		8.993	6.065	32.188	0.76	0.65	n.a.
**Total:**			**796.958**	**4982.988**	**100.00**	**100.00**	

3ah: (inseparable diastereomers, 85:15 dr):

Chromatogram

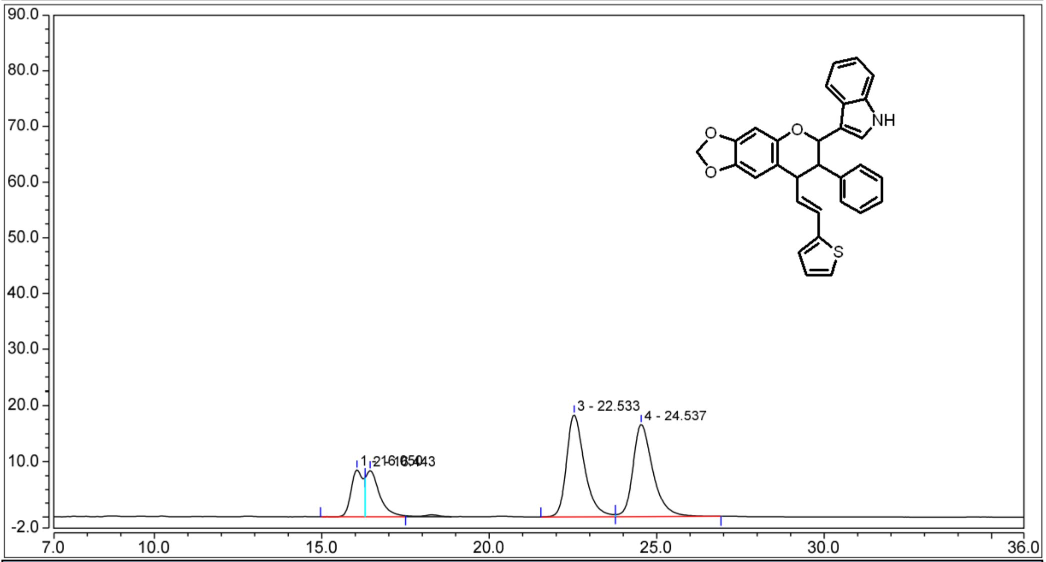

Integration Results

No.	Peak Name	Retention Time min	Area mAU*min	Height mAU	Relative Area %	Relative Height %	Amount n.a.
1		16.050	3.555	8.465	11.66	16.40	n.a.
2		16.443	3.915	8.336	12.84	16.15	n.a.
3		22.533	11.504	18.294	37.72	35.44	n.a.
4		24.537	11.522	16.529	37.78	32.02	n.a.
**Total:**			**30.497**	**51.625**	**100.00**	**100.00**	

Chromatogram

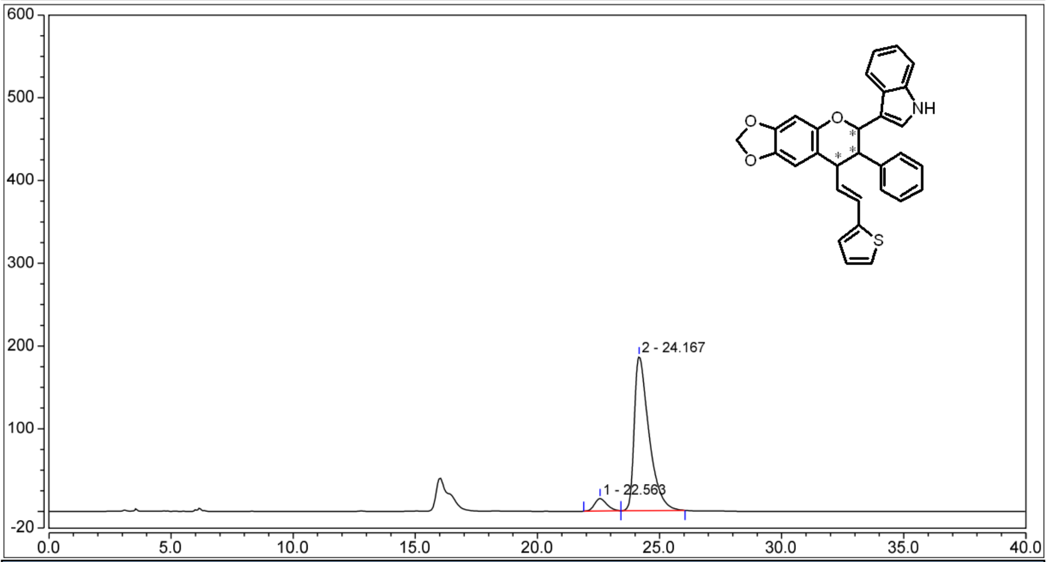

Integration Results

No.	Peak Name	Retention Time min	Area mAU*min	Height mAU	Relative Area %	Relative Height %	Amount n.a.
1		22.563	8.739	15.012	6.29	7.46	n.a.
2		24.167	130.183	186.162	93.71	92.54	n.a.
**Total:**			**138.923**	**201.174**	**100.00**	**100.00**	

4. HPLC spectra of products 6

6aa:

Chromatogram

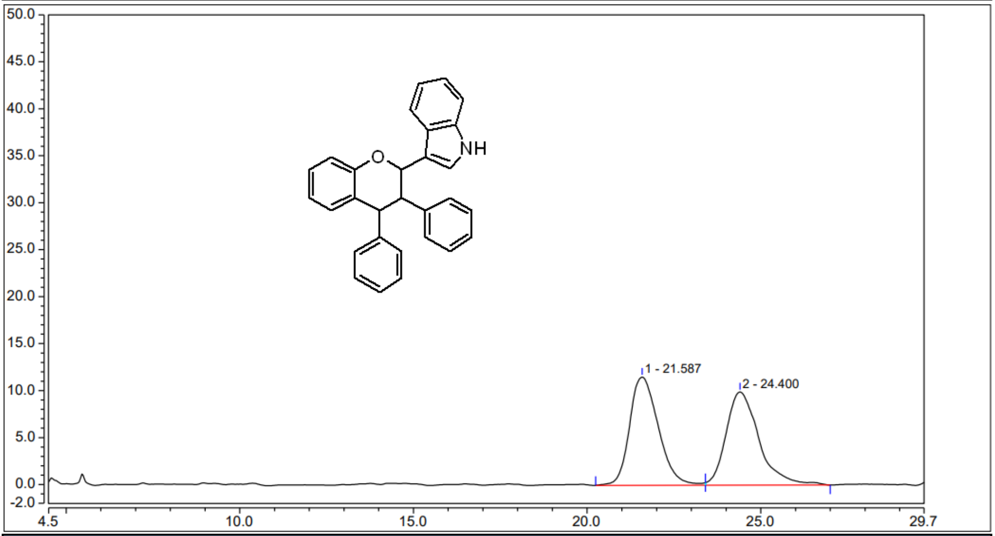

Integration Results

No.	Peak Name	Retention Time min	Area mAU*min	Height mAU	Relative Area %	Relative Height %	Amount n.a.
1		21.587	11.083	11.512	50.42	53.73	n.a.
2		24.400	10.899	9.914	49.58	46.27	n.a.
**Total:**			**21.982**	**21.426**	**100.00**	**100.00**	

Chromatogram

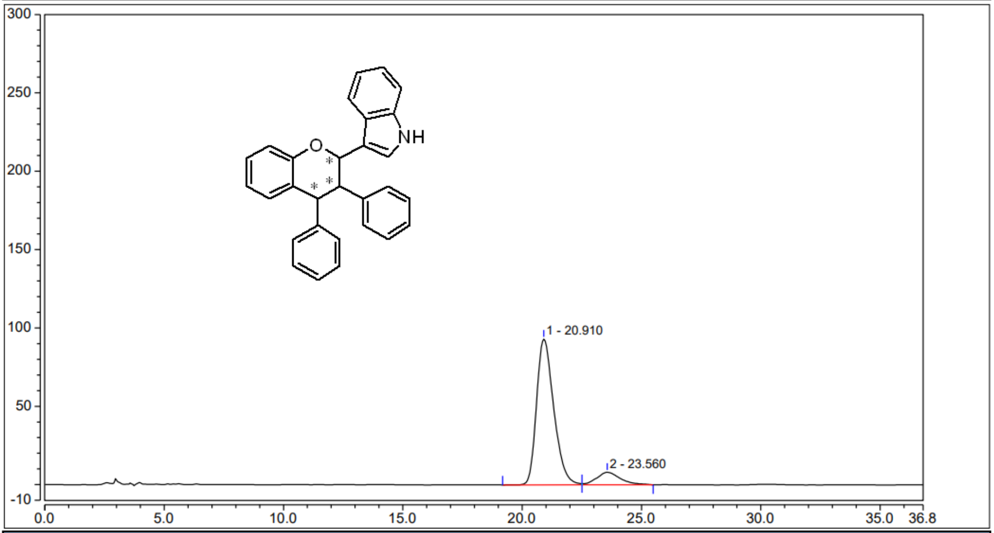

Integration Results

No.	Peak Name	Retention Time min	Area mAU*min	Height mAU	Relative Area %	Relative Height %	Amount n.a.
1		20.910	77.828	93.051	89.42	92.10	n.a.
2		23.560	9.212	7.984	10.58	7.90	n.a.
**Total:**			**87.040**	**101.035**	**100.00**	**100.00**	

6ba:

Chromatogram

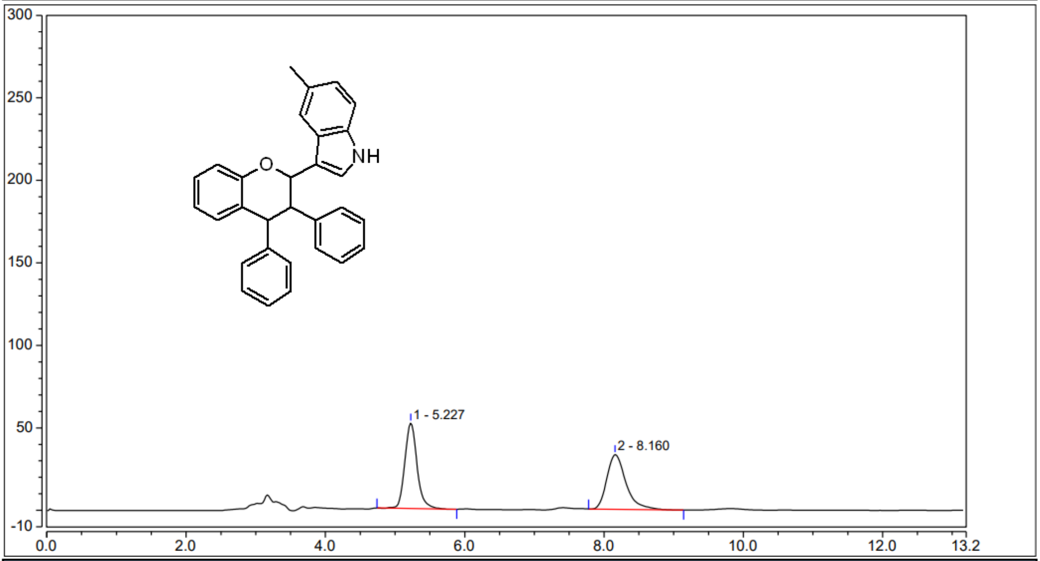

Integration Results

No.	Peak Name	Retention Time min	Area mAU*min	Height mAU	Relative Area %	Relative Height %	Amount n.a.
1		5.227	10.568	51.806	49.97	60.98	n.a.
2		8.160	10.581	33.144	50.03	39.02	n.a.
**Total:**			**21.150**	**84.950**	**100.00**	**100.00**	

Chromatogram

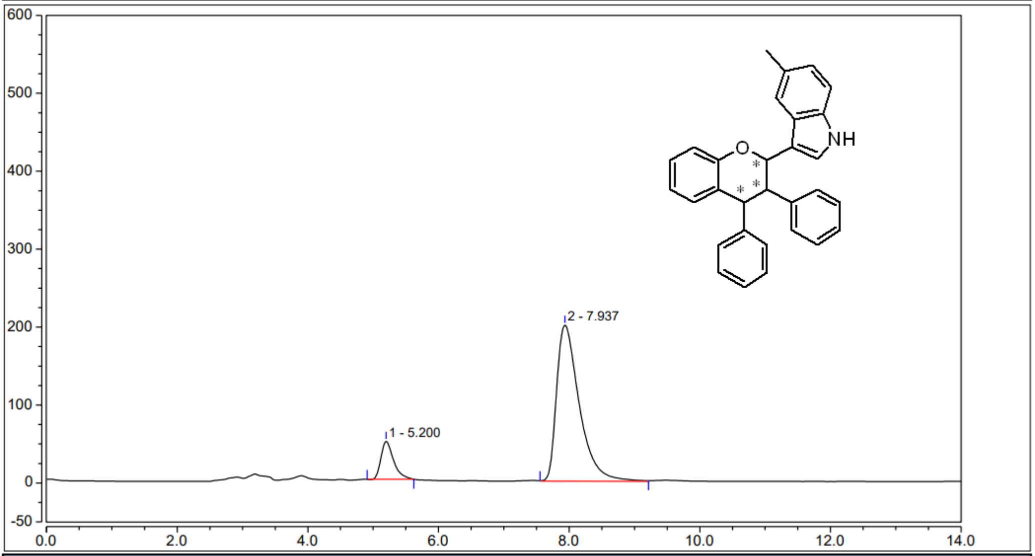

Integration Results

No.	Peak Name	Retention Time min	Area mAU*min	Height mAU	Relative Area %	Relative Height %	Amount n.a.
1		5.200	10.880	48.692	11.84	19.56	n.a.
2		7.937	80.999	200.275	88.16	80.44	n.a.
**Total:**			**91.879**	**248.968**	**100.00**	**100.00**	

6pa:

Chromatogram

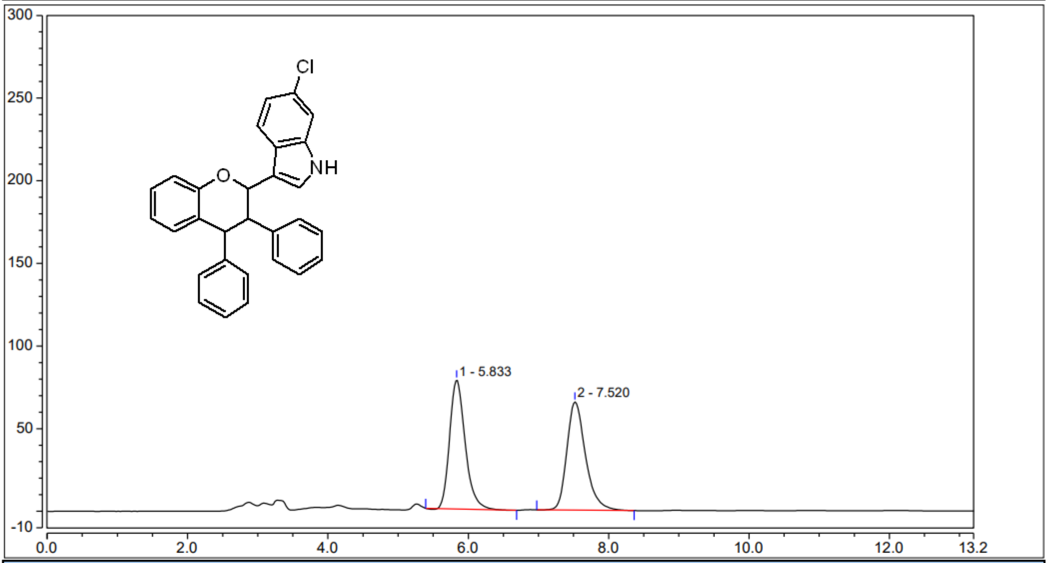

Integration Results

No.	Peak Name	Retention Time min	Area mAU*min	Height mAU	Relative Area %	Relative Height %	Amount n.a.
1		5.833	20.100	78.087	49.82	54.38	n.a.
2		7.520	20.246	65.501	50.18	45.62	n.a.
**Total:**			**40.346**	**143.589**	**100.00**	**100.00**	

Chromatogram

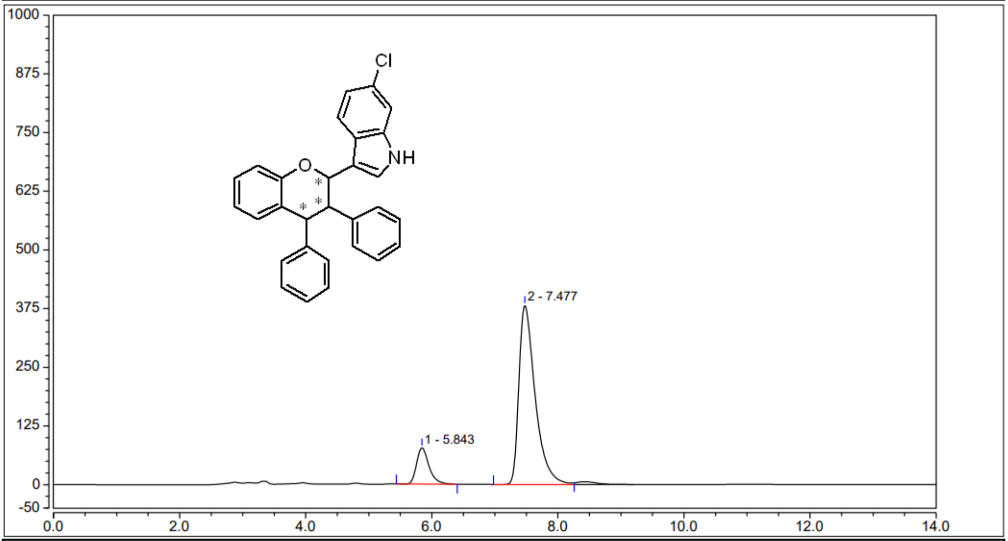

Integration Results

No.	Peak Name	Retention Time min	Area mAU*min	Height mAU	Relative Area %	Relative Height %	Amount n.a.
1		5.843	17.457	77.100	13.25	16.81	n.a.
2		7.477	114.263	381.586	86.75	83.19	n.a.
**Total:**			**131.720**	**458.686**	**100.00**	**100.00**	

6da:

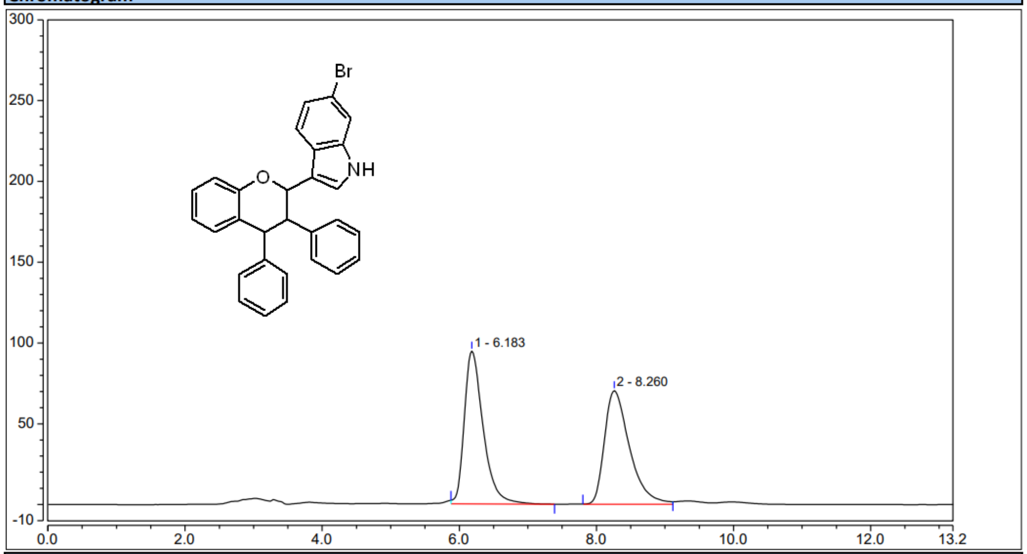

Integration Results

No.	Peak Name	Retention Time min	Area mAU*min	Height mAU	Relative Area %	Relative Height %	Amount n.a.
1		6.183	30.254	94.566	50.34	57.37	n.a.
2		8.260	29.848	70.271	49.66	42.63	n.a.
**Total:**			**60.102**	**164.837**	**100.00**	**100.00**	

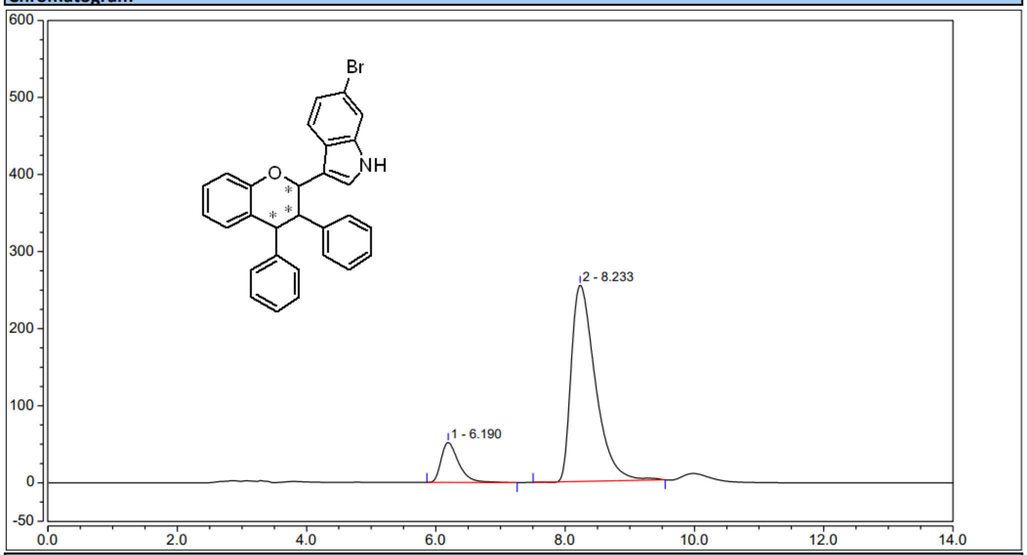

Integration Results

No.	Peak Name	Retention Time min	Area mAU*min	Height mAU	Relative Area %	Relative Height %	Amount n.a.
1		6.190	16.370	51.892	12.93	16.93	n.a.
2		8.233	110.266	254.623	87.07	83.07	n.a.
**Total:**			**126.636**	**306.515**	**100.00**	**100.00**	

6ea:

Chromatogram

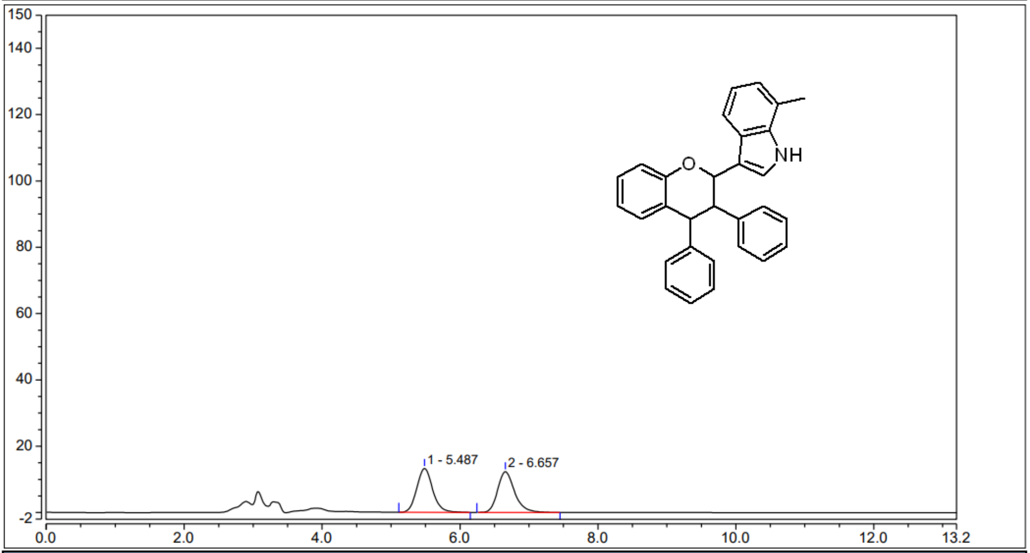

Integration Results

No.	Peak Name	Retention Time<br>min	Area<br>mAU*min	Height<br>mAU	Relative Area<br>%	Relative Height<br>%	Amount<br>n.a.
1		5.487	3.551	13.234	50.23	51.82	n.a.
2		6.657	3.518	12.303	49.77	48.18	n.a.
**Total:**			**7.069**	**25.537**	**100.00**	**100.00**	

Chromatogram

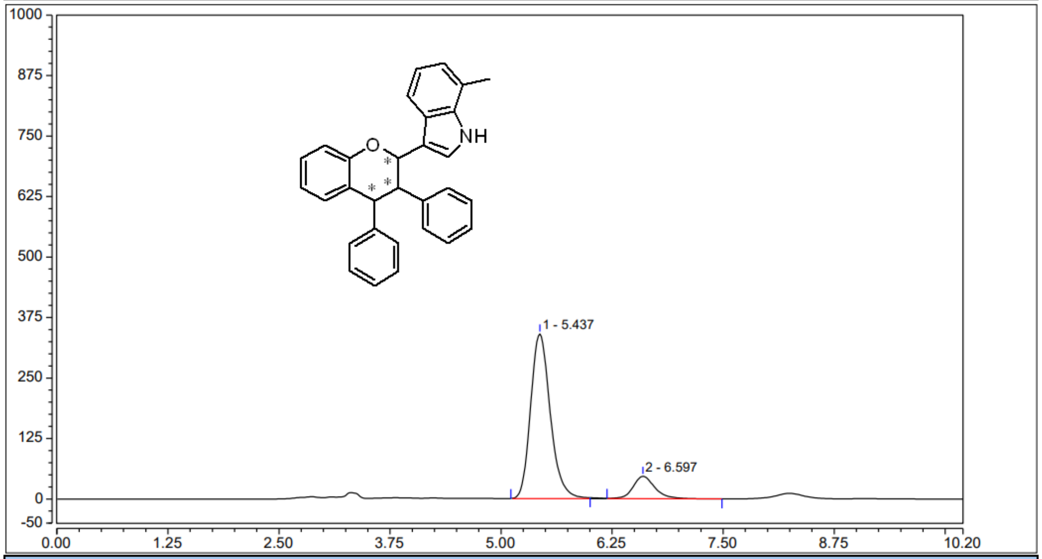

Integration Results

No.	Peak Name	Retention Time<br>min	Area<br>mAU*min	Height<br>mAU	Relative Area<br>%	Relative Height<br>%	Amount<br>n.a.
1		5.437	87.596	340.422	86.79	87.98	n.a.
2		6.597	13.334	46.495	13.21	12.02	n.a.
**Total:**			**100.930**	**386.917**	**100.00**	**100.00**	

6qa:

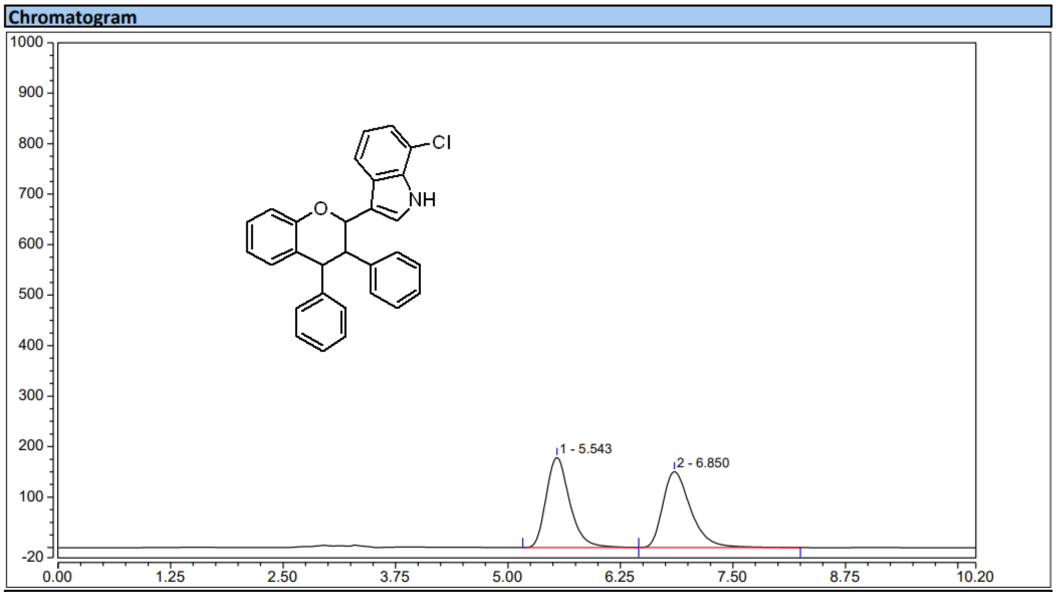

Integration Results

No.	Peak Name	Retention Time min	Area mAU*min	Height mAU	Relative Area %	Relative Height %	Amount n.a.
1		5.543	54.260	178.256	49.94	54.28	n.a.
2		6.850	54.398	150.175	50.06	45.72	n.a.
**Total:**			**108.658**	**328.431**	**100.00**	**100.00**	

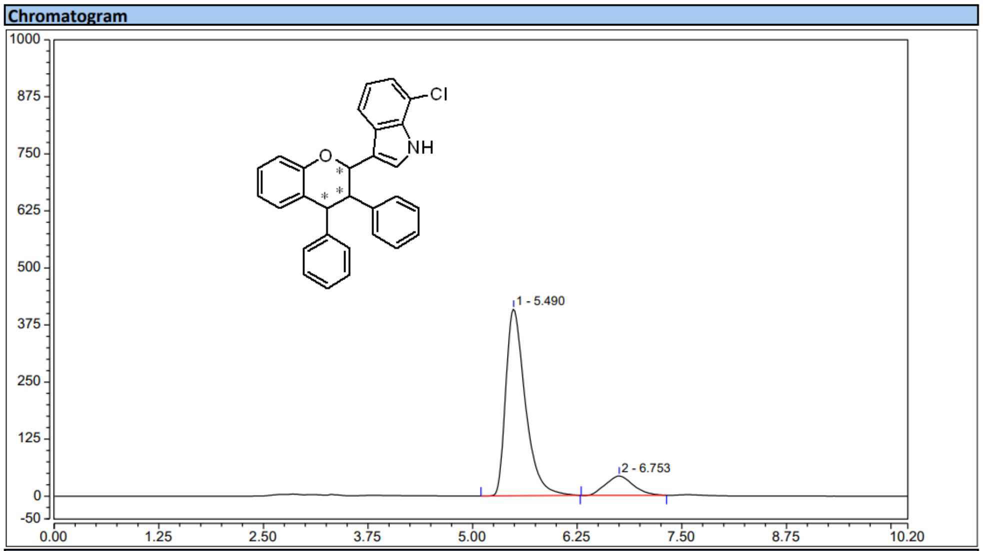

Integration Results

No.	Peak Name	Retention Time min	Area mAU*min	Height mAU	Relative Area %	Relative Height %	Amount n.a.
1		5.490	110.990	408.659	87.26	90.62	n.a.
2		6.753	16.202	42.307	12.74	9.38	n.a.
**Total:**			**127.192**	**450.966**	**100.00**	**100.00**	

6ra:

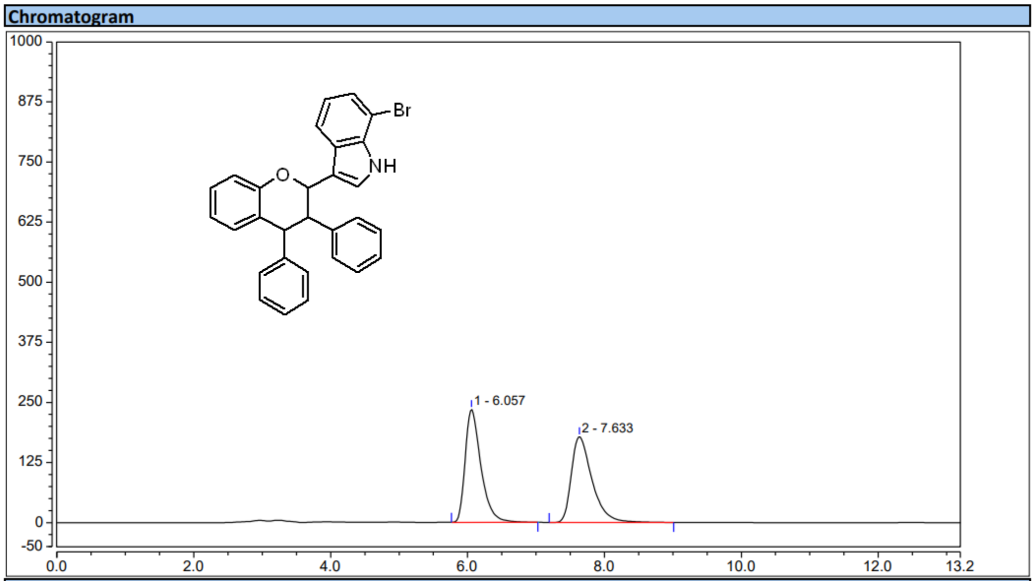

Integration Results

No.	Peak Name	Retention Time<br>min	Area<br>mAU*min	Height<br>mAU	Relative Area<br>%	Relative Height<br>%	Amount<br>n.a.
1		6.057	62.734	234.778	50.05	56.85	n.a.
2		7.633	62.606	178.228	49.95	43.15	n.a.
**Total:**			**125.340**	**413.006**	**100.00**	**100.00**	

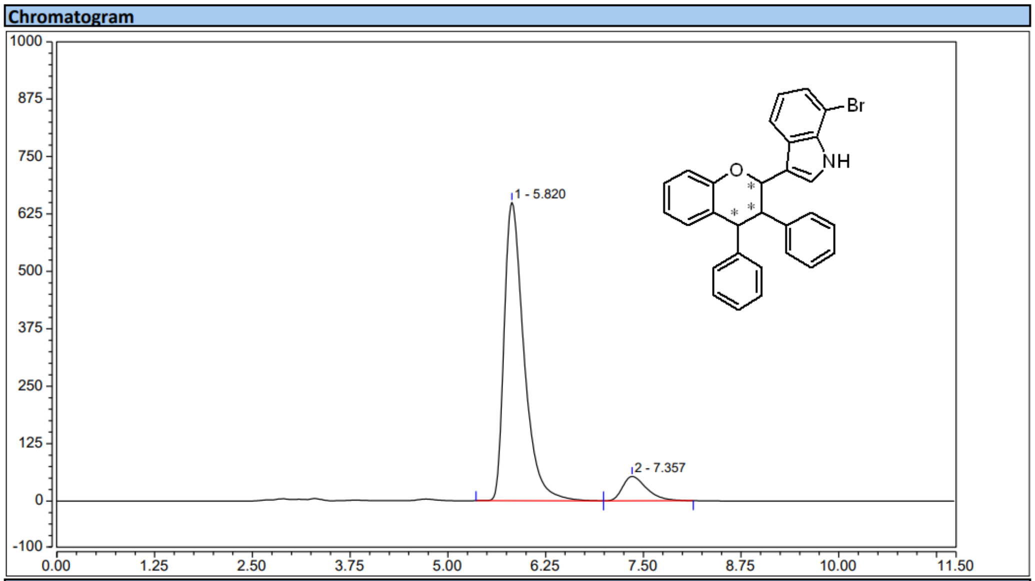

Integration Results

No.	Peak Name	Retention Time<br>min	Area<br>mAU*min	Height<br>mAU	Relative Area<br>%	Relative Height<br>%	Amount<br>n.a.
1		5.820	190.938	649.368	91.02	92.45	n.a.
2		7.357	18.845	53.048	8.98	7.55	n.a.
**Total:**			**209.783**	**702.416**	**100.00**	**100.00**	

6ka:

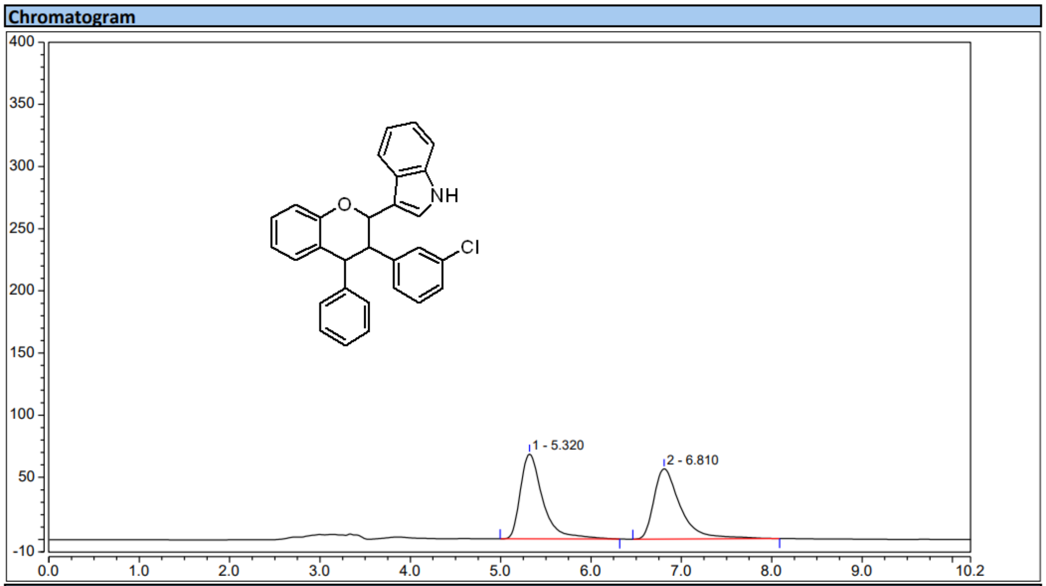

Integration Results

No.	Peak Name	Retention Time min	Area mAU*min	Height mAU	Relative Area %	Relative Height %	Amount n.a.
1		5.320	19.577	68.160	50.47	54.64	n.a.
2		6.810	19.215	56.582	49.53	45.36	n.a.
**Total:**			**38.792**	**124.742**	**100.00**	**100.00**	

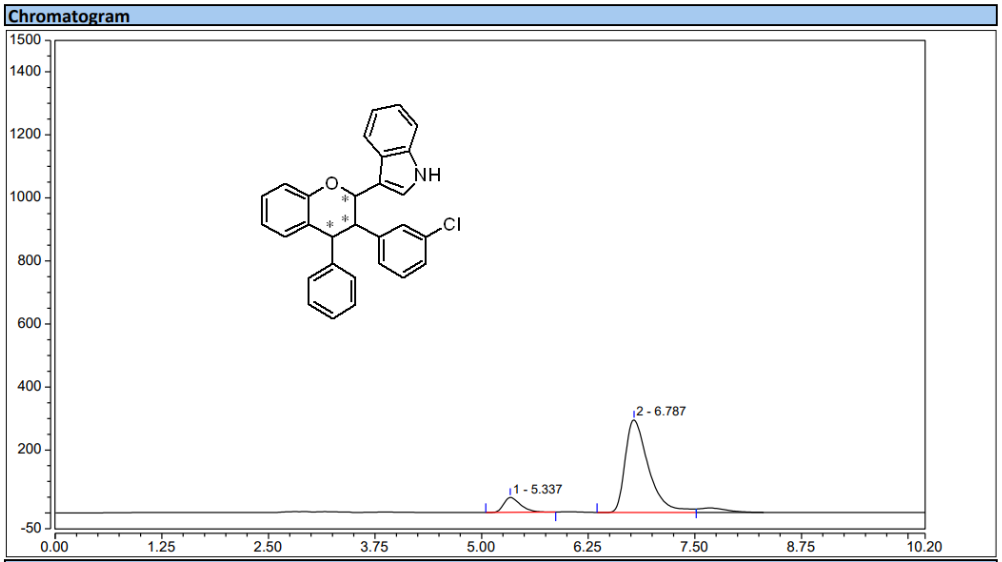

Integration Results

No.	Peak Name	Retention Time min	Area mAU*min	Height mAU	Relative Area %	Relative Height %	Amount n.a.
1		5.337	10.662	47.009	10.23	13.81	n.a.
2		6.787	93.541	293.499	89.77	86.19	n.a.
**Total:**			**104.202**	**340.508**	**100.00**	**100.00**	

6ma:

Chromatogram

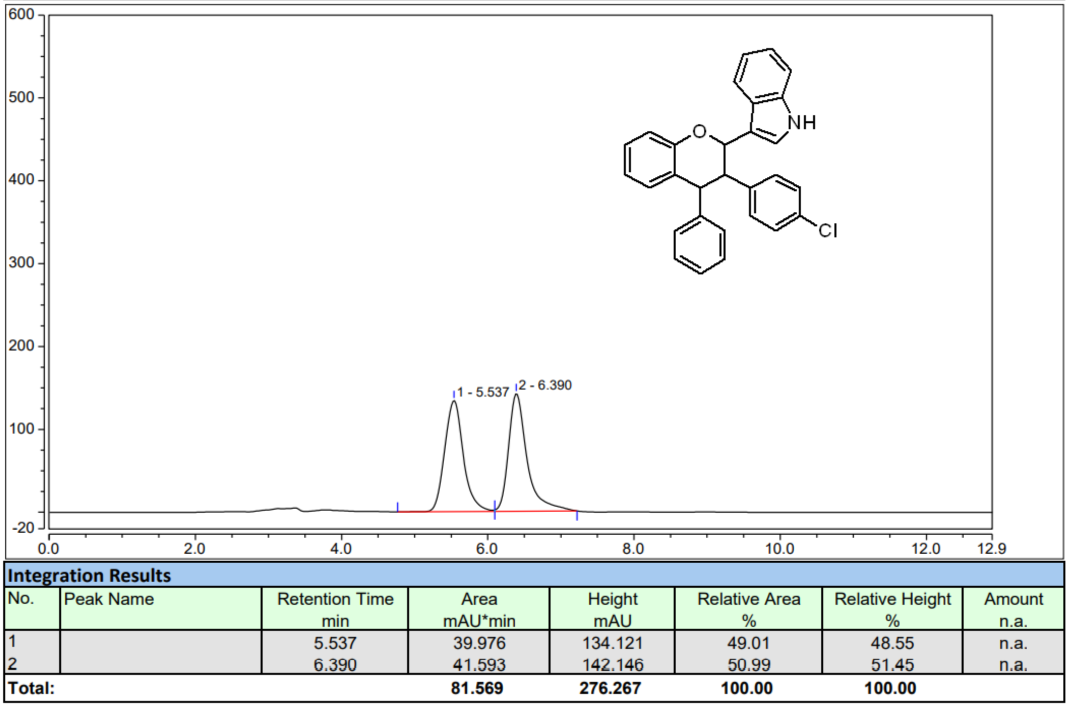

Integration Results

No.	Peak Name	Retention Time min	Area mAU*min	Height mAU	Relative Area %	Relative Height %	Amount n.a.
1		5.537	39.976	134.121	49.01	48.55	n.a.
2		6.390	41.593	142.146	50.99	51.45	n.a.
**Total:**			**81.569**	**276.267**	**100.00**	**100.00**	

Chromatogram

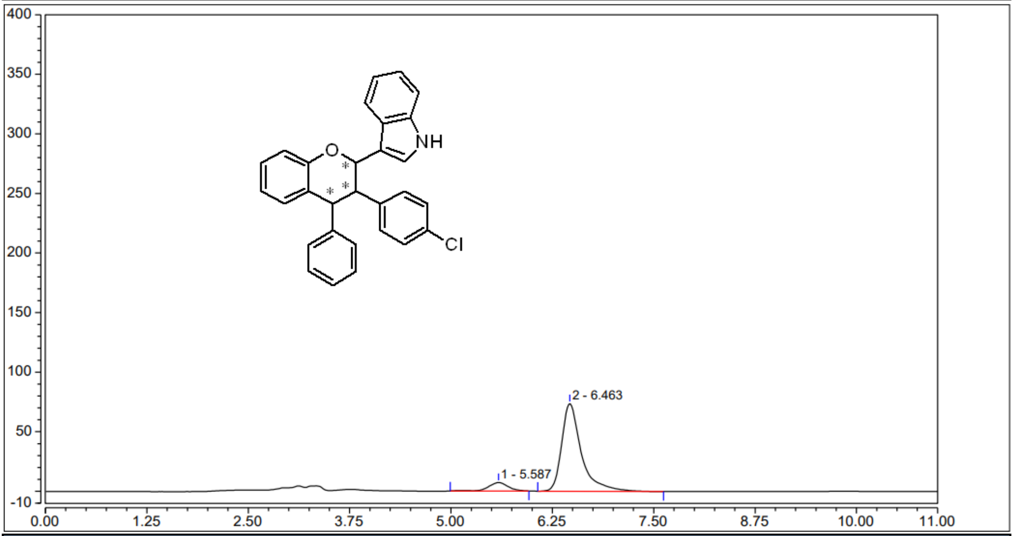

Integration Results

No.	Peak Name	Retention Time min	Area mAU*min	Height mAU	Relative Area %	Relative Height %	Amount n.a.
1		5.587	1.976	7.044	8.71	8.74	n.a.
2		6.463	20.717	73.532	91.29	91.26	n.a.
**Total:**			**22.693**	**80.575**	**100.00**	**100.00**	

6ab:

Chromatogram

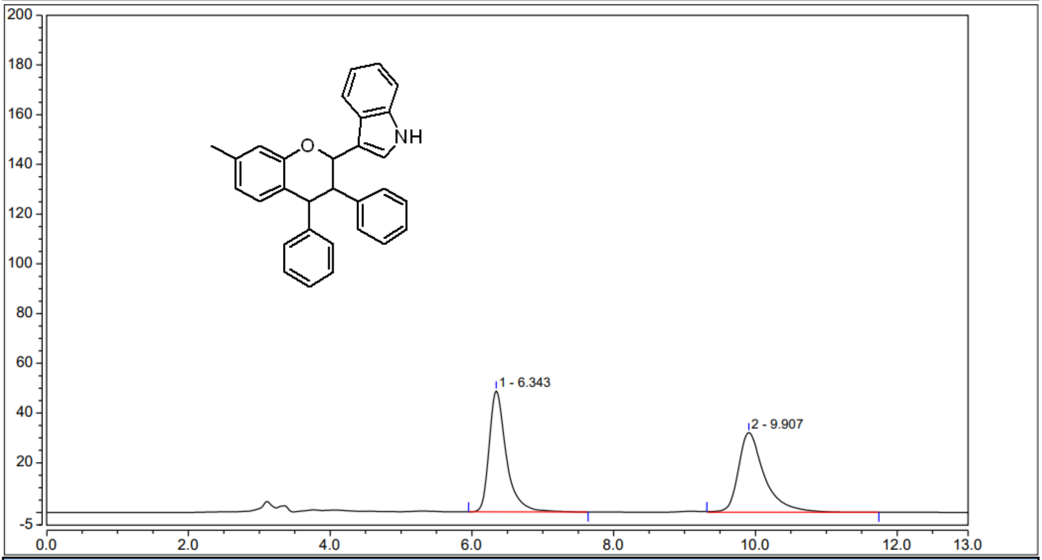

Integration Results

No.	Peak Name	Retention Time min	Area mAU*min	Height mAU	Relative Area %	Relative Height %	Amount n.a.
1		6.343	13.644	48.701	49.91	60.29	n.a.
2		9.907	13.693	32.077	50.09	39.71	n.a.
Total:			27.337	80.778	100.00	100.00	

Chromatogram

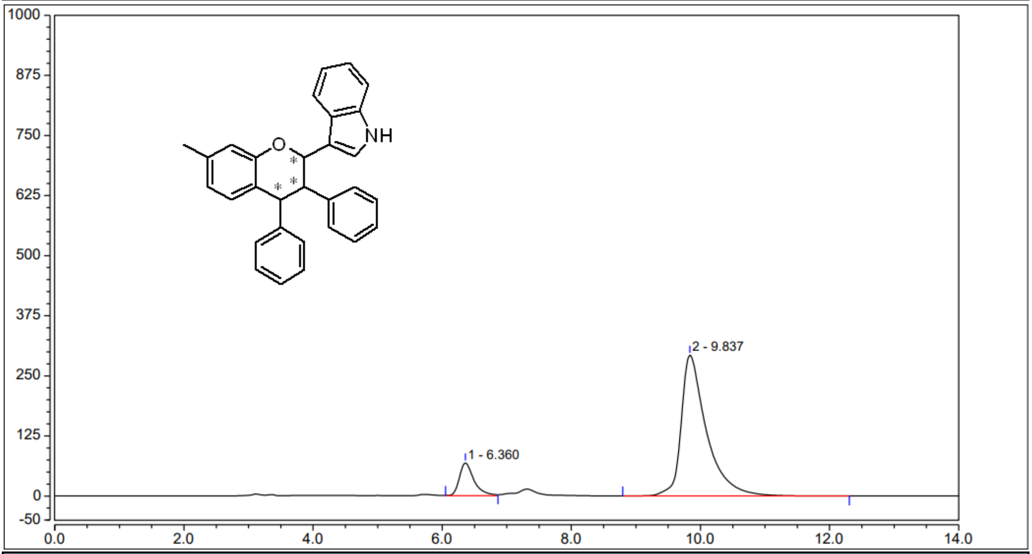

Integration Results

No.	Peak Name	Retention Time min	Area mAU*min	Height mAU	Relative Area %	Relative Height %	Amount n.a.
1		6.360	17.916	68.228	11.85	18.89	n.a.
2		9.837	133.271	292.942	88.15	81.11	n.a.
Total:			151.187	361.170	100.00	100.00	

6ac (major diastereoisomer)**:**

Chromatogram

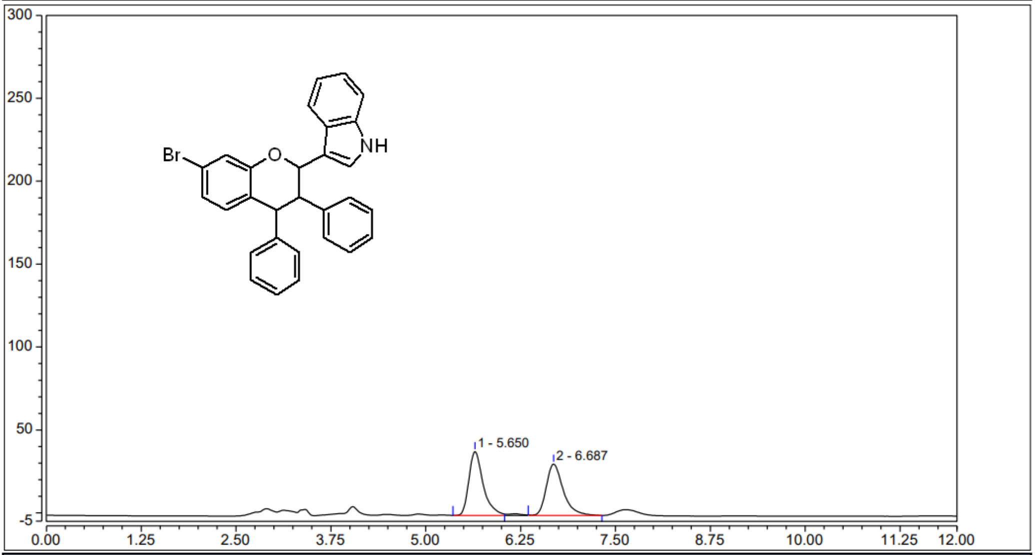

Integration Results

No.	Peak Name	Retention Time min	Area mAU*min	Height mAU	Relative Area %	Relative Height %	Amount n.a.
1		5.650	8.439	38.485	51.30	55.40	n.a.
2		6.687	8.010	30.976	48.70	44.60	n.a.
**Total:**			**16.449**	**69.461**	**100.00**	**100.00**	

Chromatogram

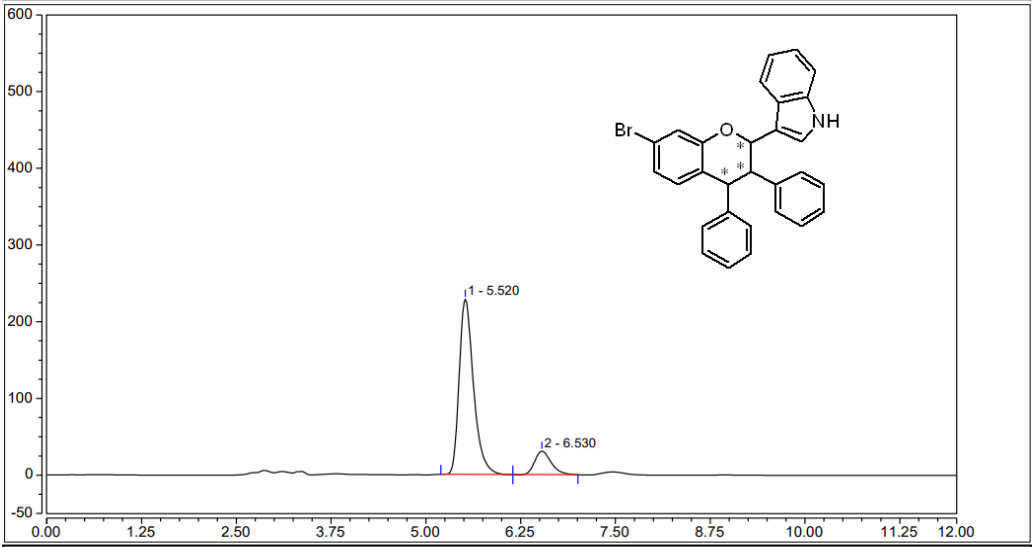

Integration Results

No.	Peak Name	Retention Time min	Area mAU*min	Height mAU	Relative Area %	Relative Height %	Amount n.a.
1		5.520	49.935	228.514	86.67	88.20	n.a.
2		6.530	7.678	30.560	13.33	11.80	n.a.
**Total:**			**57.613**	**259.073**	**100.00**	**100.00**	

6ac (minor diastereoisomer)**:**

Chromatogram

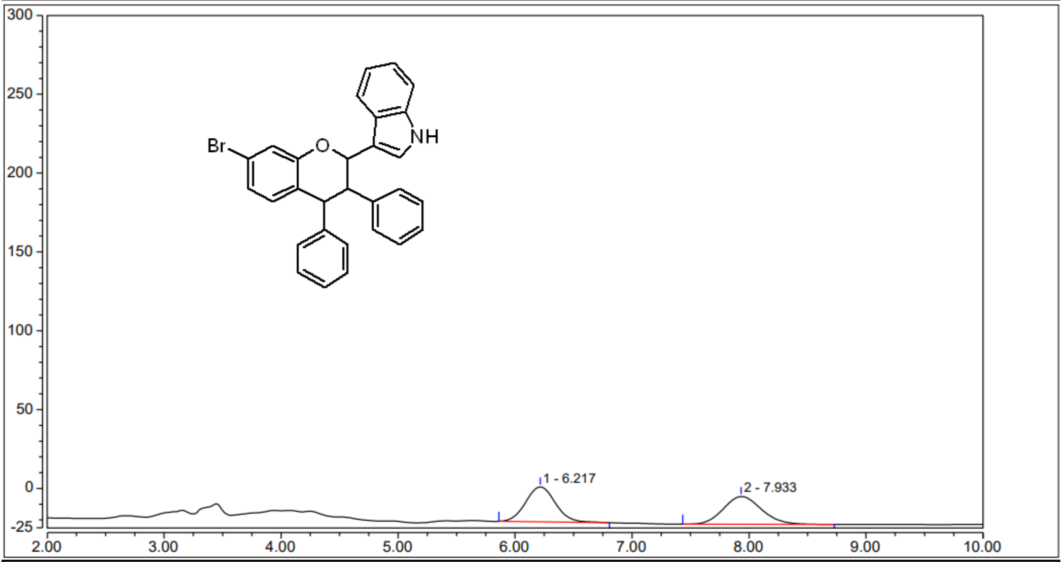

Integration Results

No.	Peak Name	Retention Time min	Area mAU*min	Height mAU	Relative Area %	Relative Height %	Amount n.a.
1		6.217	6.530	22.114	49.44	55.62	n.a.
2		7.933	6.678	17.645	50.56	44.38	n.a.
**Total:**			**13.208**	**39.759**	**100.00**	**100.00**	

Chromatogram

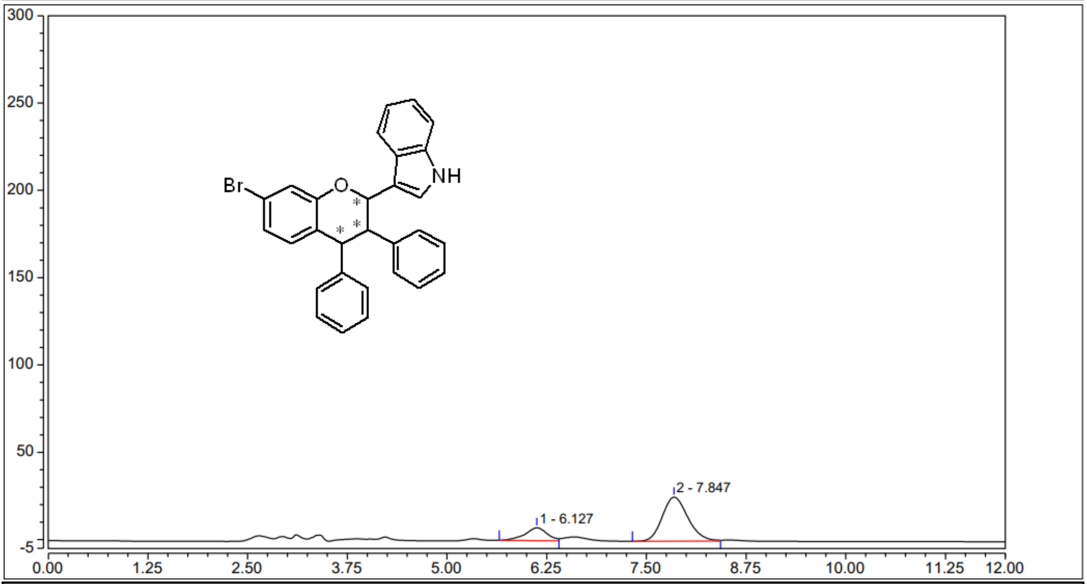

Integration Results

No.	Peak Name	Retention Time min	Area mAU*min	Height mAU	Relative Area %	Relative Height %	Amount n.a.
1		6.127	2.336	7.329	19.89	22.53	n.a.
2		7.847	9.410	25.202	80.11	77.47	n.a.
**Total:**			**11.746**	**32.531**	**100.00**	**100.00**	

6ad:

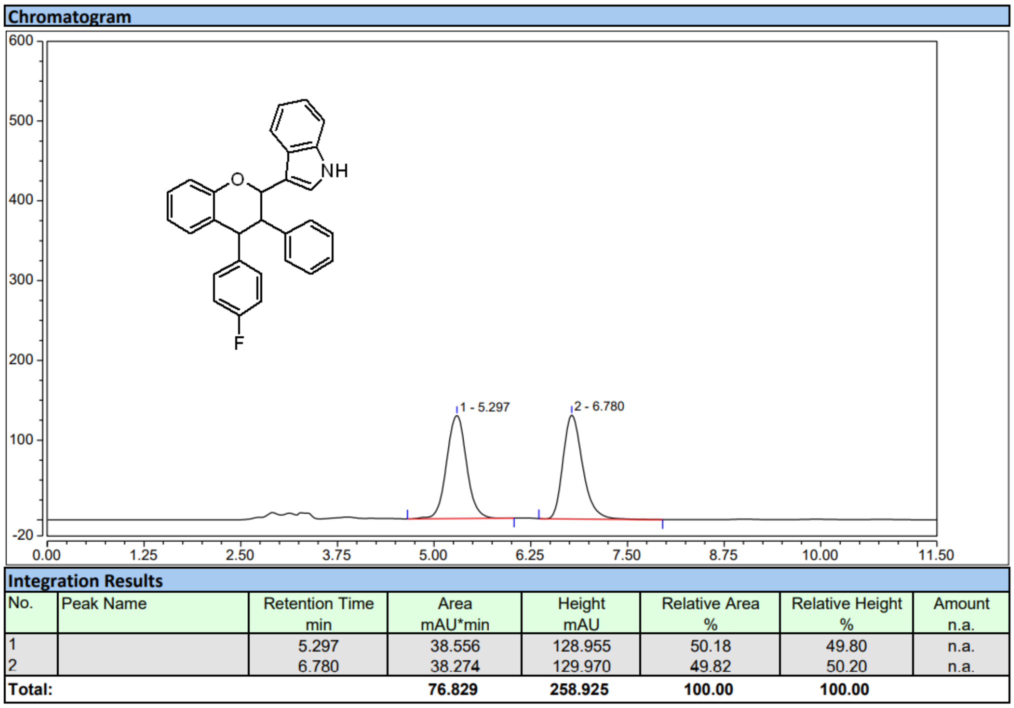

Integration Results

No.	Peak Name	Retention Time min	Area mAU*min	Height mAU	Relative Area %	Relative Height %	Amount n.a.
1		5.297	38.556	128.955	50.18	49.80	n.a.
2		6.780	38.274	129.970	49.82	50.20	n.a.
**Total:**			**76.829**	**258.925**	**100.00**	**100.00**	

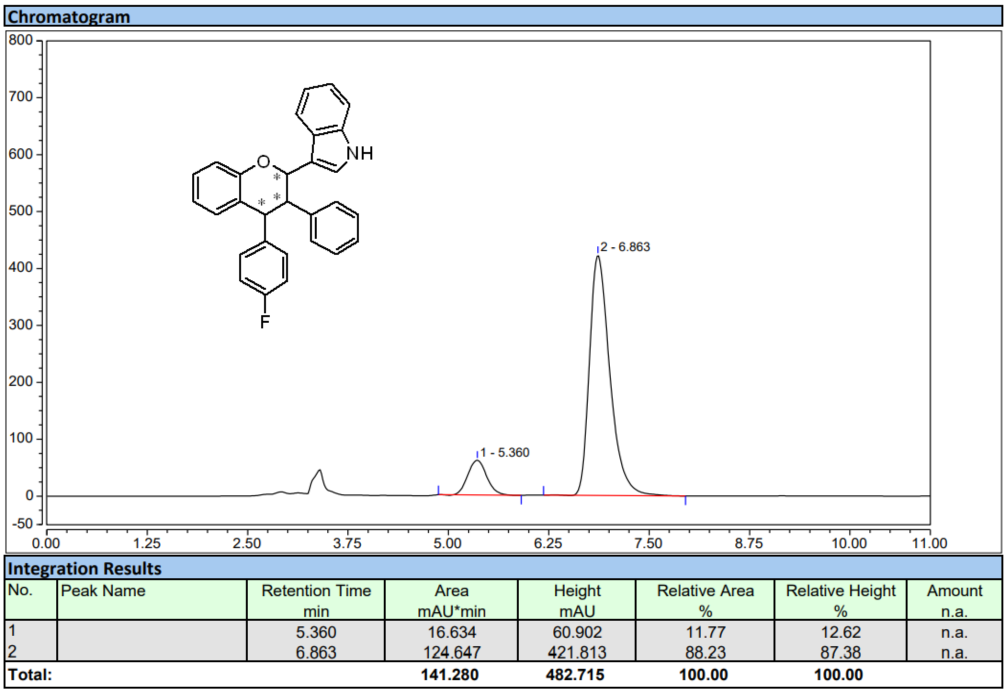

Integration Results

No.	Peak Name	Retention Time min	Area mAU*min	Height mAU	Relative Area %	Relative Height %	Amount n.a.
1		5.360	16.634	60.902	11.77	12.62	n.a.
2		6.863	124.647	421.813	88.23	87.38	n.a.
**Total:**			**141.280**	**482.715**	**100.00**	**100.00**	

6ae:

Chromatogram

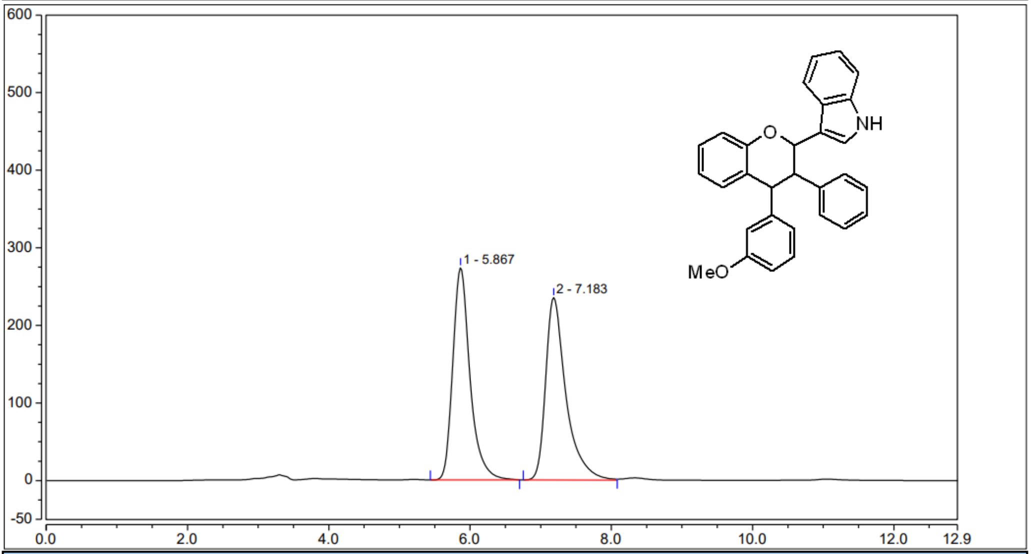

Integration Results

No.	Peak Name	Retention Time min	Area mAU*min	Height mAU	Relative Area %	Relative Height %	Amount n.a.
1		5.867	77.268	273.553	49.46	53.77	n.a.
2		7.183	78.960	235.181	50.54	46.23	n.a.
**Total:**			**156.227**	**508.734**	**100.00**	**100.00**	

Chromatogram

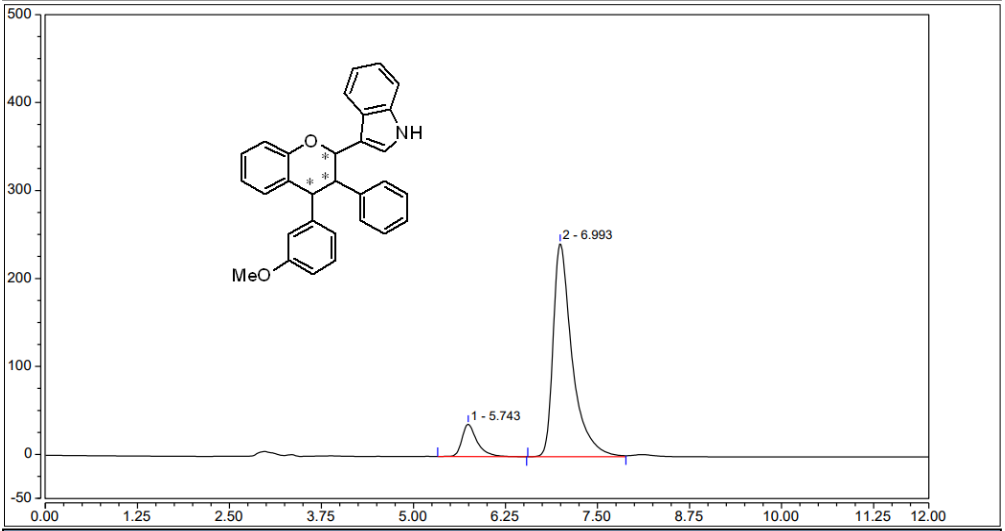

Integration Results

No.	Peak Name	Retention Time min	Area mAU*min	Height mAU	Relative Area %	Relative Height %	Amount n.a.
1		5.743	8.741	36.581	10.72	13.11	n.a.
2		6.993	72.793	242.428	89.28	86.89	n.a.
**Total:**			**81.534**	**279.008**	**100.00**	**100.00**	

6af:

Chromatogram

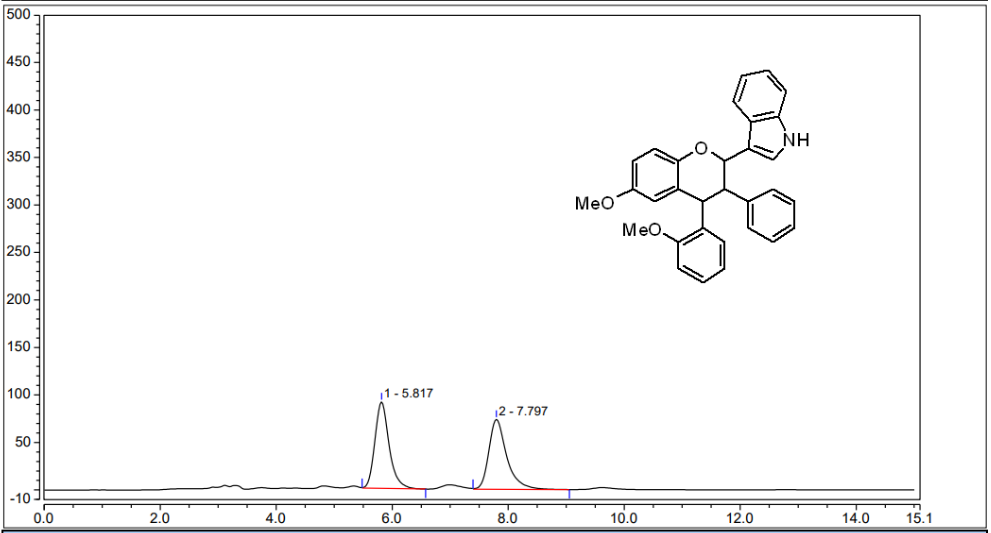

Integration Results

No.	Peak Name	Retention Time<br>min	Area<br>mAU*min	Height<br>mAU	Relative Area<br>%	Relative Height<br>%	Amount<br>n.a.
1		5.817	25.898	90.973	49.67	55.28	n.a.
2		7.797	26.245	73.607	50.33	44.72	n.a.
**Total:**			**52.144**	**164.580**	**100.00**	**100.00**	

Chromatogram

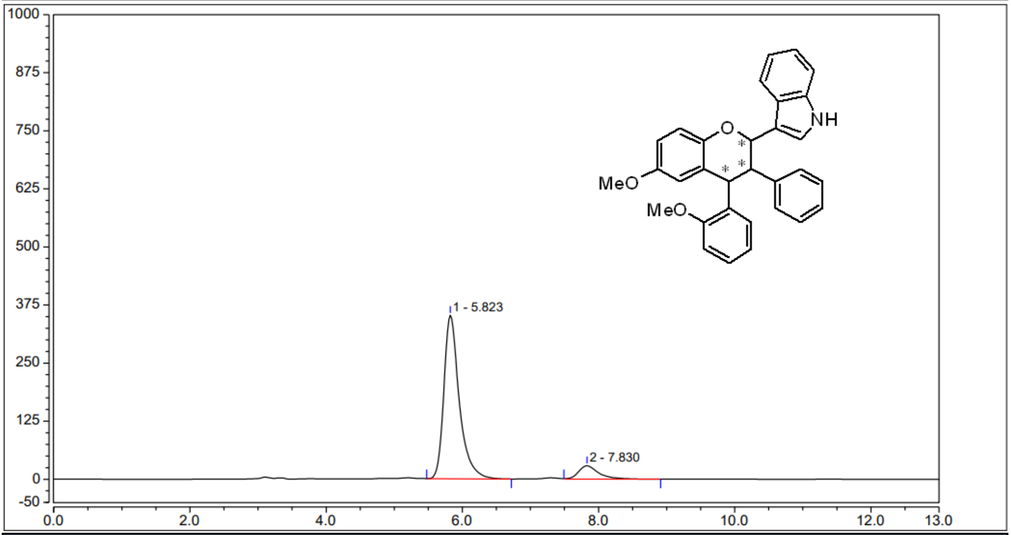

Integration Results

No.	Peak Name	Retention Time<br>min	Area<br>mAU*min	Height<br>mAU	Relative Area<br>%	Relative Height<br>%	Amount<br>n.a.
1		5.823	92.066	351.740	90.26	92.45	n.a.
2		7.830	9.940	28.733	9.74	7.55	n.a.
**Total:**			**102.006**	**380.473**	**100.00**	**100.00**	

5. NOE spectrum of product 6ma

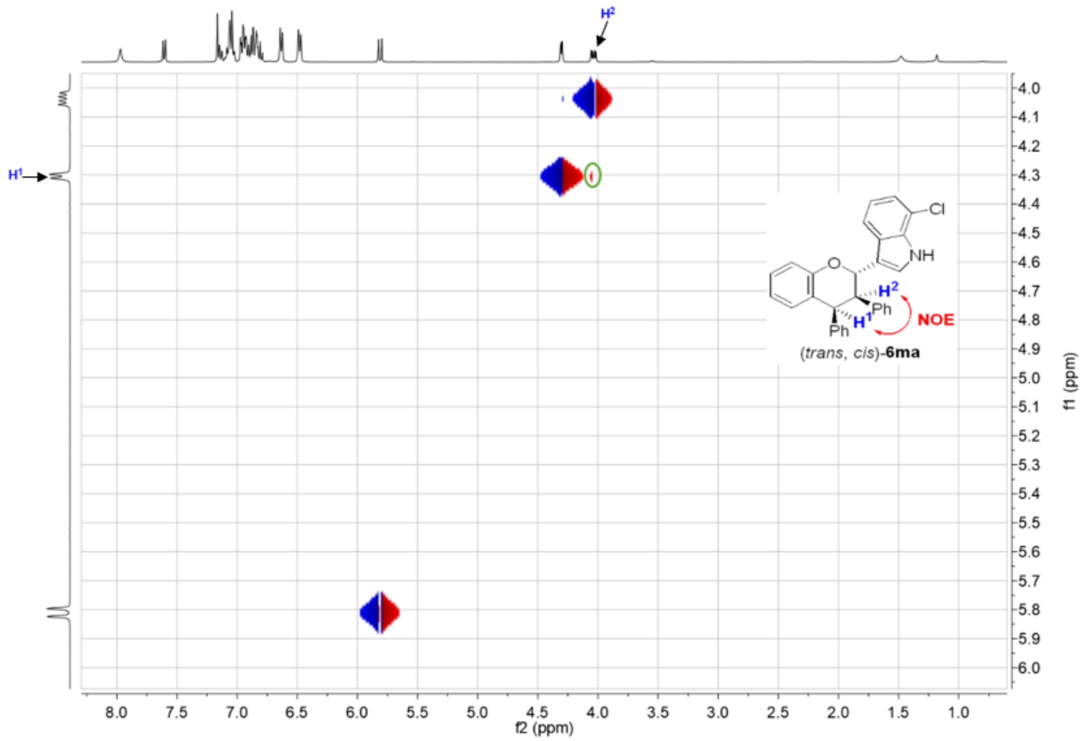

6. X-ray single-crystal data for product 3na

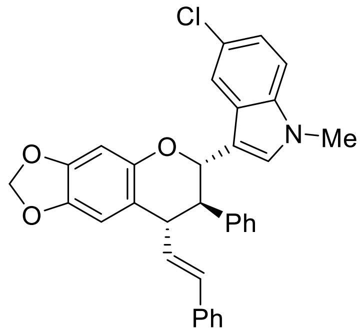

(*trans*, *trans*)-**3na**

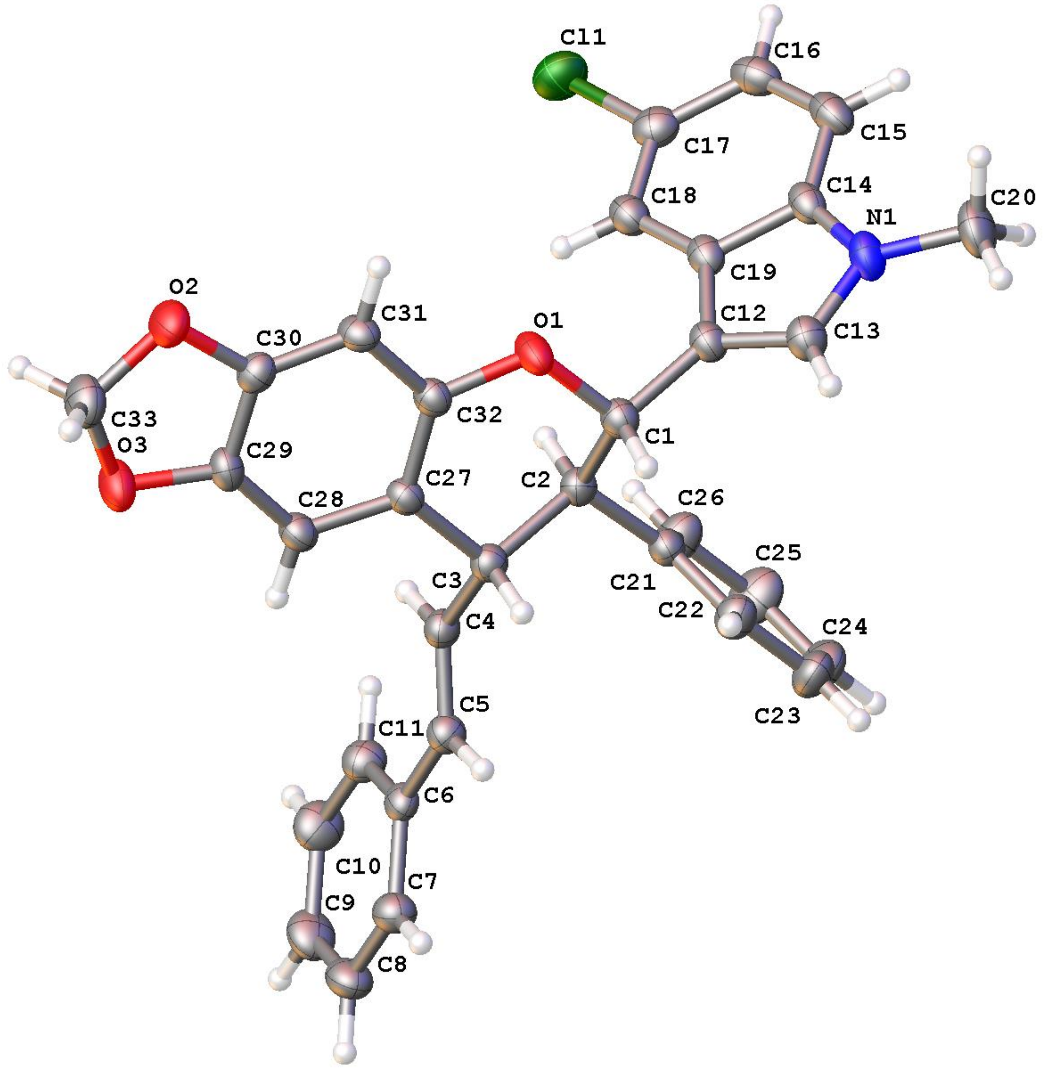

The X-ray source used for the single crystal X-ray diffraction analysis of compound **3na** was MoKα (λ = 0.71073), and the thermal ellipsoid was drawn at the 30% probability level.

Empirical formula	$C_{33}H_{26}ClNO_3$
Formula weight	520.00
Temperature/K	296.15
Crystal system	triclinic
Space group	P-1
a/Å	8.5541(14)
b/Å	12.401(2)
c/Å	15.137(2)
α/°	68.756(2)
β/°	75.804(2)
γ/°	88.195(2)
Volume/Å^3	1448.0(4)
Z	2
ρ_{calc}g/cm^3	1.193
μ/mm^{-1}	0.164
F(000)	544.0
Radiation	MoKα (λ = 0.71073)
2Θ range for data collection/°	5.392 to 51.362
Index ranges	$-10 \leq h \leq 9$, $-6 \leq k \leq 15$, $-17 \leq l \leq 18$
Reflections collected	7706
Independent reflections	5390 [R_{int} = 0.0154, R_{sigma} = 0.0352]
Data/restraints/parameters	5390/0/344
Goodness-of-fit on F^2	1.058
Final R indexes [I>=2σ (I)]	R_1 = 0.0470, wR_2 = 0.1217
Final R indexes [all data]	R_1 = 0.0689, wR_2 = 0.1344
Largest diff. peak/hole / e Å^{-3}	0.23/-0.25

7. Theoretical calculations of the reaction pathway

Computational details:

All calculations were performed using Gaussian 16, Revision A.03 package.[1] All of the reactants, intermediates, transition states and products were optimized by the DFT with the B3LYP functional. For geometry optimizations and frequency calculations, BS-I basis set system was employed. In BS-I, we employed 6-31G(d) basis sets for H, C, O, N, P, and Cl. All the stationary structures were characterized with no imaginary frequency and the transition state structures (TSs) were characterized with a single imaginary frequency. Intrinsic reaction coordinate (IRC) calculations were performed on all the TSs. The solvent effect in toluene was evaluated by B3LYP-D3 functional through the SMD[2] model, in which a better basis system BS-II was used. In BS-II, we employed 6-311++G(d,p) basis sets for the atoms. All the 3D molecular structures of the species were generated by using the CYLview program.[3]

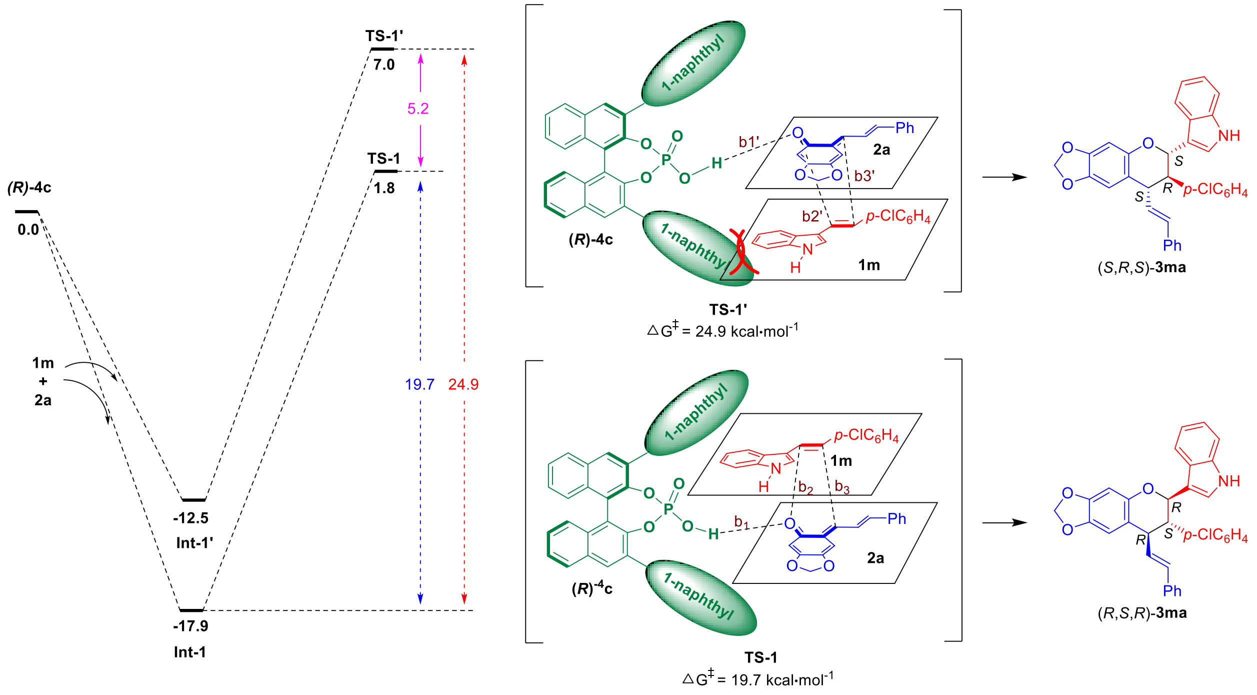

Figure S1. DFT calculated Gibbs free energy profiles for the cyclization process (in kcal/mol).

---

[1] Gaussian 16, Revision A.03, M. J. Frisch, G. W. Trucks, H. B. Schlegel, G. E. Scuseria, M. A. Robb, J. R. Cheeseman, G. Scalmani, V. Barone, G. A. Petersson, H. Nakatsuji, X. Li, M. Caricato, A. V. Marenich, J. Bloino, B. G. Janesko, R. Gomperts, B. Mennucci, H. P. Hratchian, J. V. Ortiz, A. F. Izmaylov, J. L. Sonnenberg, D. Williams-Young, F. Ding, F. Lipparini, F. Egidi, J. Goings, B. Peng, A. Petrone, T. Henderson, D. Ranasinghe, V. G. Zakrzewski, J. Gao, N. Rega, G. Zheng, W. Liang, M. Hada, M. Ehara, K. Toyota, R. Fukuda, J. Hasegawa, M. Ishida, T. Nakajima, Y. Honda, O. Kitao, H. Nakai, T. Vreven, K. Throssell, J. A. Montgomery, Jr., J. E. Peralta, F. Ogliaro, M. J. Bearpark, J. J. Heyd, E. N. Brothers, K. N. Kudin, V. N. Staroverov, T. A. Keith, R. Kobayashi, J. Normand, K. Raghavachari, A. P. Rendell, J. C. Burant, S. S. Iyengar, J. Tomasi, M. Cossi, J. M. Millam, M. Klene, C. Adamo, R. Cammi, J. W. Ochterski, R. L. Martin, K. Morokuma, O. Farkas, J. B. Foresman, and D. J. Fox, Gaussian, Inc., Wallingford CT, 2016.

[2] A. V. Marenich, C. J. Cramer, D. G. Truhlar, *J. Phys. Chem. B* **2009**, *113*, 6378.

[3] CYLview, 1.0b; C. Y. Legault, Universit édе Sherbrooke, **2009** (http://www.cylview.org).

Cartesian coordinates of the optimized structures:

***(R)*-4c**

E = -2181.658653 a.u.

C	0.67715700	1.96227100	-0.32689600
C	1.44491600	0.83662800	-0.06797600
C	2.80727600	0.70094300	-0.45641000
C	3.36456200	1.74101400	-1.17432300
C	2.61578100	2.88317200	-1.55269000
C	1.24791400	3.00050800	-1.14117400
C	-0.71586900	2.04262300	0.20276600
C	-1.61505000	1.02679800	-0.08019900
C	-2.97633800	1.03900500	0.33348500
C	-3.40096500	2.11041900	1.09165300
C	-2.51928800	3.14988900	1.48359000
C	-1.15222000	3.11759500	1.05378100
H	4.40738300	1.67764200	-1.47321000
H	-4.43769400	2.15212200	1.41511900
C	3.19544300	3.90843000	-2.34939000
C	2.45396000	4.99343800	-2.75524200
C	1.09190000	5.09234300	-2.38373000
C	0.50516400	4.12460400	-1.59820700
C	-0.27721500	4.13236200	1.53189000
C	-0.73679100	5.13880000	2.35304400
C	-2.09606900	5.18980300	2.74236100
C	-2.96462100	4.21121000	2.31765000
O	0.86832100	-0.21915200	0.64491500
O	-1.18818200	-0.06840200	-0.83040100
P	-0.19276600	-1.15758200	-0.14484900
O	0.28586800	-2.14595300	-1.12455600
O	-0.95664300	-1.73850100	1.14906700
H	-1.33616600	-2.60918600	0.94274400
C	3.62042100	-0.48649800	-0.06079300
C	4.22528800	-1.34031000	-1.04370300
C	3.83315500	-0.73809700	1.28374100
C	4.00578800	-1.19550100	-2.44230500
C	5.07378900	-2.41068400	-0.59909100
C	4.65865600	-1.80029300	1.71401200
C	4.61133000	-2.03653400	-3.34919600
H	3.33447000	-0.41975000	-2.79323800

C	5.68901200	-3.25462700	-1.56350700
C	5.27499400	-2.61304900	0.79140900
C	5.46989200	-3.07203900	-2.90917900
H	4.42053500	-1.91070000	-4.41153200
H	6.33427800	-4.05700900	-1.21316200
H	5.91999400	-3.42725800	1.11345200
H	5.94382500	-3.72709700	-3.63524300
C	-3.90434100	-0.08648800	0.01454900
C	-4.37262100	-0.87286400	1.05301300
C	-4.34441800	-0.34997200	-1.32530200
C	-5.26577200	-1.94536200	0.82146100
C	-5.24883500	-1.44055600	-1.55455900
C	-3.94071900	0.43640300	-2.43877500
C	-5.69051800	-2.22579500	-0.45599500
C	-5.69178300	-1.70602000	-2.87882500
H	-3.26844600	1.27261600	-2.27844500
C	-4.39102800	0.15399300	-3.70910700
H	-6.37443700	-3.05013400	-0.64401400
C	-5.27293700	-0.92983100	-3.93485300
H	-6.37403300	-2.53721400	-3.04130700
H	-4.06689400	0.76685800	-4.54572800
H	-5.61895300	-1.14387000	-4.94238900
H	-4.03041400	-0.66911600	2.06360500
H	-5.60978900	-2.54515500	1.65978800
H	3.36622600	-0.09507600	2.02395900
H	4.80925800	-1.96222500	2.77779400
H	4.23764700	3.80913500	-2.64287700
H	2.90547700	5.76735100	-3.36965200
H	0.50102600	5.93756900	-2.72593900
H	-0.54148500	4.21125100	-1.33081500
H	0.76925700	4.10379500	1.25219500
H	-0.04619000	5.89807700	2.70970100
H	-2.44618600	5.99297300	3.38472600
H	-4.00773000	4.22604500	2.62422400

1

E = -1131.858465 a.u.

C	-1.31793200	0.33285900	0.01788000
C	-2.72444000	0.32320800	-0.17681800

C	-3.50076900	1.48508400	-0.12172100
C	-2.84245900	2.68226900	0.13540800
C	-1.44622800	2.71873100	0.32991000
C	-0.68170900	1.55969800	0.27138100
C	-0.85152000	-1.03496200	-0.10998500
C	-1.97808600	-1.79577500	-0.36624400
H	-4.57655300	1.45492500	-0.27396400
H	-3.41356400	3.60506500	0.18616500
H	-0.96242000	3.67140500	0.52676700
H	0.39417600	1.60632800	0.41844200
H	-2.07305500	-2.86319000	-0.50780800
N	-3.09163700	-0.98904900	-0.41136700
H	-4.03414300	-1.31278700	-0.56408700
C	0.52498100	-1.47527600	0.01955200
H	1.21383000	-0.71191700	0.37758900
C	1.00884000	-2.70512300	-0.26342500
H	0.32340200	-3.45351200	-0.66131200
C	2.38979200	-3.16564800	-0.11294800
C	3.40953600	-2.39767100	0.48581800
C	2.73833800	-4.44612300	-0.58463900
C	4.71028300	-2.87661300	0.59674900
H	3.18545000	-1.41169400	0.88129100
C	4.03572300	-4.94180500	-0.47951000
H	1.97390900	-5.06509800	-1.04827400
C	5.01794100	-4.14941000	0.11119700
H	5.48244900	-2.27234700	1.06140200
H	4.28298400	-5.93058300	-0.85139500
Cl	6.66150200	-4.75931200	0.25404000

2a

E = -842.531225 a.u.

C	-5.80632300	2.09823300	-0.60560900
C	-7.18347100	2.35100600	-0.26337500
C	-7.61339000	3.50212600	0.29689800
C	-6.62508800	4.55024800	0.57129700
C	-4.82220300	2.99653900	-0.39370200
C	-7.02570200	0.29082800	-1.16059800
H	-8.64941900	3.68796600	0.55243400
H	-3.79738500	2.77000800	-0.66469400

H	-7.04577500	-0.61732900	-0.54815600
H	-7.32716900	0.07499600	-2.19146600
O	-7.91942400	1.25647700	-0.60073500
O	-5.71332500	0.84573400	-1.15159700
O	-6.95957500	5.62240800	1.08175800
C	-5.18135000	4.26635800	0.20481300
C	-4.28314200	5.26850700	0.47560200
H	-4.71308500	6.16141700	0.92661500
C	-2.87263400	5.27040000	0.23597700
H	-2.42082300	4.38909700	-0.21343500
C	-2.08987200	6.33677100	0.55302300
H	-2.58346900	7.19921300	1.00124500
C	-0.65060900	6.47712900	0.36695600
C	0.16291300	5.47026800	-0.19489700
C	-0.03115800	7.67867600	0.76661500
C	1.53161500	5.66215500	-0.34750700
H	-0.27944200	4.53133900	-0.51402900
C	1.33979000	7.87029900	0.61347500
H	-0.64142500	8.46650600	1.20183200
C	2.12820400	6.86221900	0.05526400
H	2.13934200	4.87329800	-0.78263500
H	1.79277500	8.80598000	0.92965000
H	3.19803100	7.00760400	-0.06594900

Int1

E = -4156.084226 a.u.

C	1.95091700	7.39792800	2.81110100
C	2.34179900	6.29680600	3.55889600
C	3.69160600	5.85716300	3.66280800
C	4.64150300	6.54617000	2.93463000
C	4.30129800	7.62559900	2.08190200
C	2.93731800	8.05801100	2.00051200
C	0.52373300	7.83250900	2.83601200
C	-0.47422800	6.92338500	2.51721200
C	-1.85324700	7.26929800	2.44915300
C	-2.20584500	8.56396600	2.76812700
C	-1.24782300	9.52337800	3.18346900
C	0.13726500	9.15947200	3.23423100
H	5.68288000	6.24309000	3.00351900

H	-3.25181400	8.85505100	2.71716200
C	5.28856300	8.27631000	1.29223000
C	4.94844300	9.29306100	0.43061800
C	3.59648200	9.69660100	0.31926200
C	2.61867900	9.09767900	1.08280000
C	1.06305800	10.12236900	3.72477000
C	0.64742300	11.37946600	4.10581200
C	-0.71603600	11.74738900	4.01772200
C	-1.64137600	10.83349400	3.56931400
O	1.37346800	5.59898400	4.27777600
O	-0.12441000	5.60912000	2.21953100
P	0.36846200	4.62958600	3.43832600
O	0.94195300	3.38734300	2.87190700
O	-0.79870000	4.51914400	4.49139300
H	-1.44218600	3.76034200	4.26114800
C	4.08232900	4.73074200	4.56038900
C	4.69576900	3.54192100	4.04008700
C	3.89420100	4.85912900	5.92555200
C	4.85651200	3.29643300	2.64741300
C	5.14257600	2.53529300	4.96174100
C	4.32339800	3.85860900	6.82593200
C	5.44975600	2.13986500	2.19261500
H	4.48404600	4.02581100	1.93698100
C	5.76028200	1.35862300	4.45755400
C	4.94567900	2.72576400	6.35477100
C	5.91670800	1.16383400	3.10504100
H	5.54766100	1.96903900	1.12414000
H	6.09431800	0.60471900	5.16641000
H	5.28866300	1.95492300	7.04091200
H	6.38269500	0.25535700	2.73313100
C	-2.90052100	6.27042100	2.07787200
C	-3.75425800	5.79824200	3.05740100
C	-3.06829800	5.83253500	0.72299300
C	-4.78399900	4.87699700	2.75528600
C	-4.11776000	4.90144900	0.42089000
C	-2.25322100	6.30071800	-0.34405500
C	-4.96204500	4.43760800	1.46526500
C	-4.29645500	4.47428700	-0.92383100
H	-1.46583000	7.01612800	-0.12913000
C	-2.45187600	5.86792200	-1.63686600

H	-5.75185100	3.72968200	1.22470400
C	-3.48335000	4.94368700	-1.93155400
H	-5.09947100	3.77398500	-1.14353200
H	-1.81323900	6.23220000	-2.43602600
H	-3.63714200	4.61693500	-2.95676300
H	-3.61434200	6.12786500	4.08257500
H	-5.42742200	4.51616600	3.55259400
H	3.41883700	5.75532100	6.31319500
H	4.16765400	3.99799500	7.89234500
H	6.32007800	7.94229900	1.37459600
H	5.71020300	9.77787300	-0.17364200
H	3.32560200	10.48287700	-0.38013100
H	1.58690600	9.41235700	0.97804900
H	2.10963800	9.85440200	3.80901700
H	1.37417100	12.09374900	4.48328000
H	-1.02907800	12.74428400	4.31558700
H	-2.69541100	11.09496900	3.51341600
C	2.08828900	-0.81959300	3.59229900
C	2.36961500	-2.14118700	4.03169200
C	2.54619600	-2.46389100	5.38207100
C	2.43405900	-1.42737900	6.30560700
C	2.15230100	-0.10815100	5.89122900
C	1.98098800	0.20652400	4.54719200
C	1.98345000	-0.85286200	2.14532200
C	2.19802900	-2.17008500	1.78392100
H	2.77708400	-3.47897900	5.69676400
H	2.57712700	-1.63713000	7.36274100
H	2.08134700	0.67933800	6.63588300
H	1.77379200	1.22819200	4.24070500
H	2.20164300	-2.62698800	0.80418600
N	2.42855900	-2.93883400	2.90514900
H	2.61416700	-3.92947400	2.89913700
C	1.71146800	0.29140600	1.29048800
H	1.50586500	1.22221300	1.81453300
C	1.70435300	0.27864000	-0.06139300
H	1.93203500	-0.65552800	-0.57681800
C	1.42929500	1.40605800	-0.95307800
C	1.07325100	2.69311300	-0.49563300
C	1.52092100	1.20984700	-2.34472900
C	0.82700000	3.73116100	-1.38938900

H	0.98651400	2.89674400	0.56760400
C	1.27885800	2.24018700	-3.25074400
H	1.79035900	0.22718900	-2.72538300
C	0.93379500	3.49814800	-2.76218100
H	0.54733600	4.71087000	-1.01778600
H	1.35870600	2.07016500	-4.31945400
Cl	0.62941800	4.81333600	-3.90095900
C	-1.84062300	-1.36391300	4.44946100
C	-1.68226500	-0.37512300	5.47936400
C	-1.81818700	0.95882700	5.26537800
C	-2.13432000	1.39592800	3.92409200
C	-2.12866000	-1.03790700	3.17153500
C	-1.24084300	-2.39600100	6.34856700
H	-1.69511400	1.69362600	6.05173500
H	-2.22875800	-1.80187700	2.40997500
H	-0.18087100	-2.65262600	6.44780800
H	-1.86961300	-2.98008800	7.02604200
O	-1.40640600	-1.00130300	6.64560300
O	-1.66755600	-2.60393100	5.00447300
O	-2.29543100	2.61203900	3.65314300
C	-2.26644000	0.36590100	2.84726200
C	-2.45508700	0.84352400	1.56838800
H	-2.50133100	1.92669900	1.47956500
C	-2.57260200	0.09251300	0.36104500
H	-2.56307900	-0.99343800	0.41184300
C	-2.66375500	0.71497700	-0.84745600
H	-2.63116100	1.80394400	-0.85040700
C	-2.77709200	0.09503500	-2.16023900
C	-2.98273500	-1.28792600	-2.35072100
C	-2.68039600	0.91655600	-3.30206600
C	-3.08082600	-1.82226800	-3.63055300
H	-3.07799800	-1.94469500	-1.49110400
C	-2.77531000	0.37910000	-4.58324800
H	-2.51664000	1.98286400	-3.17024300
C	-2.97630300	-0.99244600	-4.75283300
H	-3.24344100	-2.88922100	-3.75762800
H	-2.69213800	1.03008900	-5.44910700
H	-3.05444000	-1.41416800	-5.75116800

TS-1

E = -4156.051574 a.u.

C	2.14914600	7.58291800	3.13553100
C	2.82041000	6.47247700	3.62677500
C	4.23969000	6.36526300	3.64693500
C	4.96632000	7.39685500	3.08596900
C	4.34252800	8.51663300	2.48220200
C	2.91312400	8.61760600	2.49415200
C	0.66273600	7.64880000	3.24131900
C	-0.10469500	6.62310200	2.70940400
C	-1.52756100	6.64928800	2.68233500
C	-2.16085500	7.71955000	3.27941100
C	-1.43973900	8.75550400	3.92558800
C	-0.00718200	8.72519000	3.91950500
H	6.05197300	7.34676700	3.09785600
H	-3.24670400	7.76591700	3.26590100
C	5.11040100	9.53607900	1.85562100
C	4.50009900	10.60212600	1.23674800
C	3.08744200	10.68642100	1.21470400
C	2.31557000	9.72313100	1.82638900
C	0.68590000	9.74535000	4.62915300
C	0.00263900	10.75202000	5.27578800
C	-1.41137100	10.79823300	5.24914800
C	-2.11368100	9.81606300	4.58969300
O	2.07908600	5.42358900	4.16191300
O	0.53154300	5.52800700	2.13881400
P	1.28978200	4.44675200	3.11537100
O	2.13022700	3.55468700	2.27963500
O	0.23320700	3.81021800	4.07974000
H	-0.17887700	2.92955700	3.67813500
C	4.93839400	5.22497000	4.31121200
C	5.78156100	4.33127200	3.57001700
C	4.83029200	5.07913100	5.68303300
C	5.88905100	4.36718900	2.15158200
C	6.53639200	3.33956200	4.28381200
C	5.56595400	4.09340900	6.37922200
C	6.71696100	3.49579400	1.47952300
H	5.29043300	5.08116000	1.59724100
C	7.38811000	2.46241100	3.55853700
C	6.41274300	3.25041400	5.69576400

C	7.48294800	2.53928600	2.18817500
H	6.77539600	3.53562800	0.39523200
H	7.96036400	1.72051800	4.11094700
H	6.99663900	2.50132600	6.22584500
H	8.13530200	1.86001500	1.64588300
C	-2.32999100	5.56058700	2.04852400
C	-3.07362500	4.71860400	2.85443700
C	-2.39627700	5.41524500	0.62286300
C	-3.89762500	3.70944800	2.30366900
C	-3.24355400	4.39803200	0.06877500
C	-1.67593200	6.25343200	-0.27211200
C	-3.98407100	3.55434200	0.94030900
C	-3.33120700	4.26451900	-1.34435900
H	-1.03980000	7.03325700	0.13336600
C	-1.77742400	6.09272700	-1.63678000
H	-4.61702300	2.78226900	0.50932800
C	-2.61316900	5.08834100	-2.18145100
H	-3.98646900	3.49835000	-1.75321400
H	-1.21666200	6.74424500	-2.30157200
H	-2.68770400	4.97476200	-3.25951600
H	-3.00842800	4.82783700	3.93289400
H	-4.45790800	3.05697000	2.96731600
H	4.18312400	5.75159000	6.23833200
H	5.47040800	4.02015200	7.45932000
H	6.19420200	9.44818900	1.86587100
H	5.09664800	11.37204600	0.75523900
H	2.60605400	11.51690100	0.70536300
H	1.23500800	9.79845800	1.79178400
H	1.76862700	9.71922800	4.66863700
H	0.55581300	11.51521900	5.81655200
H	-1.93735400	11.60112300	5.75832000
H	-3.20095100	9.82845900	4.57464600
C	1.97958300	-0.21416000	3.98631200
C	1.79006700	-1.33471100	4.82616300
C	2.38201800	-1.44924800	6.08349100
C	3.18775500	-0.39107100	6.49753400
C	3.38915800	0.73613200	5.67962900
C	2.79311200	0.83851200	4.42433100
C	1.21108500	-0.45419200	2.76837600
C	0.59668400	-1.70332500	2.94688700

H	2.22165100	-2.32021400	6.71313300
H	3.66700200	-0.43803700	7.47148100
H	4.02075900	1.54563200	6.03189600
H	2.94897900	1.71565700	3.80320400
H	-0.05374100	-2.25170800	2.28356900
N	0.94452000	-2.21819700	4.15086100
H	0.60736100	-3.09501300	4.52042900
C	1.02153600	0.42048900	1.68465600
H	1.53472400	1.37659300	1.71955400
C	0.24984100	0.08721600	0.52676800
H	0.19628100	-0.98286900	0.32855600
C	0.49635700	0.88414900	-0.71090500
C	0.64058300	2.28166300	-0.67147300
C	0.55727600	0.24446600	-1.95786900
C	0.84195400	3.01463700	-1.83952800
H	0.60744600	2.81100400	0.27539300
C	0.76410100	0.96366800	-3.13516500
H	0.44471900	-0.83518900	-2.01368200
C	0.90133900	2.34826100	-3.06339100
H	0.94379700	4.09319300	-1.79546000
H	0.81450400	0.45679100	-4.09296900
Cl	1.15021300	3.27103700	-4.54441400
C	-3.06306200	-1.32046600	3.98817200
C	-2.54407900	-0.45688800	4.97125100
C	-1.73633100	0.60806100	4.67299200
C	-1.45119500	0.84894100	3.28793700
C	-2.79785500	-1.14997200	2.65553200
C	-3.94322000	-1.89349800	5.95817800
H	-1.33118000	1.26506400	5.43412500
H	-3.23661200	-1.81402900	1.91932100
H	-3.76244600	-2.74455000	6.62028600
H	-4.94655400	-1.47418900	6.12418500
O	-2.96132000	-0.88270200	6.20822400
O	-3.81112600	-2.30944000	4.60053500
O	-0.70413400	1.81828100	2.88894600
C	-1.98201900	-0.04468300	2.27755300
C	-1.66910200	0.32185800	0.90637800
H	-1.61494900	1.40112200	0.78565000
C	-2.34637000	-0.37388300	-0.19862200
H	-2.53098100	-1.44081200	-0.08213200

C	-2.70117700	0.23811800	-1.34700400
H	-2.45907500	1.29473600	-1.45198600
C	-3.37581000	-0.36204300	-2.50358300
C	-3.98133800	-1.63350200	-2.47344300
C	-3.42882700	0.36630700	-3.70691600
C	-4.59922200	-2.15735600	-3.60529500
H	-3.98357200	-2.20890000	-1.55196800
C	-4.04606900	-0.15866500	-4.84120000
H	-2.96539100	1.34904800	-3.74852800
C	-4.63332400	-1.42422800	-4.79600000
H	-5.06378700	-3.13890400	-3.55838400
H	-4.06850500	0.42128200	-5.75998100
H	-5.11924300	-1.83482300	-5.67686100

Int1’

E = -4156.082175 a.u.

C	2.29401800	7.36606000	2.37659800
C	3.14317700	6.37336300	2.84378800
C	4.55786200	6.40329100	2.67047400
C	5.08258800	7.44823000	1.93428200
C	4.26276700	8.43374400	1.33085000
C	2.84569700	8.39594500	1.53863600
C	0.84486200	7.33114600	2.73169100
C	0.09188500	6.20040300	2.45738500
C	-1.30987500	6.11185000	2.68932200
C	-1.92699800	7.19779300	3.27468100
C	-1.20460500	8.35335800	3.66678400
C	0.20314000	8.42751400	3.40871200
H	6.15917700	7.51554100	1.80379000
H	-2.99801400	7.16186200	3.45578900
C	4.82224000	9.45527700	0.51525500
C	4.01984400	10.38689900	-0.10149000
C	2.61623700	10.32876700	0.07018100
C	2.04492200	9.36272400	0.86928900
C	0.90880300	9.56925700	3.88070600
C	0.25451900	10.59115000	4.53372900
C	-1.14215300	10.53215300	4.75108900
C	-1.85186500	9.43202700	4.32839500
O	2.60234400	5.31720900	3.57253200

O	0.71621800	5.09191300	1.89085200
P	1.72635100	4.18606500	2.78900100
O	2.46027800	3.23218600	1.92495700
O	0.89842600	3.60760500	3.99860800
H	0.91130600	2.60719700	4.09840300
C	5.44960200	5.40170100	3.32391700
C	6.40507400	4.63762000	2.57106100
C	5.39690100	5.25322200	4.70025100
C	6.48425500	4.66939900	1.15000100
C	7.31274300	3.77637900	3.27750300
C	6.28155600	4.39287400	5.38613700
C	7.42270900	3.92488100	0.47039900
H	5.77825200	5.27876700	0.59700600
C	8.27365000	3.02808900	2.54383500
C	7.22892800	3.67830800	4.69081600
C	8.33513700	3.10126300	1.17190400
H	7.45530200	3.95825200	-0.61489200
H	8.95830800	2.38596400	3.09353100
H	7.92315000	3.02322500	5.21213700
H	9.06704800	2.51585600	0.62259000
C	-2.08749100	4.88298400	2.35571500
C	-2.64167200	4.14737100	3.38811500
C	-2.30019600	4.46990400	0.99858300
C	-3.39842300	2.97901600	3.13820000
C	-3.06426100	3.28092600	0.75080500
C	-1.80348300	5.19650800	-0.11802000
C	-3.59830600	2.55133100	1.84656300
C	-3.27621800	2.86156900	-0.59056200
H	-1.23761700	6.10635900	0.05306100
C	-2.03130500	4.76520200	-1.40585700
H	-4.16187500	1.64377500	1.64673000
C	-2.77089400	3.58290100	-1.64745800
H	-3.84609800	1.95213700	-0.76501300
H	-1.63793000	5.33437300	-2.24384000
H	-2.93681800	3.24853800	-2.66793200
H	-2.46727200	4.46280200	4.41272600
H	-3.80343600	2.41516300	3.97354200
H	4.66834600	5.82435100	5.26704800
H	6.21419900	4.31093000	6.46751200
H	5.90081600	9.47637100	0.37859800

H	4.45761000	11.15852400	-0.72874000
H	1.98177300	11.05035300	-0.43727000
H	0.96774000	9.32690400	0.98329100
H	1.98071000	9.62735600	3.73338500
H	0.81905500	11.44850400	4.89024000
H	-1.64675100	11.34798200	5.26131400
H	-2.92261700	9.36287500	4.50481900
C	-3.14296700	-0.79847600	5.13179100
C	-4.12168500	-1.61702500	5.75529200
C	-4.59472500	-1.37158100	7.04882000
C	-4.06914200	-0.27660800	7.72542800
C	-3.09586900	0.55116800	7.12742200
C	-2.62898400	0.30293200	5.84046300
C	-2.90114200	-1.33796300	3.80678000
C	-3.72505700	-2.44262900	3.69445700
H	-5.34514100	-2.01030600	7.50786500
H	-4.41436600	-0.05535300	8.73165300
H	-2.70609600	1.39912100	7.68414300
H	-1.86872400	0.94033400	5.39693900
H	-3.84460200	-3.13513800	2.87301600
N	-4.45345200	-2.60946900	4.85266600
H	-5.11685900	-3.35036500	5.01707500
C	-1.98302100	-0.77816300	2.82980900
H	-1.35527700	0.02530800	3.20737500
C	-1.85608700	-1.15278200	1.53734600
H	-2.48817500	-1.95775200	1.16005500
C	-0.95669600	-0.56724700	0.54099000
C	-0.94499800	-1.09415000	-0.76465000
C	-0.10555100	0.52513400	0.80852700
C	-0.13041600	-0.56403900	-1.76317800
H	-1.58964700	-1.93752400	-1.00246000
C	0.70842000	1.07283800	-0.17763800
H	-0.07420100	0.96218000	1.80114100
C	0.68928600	0.52079700	-1.45981200
H	-0.13379500	-0.98444900	-2.76352800
H	1.33983000	1.92384700	0.05579900
Cl	1.71832100	1.20586600	-2.71832200
C	2.91454900	-2.09023700	6.01875200
C	1.69796000	-1.63513900	6.62041200
C	0.96426000	-0.59894700	6.13863700

C	1.44173200	0.08210700	4.95218500
C	3.42505900	-1.51594000	4.90869700
C	2.48338400	-3.37342000	7.81216800
H	0.03826900	-0.27537100	6.59702200
H	4.35004700	-1.87373300	4.47285300
H	2.98834400	-3.26035400	8.77743300
H	2.04846800	-4.37427900	7.71155400
O	1.43850300	-2.39742800	7.71321300
O	3.40746300	-3.13914300	6.75450300
O	0.77227400	1.03411200	4.47082900
C	2.70878000	-0.40052700	4.32480200
C	3.13339300	0.24167300	3.17763400
H	2.54168600	1.08982300	2.84647900
C	4.24918000	-0.09218400	2.35616800
H	4.85834400	-0.95627800	2.60970300
C	4.52060900	0.62632900	1.22848900
H	3.87734000	1.47932100	1.01996500
C	5.57056100	0.36632200	0.25463900
C	6.61390700	-0.55987000	0.46552000
C	5.53784300	1.06574700	-0.96907800
C	7.57769700	-0.77992000	-0.51206200
H	6.67726900	-1.09862100	1.40622400
C	6.49851200	0.83722400	-1.95121300
H	4.73732300	1.77833400	-1.14700100
C	7.52300400	-0.08497100	-1.72605000
H	8.37636600	-1.49427500	-0.33073800
H	6.44685000	1.37712500	-2.89267500
H	8.27550300	-0.26309500	-2.48956700

TS-1’

E = -4156.043350 a.u.

C	2.20721600	7.40861900	2.70363600
C	2.94890300	6.33691800	3.17872100
C	4.35419700	6.20761900	2.98682200
C	4.98570600	7.18352700	2.24142800
C	4.27520400	8.25732200	1.64891300
C	2.86317000	8.37476200	1.86520800
C	0.75562800	7.50264800	3.03740300
C	-0.08658000	6.43951300	2.74439700

C	-1.49587300	6.48152700	2.94667700
C	-2.02696700	7.61683100	3.52357400
C	-1.21304500	8.70224500	3.93510300
C	0.20034500	8.64973400	3.70475400
H	6.06019500	7.12206900	2.09022600
H	-3.10080600	7.67810900	3.68002300
C	4.93811500	9.21212000	0.83006600
C	4.23975600	10.22743700	0.21882800
C	2.83928900	10.32327100	0.39856100
C	2.16995100	9.42399600	1.19949200
C	0.99623200	9.72358400	4.19261900
C	0.42359200	10.79932500	4.83556300
C	-0.97684900	10.86535500	5.02636200
C	-1.77407500	9.83385800	4.58698600
O	2.29949000	5.35144800	3.91415200
O	0.44288100	5.28456600	2.18661600
P	1.37394900	4.28381900	3.09221300
O	2.07161300	3.32452000	2.20856700
O	0.47424200	3.73760800	4.25915900
H	0.39785100	2.71239300	4.27008900
C	5.12797300	5.09536900	3.61247300
C	5.87226600	4.16109100	2.81687400
C	5.16545100	4.99487600	4.99257400
C	5.83483000	4.14876000	1.39417000
C	6.67343600	3.17138900	3.48143400
C	5.94665600	4.01224000	5.63973500
C	6.56612200	3.23306000	0.67084700
H	5.20227400	4.86257900	0.87857400
C	7.42089800	2.24636800	2.70229600
C	6.69434000	3.12599800	4.89991000
C	7.37422100	2.27433100	1.32798700
H	6.51495100	3.23673700	-0.41446800
H	8.02597900	1.50457000	3.21883300
H	7.30572800	2.37175100	5.39004800
H	7.93993900	1.55405300	0.74377000
C	-2.38350800	5.33677600	2.58672800
C	-3.06547000	4.68411400	3.59787300
C	-2.59054200	4.93594000	1.22363500
C	-3.95710800	3.62160400	3.32042100
C	-3.48949700	3.85129300	0.94825000

C	-1.96386500	5.58476500	0.12435700
C	-4.16069100	3.20914500	2.02403000
C	-3.70319900	3.45221900	-0.39919000
H	-1.29534900	6.41737500	0.31433500
C	-2.19658300	5.17651000	-1.17093600
H	-4.84669600	2.39498900	1.80118900
C	-3.07131900	4.09584000	-1.43841900
H	-4.38220100	2.62538200	-0.59442200
H	-1.70707300	5.68860700	-1.99494000
H	-3.24156600	3.77946100	-2.46370100
H	-2.89665300	4.98565300	4.62721400
H	-4.48003400	3.13758400	4.14112400
H	4.59077400	5.69578000	5.59060700
H	5.95769900	3.96938600	6.72547200
H	6.01158700	9.11434500	0.68655800
H	4.75615400	10.94720900	-0.41030100
H	2.28489500	11.11108500	-0.10431700
H	1.09593400	9.50662000	1.31879700
H	2.07158900	9.68470500	4.06484700
H	1.05545100	11.60261000	5.20482300
H	-1.41598900	11.72262900	5.52912900
H	-2.85003000	9.86117600	4.74238000
C	-2.19600200	-1.02984000	4.57752100
C	-2.17989500	-2.08364700	5.52063100
C	-3.12880900	-2.20444000	6.53654000
C	-4.11692300	-1.22559400	6.59986800
C	-4.14721700	-0.16217000	5.67807400
C	-3.19507800	-0.05280900	4.66820700
C	-1.06887400	-1.23790900	3.67804600
C	-0.42522900	-2.39164200	4.13727600
H	-3.09785200	-3.02277300	7.25060500
H	-4.87378600	-1.28216800	7.37693700
H	-4.92768400	0.58899400	5.76227200
H	-3.22048200	0.78158100	3.97350200
H	0.45639100	-2.88540800	3.75840400
N	-1.08768500	-2.89242400	5.21412400
H	-0.79801500	-3.70207100	5.74231800
C	-0.64418900	-0.40133900	2.62344300
H	-1.25875100	0.46130000	2.39487900
C	0.45977200	-0.69377500	1.76945500

H	0.72097100	-1.75088100	1.73859400
C	0.45389400	-0.06891800	0.41304300
C	0.69496600	-0.85561800	-0.72259900
C	0.21427000	1.30239000	0.23344600
C	0.67427300	-0.30580500	-2.00412700
H	0.89617800	-1.91793100	-0.60789800
C	0.19054800	1.86878100	-1.03976400
H	0.07900300	1.94912400	1.09338400
C	0.41553200	1.05575000	-2.15002300
H	0.85631800	-0.92487900	-2.87618700
H	0.00332800	2.92984900	-1.16097000
Cl	0.38099000	1.76092300	-3.76525000
C	2.84184200	-1.44592900	6.05206400
C	1.92589500	-0.68272400	6.80100500
C	1.06433500	0.21343700	6.22707600
C	1.15021700	0.39322600	4.80634800
C	2.94166900	-1.33674400	4.69044500
C	3.21556500	-1.83198100	8.21857900
H	0.35118900	0.79319700	6.80124300
H	3.68146200	-1.91057600	4.14379700
H	4.04285300	-1.23315700	8.62721600
H	3.00869900	-2.69653200	8.85512800
O	2.04181400	-1.01907200	8.12845800
O	3.54478900	-2.27657700	6.90397100
O	0.36652000	1.19596500	4.17019300
C	2.09721100	-0.39179000	4.04055000
C	2.13978200	-0.09276100	2.62158400
H	1.94876300	0.95979800	2.42168100
C	3.19125000	-0.68030200	1.77984600
H	3.46827600	-1.71794000	1.96277300
C	3.78366900	0.00602800	0.78130700
H	3.45714400	1.03115800	0.61250500
C	4.83263400	-0.47859600	-0.12237200
C	5.56792000	-1.65787000	0.10638700
C	5.12514700	0.26760400	-1.27949300
C	6.54356900	-2.07818900	-0.79329800
H	5.38754300	-2.23963800	1.00590800
C	6.09941800	-0.15444900	-2.18285300
H	4.56867300	1.18165600	-1.46982300
C	6.81333300	-1.33045900	-1.94415600

H	7.10233100	-2.98911200	-0.59405700
H	6.30088100	0.43520400	-3.07335000
H	7.57675400	-1.66001600	-2.64377000
